# On–DNA Platform Molecules Based on a Diazide Scaffold II: A Compact Diazide Platform Designed for Small–Molecule Drug Discovery

**DOI:** 10.3390/ijms27020828

**Published:** 2026-01-14

**Authors:** Hiroyuki Miyachi, Masaki Koshimizu, Masashi Suzuki

**Affiliations:** Lead Exploration Unit, Drug Discovery Initiative, University of Tokyo, 7-3-1 Hongo, Bunkyo, Tokyo 113-0033, Japan

**Keywords:** DNA encoded library, DEL, chemical space, on–DNA diazide platform, reactivity hierarchy, the number of Ro5VC, QED

## Abstract

Expanding the chemical diversity of DNA–encoded libraries (DELs) is crucial for identifying binders to emerging drug targets using DEL technology. In the present study, as part of our ongoing efforts to develop on–DNA diazide platforms (D–DAPs)—platform molecules possessing both aromatic and aliphatic azide groups on a single core reactive scaffold—we designed and synthesized a new compact diazide platform, designated as a compact D–DAP (C–D–DAP). This molecule is based on a low–molecular–weight reactive scaffold, 3–azido–5–(azidomethyl)benzoic acid, to facilitate small–molecule drug discovery targeting enzymes and G protein–coupled receptors (GPCRs). Furthermore, we established two distinct stepwise warhead construction strategies that exploit the chemoselective transformations of the azide groups in the C–D–DAP, which exhibit different reactivities. In addition, four virtual DELs were generated based on stepwise warhead elaboration from the C–D–DAP scaffold. Comparative chemical diversity analysis against bioactive compounds from ChEMBL revealed that these virtual libraries populate structural regions that are sparsely represented among known molecules. Each virtual library also occupies a distinct region of structural space relative to the others and displays intermediate quantitative estimate of drug–likeness (QED) values.

## 1. Introduction

DNA–encoded library (DEL) technology has recently undergone rapid development as a powerful approach to hit discovery for therapeutic targets [1]. In DEL technology, small–molecule compounds (warhead) are covalently linked to DNA tags, allowing their structural information to be recorded as DNA sequences [2]. This approach enables the integrated synthesis and analysis of libraries comprising millions to hundreds of millions of compounds. Owing to this unique feature, DELs have attracted considerable attention as a discovery platform for previously unidentified compounds that surpasses conventional high–throughput screening (HTS) [3].

To further advance the application of DELs in drug discovery, it is essential to expand the chemical space occupied by library compounds and explore regions that remain uncharted in traditional medicinal chemistry [4]. Achieving this goal requires the development of platform molecules that can facilitate diverse structural transformations during library design. Such platform molecules play a crucial role in enhancing structural diversity and synthetic efficiency in DELs through controlled reactivity and modular design [5].

Previously, we proposed an on-DNA diazido platform (D–DAP) that incorporates both aromatic and aliphatic azide groups within a single molecule [6]. We reported the synthesis and DEL applicability of three representative D–DAPs based on homoalanine (**1**), lysine (**2**), and proline (**3**) scaffolds. In particular, a virtual library constructed using the proline-based D–DAP (**3**) was analyzed to assess its chemical characteristics. The resulting library mainly occupied the extended rule of 5 (eRo5) region, consisting of compounds with an average molecular weight of approximately 650, situated between small molecules (rule of 5–compliant) and middle molecules (beyond rule of 5). Furthermore, the calculated clogP values ranged from 2.4 to 4.4, indicating physicochemical properties suitable for interacting with hydrophobic protein–protein interaction (PPI) interfaces. These findings suggest that this DEL design may be effective for generating novel binders against challenging targets.

Meanwhile, the identification of small-molecule binders targeting conventional drug discovery targets, such as enzymes and G protein–coupled receptors (GPCRs), remains of great importance. However, in our previously reported D–DAP–derived library, compounds with molecular weights below 500 accounted for only 1.1% (439 out of 40,000), indicating insufficient coverage of the low–molecular–weight chemical space.

**Figure 1:**
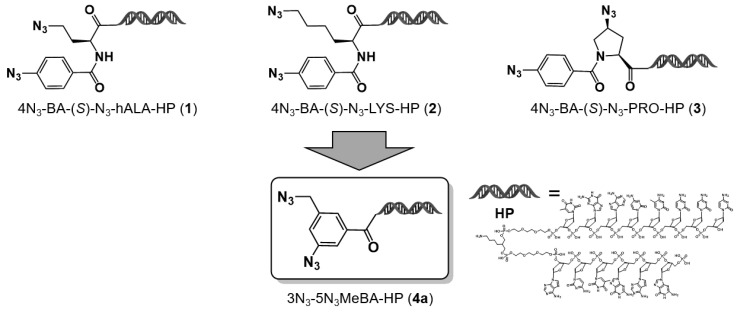
Design and derivation of the compact diazide platform molecule (**4a**) from on–DNA diazide intermediates. Compounds **1**–**3**, prepared from hAla, Lys, and Pro precursors, were convergently transformed into the compact diazide scaffold **4a**. HP = DNA headpiece.

To address this limitation, we designed and synthesized a newly described compact-type D–DAP (Compact D–DAP: C–D–DAP) as a novel platform molecule enabling the construction of libraries with smaller warheads. The C–D–DAP is based on 3–azido–5–(azidomethyl)benzoic acid–DNA headpiece (HP) (**4a**), corresponding to the minimal structural unit of the DAP family. The study by Hosoya and colleagues established the utility of compound 3–azido–5–(azidomethyl)benzoic acid (**5**) in chemical biology [7]; however, its potential in DEL construction has yet to be realized (Figure 1).

## 2. Results and Discussion

### 2.1. Organocatalyzed [3+2] Cycloaddition Reactions of 3–Azido–5–(Azidomethyl)Benzoic Acid–HP

**Figure 2:**
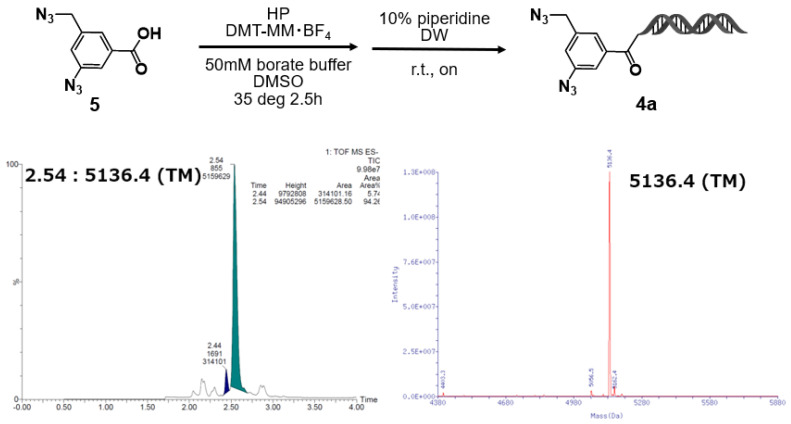
Synthesis of on–DNA diazide conjugate via DMTMM−mediated amidation and its LC/MS characterization. DMTMM−mediated amidation of **5** to HP followed by the removal of the DMT−adduct yielded the on−DNA conjugate. LC/MS showed the expected mass of 5136.4 (Target molecule: (TM)).

The starting material, 3–azido–5–(azidomethyl)benzoic acid–HP (**4a**), was synthesized by coupling 3–azido–5–(azidomethyl)benzoic acid (**5**) with the HP [sequence: 5′–/5Phos/GAGTCA/iSp9/iUniAmM/iSp9/TGACTCCC–3′] using 4–(4,6–dimethoxy–1,3,5–triazin–2–yl)–4–methylmorpholinium tetrafluoroborate (DMT–MM BF_4_) as a condensation reagent [8]. The reaction was carried out at room temperature for 2.5 h. After completion, the desired product (**4a**) was obtained in nearly quantitative yield by ethanol precipitation followed by piperidine treatment (Figure 2). The synthesis was performed on the nanomolar scale, and to avoid losses during purification, the resulting crude product was used directly in the subsequent reaction without further purification.

**Figure 3:**
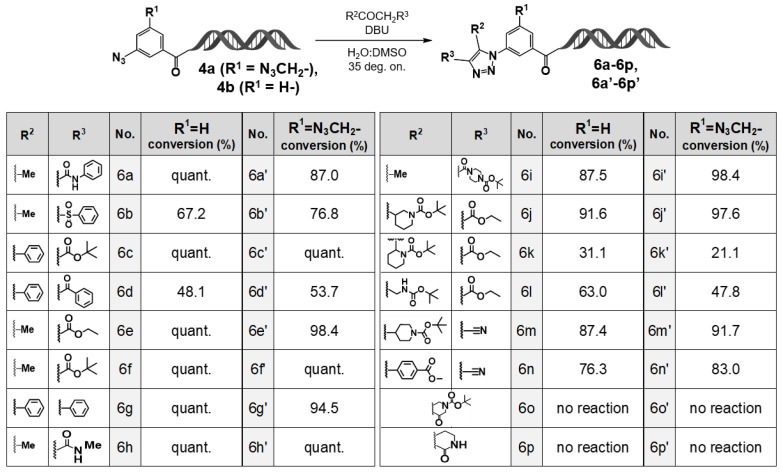
Cycloaddition reactions of 3–azido–5–(azidomethyl)benzoic acid–HP. Organocatalyzed [3+2] cycloaddition reactions of 3–azido–5–(azidomethyl)benzoic acid–HP with various active methylene compounds. Substrate scope and conversion profiles of the on-DNA organocatalyzed [3+2] cycloaddition leading to triazole products (**6a**–**6p**, **6a′**–**6p′**). DBU-mediated on–DNA [3+2] cycloaddition was performed with diverse carbonyl substrates. LC/MS conversion values are shown for R^3^ = H (**6a**–**6p**) and R^3^ = N_3_CH_2_– (**6a′**–**6p′**). “quant.” = >95% conversion.

In the case of 3–azidobenzoic acid–HP (**4b**), previous studies by Xu et al. and our group have demonstrated that the corresponding 1,4,5–trisubstituted 1,2,3–triazole derivatives can be obtained quantitatively via organocatalytic [3+2] cycloaddition reactions [9]. By contrast, to our knowledge, no reports have previously described such a reaction using 3–azido–5–(azidomethyl)benzoic acid–HP (**4a**) as a substrate. Therefore, in the present study, we aimed to elucidate the effect of the azidomethyl substituent at the 5–position on the [3+2] cycloaddition reactivity of the 3–position azide group. To this end, both 3–azido–5–(azidomethyl)benzoic acid–HP (**4a**) and 3–azidobenzoic acid–HP (**4b**) were subjected to organocatalytic [3+2] cycloaddition reactions with various active methylene compounds (Figure 3).

First, the reaction of 3–azidobenzoic acid–HP (**4b**) with active methylene compounds was conducted using 1,8–diazabicyclo[5.4.0]undec–7–ene (DBU) as the organic base catalyst at 35 deg overnight. As a result, when β–ketoamides (**6a**, **6h**), β–ketoesters (**6c**, **6e**, **6f**), or phenylbenzyl ketone (**6g**) were used as substrates, the corresponding 1,4,5–trisubstituted 1,2,3–triazole derivatives were obtained quantitatively. By contrast, reactions with β–ketosulfone (**6b**) or 1,3–diketone (**6d**) afforded the desired triazole products in moderate yields.

To further expand structural diversity, we investigated reactions employing active methylene compounds (**6i**–**6p**) bearing functional substituents at the R_2_ and R_3_ positions. High yields of the desired triazole products were obtained with **6i**, **6j**, **6m**, and **6n**. However, when tert–butyl 2–(3–ethoxy–3–oxopropanoyl)piperidine–1–carboxylate (**6k**) was used as a substrate, the reaction afforded the desired product in low yield. Moreover, reactions with cyclic active methylene compounds **6o** and **6p** did not proceed under the same conditions.

Interestingly, in the reaction using **6k**, the major product was identified as the corresponding amine resulting from the reduction of the azide group. It has been reported that diazo-transfer reactions (Regitz–diazo transfer [10]) can compete with organocatalytic [3+2] cycloadditions under certain reaction conditions, suggesting that such a competing process likely occurred in this case as well. A comparison of the regioisomeric substrates **6j** and **6k** further supports this hypothesis: although **6j** afforded the 1,2,3–triazole product in high yield, diazo transfer predominated in the reaction with **6k**. These results suggest that a bulky substituent adjacent to the carbonyl group in the active methylene compound sterically hinders nucleophilic attack by the terminal nitrogen of the azide on the carbonyl and thereby favors the diazo–transfer pathway.

The products obtained from each reaction were analyzed using quadrupole time–of–flight mass spectrometry (QTOF–MS). In all reaction systems, mass peaks corresponding to species in which only one azide group had reacted were observed, whereas no signals corresponding to products containing two 1,2,3–triazole rings—arising from simultaneous [3+2] cycloaddition at both azide sites—were detected. (To the best of our knowledge, this is the first report of an organocatalyzed [3+2] cycloaddition performed on an on–DNA substrate bearing both an aromatic azide and an azidomethyl group on a benzene ring. While base-catalyzed [3+2] cycloadditions have been reported for on–DNA substrates containing either an aromatic azide or an azidomethyl substituent [11], and base–catalyzed [3+2] cycloadditions of off–DNA azidobenzene or azidomethylbenzene derivatives with active methylene compounds are known [12], the combined on–DNA system has not been described previously).

Subsequently, we investigated the organocatalytic [3+2] cycloaddition reactions using 3–azido–5–(azidomethyl)benzoic acid–HP (**4a**) as the substrate. The results revealed that **4a** exhibited reactivity similar to that of the structurally related 3–azidobenzoic acid–HP (**4b**). Specifically, when β–ketoamides (**6a′**, **6h′**), β–ketoesters (**6c′**, **6e′**, **6f′**), and phenylbenzyl ketone (**6g′**) were employed as active methylene compounds, the corresponding 1,4,5–trisubstituted 1,2,3–triazole derivatives were obtained in high yields. By contrast, reactions with β–ketosulfone (**6b′**) and 1,3–diketone (**6d′**) afforded the desired 1,2,3–triazole products in moderate yields.

Reactions using active methylene compounds bearing structural diversity–enhancing functional groups at the R_2_ and R_3_ positions (**6i′**–**6p′**) were also examined. High yields of the desired 1,2,3–triazole products were obtained with **6i′**, **6j′**, **6m′**, and **6n′**, whereas the reaction with **6k′** proceeded with low yield. Moreover, reactions with cyclic active methylene compounds **6o′** and **6p′** did not proceed, and in the reaction with **6k′**, the major product was the corresponding amine formed by reduction of the azide group. These reaction outcomes were consistent with those observed in the **4b**-based system.

Taken together, these results demonstrate that the presence of the azidomethyl substituent at the 5–position of 3–azido–5–(azidomethyl)benzoic acid–HP (**4a**) exerts no substantial effect on the organocatalytic [3+2] cycloaddition reactivity. In other words, compound **4a** exhibits chemoselective single–azide reactivity comparable to that of the conventional 3–azidobenzoic acid–HP (**4b**).

### 2.2. Stepwise Synthesis of DEL Warhead Using the C–D–DAP (1): On-DNA Diazido Platform

In the organocatalytic [3+2] cycloaddition reactions of 3–azido–5–(azidomethyl)benzoic acid–HP (**4a**) with various active methylene compounds, it was revealed that the azide group at the 3–position on the aromatic ring reacted selectively, whereas the azidomethyl group at the 5–position remained unreacted. This result indicates that the C–D–DAP, which contains two azide groups with distinct reactivities—an aromatic azide and an aliphatic azide—within a single molecule, can serve as a novel platform molecule suitable for stepwise reaction control.

**Figure 4:**
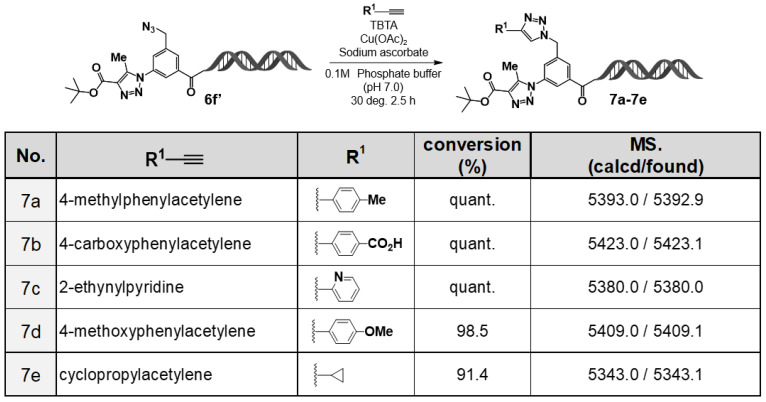
Sequential transformations of 3–azido–5–(azidomethyl)benzoic acid–HP via double click reactions. Substrate scope of the on–DNA CuAAC transformation using acetylene derivatives (**7a**–**7e**) and corresponding LC/MS confirmation. CuAAC of the on–DNA azide with acetylenes **7a**–**7e** was performed under standard conditions. LC/MS confirmed high conversions and the expected masses (calcd/found).

Based on this finding, we evaluated the utility of the C–D–DAP as an on–DNA platform molecule (Figure 4). First, using *tert*–butyl acetoacetate as the active methylene compound, an organocatalytic [3+2] cycloaddition reaction was conducted, affording the desired 1,4,5–trisubstituted 1,2,3–triazole in high yield. Subsequently, the remaining azide group was subjected to a copper(I)–catalyzed azide–alkyne cycloaddition (CuAAC) reaction [13,14,15] with various alkynes, including aromatic alkynes (**7a**, **7b**, **7d**), a heteroaromatic alkyne (**7c**), and an aliphatic alkyne (**7e**). In all cases, the corresponding 1,4–disubstituted 1,2,3–triazole products were obtained in high yields.

These results demonstrate that the C–D–DAP functions as a novel on–DNA platform molecule applicable to “double–click” transformations, in which two azide groups of different reactivities undergo stepwise conversion. Thus, sequential application of the organocatalytic [3+2] cycloaddition and CuAAC reactions enables the construction of on-DNA compound libraries bearing two distinct types of 1,2,3–triazole rings.

### 2.3. DEL-Compatible Synthesis Using the C–D–DAP (2): On–DNA Azido–Amino Platform

**Figure 5:**
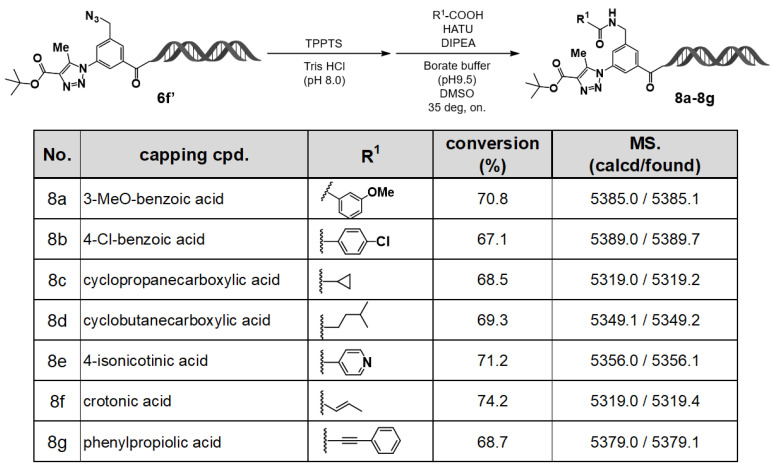
On–DNA HATU–mediated amidation with carboxylic acids: capping diversity and LC/MS–confirmed conversions (**8a**–**8g**). On–DNA amidation (capping) with carboxylic acids **8a**–**8g** was performed under HATU/DIPEA conditions. LC/MS confirmed conversions and expected product masses.

The azide group is a latent reactive site that can be readily reduced to an amino group. Therefore, in this study, we explored the feasibility of stepwise DEL construction using the C–D–DAP by first performing an organocatalytic [3+2] cycloaddition reaction, followed by selective reduction of the remaining azide group with triphenylphosphine-3,3′,3″–trisulfonic acid trisodium salt (TPPTS) to generate an amino group. This amino functionality was subsequently utilized in coupling reactions with various electrophilic reagents to examine the potential for stepwise synthesis (Figure 5).

In the final step, amidation was carried out using *O*–(7–azabenzotriazol–1–yl)–*N*,*N*,*N*′,*N*′–tetramethyluronium hexafluorophosphate (HATU) as the coupling reagent and *N*,*N*–diisopropylethylamine (DIPEA) as the base. Regardless of the type of carboxylic acid employed—aromatic (**8a**, **8b**), heteroaromatic (**8e**), aliphatic (**8c**, **8d**), or electrophilic carboxylic acids (**8f**, **8g**)—the desired amide products were obtained in high yields.

These results demonstrate that the C–D–DAP enables stepwise reactions at two azide groups with distinct reactivities. Specifically, following the formation of a 1,4,5–trisubstituted 1,2,3–triazole via organocatalytic [3+2] cycloaddition, subsequent reduction of the remaining azide to an amino group and its amidation can be sequentially performed. This strategy allows the construction of on-DNA compound libraries bearing both a 1,4,5–tri–substituted 1,2,3–triazole and a substituted aminomethyl group on the benzene ring.

### 2.4. Selective Reduction of Aromatic Azides: A New Approach for DEL Construction Initiated from the C–D–DAP

In DEL synthesis, DNA barcodes and primers are typically ligated using enzymatic reactions mediated by T4 DNA ligase [16]. However, the T4 DNA ligase buffer, which contains dithiothreitol (DTT) as a stabilizing agent, was suspected to induce unwanted reduction of aromatic azide groups [17]. To examine this, 4–azidobenzoic acid–HP (**9**) was treated in 10% T4 DNA ligase buffer at room temperature overnight. As anticipated, the aromatic azide group was reduced entirely to the corresponding amine.

This finding highlights an important consideration for DEL workflows. In conventional DEL synthesis, barcode ligation generally precedes the chemical reactions used to construct the reactive region via building blocks (BBs). However, when warhead-bearing aromatic azides are employed, the sequence of operations must be reversed—namely, the warhead-forming reactions should be performed before barcode ligation—to prevent undesired reduction during the enzymatic step.

**Figure 6:**
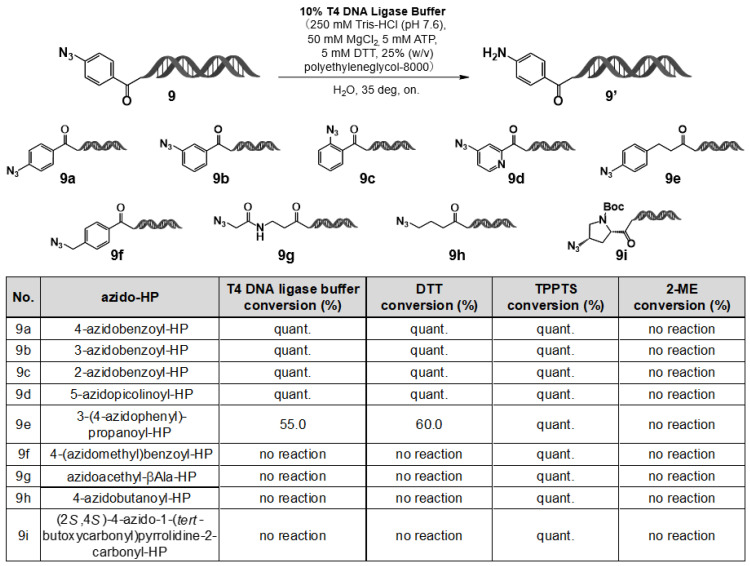
Comparison of T4 DNA ligase buffer, DTT, TPPTS, and 2–ME for the reduction of azido-HP substrates (**9a**–**9i**). Azido–HPs **9a**–**9i** were reduced under T4 ligase buffer, DTT, TPPTS, or 2–ME. LC/MS conversion values are shown; quant. = >95%.

To investigate further whether this reduction behavior extends to aliphatic azides, a series of azide–HP derivatives was evaluated under various reducing conditions (results summarized in Figure 6). Aromatic azides were completely reduced to amines upon treatment with T4 DNA ligase buffer or DTT for 24 h at room temperature, whereas aliphatic azides remained unchanged under identical conditions. By contrast, both aromatic and aliphatic azides were fully reduced in the presence of the stronger reducing agent TPPTS, whereas no reduction was observed with 2–mercaptoethanol (ME). These results demonstrate that aromatic azides can be selectively reduced in the presence of T4 DNA ligase buffer and DTT.

**Figure 7:**
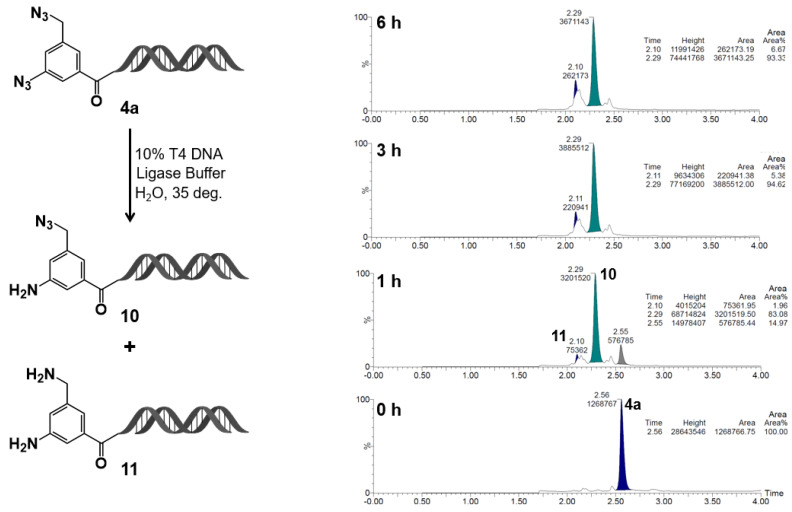
Reduction kinetics of on–DNA diazide **4a** in T4 DNA ligase buffer at 35 deg: formation of monoamine **10** and diamine **11**. On–DNA diazide **4a** was incubated in 10% T4 DNA ligase buffer (Tris–HCl pH 7.6, MgCl_2_, ATP, DTT, PEG–8000) in UltraPure distilled water at 35 deg. Reaction progress was monitored by LC/MS at 0, 1, 3, and 6 h. The parent diazide **4a** decreased over time, with formation of monoamine **10** and diamine **11**. Chromatograms show relative peak areas at each time point.

Based on this insight, the selective reduction of the aromatic azide group in compound **4a** was examined in detail (Figure 7). Upon treatment with T4 DNA ligase buffer, the aromatic azide was predominantly reduced to its amine form (compound **10**) within 1 h at room temperature, with complete consumption of the starting material after 3 h. In contrast to 4–azidomethylbenzoic acid–HP (**9f**), when **4a** was used as the substrate, a very small extent of reduction of the alkyl azide group was also observed. However, the formation of the reduction product **11** derived from the azidomethyl group at the 5–position remained below a few percent after 3 h and reached only about 10% after 6 h.

Taken together, these findings indicate that selective reduction of the aromatic azide group in **4a** can be achieved under mild, enzyme-compatible conditions. The resulting amino group can then be capped, followed by subsequent transformations of the remaining azidomethyl group. This stepwise reactivity control provides a new and practical strategy for constructing DELs based on the C–D–DAP scaffold.

### 2.5. DEL–Compatible Synthesis Using the C–D–DAP (3): On–DNA Amino–Azido Platform

**Figure 8:**
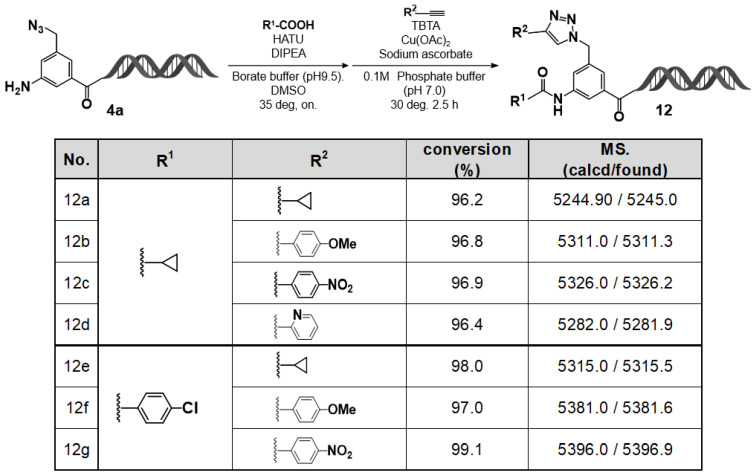
Two-step diversification of on–DNA amino intermediate via HATU–mediated amidation and CuAAC: substrate scope and LC/MS analysis (**12a**–**12g**). On–DNA amidation followed by CuAAC produced **12a**–**12g** with high conversions. LC/MS verified the expected masses (calcd/found).

Building on the findings from the previous section, we explored a stepwise reaction strategy for the C–D–DAP that enables differential functionalization of two azide groups with distinct reactivities. Specifically, selective reduction of the aromatic azide was first achieved using T4 DNA ligase buffer, followed by a Cu(I)–catalyzed azide–alkyne cycloaddition (CuAAC) reaction applied to the remaining azidomethyl group. The results are summarized in Figure 8.

Initially, the C–D–DAP was treated in 10% T4 DNA ligase buffer at room temperature for 5 h. After ethanol precipitation and lyophilization, the desired amino derivative—formed via selective reduction of the aromatic azide—was successfully obtained. Subsequent amidation reactions of this amino compound were conducted with cyclopropane carboxylic acid and 4–chlorobenzoic acid in borate buffer (pH 9.5), employing HATU as the coupling reagent and DIPEA as the base. In both cases, the corresponding amide products were obtained in good to excellent yields.

Next, the remaining azidomethyl group at the 5–position was subjected to CuAAC reactions with various terminal alkynes. Regardless of the alkyne type—aromatic, heteroaromatic, or aliphatic—the desired 1,4–disubstituted 1,2,3–triazole products were formed in high yields.

Collectively, these results demonstrate that the C–D–DAP scaffold serves as a versatile on–DNA platform for sequential reactions. By combining selective reduction of the aromatic azide, amine capping through amide bond formation, and subsequent CuAAC transformation of the residual azidomethyl group, we have established a practical and generalizable strategy for constructing structurally diverse on-DNA compound libraries.

### 2.6. DEL–Compatible Synthesis Using the C–D–DAP (4): On–DNA Diamino Platform

**Figure 9:**
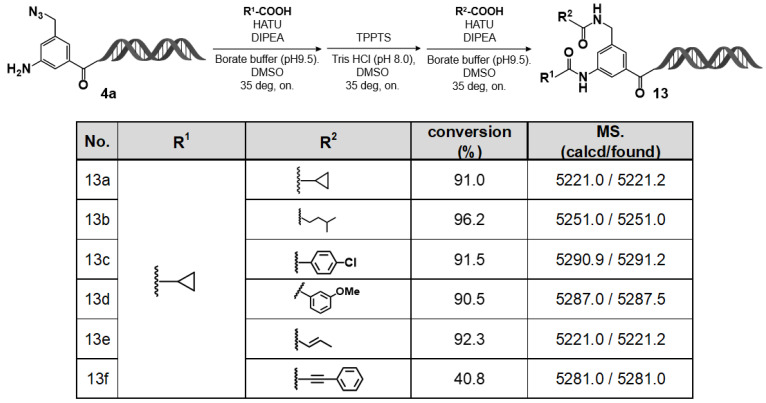
Sequential on–DNA amidation (R^1^–COOH) and TPPTS-assisted reduction followed by second amidation (R^2^–COOH): formation of bis-amide products **13a**–**13f**. Sequential amidation–TPPTS reduction–amidation-afforded products **13a**–**13f**. LC/MS showed high conversions and correct masses.

Using the C–D–DAP, we investigated the construction of an on–DNA diamino platform via a two–step amidation sequence (“double amidation”), involving sequential selective reductions of two azide groups with different reactivities. Specifically, the aromatic azide was first selectively reduced using T4 DNA ligase buffer, followed by amine capping. The remaining azidomethyl group was subsequently reduced with TPPTS and capped again via amidation. The results are summarized in Figure 9.

In practice, the C–D–DAP was treated with 10% T4 DNA ligase buffer at room temperature for 5 h. After ethanol precipitation and lyophilization, the amino derivative formed by selective reduction of the aromatic azide was obtained. This intermediate was amidated with cyclopropane carboxylic acid in borate buffer (pH 9.5) using HATU as the coupling reagent and DIPEA as the base, affording the desired amide product in good to excellent yield. The resulting product was collected by ethanol precipitation and lyophilization and used directly in the next step.

Subsequently, the intermediate was dissolved in Tris–HCl buffer (pH 8.0) and treated with TPPTS to reduce the azidomethyl group, yielding the corresponding aminomethyl derivative. This derivative was then reacted with various carboxylic acids under the same amidation conditions (HATU/DIPEA in borate buffer, pH 9.5) to afford the target diamide products in good to excellent yields.

Overall, these results demonstrate that the C–D–DAP can serve as a highly versatile starting scaffold for sequential selective reduction and amine capping of aromatic and aliphatic azides. This approach enables the stepwise synthesis of on–DNA diamide compound libraries, expanding the accessible chemical diversity of DELs through precise multistep transformations on–DNA.

### 2.7. Chemical Space Analysis of Virtual DELs Derived from the C–D–DAP

**Figure 10:**
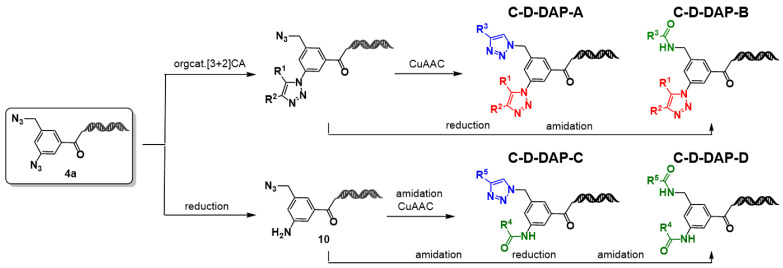
Synthetic routes to the C-D-DAP platform libraries (A–D). On–DNA diazide platform molecule was diversified via organocatalytic [3+2] cycloaddition, reduction, CuAAC, and amidation to generate four DEL–compatible sub–libraries. [3+2]CA reaction component (red), CuAAC reaction component (blue), amidation reaction component (green).

Starting from the precursor compound **4a**, the authors demonstrated that application of either aromatic azide-selective organocatalytic [3+2] cycloaddition or aromatic azide-selective reduction enables the construction of four distinct DELs: C–D–DAP–A, C–D–DAP–B, C–D–DAP–C, and C–D–DAP–D (Figure 10). To characterize and compare the properties of these libraries, a chemical space analysis was performed for the corresponding virtual DELs and compared with compounds from the ChEMBL database [18].

Chemical space analysis was conducted using the KNIME Analytics Platform (version 5.4.3) within a custom Conda environment (Python 3.11.6, RDKit version 2024.09.2 [19], Marvin Extensions 4.7.0 [20]). BBs were sourced from the Enamine commercial catalog. After desalting, compounds that satisfied all rule of 2 (Ro2) criteria [21]—molecular weight < 200, clogP < 2, hydrogen bond donors (HBDs) ≤ 2, and hydrogen bond acceptors (HBAs) ≤ 4—were retained for further use.

The selected BBs were converted into Morgan fingerprints (1024 bits, radius = 2) [22], and K–means clustering [23] was applied to group structurally similar compounds. Representative BBs were selected as cluster centroids. Ultimately, 200 derivatives of each of the active methylene compounds, terminal alkynes, and carboxylic acids were selected.

Based on the type and order of BB combinations, C–D–DAP–derived DELs were categorized as follows:

- C–D–DAP–A: BB_1_ = active methylene derivative, BB_2_ = terminal alkyne derivative
- C–D–DAP–B: BB_1_ = active methylene derivative, BB_2_ = carboxylic acid derivative
- C–D–DAP–C: BB_1_ = carboxylic acid derivative, BB_2_ = terminal alkyne derivative
- C–D–DAP–D: BB_1_ and BB_2_ = carboxylic acid derivatives

**Figure 11:**
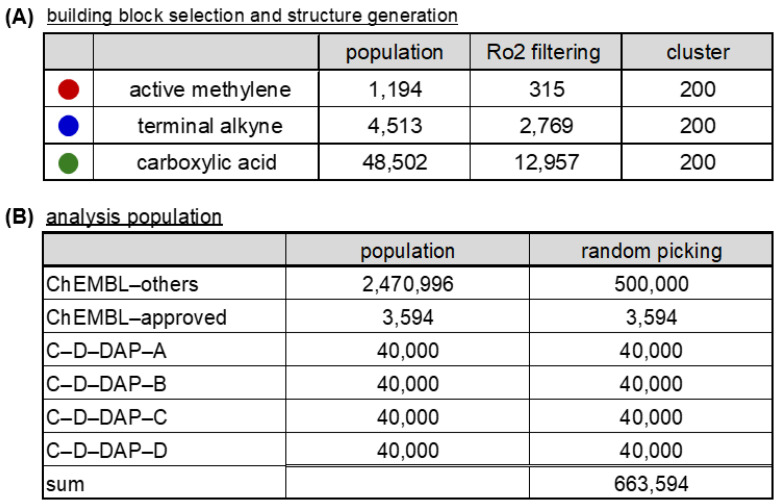
Summary of building block (BB) selection and population sampling for chemical space analysis (**A**) and Workflow for BB filtering and dataset construction for comparative chemical space analysis (**B**). (**A**) Three classes of commercially available BBs—active methylene, terminal alkyne, and carboxylic acid—were filtered by Ro2 and clustered (200 clusters each) to generate representative sets for virtual DEL construction. (**B**) For chemical space analysis, representative populations were prepared from ChEMBL (approved, investigated, and remaining compounds) and the four C–D–DAP libraries. Random sampling was applied where indicated.

For each DEL type, 40,000 compounds (200 × 200 combinations) were generated in silico, and the resulting virtual libraries were subjected to chemical space mapping and comparative analysis with ChEMBL–derived molecular space (Figure 11).

### 2.8. Comparative Analysis of Physicochemical Properties and Drug–likeness of Virtual DELs

**Figure 12:**
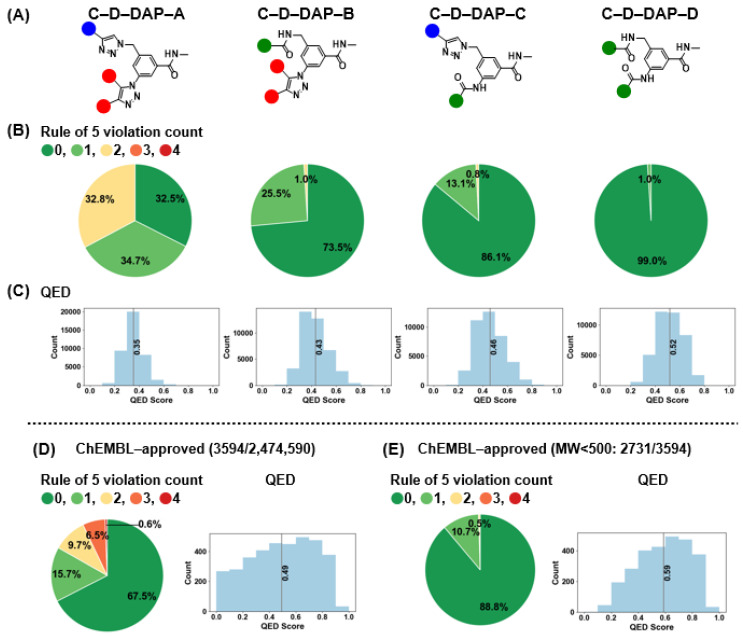
Drug–likeness assessment of the C–D–DAP libraries: rule–of–5 violation profiles and QED score distributions compared with ChEMBL–approved drugs. (**A**) Structures of the four virtual C–D–DAP libraries (**A**–**D**). (**B**) Distribution of Lipinski’s rule of 5 (Ro5) violation counts across each library, showing the proportion of compounds with 0–4 violations. (**C**) Quantitative estimate of drug-likeness (QED) score distributions for each library. (**D**) Ro5 violation and QED distribution for all ChEMBL–approved compounds (*n* = 3594). (**E**) Same analyses applied to ChEMBL approved compounds with MW < 500 (*n* = 2731) for comparison with DEL-like space.

The C–D–DAP scaffold was designed as a platform molecule for the identification of binders targeting small–molecule drug discovery targets, with the warhead expected to exhibit favorable oral absorption properties. To evaluate the physicochemical and drug-likeness characteristics of the virtual libraries derived from this scaffold (C–D–DAP–A, –B, –C, and –D), we calculated two representative descriptors: the number of Lipinski’s rule of five violation counts (Ro5VC) [24] and the quantitative estimate of drug-likeness (QED) [25] (Figure 12A–C).

Ro5VC indicates the number of physicochemical parameters that exceed Lipinski’s criteria [26]; two or more violations generally imply reduced oral absorption or membrane permeability. By contrast, QED, proposed by Bickerton et al. (Pfizer, New York, NY, USA), provides a continuous 0–1 score that quantitatively reflects drug-likeness based on eight molecular descriptors: molecular weight, lipophilicity, hydrogen bond acceptor (HBA)/hydrogen bond donor (HBD) counts, polar surface area, rotatable bond count, aromatic ring count, and structural alerts (e.g., PAINS [27]). A QED value approaching 1 denotes higher similarity to marketed drugs.

**Figure 13:**
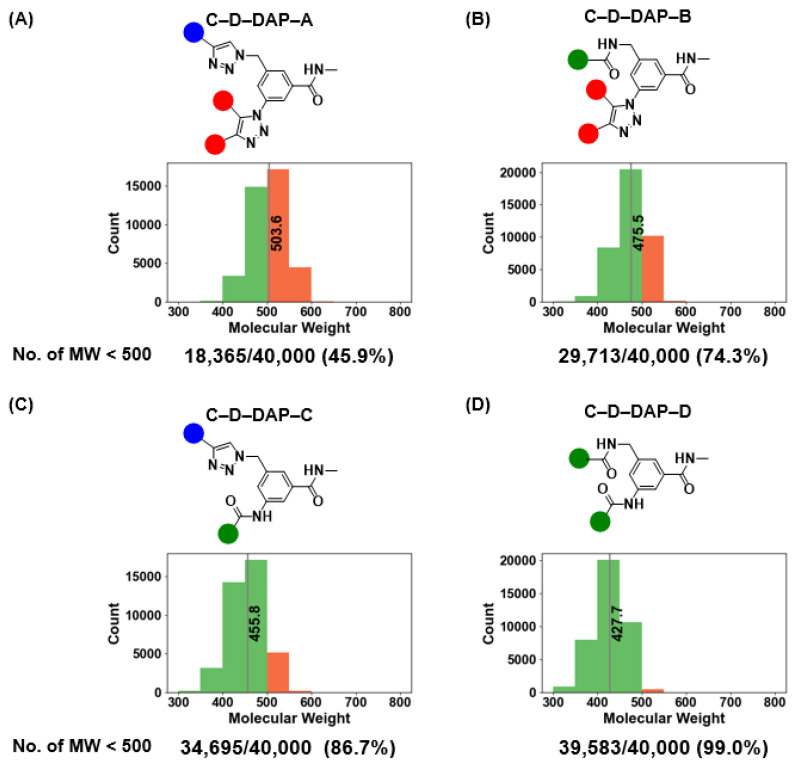
Molecular weight distributions of the C–D–DAP libraries (**A**–**D**) and proportions of compounds with MW < 500. (**A**–**D**) Histograms show molecular weight (MW) distributions for each C–D–DAP library (**A**–**D**). The red bar indicates the mean MW for each library. The number and percentage of compounds with MW < 500 are shown below each plot (*n* = 40,000 per library). C–D–DAP–C and C–D–DAP–D exhibit particularly high fractions of MW < 500, aligning well with typical small-molecule drug-like space.

The analysis results are summarized in Figure 13. For Ro5VC, color coding was applied (0 = green, 1 = light green, 2 = yellow, 3 = orange, 4 = red). The fractions of compounds with Ro5VC = 0 were 32.5%, 73.5%, 86.1%, and 99.0% for C–D–DAP–A, –B, –C, and –D, respectively, whereas those with Ro5VC = 1 were 34.7%, 25.5%, 13.1%, and 1.0%. Compounds with Ro5VC = 2 were observed only in C–D–DAP–A (32.8%) and –B (1.0%), and were absent in –C and –D. When compounds with Ro5VC ≤ 1 were considered collectively, their proportions were 67.2%, 99.0%, 99.2%, and 100.0% for C–D–DAP–A through –D, suggesting a progressive improvement in predicted oral absorption and permeability in the order of D > C > B > A.

The mean QED values followed the same trend, increasing from 0.35 (A) to 0.43 (B), 0.46 (C), and 0.52 (D). The lower QED of C–D–DAP–A primarily stems from the higher proportion of compounds with Ro5VC = 2. In particular, the 1,2,3–triazole ring in the base structure of C–D–DAP–A contributes two HBAs, and the amide linkage to the HP tag contributes one additional HBA, resulting in a cumulative HBA count of five in the warhead—likely contributing to the reduced QED score. A strong correlation (r = 0.96) was observed between the proportion of compounds with Ro5VC = 0 and the QED values, indicating that both parameters consistently reflect the drug-likeness trend across the four libraries (see Supplementary Material).

Collectively, these results reveal that each virtual C–D–DAP–derived DEL exhibited distinct physicochemical and drug-likeness profiles at the warhead level. These findings highlight that variations in the core reactive scaffold architecture substantially influence the overall physicochemical landscape of the corresponding libraries, emphasizing the importance of scaffold design in modulating DEL chemical space.

### 2.9. Comparison Between C–D–DAP–Derived Virtual DELs and ChEMBL–Approved Drugs

As shown in Figure 12D, we analyzed the distribution of Ro5VC and QED values for 3594 approved drugs in ChEMBL version 35 with confirmed structural information (“ChEMBL-approved”). The Ro5VC distribution was as follows: 0 (67.5%), 1 (15.7%), 2 (9.7%), 3 (6.5%), and 4 (0.6%), with compounds having Ro5VC = 0 or 1 accounting for 83.1% of the total. This proportion was lower than that observed for the C–D–DAP–B, –C, and –D libraries, likely reflecting the inclusion of non-orally administered drugs, such as injectables, within the ChEMBL dataset.

The median QED value for the ChEMBL-approved compounds was 0.49, comparable to those of C–D–DAP–C (0.46) and C–D–DAP–D (0.52).

Inspection of the QED histogram revealed that 547 drugs (15.2% of the total) exhibited QED values between 0.0 and 0.2. These low–QED compounds were primarily peptides and peptide derivatives, including high-molecular-weight agents such as cefiderocol sulfate tosylate (MW 3061.6, QED 0.0040), insulin detemir (MW 5916.9, QED 0.0073), and pramlintide (MW 3949.5, QED 0.0084). The presence of such large peptide–based therapeutics likely contributes to lowering the overall average QED of the ChEMBL–approved dataset (other representative low–QED compounds are listed in the Supplementary Material).

To provide a more relevant comparison with small-molecule DEL warheads, we next focused on a subset of approved drugs with molecular weights below 500 Da (2731 compounds) (Figure 12E). In this subset, the proportions of compounds with Ro5VC = 0, 1, and 2 were 88.8%, 10.7%, and 0.5%, respectively, giving a combined total of 99.5% for Ro5VC ≤ 1. The median QED value for this subset was 0.59, slightly higher than that of the C–D–DAP–C and –D libraries. These results indicate that the warheads of C–D–DAP–C and –D possess physicochemical properties—particularly with respect to oral absorption and membrane permeability—comparable to those of orally available small–molecule drugs.

We further examined the average molecular weights and distributions of the warheads in each C–D–DAP–derived virtual library (Figure 13). The average molecular weights for C–D–DAP–A, –B, –C, and –D were 503.6, 475.5, 455.8, and 427.7, respectively, with C–D–DAP–A being the heaviest. This distribution reflects the use of relatively large active methylene BBs common to C–D–DAP–A and –B.

The proportion of compounds with molecular weight below 500 Da was 45.9% (18,365/40,000) for C–D–DAP–A, 74.3% (29,713/40,000) for –B, 86.7% (34,395/40,000) for –C, and 99.0% (39,583/40,000) for –D. Notably, nearly all warheads in C–D–DAP–D and approximately half in C–D–DAP–A fell below the 500 Da threshold, demonstrating that all four libraries consist predominantly of small–molecule–like compounds suitable for binder discovery against small–molecule drug targets.

### 2.10. Chemical Space Analysis of the C–D–DAP Libraries

The C-D-DAP libraries were designed from 3–azido–5–(azidomethyl)benzoic acid–HP (**4a**) as a core scaffold to identify binders for small-molecule drug targets. Their warheads showed favorable profiles for oral absorption and moderate drug–likeness, with most compounds having molecular weights below 500 Da. These properties suggested potential overlap with the chemical space occupied by approved drugs in the ChEMBL bioactive compound database.

To evaluate this, a model of chemical space was constructed from 500,000 randomly selected compounds from ChEMBL version 35. Extended circular fingerprints (FCFP4; radius = 2, 1024 bits) were generated and projected onto two dimensions using uniform manifold approximation and projection [28] (UMAP; n_neighbors = 50, metric = “jaccard”). The trained model was applied to four C–D–DAP libraries (A–D; 40,000 compounds each) and to 3594 approved drugs in ChEMBL, enabling direct visualization of their distributions within a unified space.

**Figure 14:**
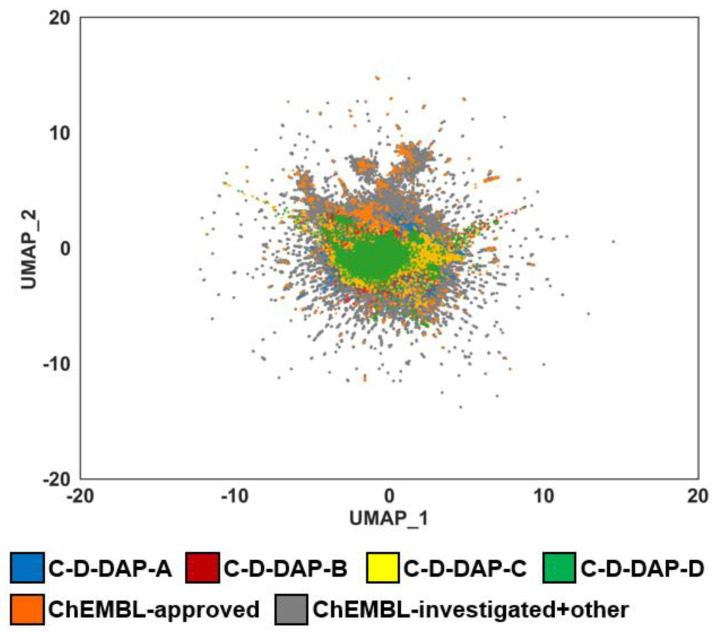
Comparison of chemical space distribution of C–D–DAP libraries and ChEMBL datasets using UMAP dimensionality reduction. UMAP (Uniform Manifold Approximation and Projection) was applied to depict the chemical space of four virtual C–D–DAP libraries (A–D; blue, red, yellow, green) in comparison with ChEMBL approved compounds (orange) and ChEMBL investigated + other compounds (gray). All datasets were embedded using identical molecular descriptors and UMAP parameters. Substantial overlap between C–D–DAP libraries and drug-like ChEMBL compounds indicates that the designed DEL scaffolds occupy pharmaceutically relevant chemical space.

The analysis showed that the C–D–DAP libraries formed distinct local clusters within the ChEMBL bioactive compound space, partially overlapping with high–density regions of approved drugs. This overlap indicates chemical proximity to known drug–like molecules while extending into less–explored regions. Thus, the **4a**-based DEL design effectively bridges established drug space and novel chemical territory, providing a promising framework for DEL screening in small–molecule drug discovery (Figure 14).

### 2.11. Comparative Analysis of the Chemical Space of C–D–DAP Libraries

UMAP analysis revealed that all four virtual DELs (C–D–DAP–A to –D) partially overlapped with the chemical space of ChEMBL-approved drugs, suggesting their suitability for small–molecule drug discovery. Because the point density was too high for quantitative comparison, kernel density estimation (KDE) was used to evaluate the spatial distribution, overlap with the approved-drug region, and correlation with mean QED values.

**Figure 15:**
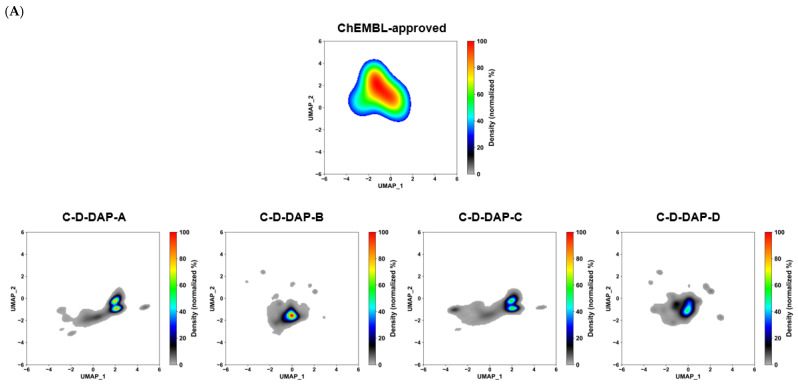

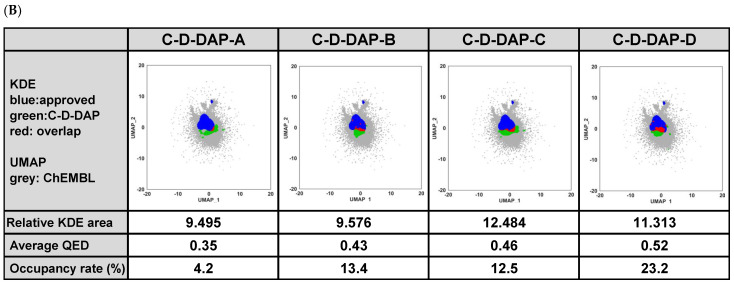
Kernel density and UMAP–based analysis of chemical space overlap between C–D–DAP libraries (A–D) and ChEMBL approved compounds. (**A**) KDE (kernel density estimation) maps of UMAP–embedded chemical space for ChEMBL approved compounds (top) and each C–D–DAP library (A–D, bottom). ChEMBL density is shown in color, with the C–D–DAP distributions overlaid in grayscale to visualize spatial overlap and regions of high density. (**B**) UMAP scatterplots showing ChEMBL compounds (gray), C–D–DAP compounds (green), approved drugs (blue) and overlap with C–D–DAP compounds and approved drugs (red). Calculated metrics include KDE area (a measure of occupied chemical space), average QED score, and occupancy rate (%), defined as the proportion of the ChEMBL–approved KDE region covered by each C–D–DAP library.

KDE was applied to the two-dimensional UMAP coordinates (UMAP_1, UMAP_2) using scipy.stats.gaussian_kde with a bandwidth estimated by Scott’s rule. Density values above 0.01 were integrated to estimate occupied area and normalized to generate heatmaps. As shown in Figure 15A, all libraries were located in similar regions, reflecting their shared warhead scaffold (**4a**). Minor differences in spread and density were observed, with occupied area decreasing in the order C–D–DAP–C > D > B > A.

The degree of overlap with the ChEMBL–approved region (Figure 15B) followed the order D > C > B > A. C–D–DAP–D showed the highest overlap (~25%), indicating strong affinity for drug-relevant chemical space, likely due to its amide-linked structure, a common pharmacophore motif. By contrast, triazole-linked A and B occupied more isolated subspaces, contributing additional diversity.

A strong linear correlation (r^2^ = 0.93) was found between the overlap rate and average QED value, quantitatively confirming that libraries with higher drug-likeness are more closely aligned with the approved–drug space (see Supplementary Material). These findings validate the pharmacological relevance of the warhead series in the order D > C > B > A.

Together, UMAP–KDE integrated analysis provided a quantitative and intuitive approach for assessing library design. Beyond simple visualization, this method enables comparison of scaffold-specific occupation areas and relative positioning within known drug space, offering a practical metric for scaffold selection and diversity optimization in DEL design.

Overall, all four C–D–DAP libraries clustered near the main ChEMBL–approved region while maintaining scaffold–specific subspaces. This clustering demonstrates that the C–D–DAP framework effectively covers pharmaceutically relevant chemical space while retaining structural diversity, supporting its potential as a versatile on–DNA platform for future DEL synthesis and screening.

## 3. Materials and Methods

### 3.1. General Information

Unless otherwise noted, materials, the DNA headpiece (HP–NH_2_) (5′–/5phos/GAGTCA/iSp9/iUniAmM/iSp9/TGACTCCC–3′) and solvents were obtained from commercial suppliers and used without further purification. All on–DNA reactions were performed in a 0.2 mL PCR tube or 1.5 mL/2.0 mL microtubes. UPLC–MS was used to analyze on-DNA reactions in studies to optimize reaction conditions and extend substrate scope. Typically, 1.0 μL samples were dissolved in an appropriate amount of UltraPure^TM^ (Thermo Fisher Scientific Inc., Waltham, MA, USA) distilled water and injected into a reverse-phase chromatography column (Waters XBridge Oligonucleotide BEH C18 column, 1.7 μm, 2.1 × 50 mm) (Waters Inc., Milford, MA, USA) at 60 deg. The column was eluted as follows: 10–90% solvent B over 4.5 min, 0.4 mL/min, λ = 260 nm; solvent A: water/1,1,1,3,3,3–hexafluoro–2-propanol/triethylamine = 100/2/0.1 (*v*/*v*); solvent B: methanol/1,1,1,3,3,3–hexafluoro–2-propanol/triethylamine/water = 100/2/0.1/2 (*v*/*v*). The effluents were analyzed using a XevoG2–XS Q–TOF (Waters Inc., Milford, MA, USA) equipped with an electrospray ionization source.

On–DNA reaction yield calculation: ignoring UV coefficient differences across all on-DNA products and assuming 100% total DNA recovery, the yield of DNA products was determined from the total ion chromatography peak area.

### 3.2. General Procedures for the Synthesis of DNA Compounds

#### General Procedure for the Synthesis of DNA–Conjugated Azides

To a solution of DNA HP (1 mM in UltraPure distilled water) in borate buffer (50 mM, pH 9.5, 20 μL) and UltraPure distilled water (80 μL), a mixture consisting of DMTMM·BF_4_ (200 mM in DMSO, 30 μL), azide compound (200 mM in DMSO, 30 μL), and DMSO (40 μL) was added. The resulting mixture was vortexed vigorously, briefly centrifuged, and incubated at 35 deg. overnight with shaking. Subsequently, 5 M NaCl (40 μL) and cold ethanol (1.60 mL) were sequentially added. The mixture was vortexed again, centrifuged briefly, and stored at −80 deg. for 30 min. After storage, the sample was centrifuged at 16,000× *g* for 30 min at 4 deg. to remove the supernatant. The resulting pellet was redissolved in UltraPure distilled water and lyophilized. The lyophilizate was redissolved in UltraPure distilled water, and the same amount of 10% aqueous piperidine was added. The mixture was vortexed vigorously, briefly centrifuged, and incubated at 35 deg. for 2 h with shaking. Subsequently, 5 M NaCl (40–60 μL) and cold ethanol (1.60 mL) were sequentially added. The mixture was vortexed again, centrifuged briefly, and stored at −80 deg. for 30 min. After storage, the sample was centrifuged at 16,000× *g* for 30 min at 4 deg. to remove the supernatant. The resulting pellet was redissolved in UltraPure distilled water and lyophilized. The obtained material was used directly in subsequent reactions without further purification.

### 3.3. General Procedure for the on–DNA Enolate–Azide [3+2] Cycloaddition Reaction

To a solution of azido–modified DNA headpiece (500 μM UltraPure distilled water solution, 8 μL), DMSO (26.4 μL), an active methylene compound (4.8 μL, 100 mM in DMSO), and DBU (0.8 μL, 100 mM in DMSO) were added sequentially. The resulting mixture was vortexed vigorously, briefly centrifuged, and incubated at 35 deg. overnight with continuous shaking. After the reaction, a 1 μL aliquot of the reaction mixture was diluted with UltraPure distilled water (150 μL) and analyzed by LC–MS.

The conversion of DNA–conjugated products was estimated from integrated peak areas in the total ion chromatogram, without correction for differences in UV absorbance coefficients among the products, assuming complete DNA recovery.

### 3.4. General Procedure for the on–DNA CuAAC Reaction

Lyophilized azide–conjugated DNA in UltraPure distilled water (500 μM, 10 μL) was added to an alkyne derivative (200 mM in DMSO, 4 μL), phosphate buffer (0.1 M, pH 7.0, 2.4 μL), TBTA ligand (25 mM in DMSO, 1.6 μL), Cu(OAc)_2_ (50 mM in UltraPure distilled water, 1.6 μL), and sodium ascorbate (50 mM in UltraPure distilled water, 1.6 μL). The reaction mixture was incubated at 40 deg. for 150 min with shaking. Afterward, sodium diethyldithiocarbamate (Na–DTC, 2000 mM in UltraPure distilled water, 2 μL) was added to quench the reaction, followed by incubation for an additional 30 min at room temperature. The mixture was then centrifuged at 12,000 rpm for 5 min at 4 deg. An aliquot of the supernatant (5 μL) was diluted with UltraPure distilled water (150 μL) and subjected to LC-MS analysis.

### 3.5. General Procedure for TPPTS Reduction of on–DNA Azide

Lyophilized azide-conjugated DNA in UltraPure distilled water (500 μM, 20 μL) was added, and Tris–HCl buffer (0.1 M, pH 8.0, 42 μL) and TPPTS (200 mM in UltraPure distilled water, 8.0 μL) were added sequentially. The resulting mixture was incubated at 40 deg. overnight with shaking. Following the reaction, the mixture was centrifuged at 12,000 rpm for 5 min at 4 deg. An aliquot of the supernatant (5 μL) was diluted with UltraPure distilled water (150 μL) and analyzed by LC-MS.

### 3.6. General Procedure for T4 DNA Ligase Buffer Reduction of on–DNA Azide

Lyophilized azide–conjugated DNA in UltraPure distilled water (90 μL) was added, as well as 10× T4 DNA ligase buffer (10 μL). The resulting mixture was incubated at 40 deg. overnight with shaking. Following the reaction, the mixture was centrifuged at 12,000 rpm for 5 min at 4 deg. An aliquot of the supernatant (5 μL) was diluted with UltraPure distilled water (150 μL) and analyzed by LC–MS.

### 3.7. General Procedure for DTT Reduction of on–DNA Azide

Lyophilized azide-conjugated DNA in UltraPure distilled water (90 μL) was added to DTT buffer (20 mM, 10 μL). The resulting mixture was incubated at 40 deg. overnight with shaking. Following the reaction, the mixture was centrifuged at 12,000 rpm for 5 min at 4 deg. An aliquot of the supernatant (5 μL) was diluted with UltraPure distilled water (150 μL) and analyzed by LC–MS.

### 3.8. General Procedure for 2–ME Reduction of on–DNA Azide

Lyophilized azide–conjugated DNA in UltraPure distilled water (90 μL) was added to a 2–ME solution (20 mM, 10 μL). The resulting mixture was incubated at 40 deg. overnight with shaking. Following the reaction, the mixture was centrifuged at 12,000 rpm for 5 min at 4 deg. An aliquot of the supernatant (5 μL) was diluted with UltraPure distilled water (150 μL) and analyzed by LC–MS.

## 4. Conclusions and Outlook

Recent advances in therapeutic modalities such as antibody drugs, nucleic acid therapeutics, peptide medicines, and cell-based therapies have enabled precise control of complex biomolecular interactions, offering new treatments for diseases previously inaccessible to traditional small molecules. Nevertheless, small-molecule drug discovery remains central to pharmaceutical development because of its broad target coverage, cost-effectiveness, and suitability for oral administration [29,30,31,32]. However, concerns about the depletion of druggable targets highlight the need for efficient discovery of bioactive compounds by exploring broader, more diverse chemical spaces. Even in areas with numerous available drugs, such as hypertension, subsets of patients remain refractory [33,34,35,36], emphasizing the necessity for new strategies that transcend the limits of conventional HTS.

Against this backdrop, DEL technology has emerged as a transformative platform that enables the exploration of an enormous chemical diversity far exceeding that of HTS. DEL enables the synthesis, encoding, and screening of millions to billions of compounds simultaneously in a single reaction vessel, dramatically reducing experimental effort while increasing the likelihood of discovering novel scaffolds and binding modes. As such, DEL is expected to continue expanding the frontier of small-molecule drug discovery.

In DEL synthesis, platform molecules serve as central scaffolds that balance chemical diversity with synthetic reproducibility. These scaffolds possess a DNA–anchoring site and multiple reactive handles, allowing sequential introduction of diverse BBs and efficient multistep on–DNA synthesis. Representative examples include 2,4,6–trichloro–1,3,5–triazine [37,38,39,40] and protected amino acid derivatives such as (4S) –*N*^α^–9–Fluorenyl–methoxycarbonyl–*N*^ε^–*t*–butyloxycarbonyl–lysine (Fmoc–Lys(Boc)–OH) and (4S)–4–[[9H–fluoren–9–ylmethoxy(oxo)methyl]amino]–5–oxopentanoic acid tert–butyl ester (Fmoc–Glu(tBu)–OH) [41,42,43,44,45]. Triazine scaffolds are especially useful because each chlorine atom exhibits distinct nucleophilic substitution reactivity, enabling stepwise diversification at three positions. However, the reactivity differences among the chlorines complicate precise reaction control: the first substitution occurs readily, whereas the second and third often require higher temperatures, leading to byproducts and lower yields. These challenges can reduce reaction reproducibility and library fidelity during multistep DEL synthesis.

Similarly, amino–acid–derived platforms require deprotection of side–chain protecting groups, extending the synthetic sequence and demanding strict control of DNA stability. Moreover, when using DMTMM salts—commonly applied coupling agents in DEL amidation—undesired DNA–DMT adducts can form, requiring additional base treatments for removal. Such steps not only increase complexity but may also compromise reaction efficiency and DNA integrity. Therefore, despite their versatility, existing platform scaffolds remain limited by chemical constraints, including issues of selectivity, byproduct formation, and DNA stability. Designing new platform molecules that combine broad BB compatibility with robust reaction control is thus crucial for advancing DEL technology.

To address these challenges, we developed an on–DNA diazide platform (DAP), a new DEL construction strategy that exploits the reactivity difference between aromatic and aliphatic azides. This approach allows stepwise structural elaboration from a single functional group, thereby expanding chemical diversity through deliberate control of reactivity hierarchy. Unlike conventional multifunctional scaffolds, DAP enables programmed, multistep transformations on the same DNA–conjugated framework.

We first established an organocatalyzed [3+2] cycloaddition that proceeds selectively with aromatic azides, which served as a key step in the construction of the C–D–DAP–A and –B libraries. The reaction occurs smoothly at room temperature, with high yield and excellent DNA compatibility. Subsequently, we discovered that dithiothreitol (DTT), a component of T4 DNA ligase buffer, acts as a selective reducing agent for aromatic azides, leaving aliphatic azides intact. This finding enabled orthogonal transformations on the same scaffold and the creation of additional library types (C–D–DAP–C, –D).

The DTT–based selective reduction also proved applicable to substrates that perform poorly in the organocatalyzed [3+2] cycloaddition due to competing Regitz–diazo transfer. For instance, 2–azidobenzoic acid–HP was efficiently converted under mild conditions, demonstrating high chemoselectivity and broad applicability to aromatic azides that are otherwise difficult to manipulate. Furthermore, the reaction was compatible with our previously reported amino–acid–derived DAP scaffolds (compounds **1**–**3**), substantially expanding platform versatility. In one case, the azido group at the 4–position of the benzoyl moiety of **2** was selectively reduced under T4 DNA ligase buffer conditions, followed by amine capping and subsequent transformation of the aliphatic side chain (see Supplementary Material). This stepwise on–DNA synthesis successfully yielded a new DEL, confirming that DAP scaffolds allow chemical differentiation and sequential modification of multiple azide groups on–DNA.

Some limitations remain. Ortho–substituted aromatic azides are unsuitable as warheads, and in the [3+2] cycloaddition, active methylene BBs bearing bulky substituents near the carbonyl group tend to undergo Regitz–diazo transfer rather than triazole formation. These side reactions arise from steric and electronic effects, underscoring the importance of careful BB design.

In summary, the on–DNA diazide–based azide–selective platform strategy (DAP Strategy) represents a rational framework for DEL synthesis that overcomes the selectivity constraints of conventional multifunctional scaffolds. By leveraging the intrinsic reactivity hierarchy between aromatic and aliphatic azides, this approach enables orthogonal, multistep structural transformations from a single functional group—thereby providing a versatile and design-oriented method for expanding DEL chemical diversity.

Looking forward, further development of aromatic azide–selective reactions and optimized conditions will broaden the scope and utility of the DAP Strategy. Combined with large–scale DEL synthesis and screening studies, this strategy has the potential to serve as a general, practical platform for constructing chemically diverse and synthetically robust DNA–encoded libraries, ultimately contributing to the discovery of new small-molecule therapeutics.

## Data Availability

The original contributions presented in this study are included in the article/Supplementary Material. Further inquiries can be directed to the corresponding author.

## Supplementary Materials

The following supporting information can be downloaded at: https://www.mdpi.com/article/10.3390/ijms27020828/s1.
