# Supplementary material for: On–DNA Platform Molecules Based on a Diazide Scaffold II: A Compact Diazide Platform Designed for Small–Molecule Drug Discovery
Source: Int J Mol Sci. 2026 Jan 14;27(2):828. doi: 10.3390/ijms27020828 (PMC12841052; doi:10.3390/ijms27020828)

SUPPORTING INFORMATION

Development of Novel On-DNA Platform Molecules Based on a Diazide Scaffold II: A Compact Diazide Platform Designed for Small-Molecule Drug Discovery

Hiroyuki Miyachi*, Masaki Koshimizu, Masashi Suzuki

Lead Exploration Unit, Drug Discovery Initiative, University of Tokyo,7-3-1 Hongo, Bunkyo, Tokyo 113-0033, Japan

I. General Information

Unless otherwise noted, materials, DNA headpiece (HP-NH2) (5'- / 5phos / GAGTCA / iSp9 / iUniAmM / iSp9 / TGACTCCC-3', Figure S1) and solvents obtained from commercial suppliers were used without further purification. All on-DNA reactions were performed in 0.2 mL PCR tube or 1.5mL / 2.0 mL micro tubes.

On-DNA reactions in the studies of reaction condition optimization and substrate scope extension were analyzed by UPLC-MS. Typically, 1.0 uL samples were dissolved in an appropriate amount of UltraPure™ distilled water and injected into a reverse-phase chromatography column (Waters XBridge Oligonucleotide BEH C18 column, 1.7 μm, 2.1 × 50 mm) at 60° C. The elution was carried out as followings: 10–90% solvent B over 4.5 min, 0.4 mL/min, λ = 260 nm; solvent A: water / 1,1,1,3,3,3-hexafluoro-2-propanol / triethylamine = 100 / 2 / 0.1 (v/v); solvent B: methanol / 1,1,1,3,3,3-hexafluoro-2-propanol / triethylamine / water = 100 / 2 / 0.1 / 2 (v/v). The effluents were analyzed by a Xevo G2-XS Q-TOF with electrospray ionization source was used for detection.

On DNA reaction yield calculation: Ignoring UV coefficient difference for all on DNA products and assuming 100% of DNA total recovery, the yield of DNA products was determined from total ion chromatography peak area.

II. Abbreviations

DEL:DNA-encoded library
HTS:high-throughput screening
QTOF-MS:quadrupole time-of-flight mass spectrometry
PPI:protein–protein interaction
D-DAP:on-DNA diazido platform
eRo5:extended Rule of 5
GPCR:G protein–coupled receptor
HP:head piece
DMTMM BF_4: 4-(4,6-Dimethoxy-1,3,5-triazin-2-yl)-4-methylmorpholinium tetrafluoroborate
DBU:1,8-diazabicyclo[5.4.0]undec-7-ene
QTOF-MS:quadrupole time-of-flight mass spectrometry
CuAAC: Copper(I)-Catalyzed Alkyne–Azide Cycloaddition
DTT:dithiothreitol
2-ME: 2-mercaptoethanol
BB:building blocks
DIPEA: N,N-diisopropylethylamine
DMSO: dimethyl sulfoxide
HATU: 2-(7-Azabenzotriazol-1-yl)-N,N,N',N'-tetramethyluronium hexafluorophosphate
ATP: adenosine Triphosphate
PEG:polyethylene glycol
HBD:hydrogen bond donor
HBA:hydrogen bond acceptor
QED:Quantitative Estimate of Drug-likeness
Ro5VC:Lipinski's Rule of Five violation counts
QED:Quantitative Estimate of Drug-likeness
PAINS:pan-assay interference compounds
MW:molecular weight
UMAP:Uniform Manifold Approximation and Projection
KDE:kernel density estimation
LC-MS: Liquid Chromatography-Mass spectrometry
Na DTC: Sodium diethyldithiocarbamate
TBTA: Tris[(1-benzyl-1*H*-1,2,3-triazol-4-yl)methyl]amine
TCEP: Tris(2-carboxyethyl)phosphine
TPPTS: 3,3′,3″-Phosphanetriyltris(benzenesulfonic acid) trisodium salt
Tris HCl: tris(hydroxymethyl)aminomethane hydrochloride

Chemical structure of HP

(5’-/5phos/GAGTCA/iSp9/iUniAmM/iSp9/TGACTCCC-3’)

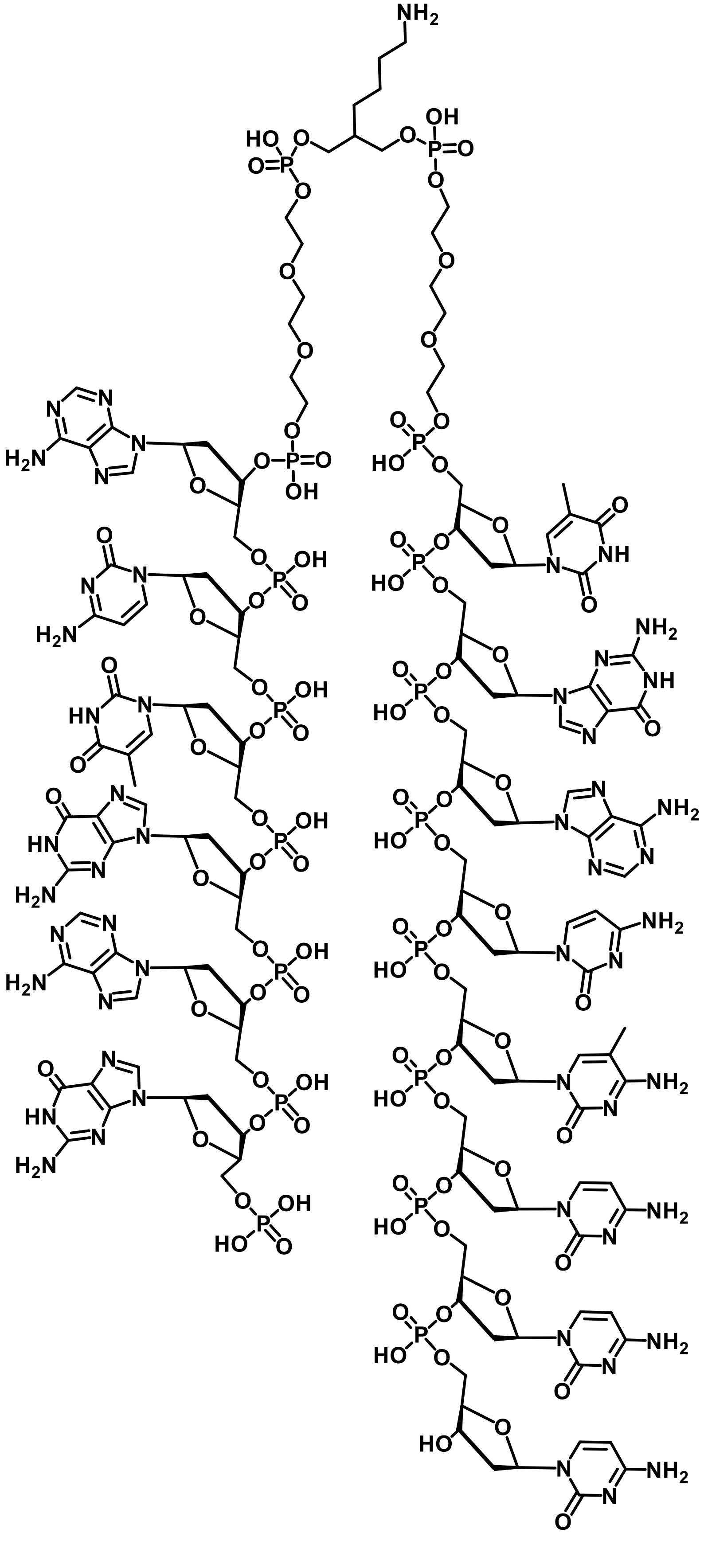

2.1. Organocatalyzed [3+2] Cycloaddition Reactions of 3-azido-5-(azidomethyl)benzoic acid-HP

Synthesis of on-DNA diazide conjugate via DMTMM-mediated amidation and its LC/MS characterization.

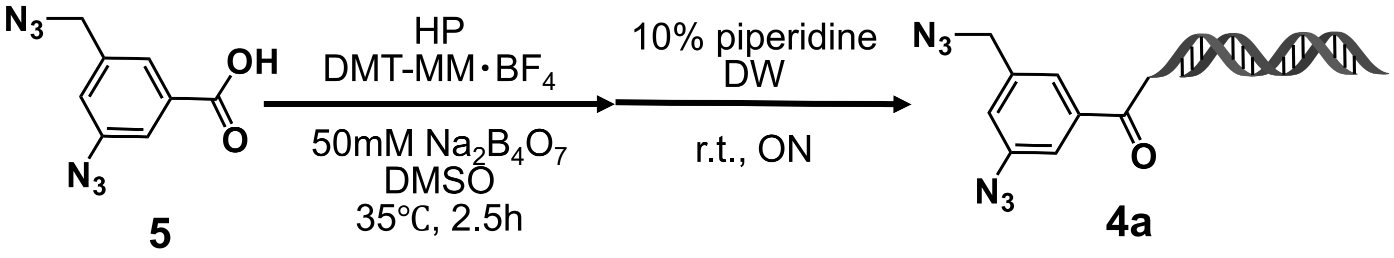

calcd.: 5137.0

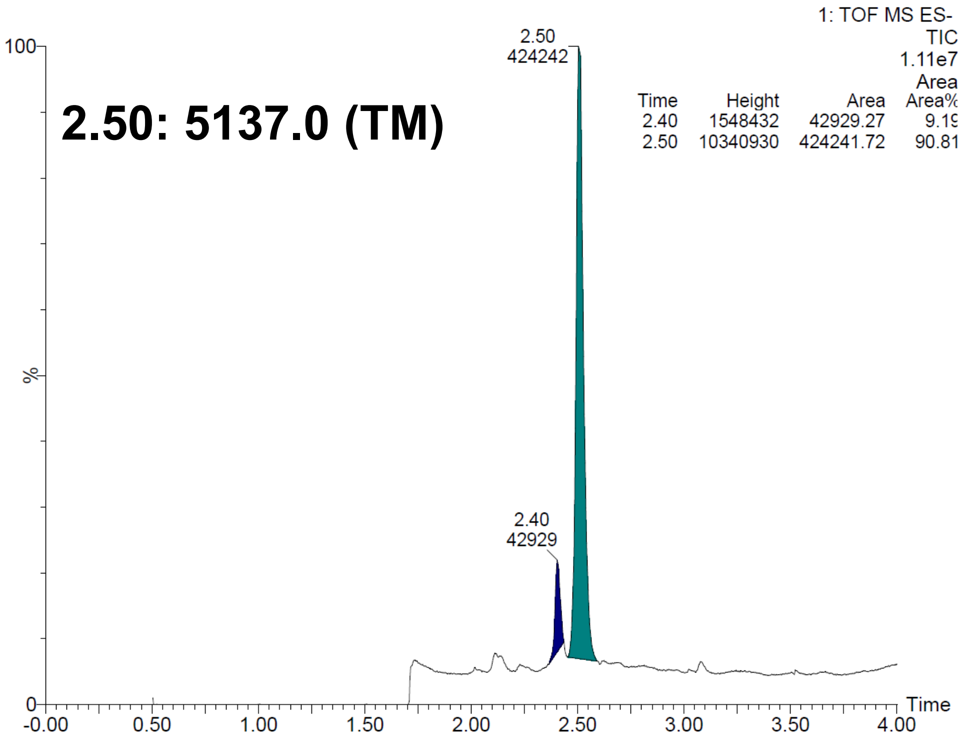

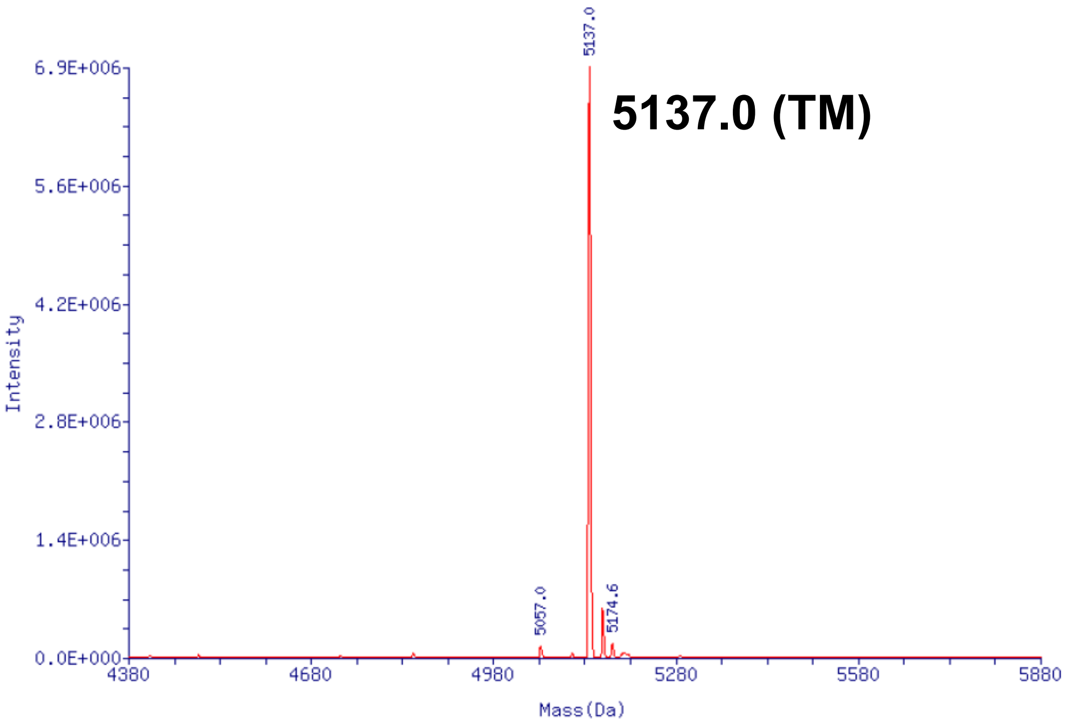

6a

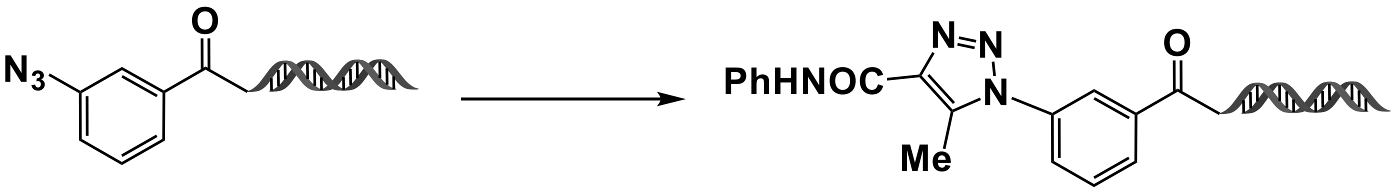

calcd.: 5241.4

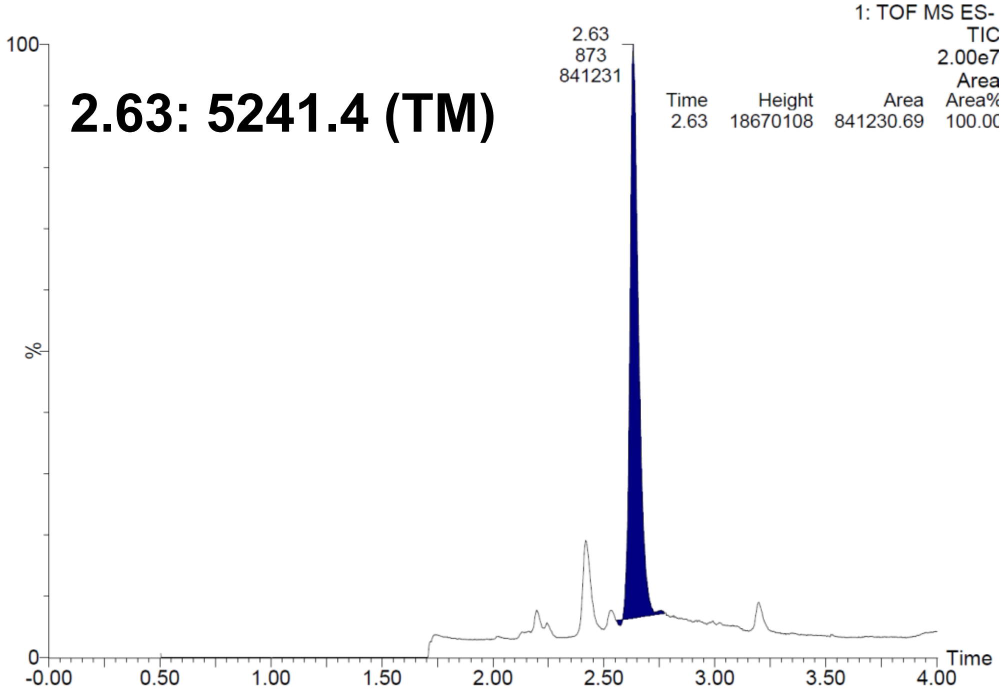

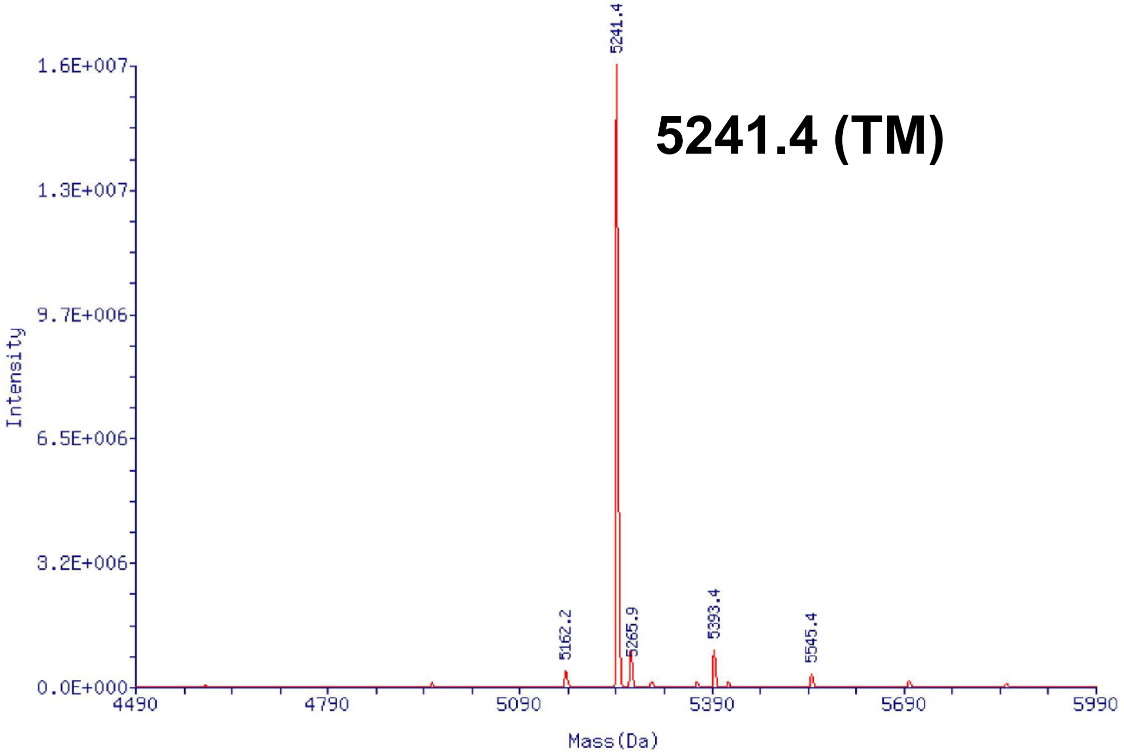

6b

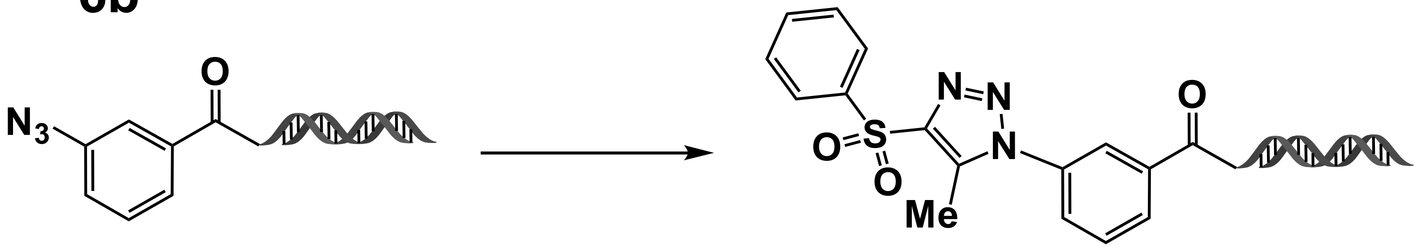

calcd.: 5262.0

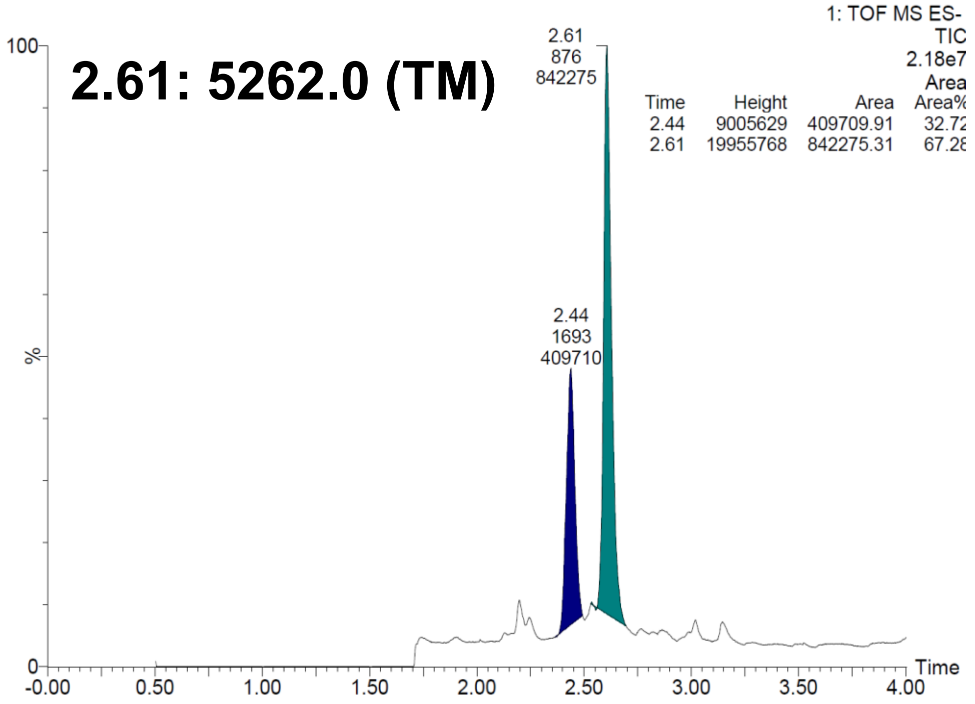

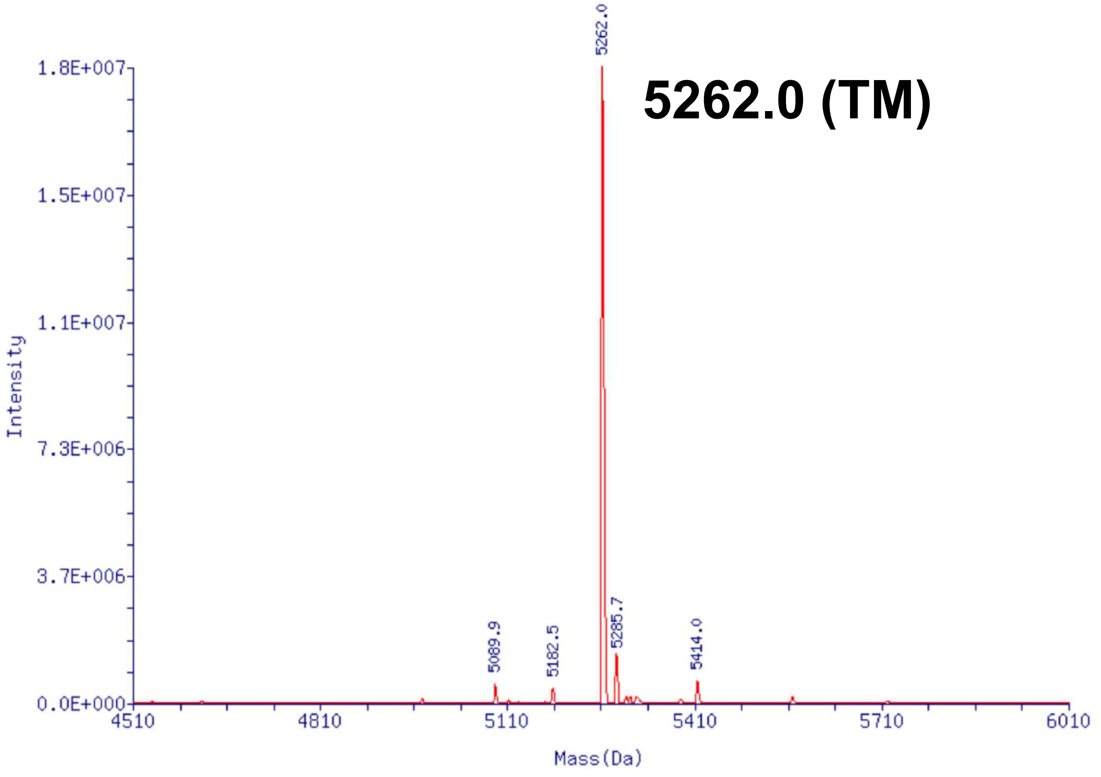

6c

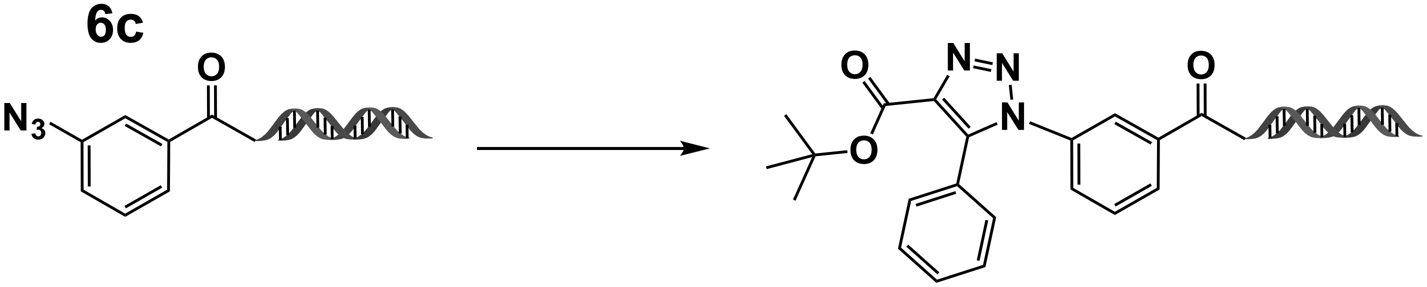

calcd.: 5284.1

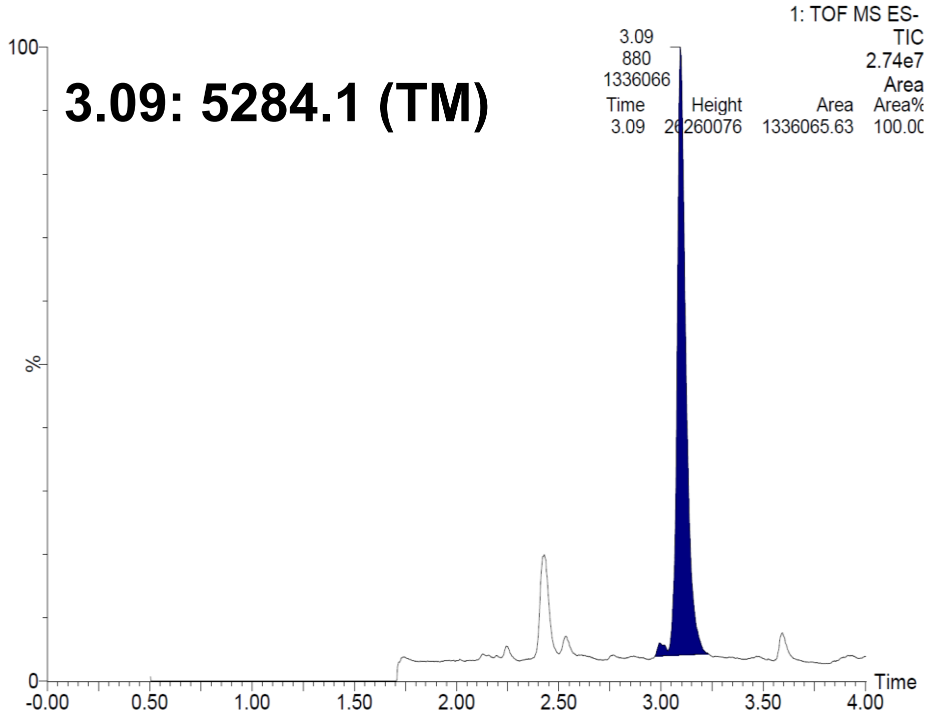

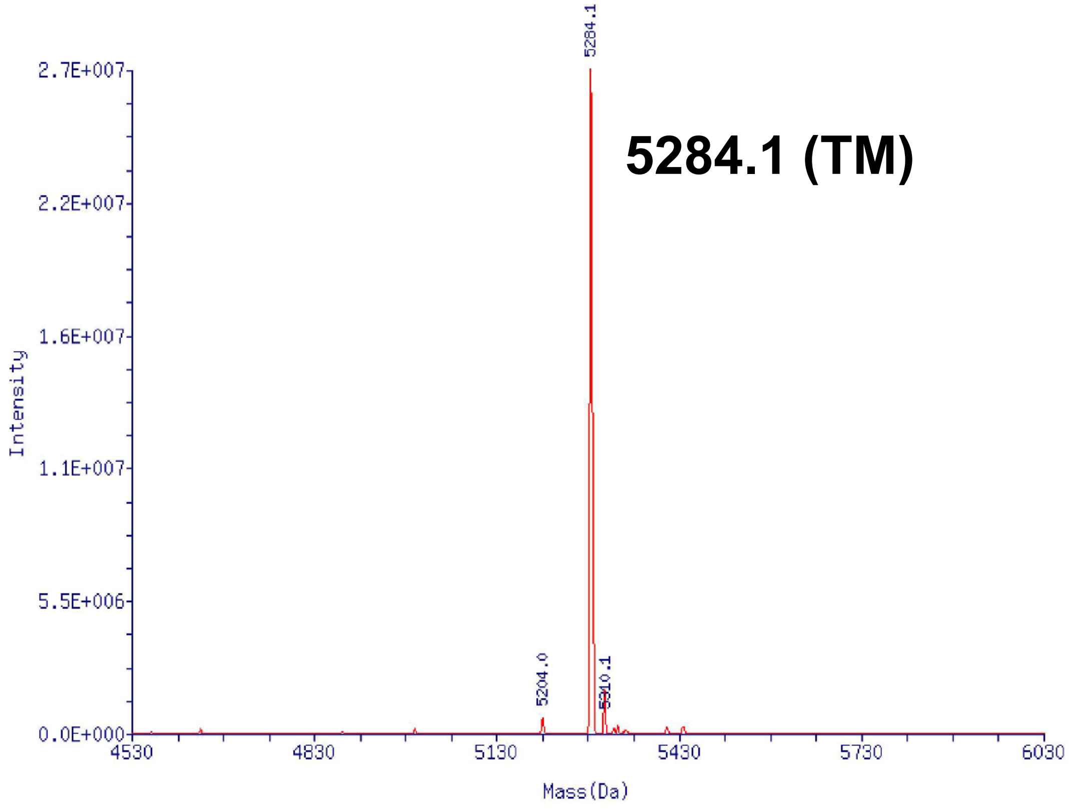

6d

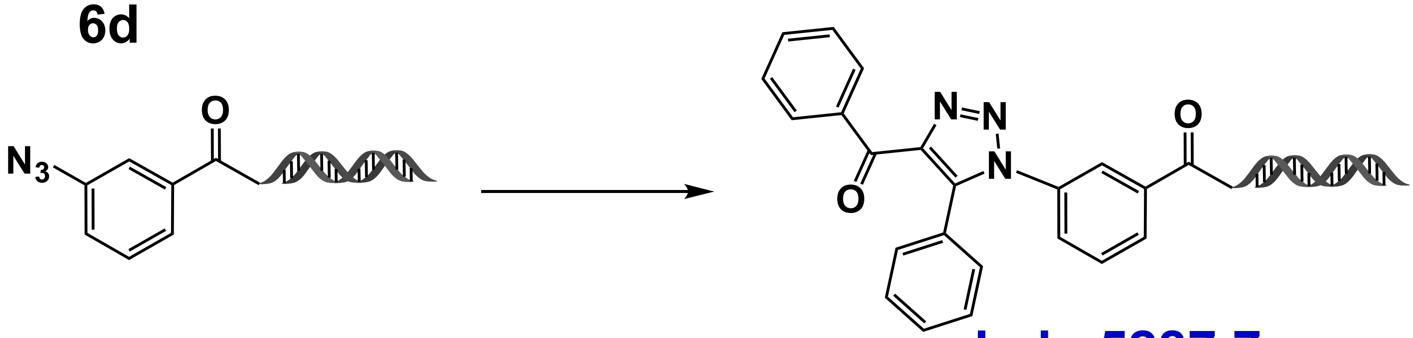

calcd.: 5287.7

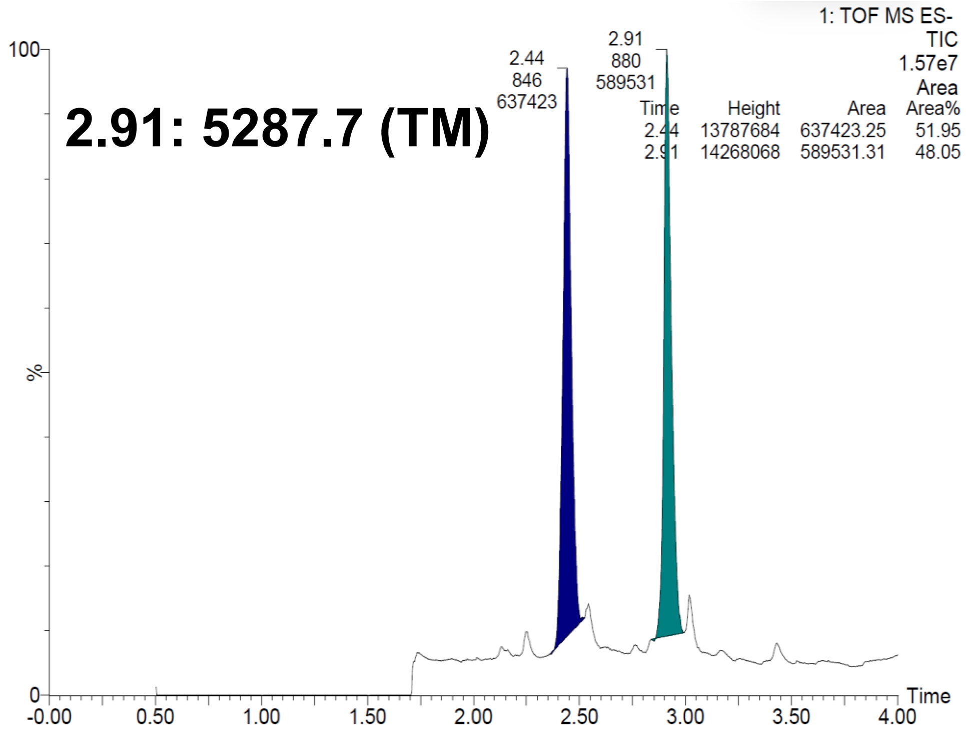

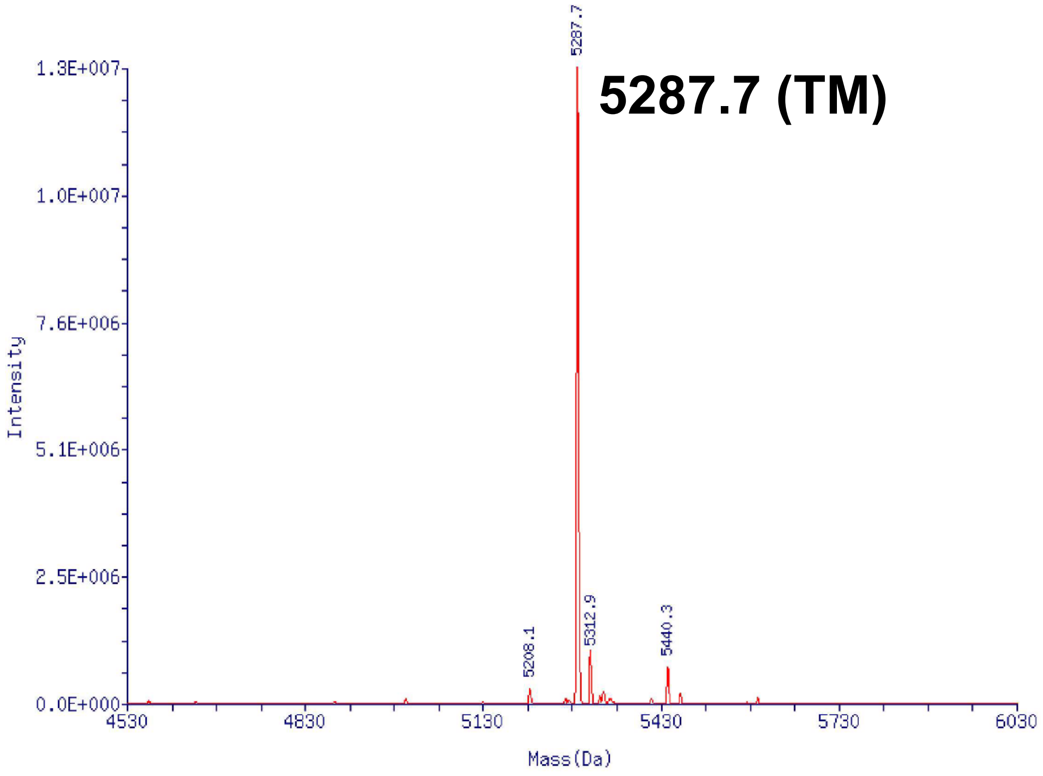

6e

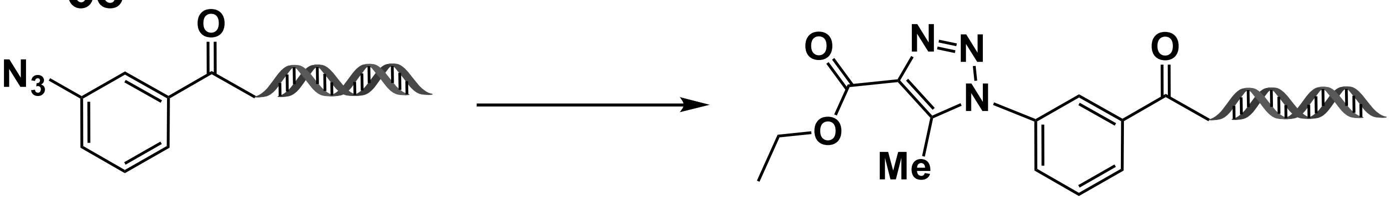

calcd.: 5193.9

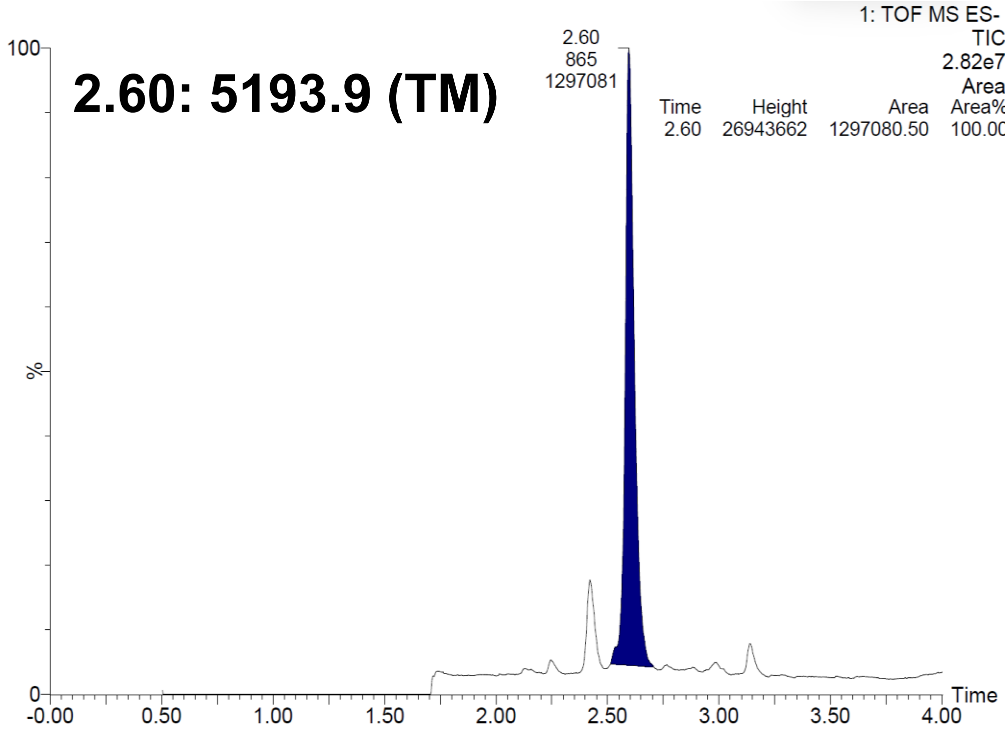

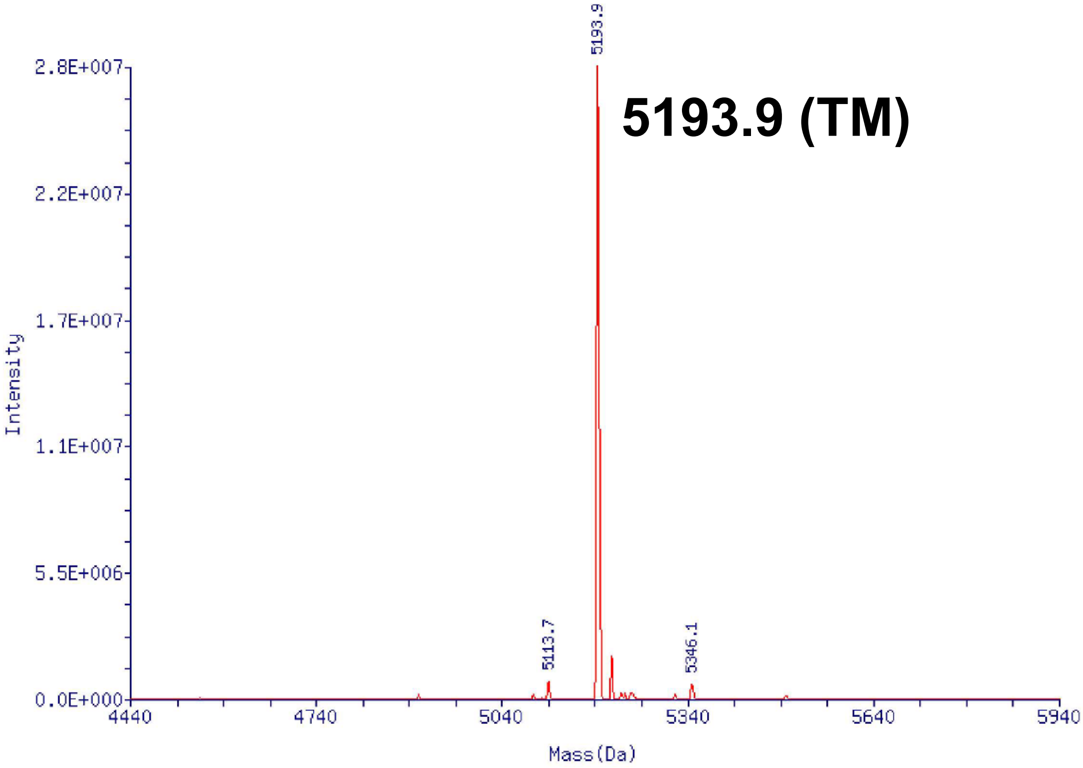

6f

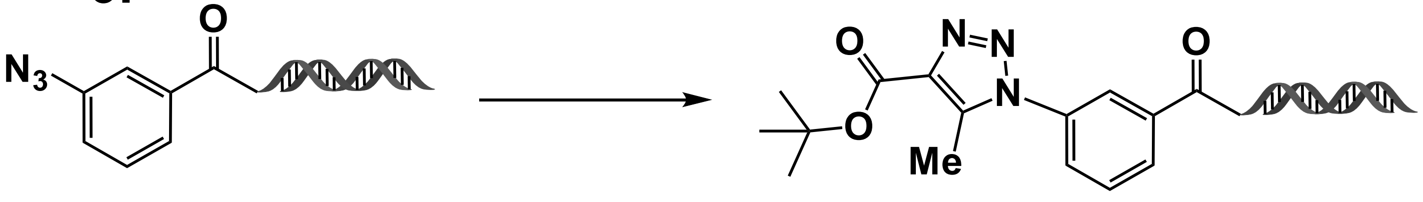

calcd.: 5222.1

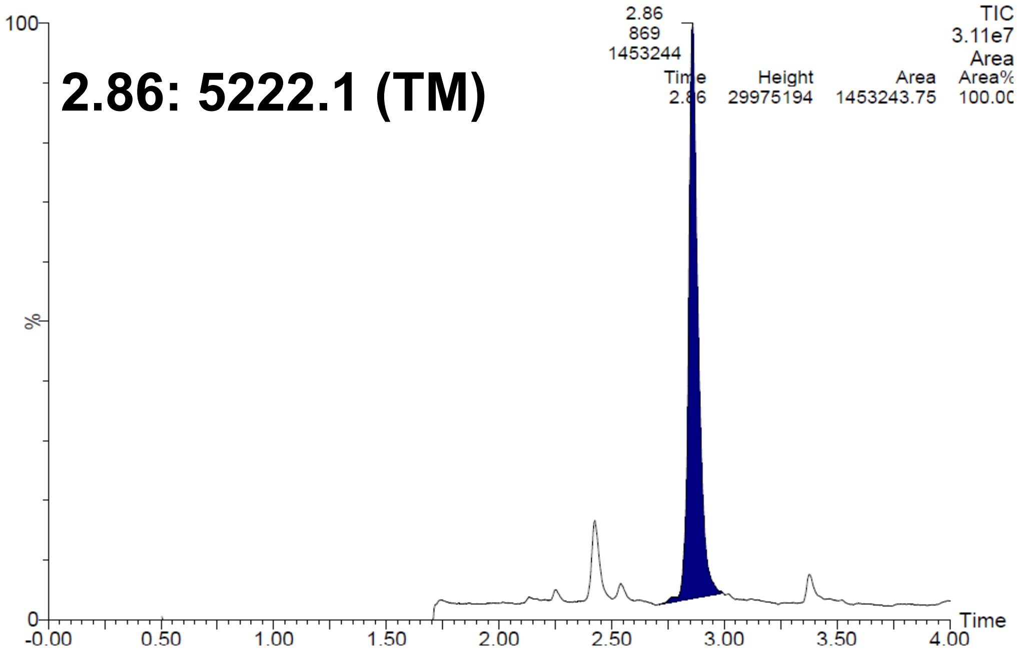

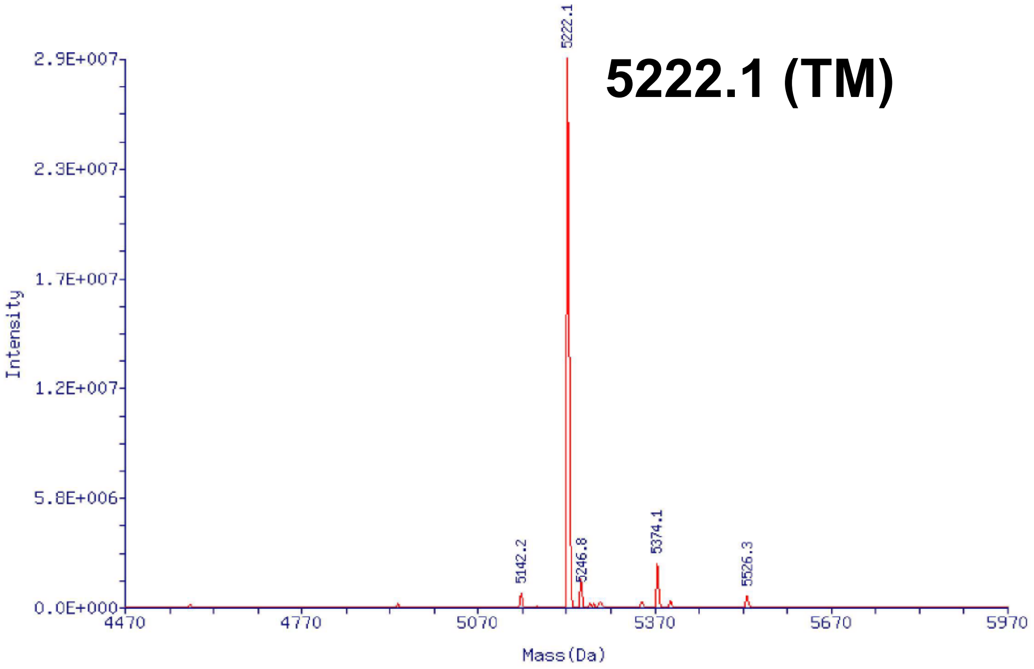

6g

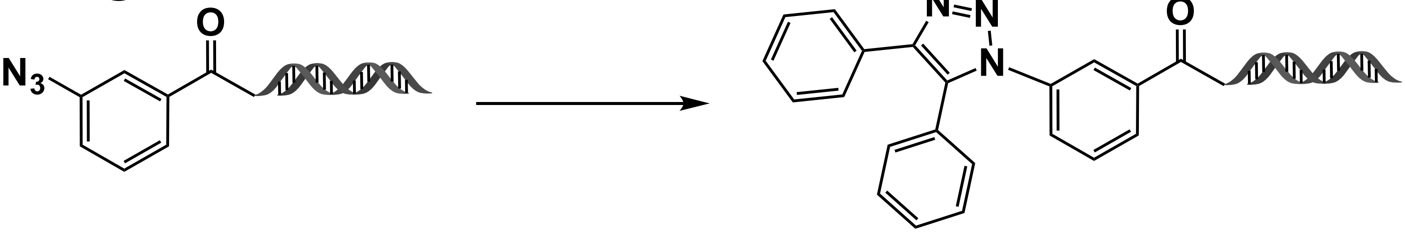

calcd.: 5260.0

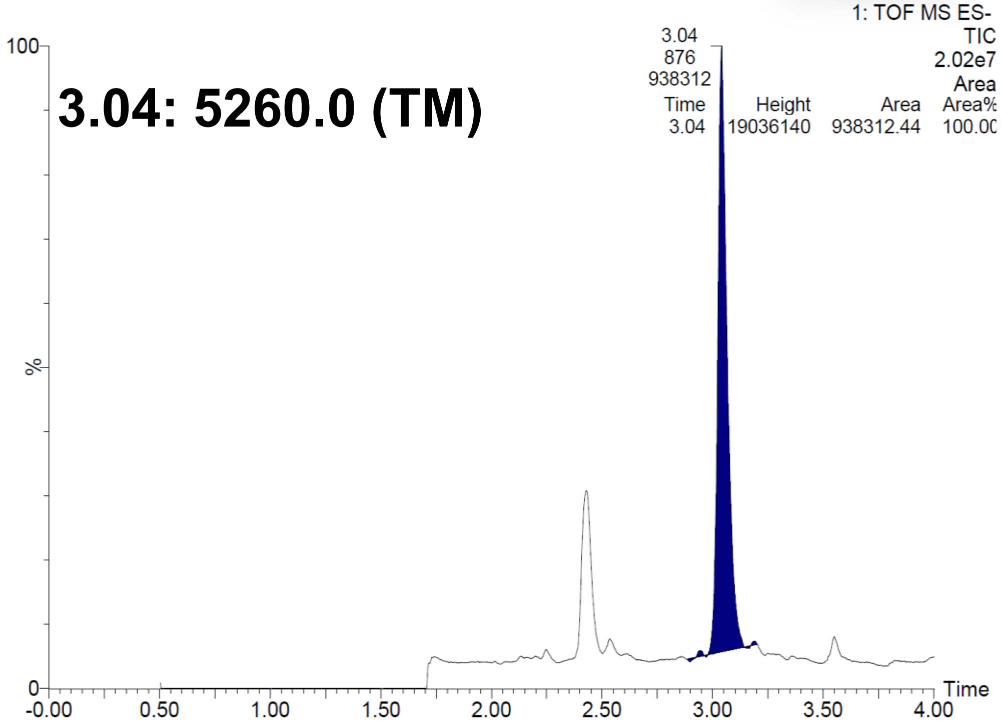

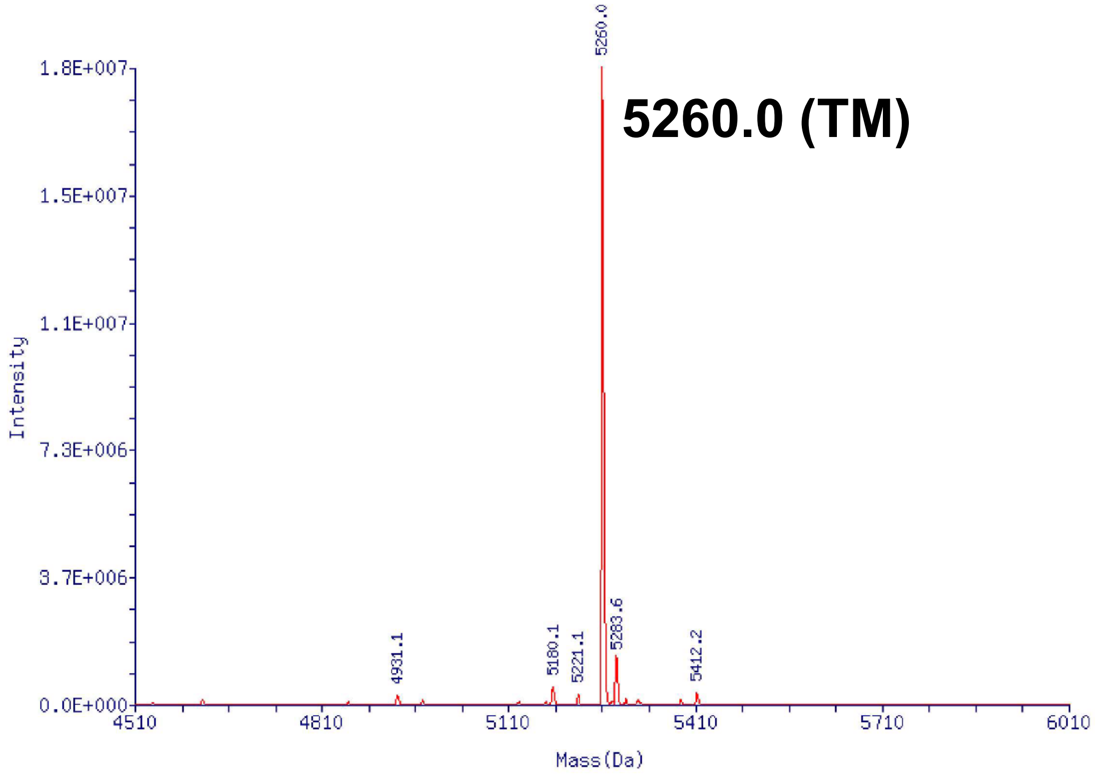

6h

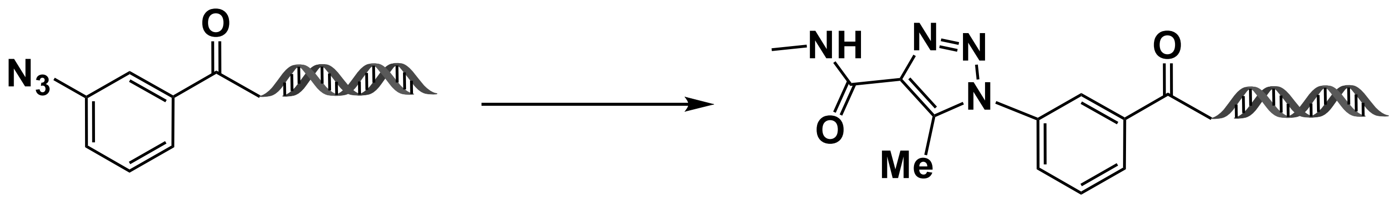

calcd.: 5179.0

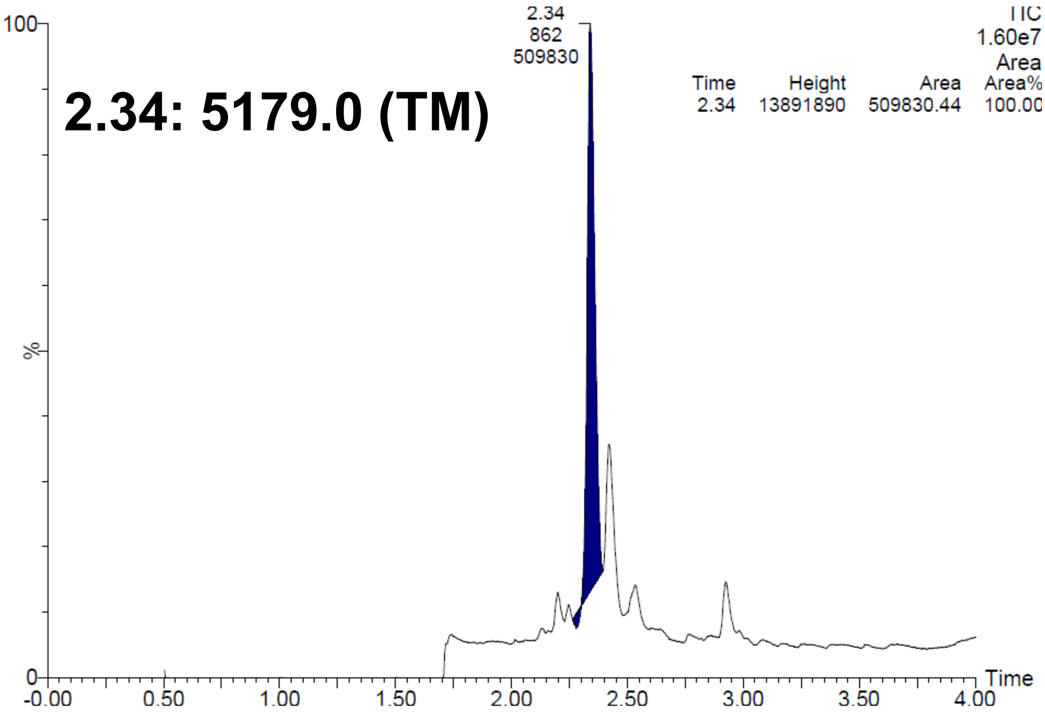

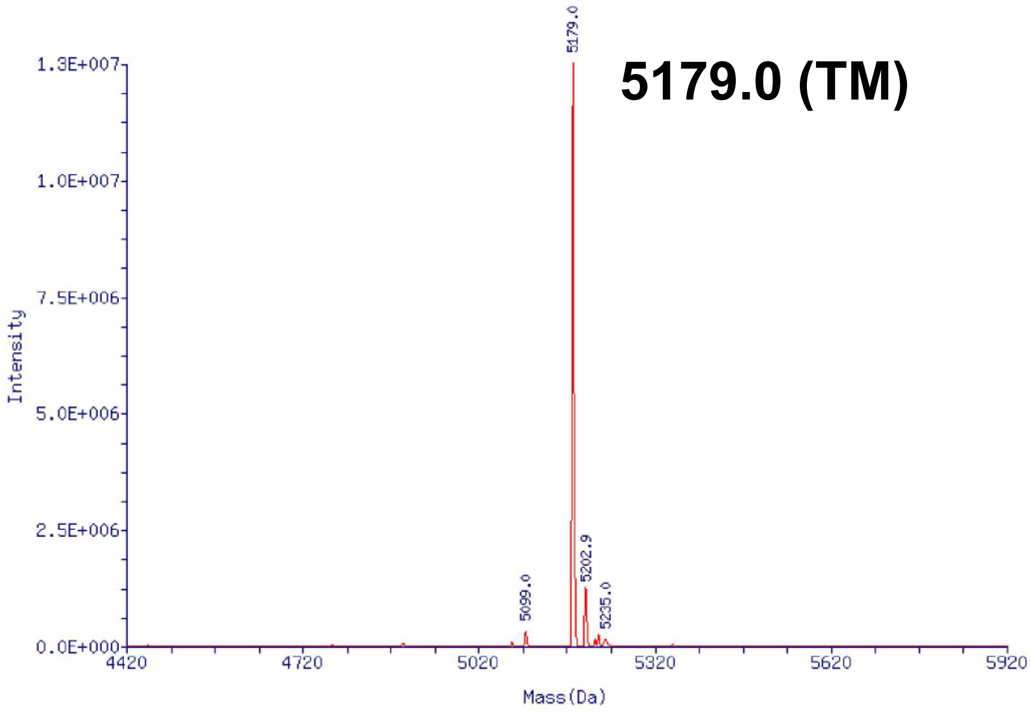

6i

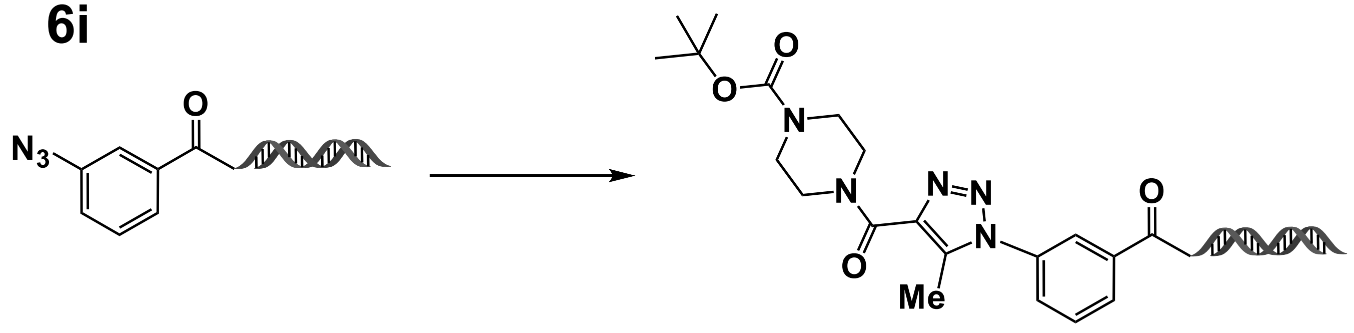

calcd.: 5334.1

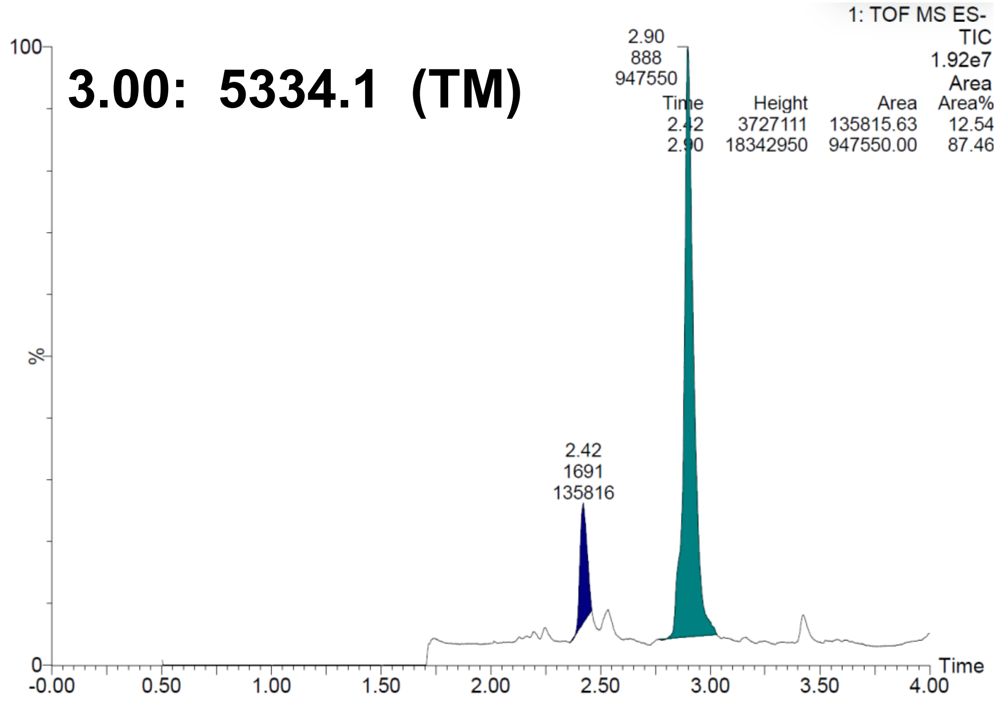

3.00: 5334.1 (TM)
1: TOF MS ES-
TIC
1.92e7
Area
Time Height Area Area%
2.42 3727111 135815.63 12.54
2.90 18342950 947550.00 87.46
2.90
888
947550
2.42
1691
135816
%
Time

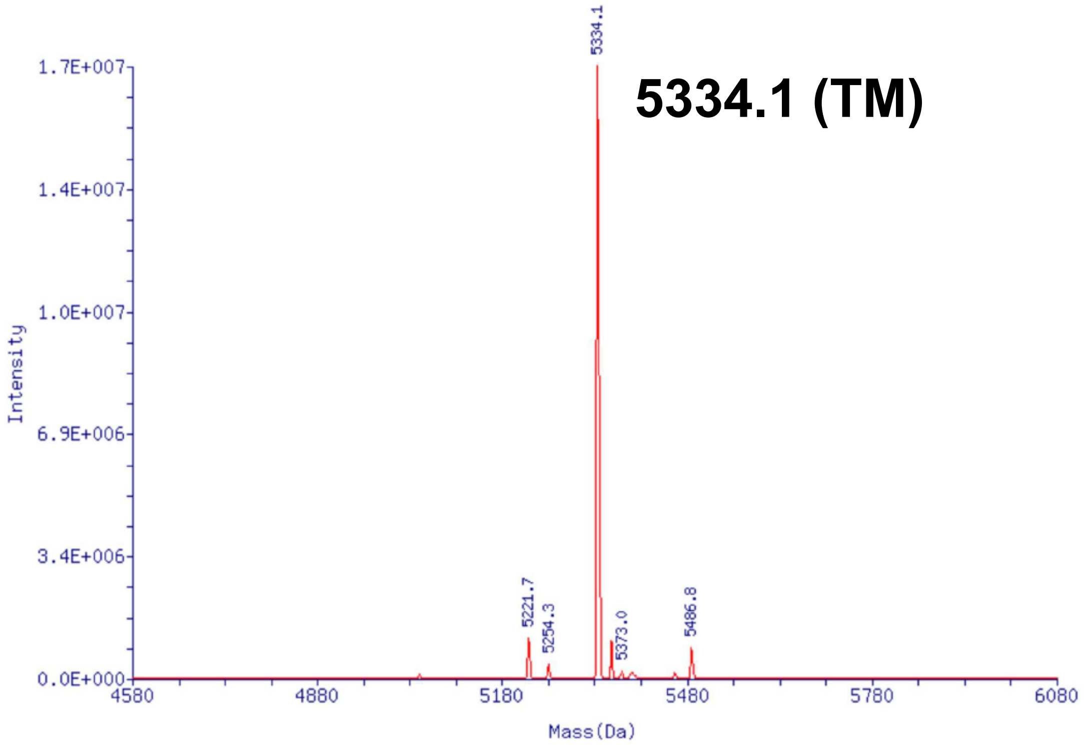

5334.1 (TM)
5334.1
5221.7
5254.3
5373.0
5486.8
Intensity
Mass(Da)

6j

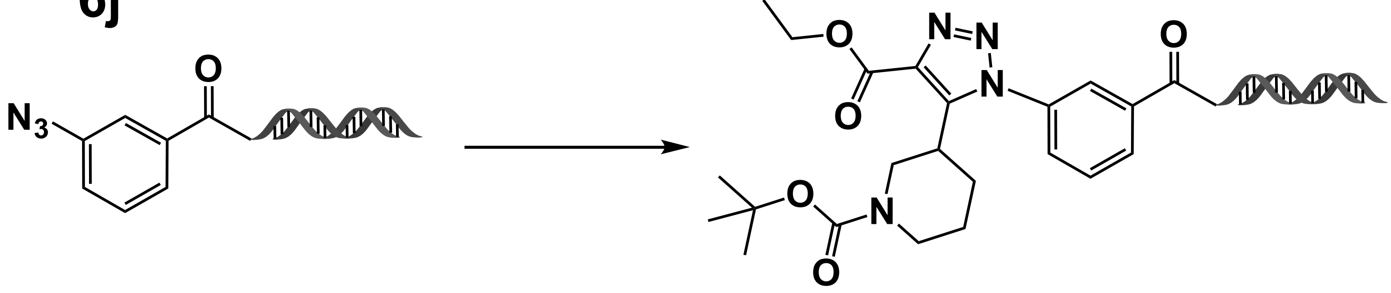

N3
O
N=N
N
O
O
O
N
O
calcd.: 5363.3

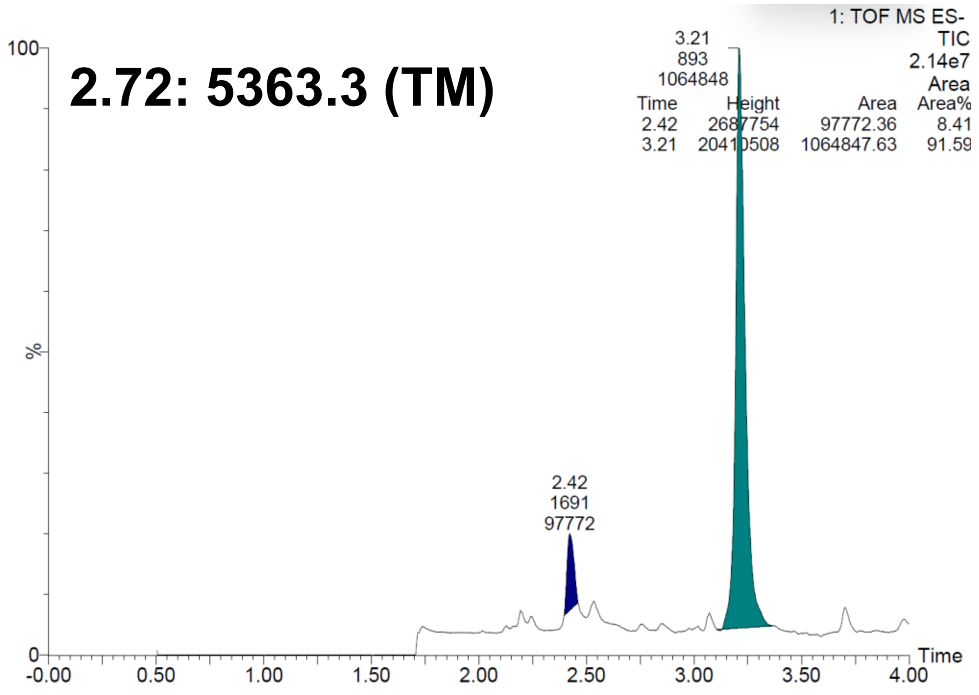

2.72: 5363.3 (TM)
1: TOF MS ES-
TIC
2.14e7
Area
3.21
893
1064848
Time Height Area Area%
2.42 2687754 97772.36 8.41
3.21 20410508 1064847.63 91.59
2.42
1691
97772
100
%
0
-0.00 0.50 1.00 1.50 2.00 2.50 3.00 3.50 4.00
Time

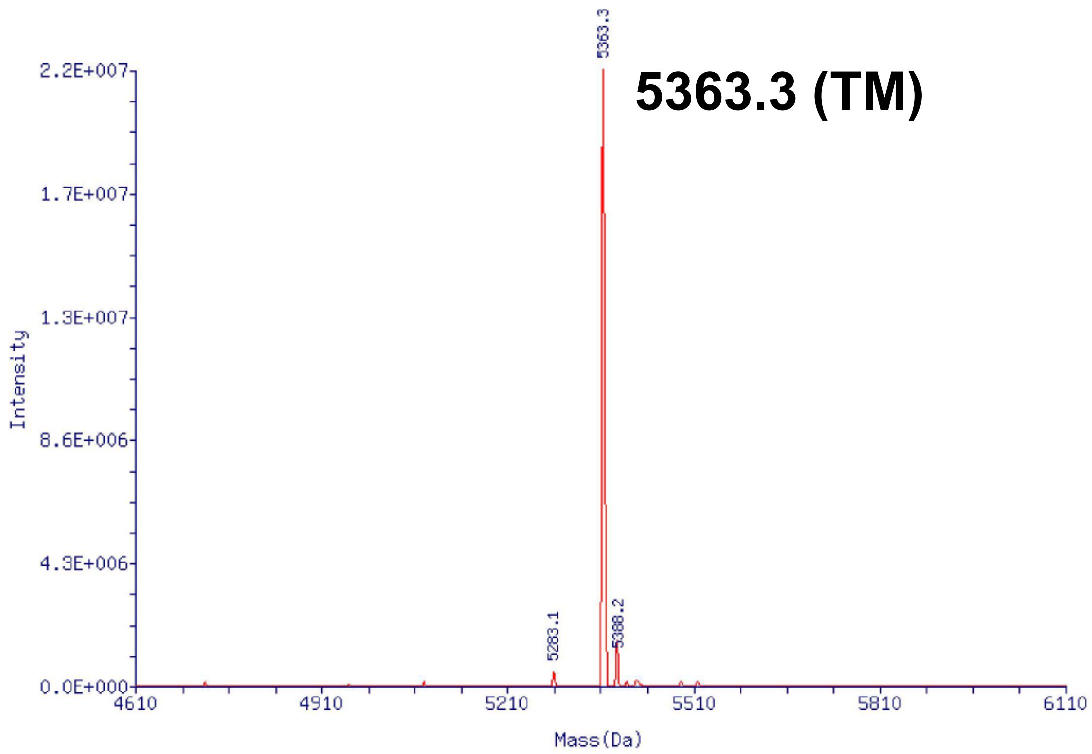

5363.3
5363.3 (TM)
5283.1
5388.2
2.2E+007
1.7E+007
1.3E+007
8.6E+006
4.3E+006
0.0E+000
Intensity
4610 4910 5210 5510 5810 6110
Mass(Da)

6k

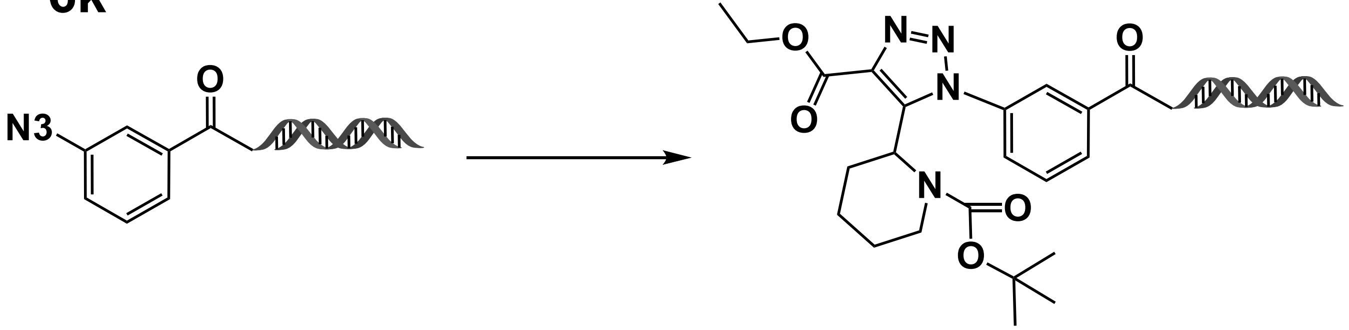

calcd.: 5363.3

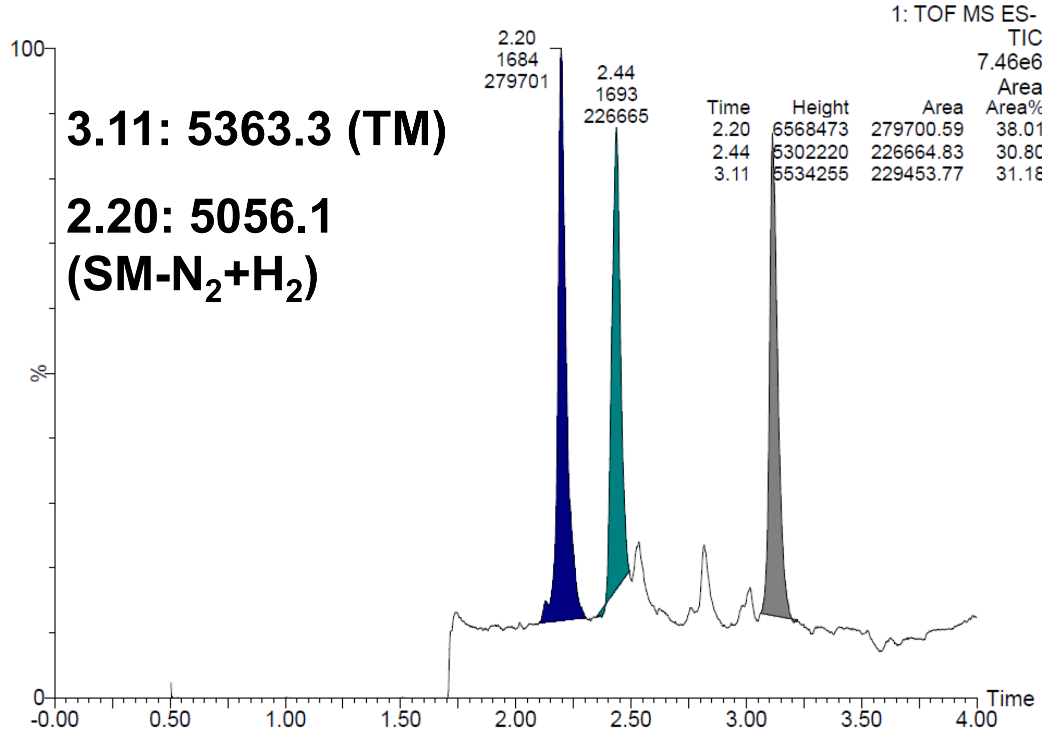

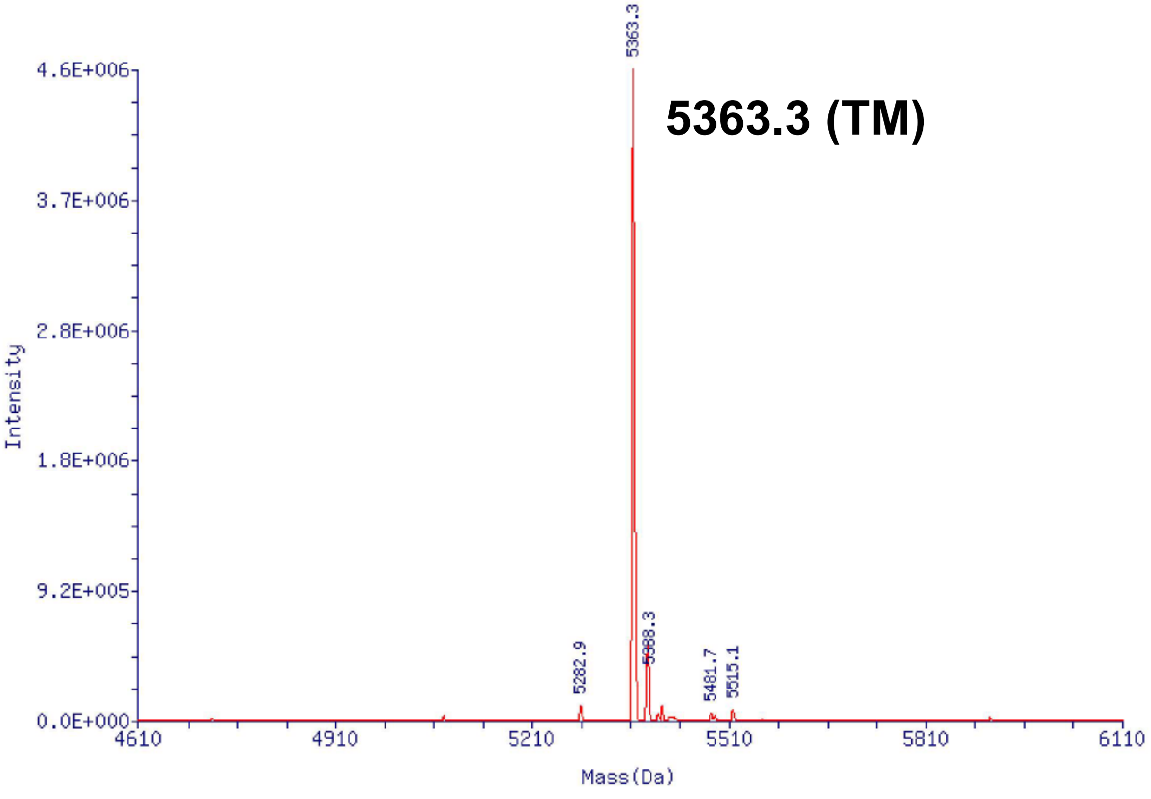

6l

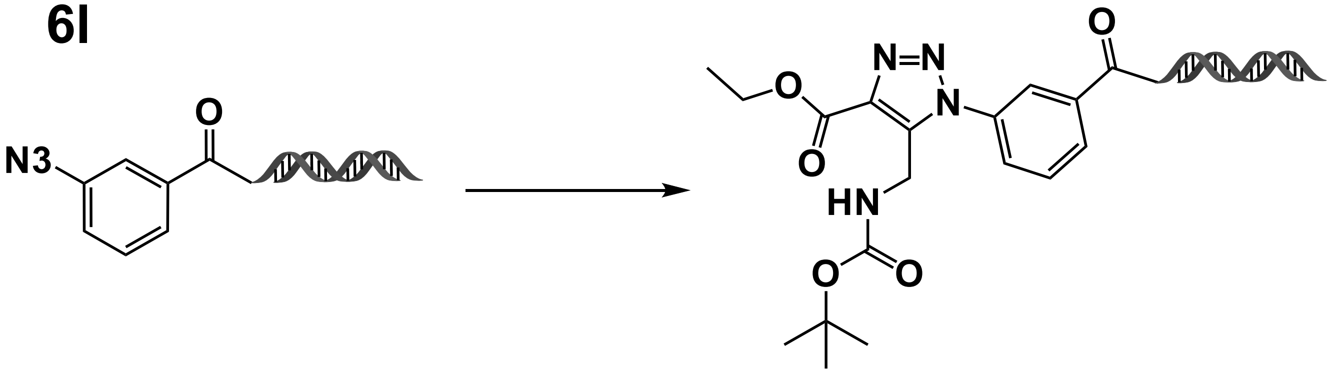

calcd.: 5309.1

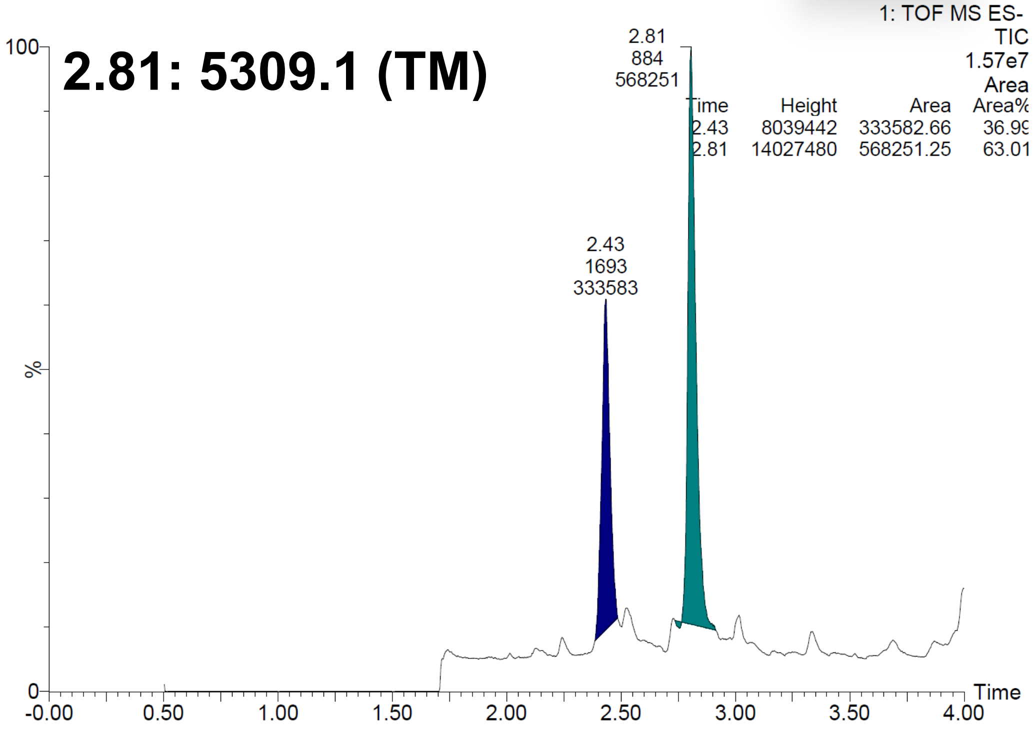

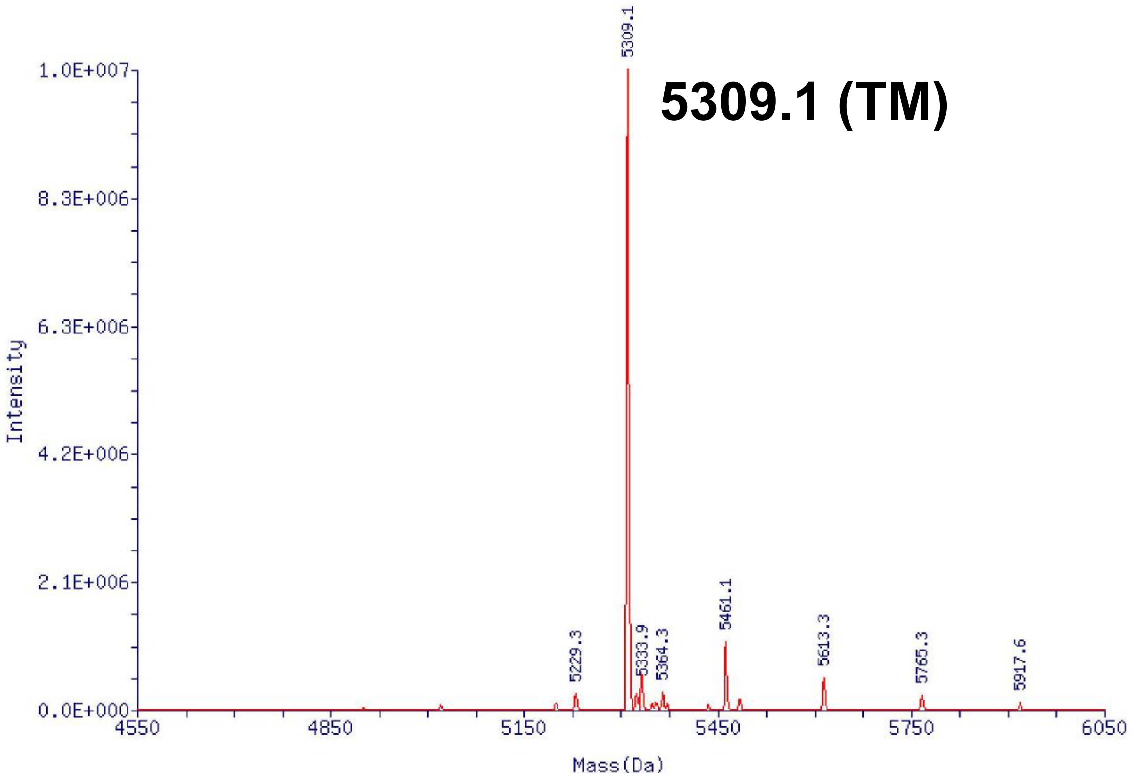

6m

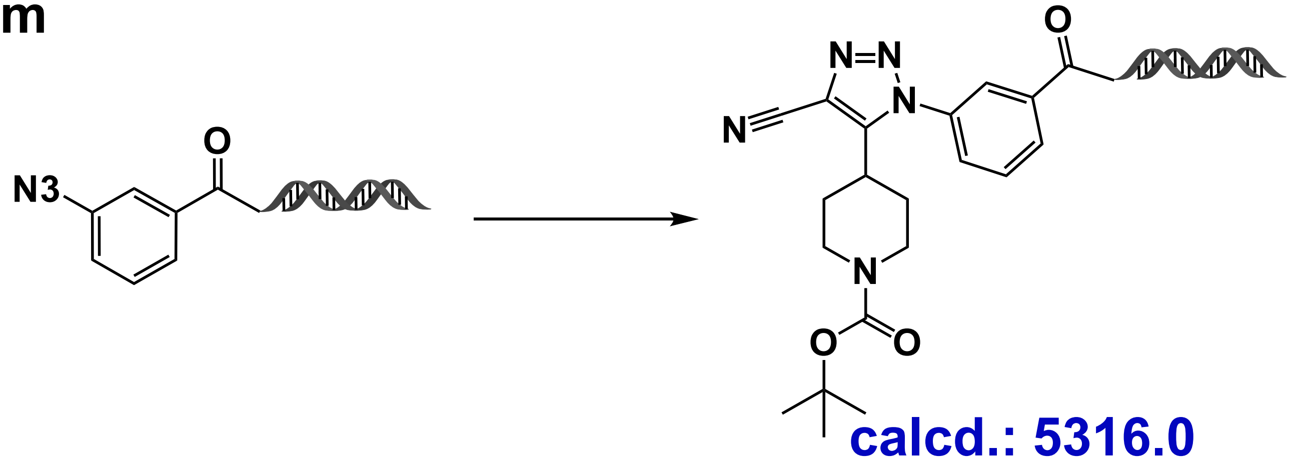

calcd.: 5316.0

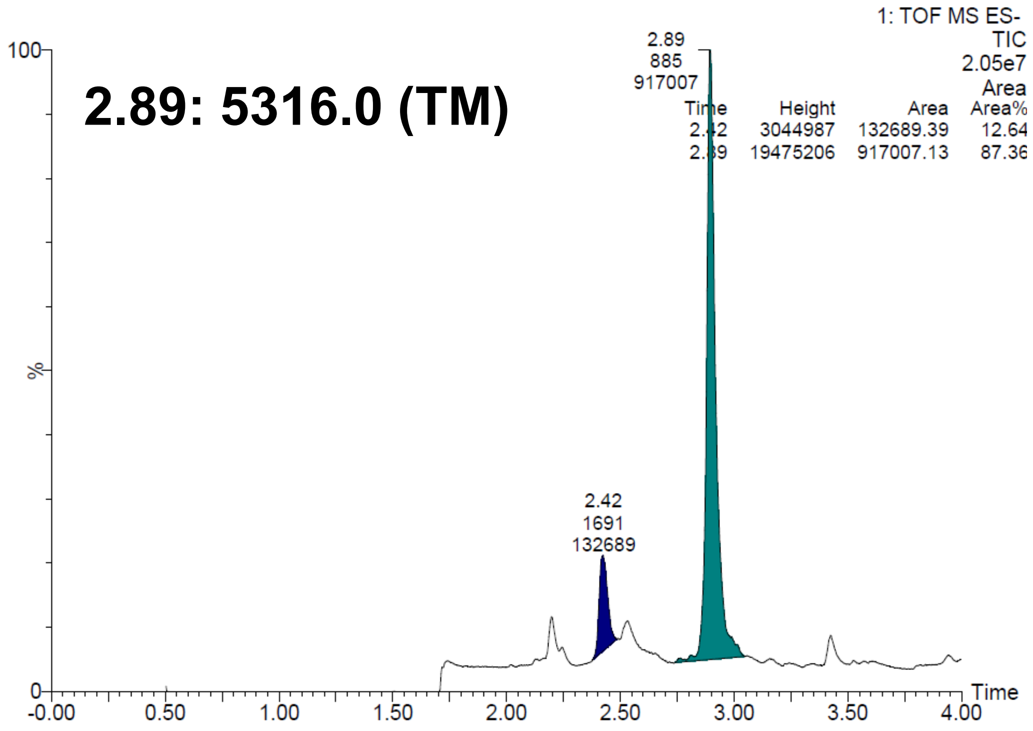

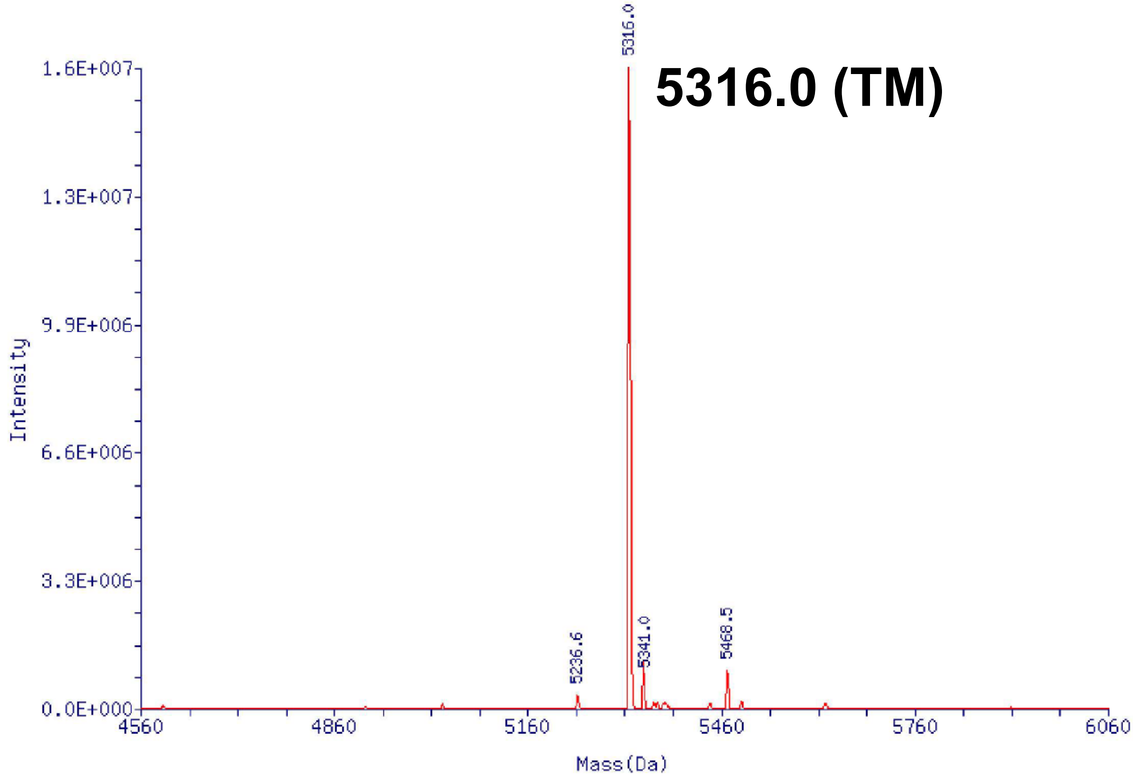

6n

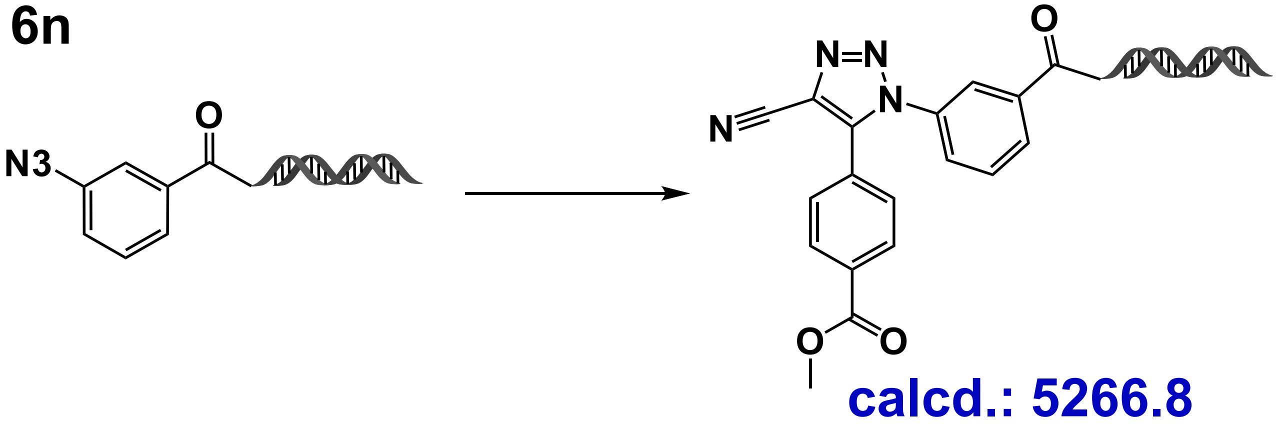

N3
O
N=N
N
N
O
O
O
calcd.: 5266.8

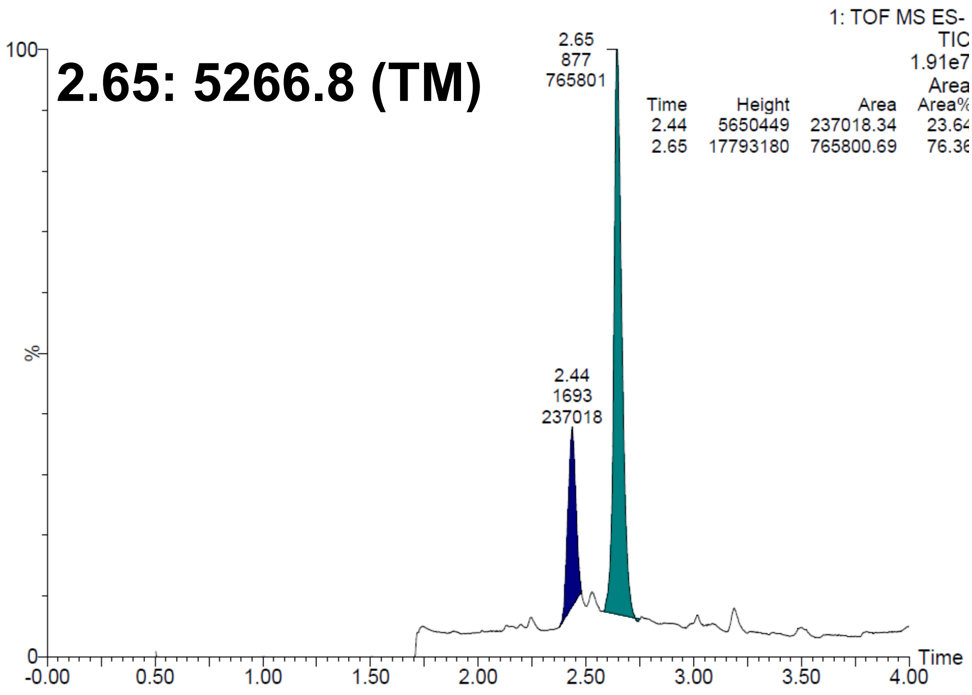

1: TOF MS ES-
TIC
1.91e7
Area
2.65: 5266.8 (TM)
2.65
877
765801
Time Height Area Area%
2.44 5650449 237018.34 23.64
2.65 17793180 765800.69 76.36
2.44
1693
237018
100
%
0
-0.00
0.50
1.00
1.50
2.00
2.50
3.00
3.50
4.00
Time

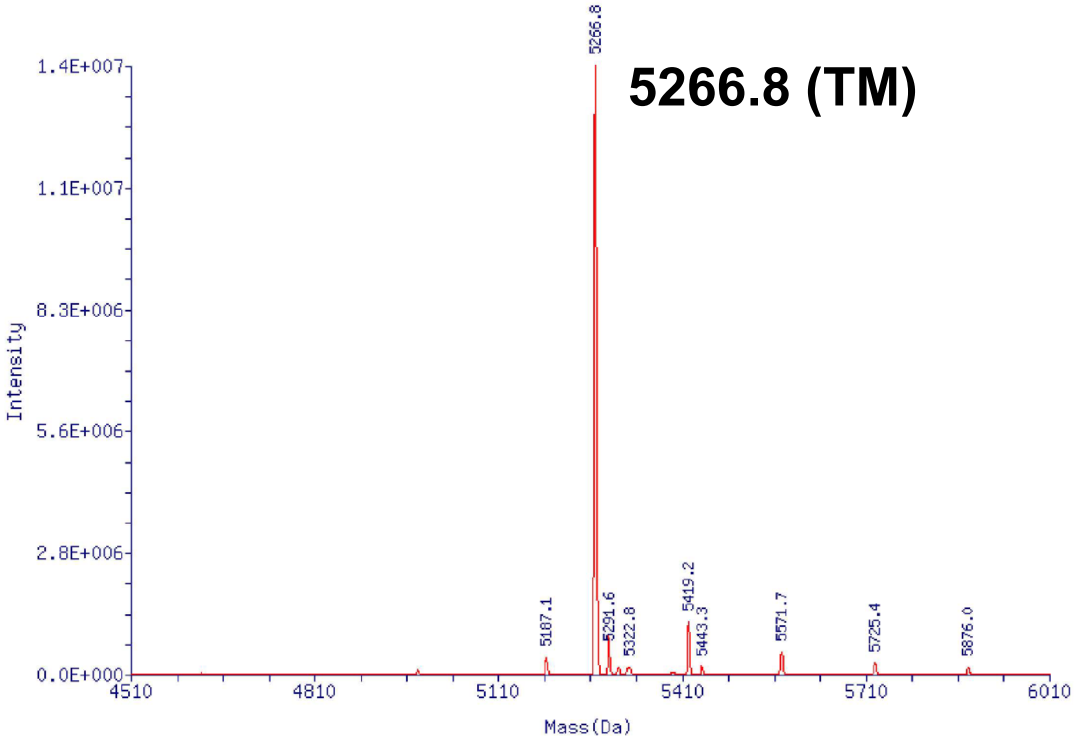

5266.8
5266.8 (TM)
1.4E+007
1.1E+007
8.3E+006
5.6E+006
2.8E+006
0.0E+000
Intensity
5187.1
5291.6
5322.8
5419.2
5443.3
5571.7
5725.4
5876.0
4510
4810
5110
5410
5710
6010
Mass(Da)

6a'

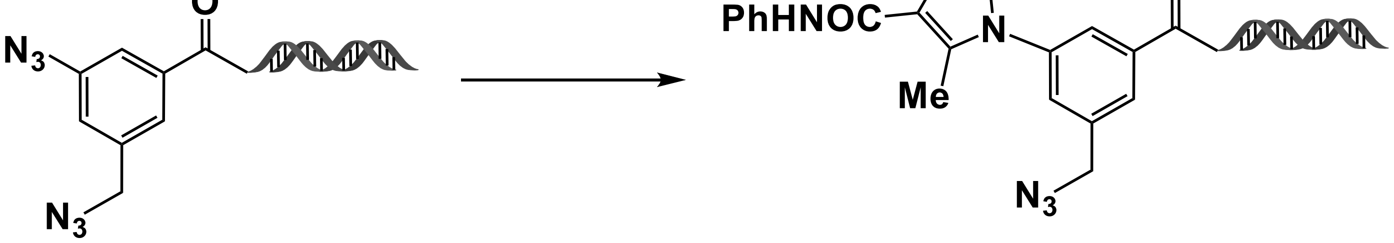

calcd.: 5296.0

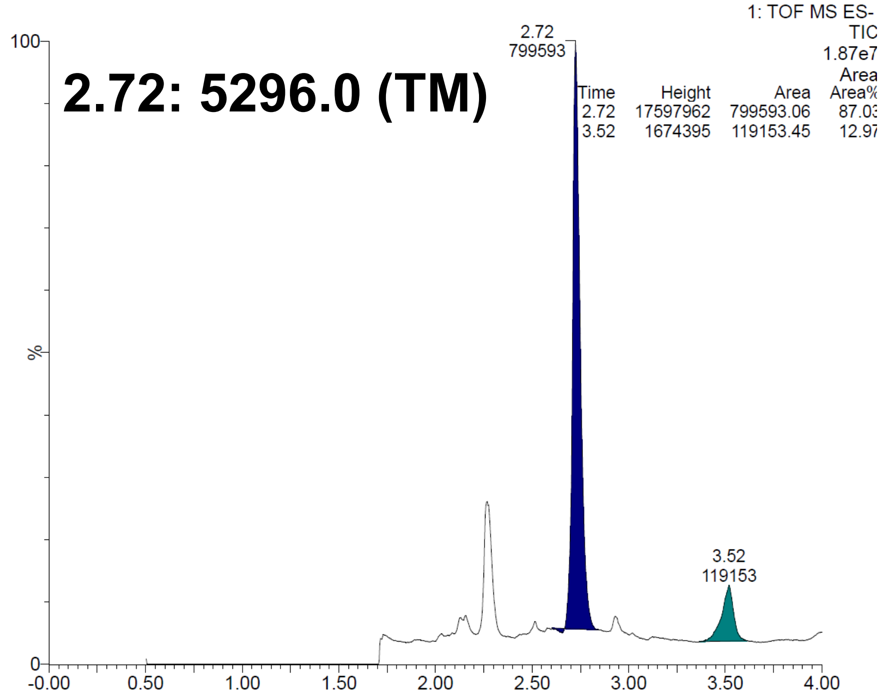

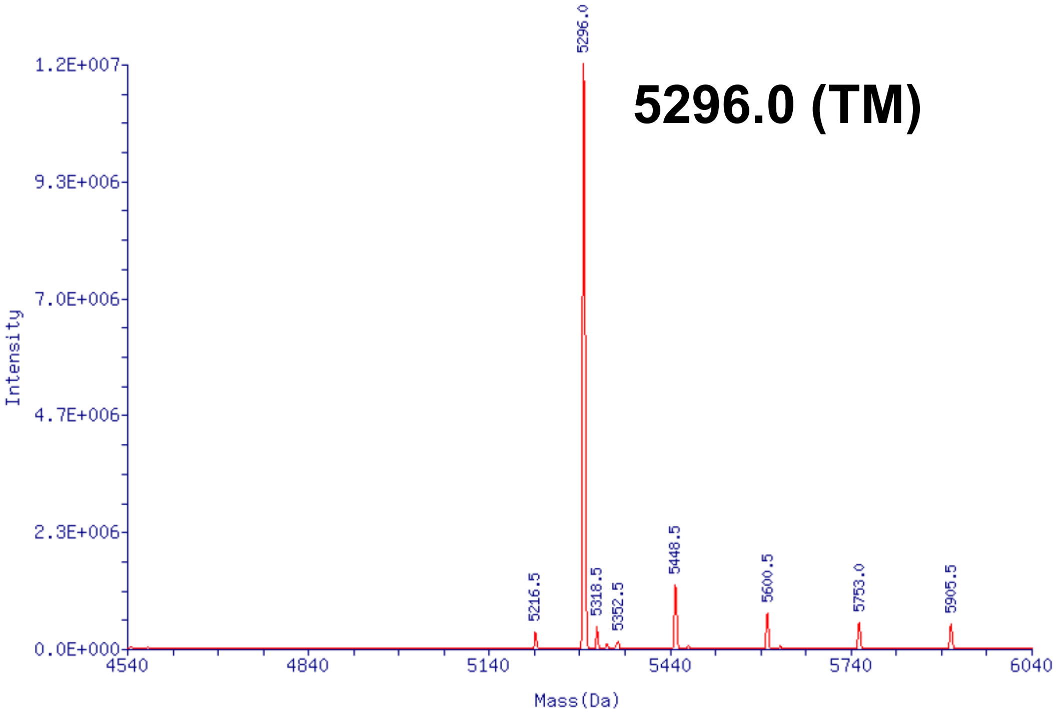

6b’

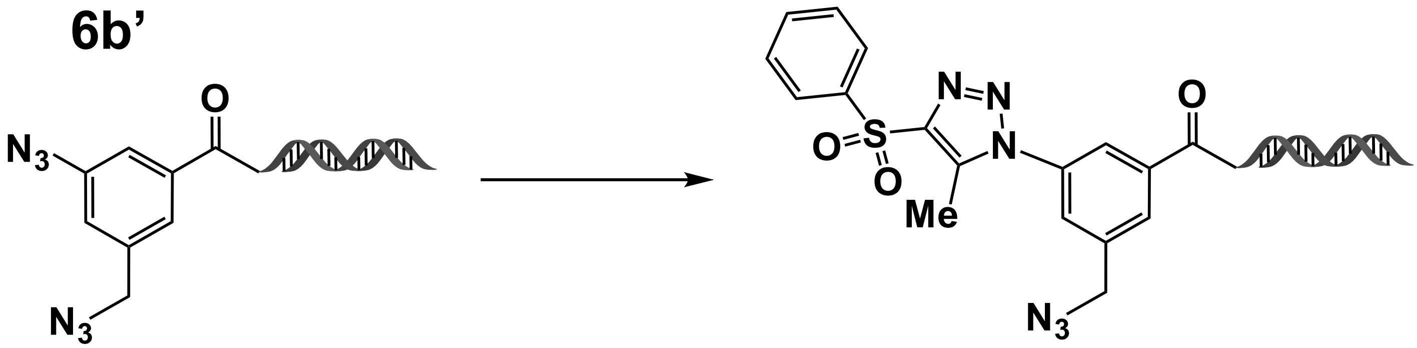

calcd.: 5317.5

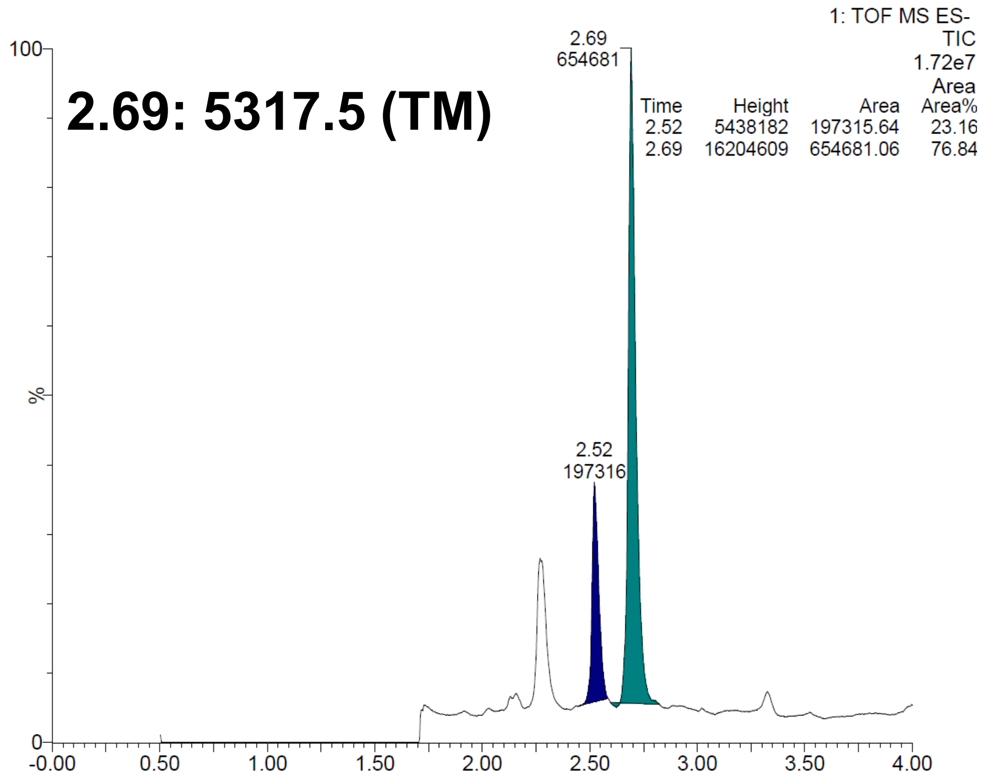

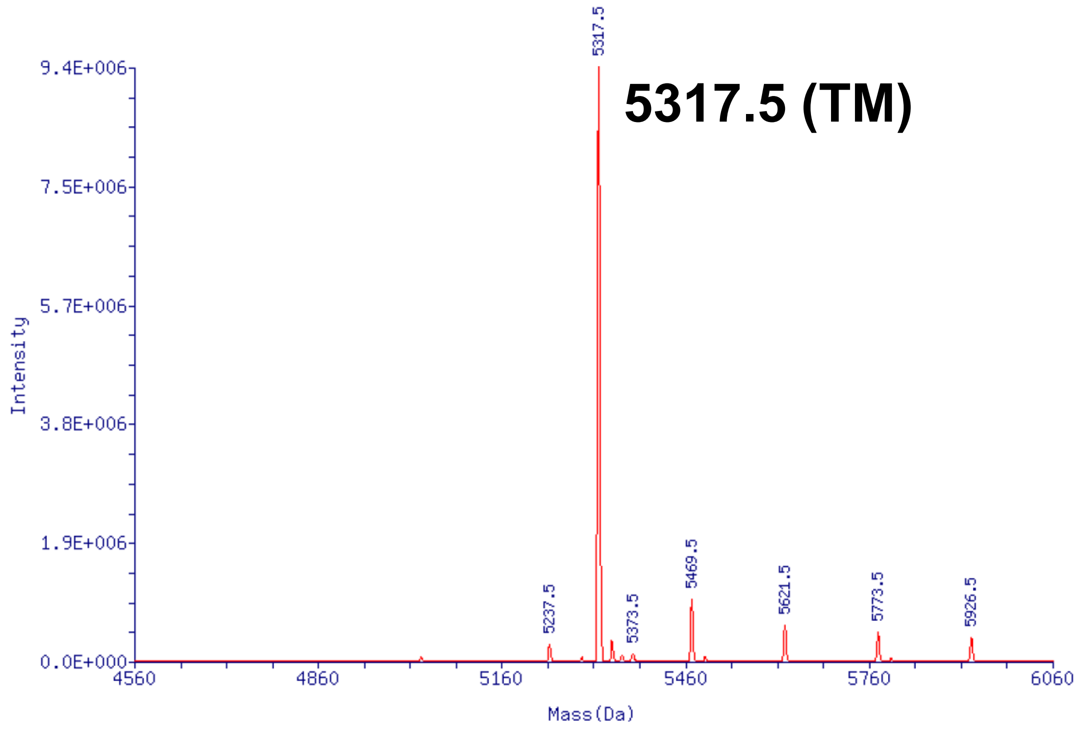

6c'

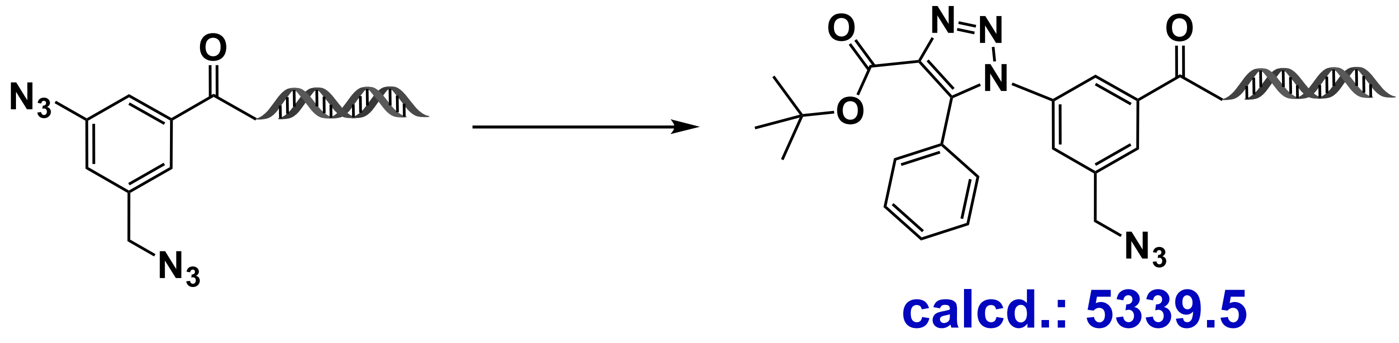
N3
O
N3
O
N=N
N
O
O
N3
calcd.: 5339.5

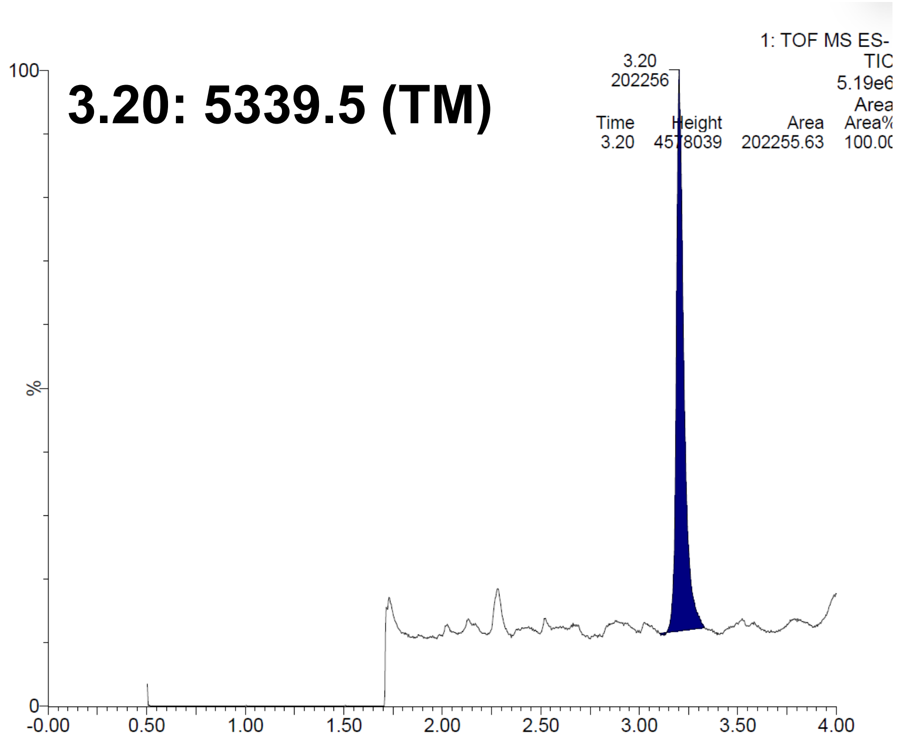
3.20: 5339.5 (TM)
1: TOF MS ES-
TIC
5.19e6
Area
3.20
202256
Time Height Area Area%
3.20 4578039 202255.63 100.00
100
%
0
-0.00
0.50
1.00
1.50
2.00
2.50
3.00
3.50
4.00

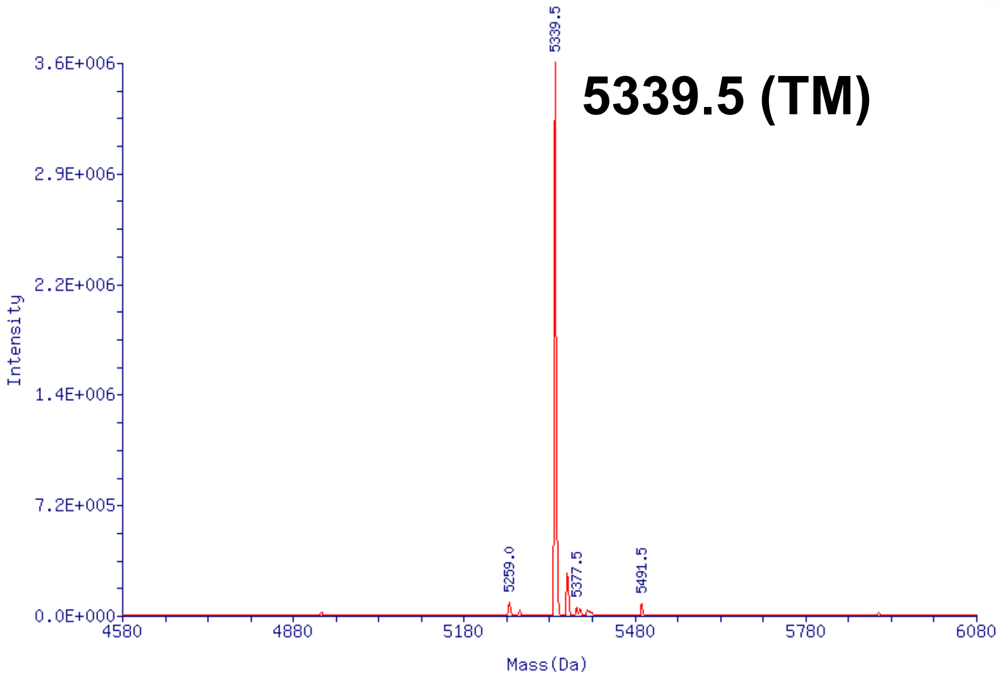
5339.5
5339.5 (TM)
3.6E+006
2.9E+006
2.2E+006
1.4E+006
7.2E+005
0.0E+000
Intensity
5259.0
5377.5
5491.5
4580
4880
5180
5480
5780
6080
Mass(Da)

6d’

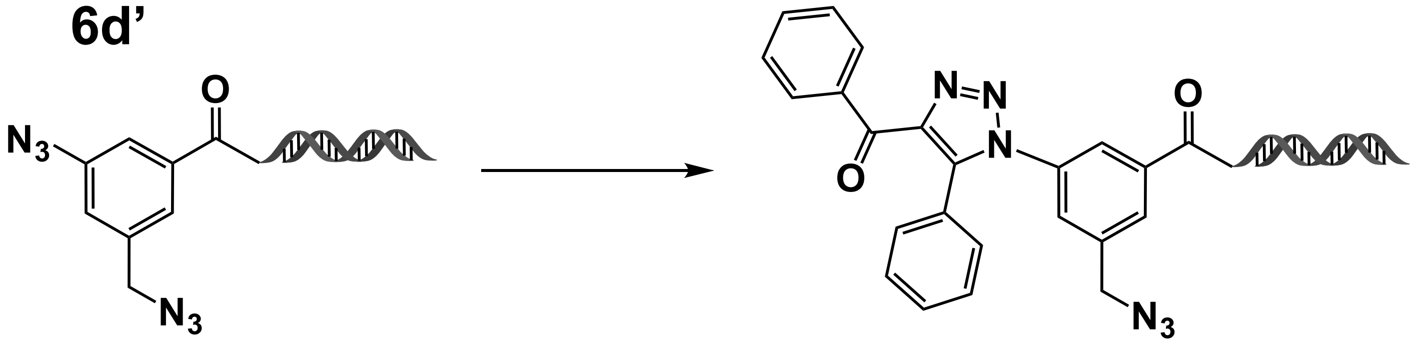

calcd.: 5343.5

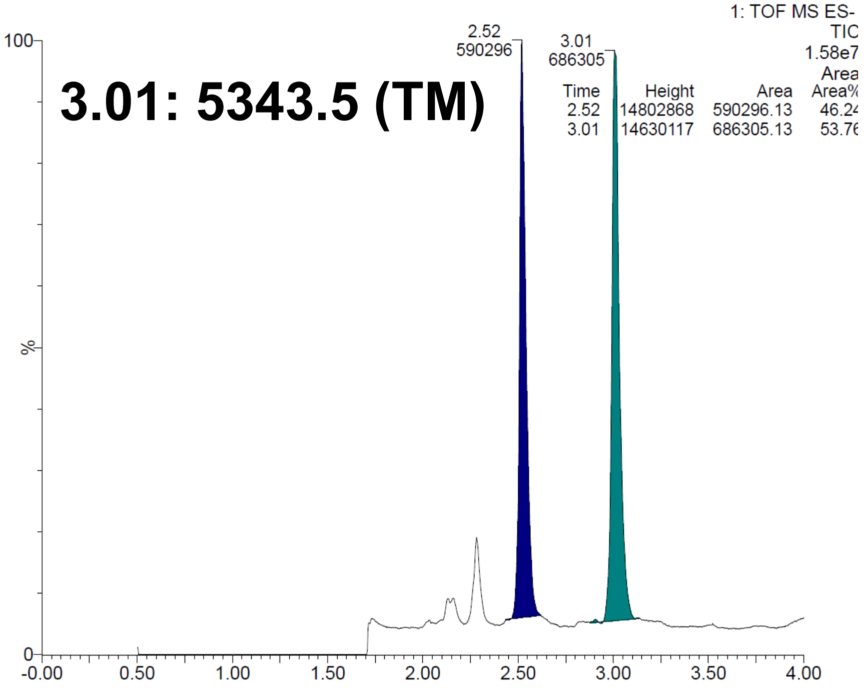

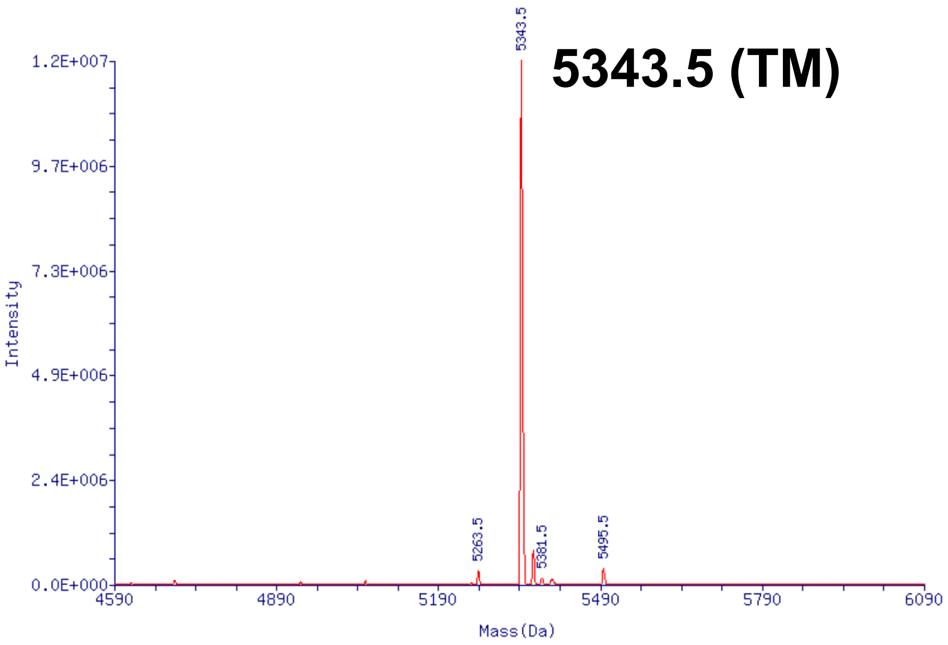

6e’

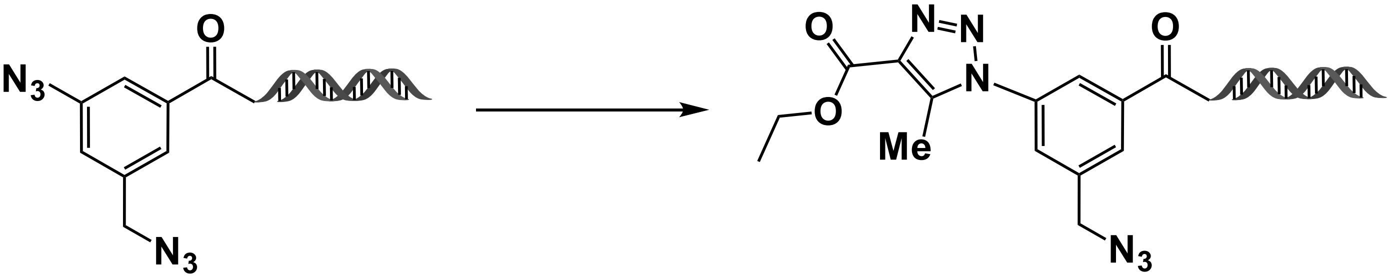

calcd.: 5248.5

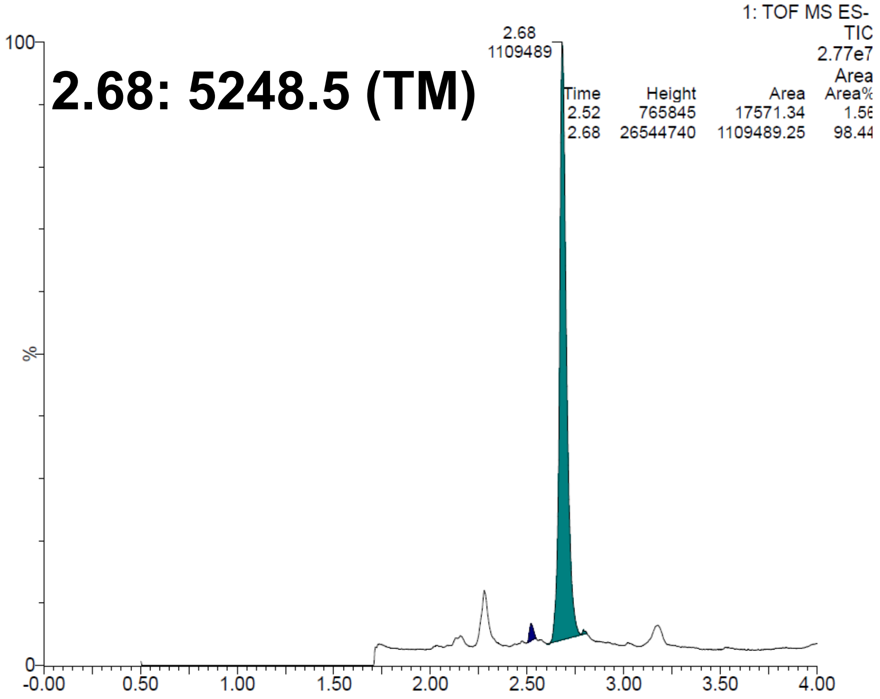

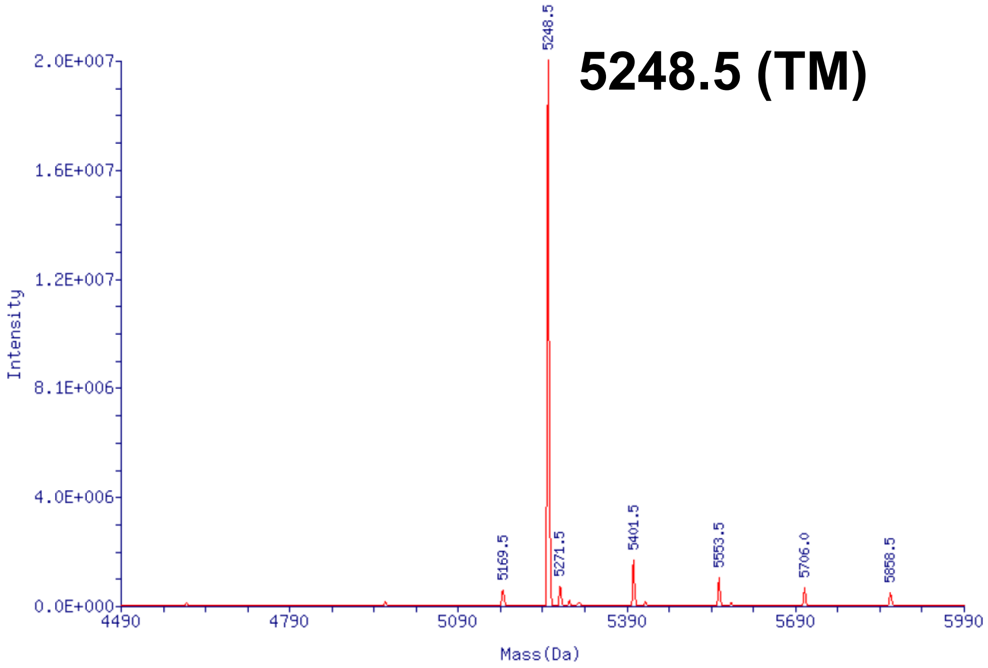

6f’

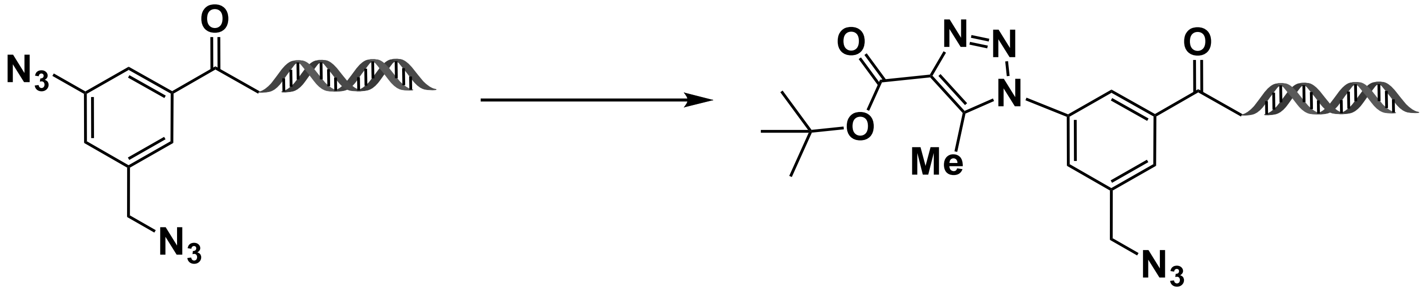

calcd.: 5277.0

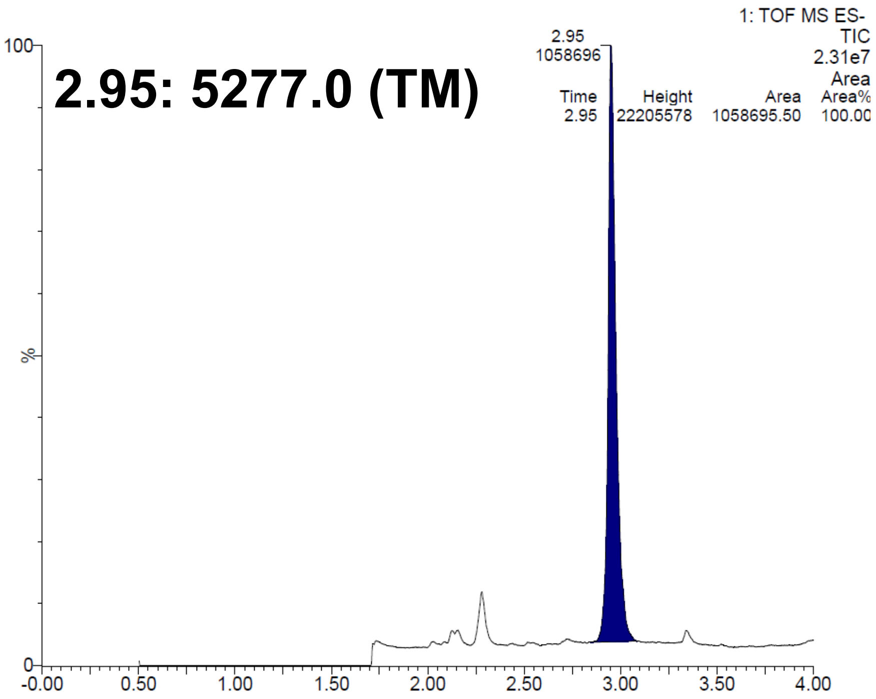

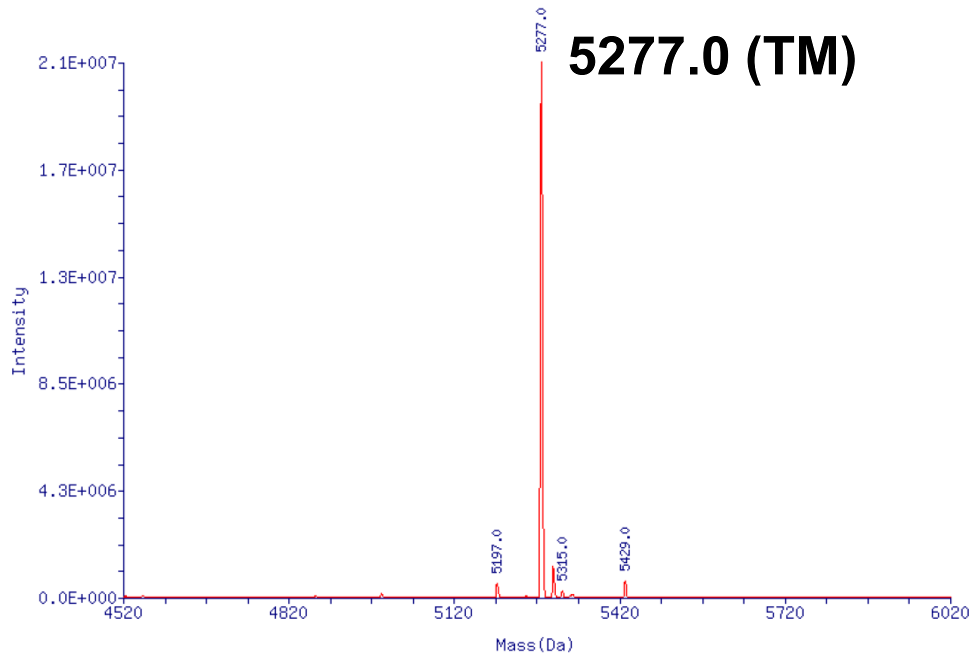

6g’

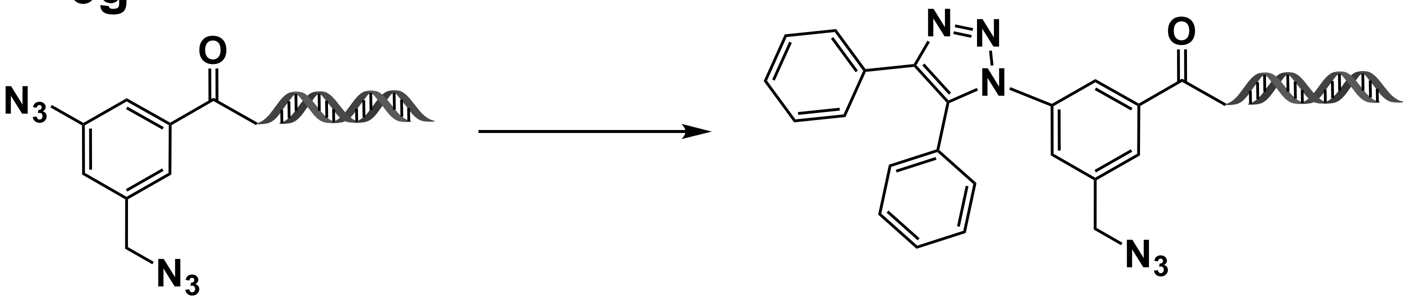

calcd.: 5315.5

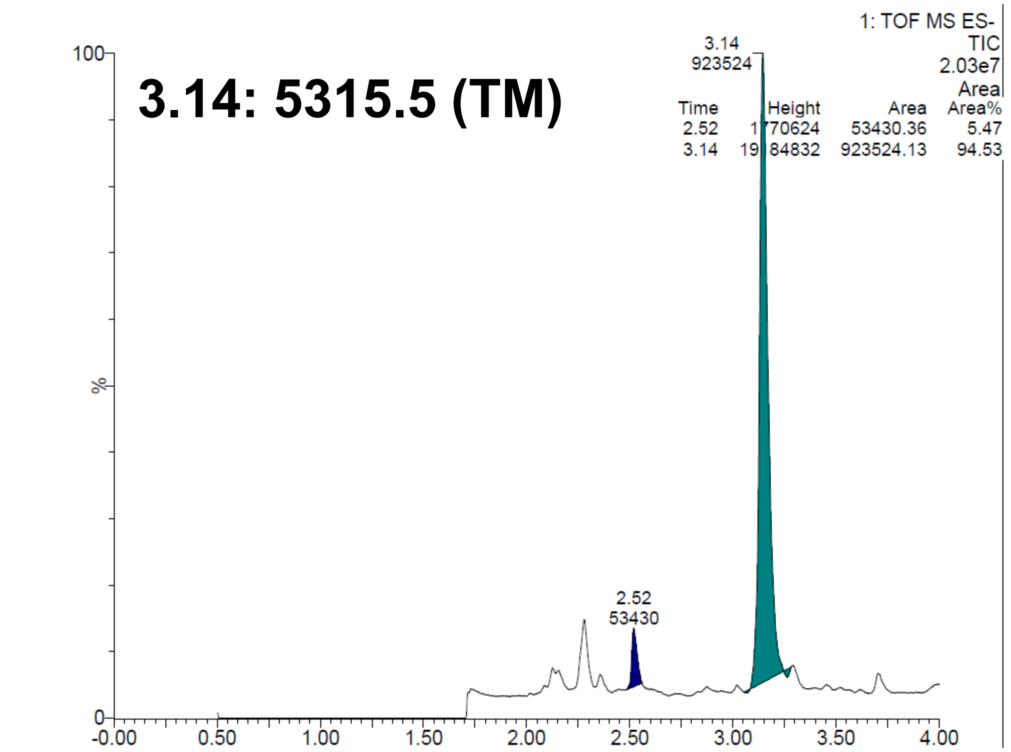

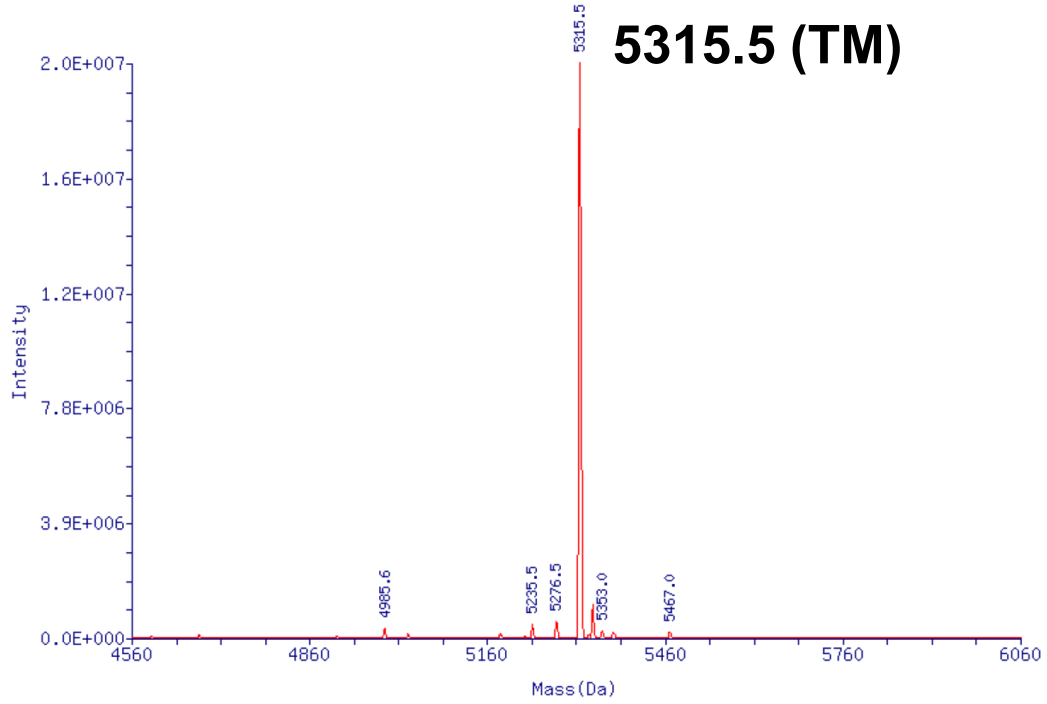

6h’

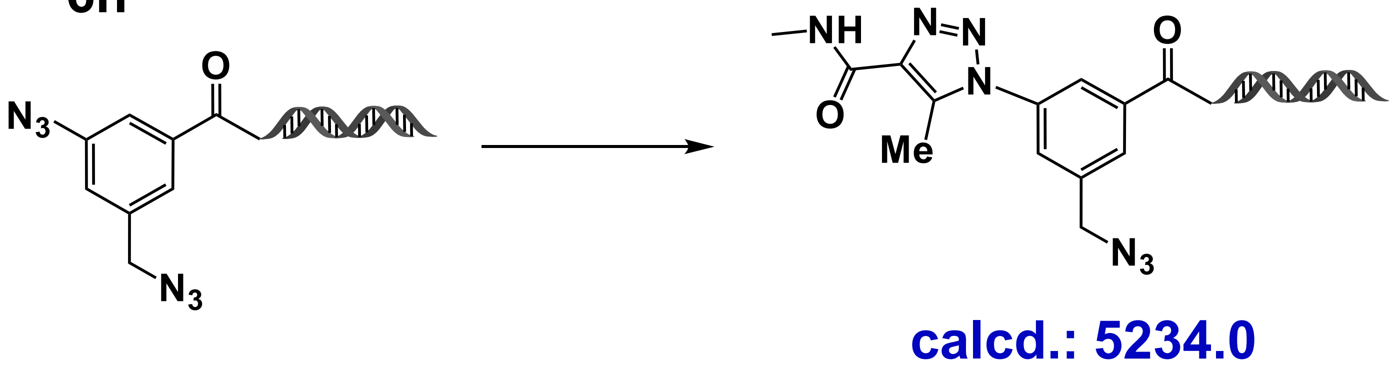

N3
O
N3
NH
N=N
N
O
Me
O
N3
calcd.: 5234.0

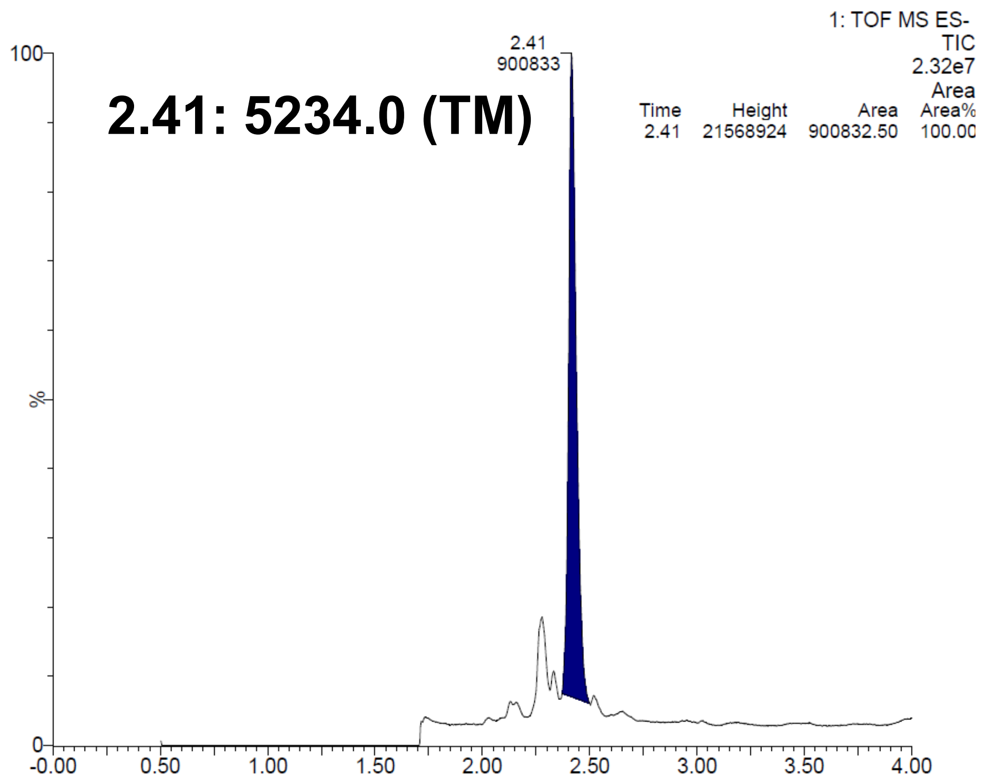

1: TOF MS ES-
TIC
2.32e7
Area
Time
Height
Area
Area%
2.41
21568924
900832.50
100.00
2.41
900833
2.41: 5234.0 (TM)
100
%
0
-0.00
0.50
1.00
1.50
2.00
2.50
3.00
3.50
4.00

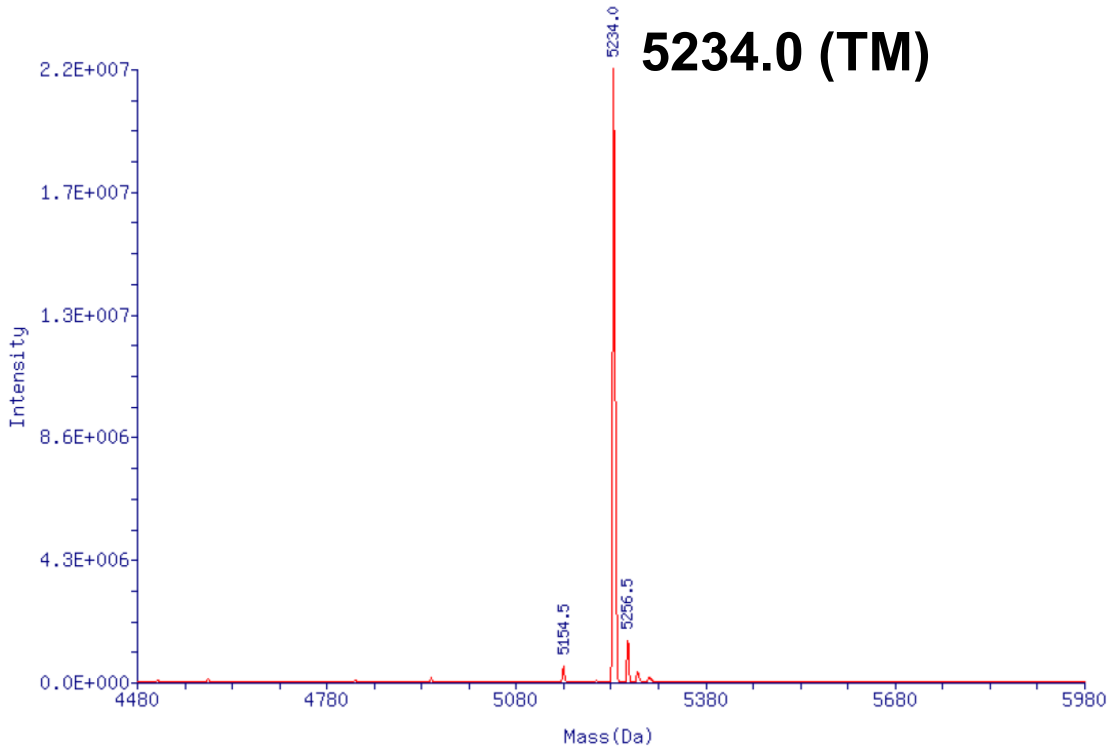

5234.0
5234.0 (TM)
2.2E+007
1.7E+007
1.3E+007
8.6E+006
4.3E+006
0.0E+000
Intensity
5154.5
5256.5
4480
4780
5080
5380
5680
5980
Mass(Da)

6i'

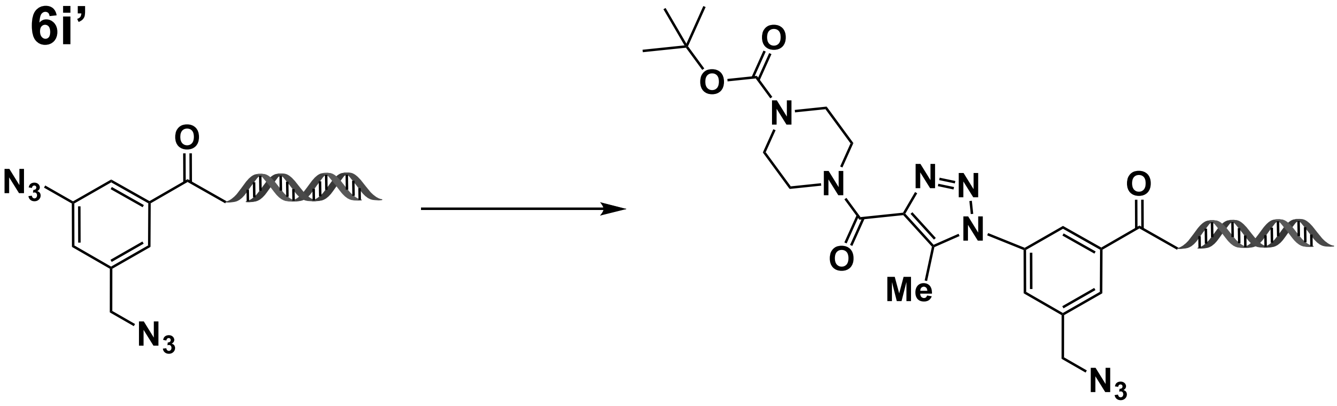

calcd.: 5389.5

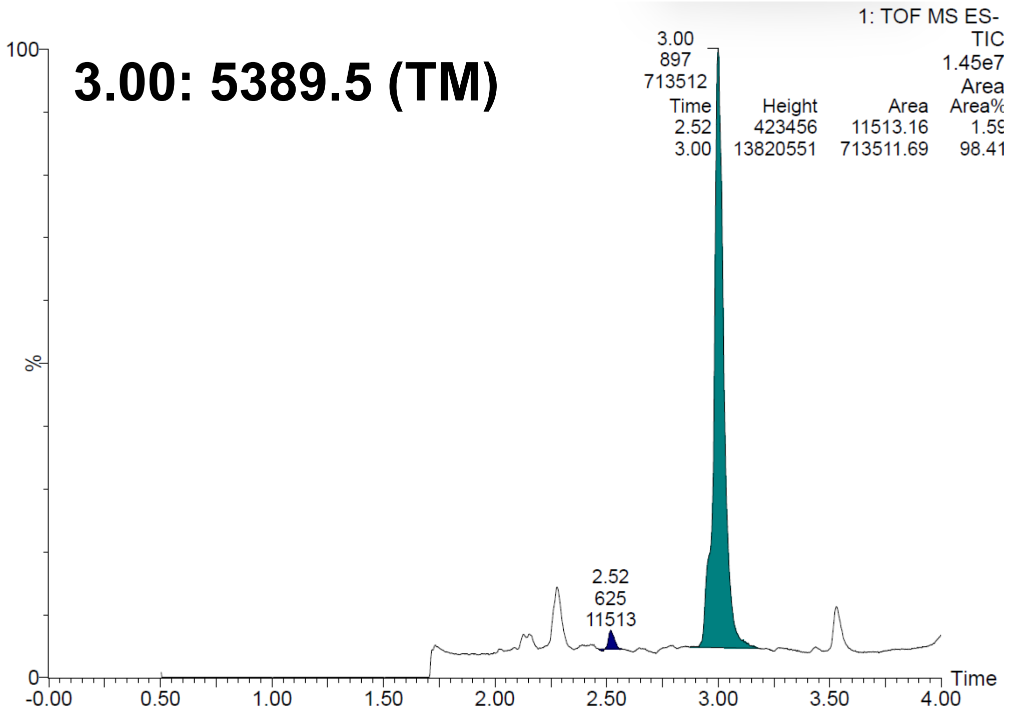

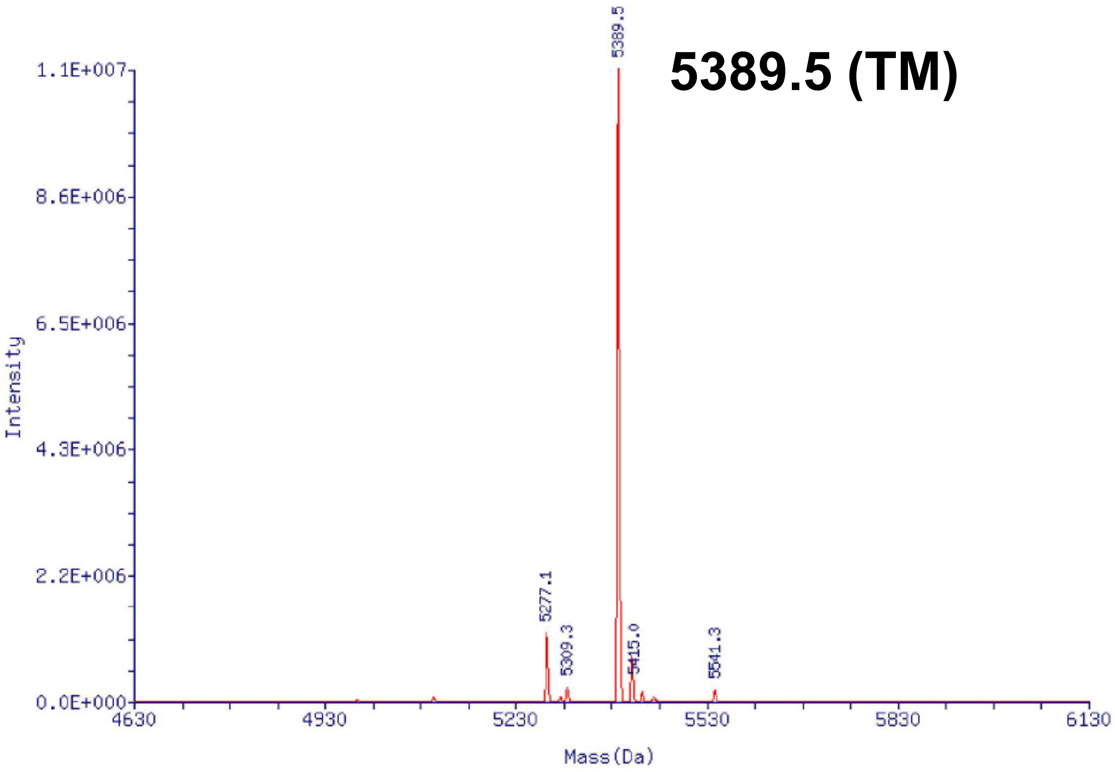

6j'

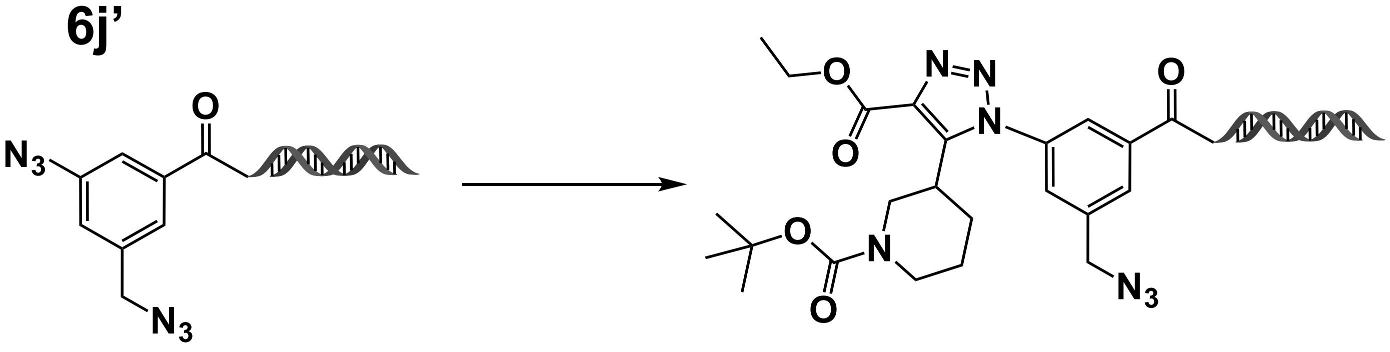

calcd.: 5418.3

2.72: 5418.3 (TM)

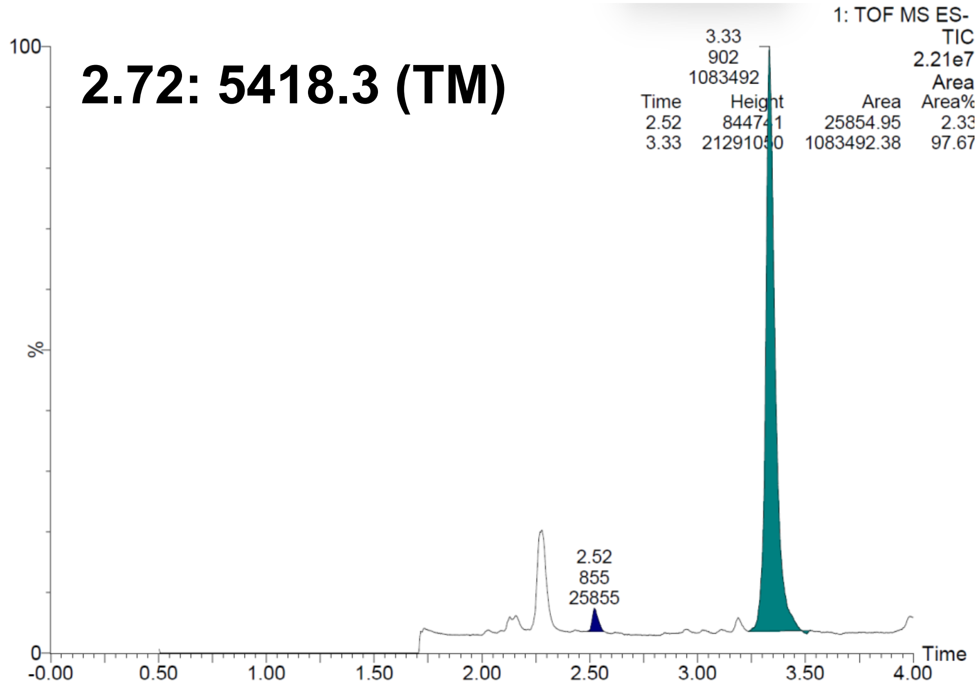

5418.3 (TM)

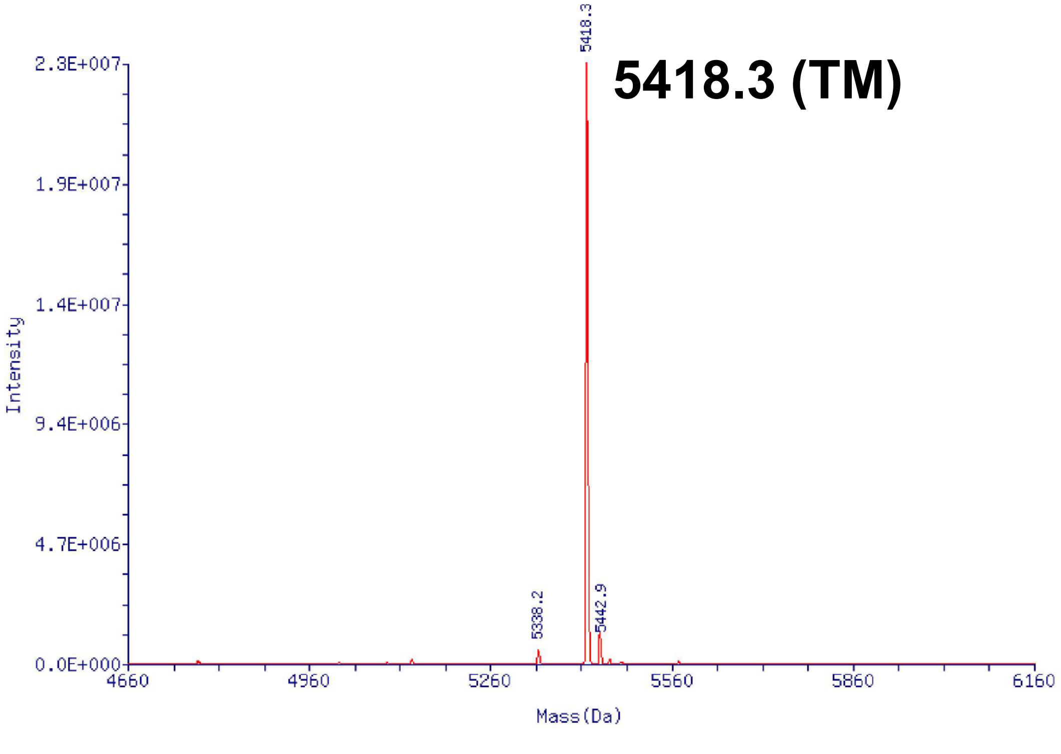

6k'

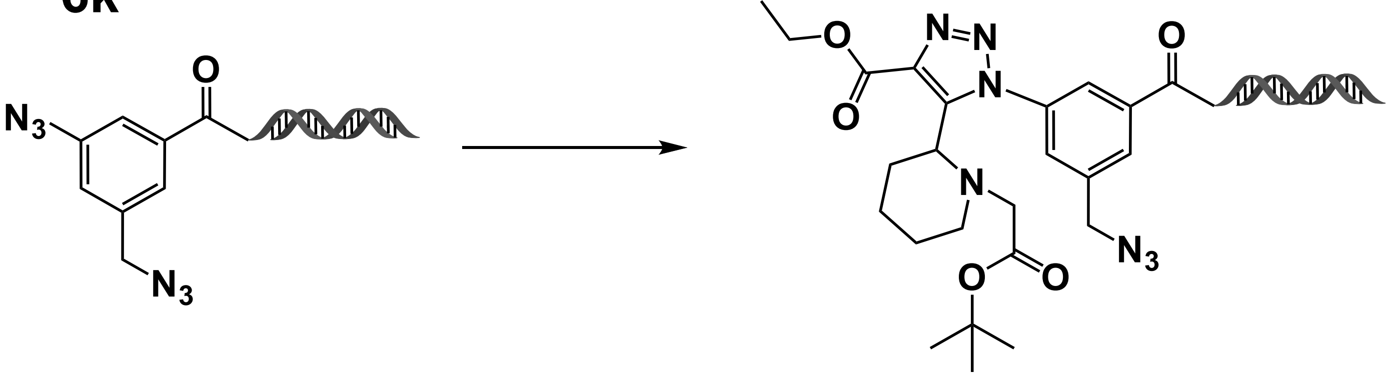

calcd.: 5418.4

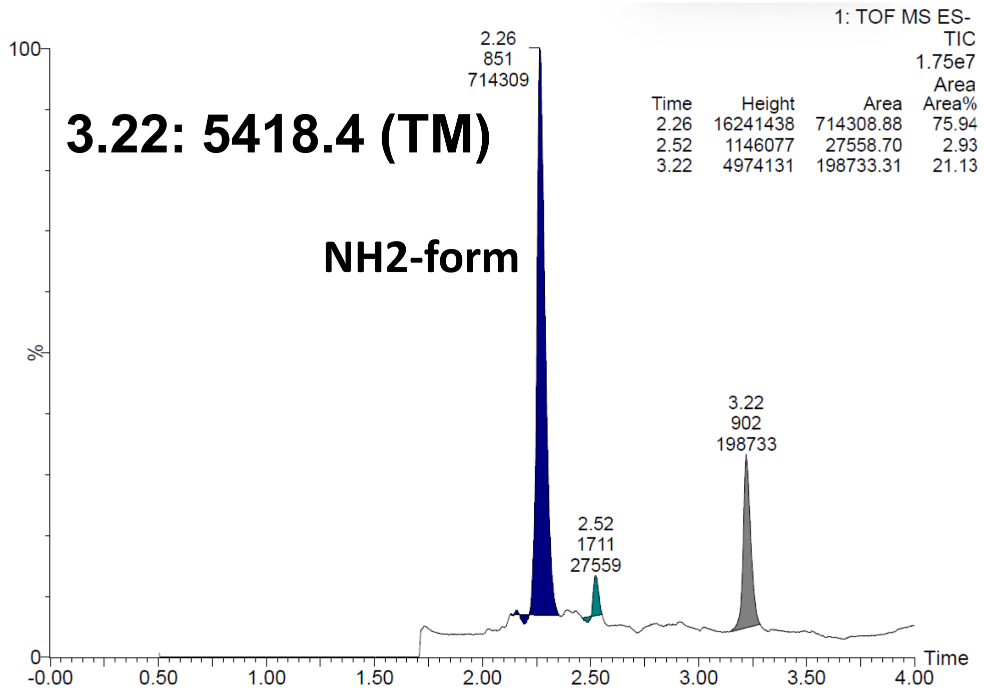

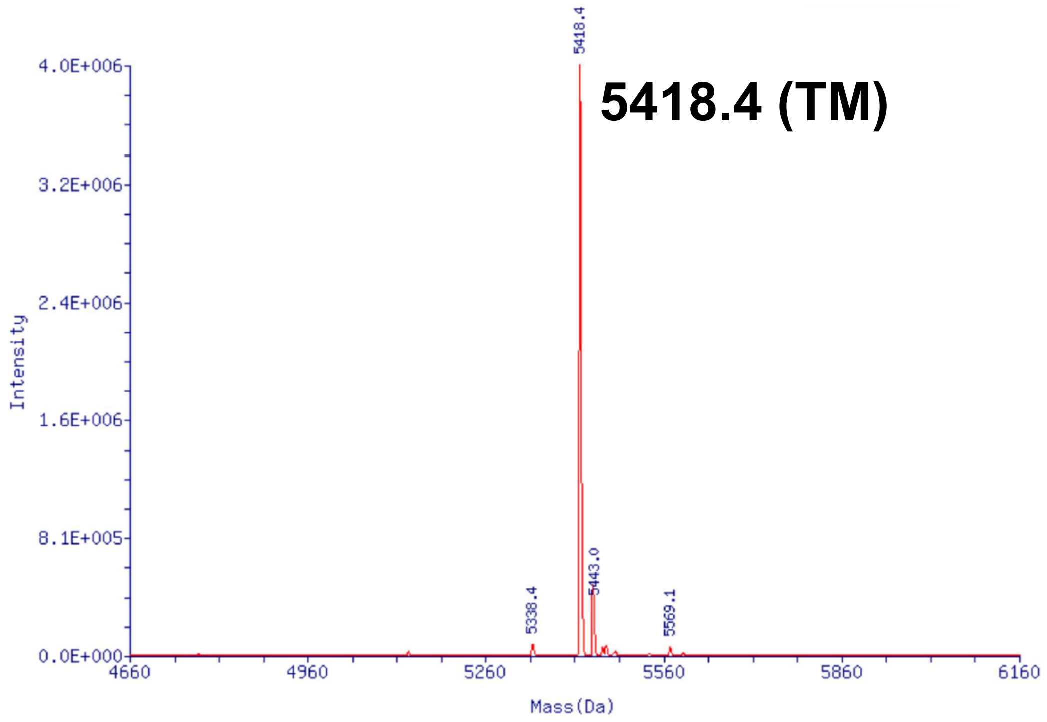

6l'

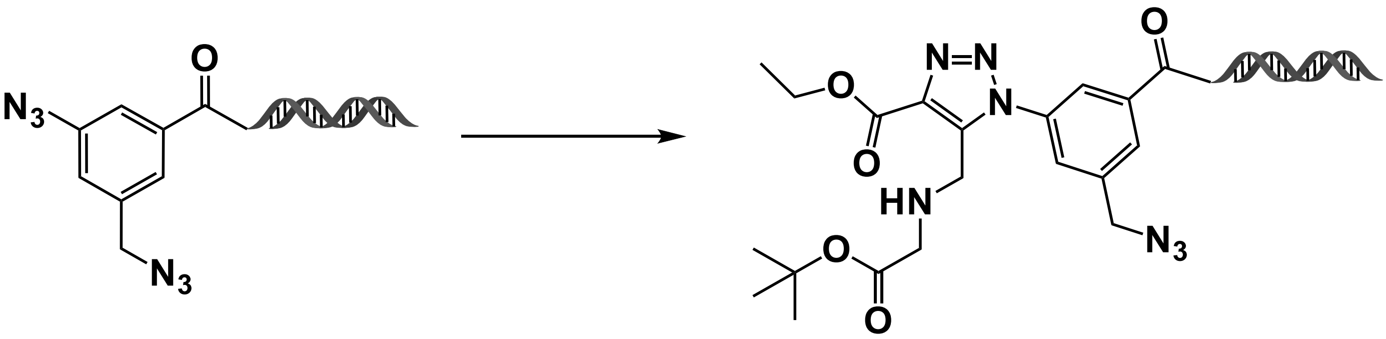

calcd.: 5364.2

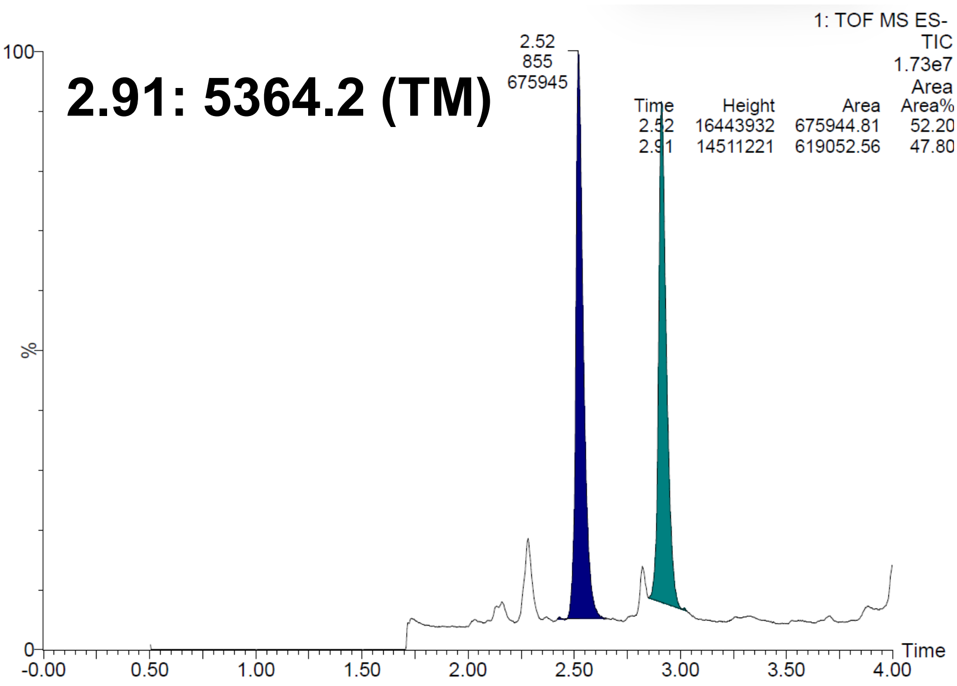

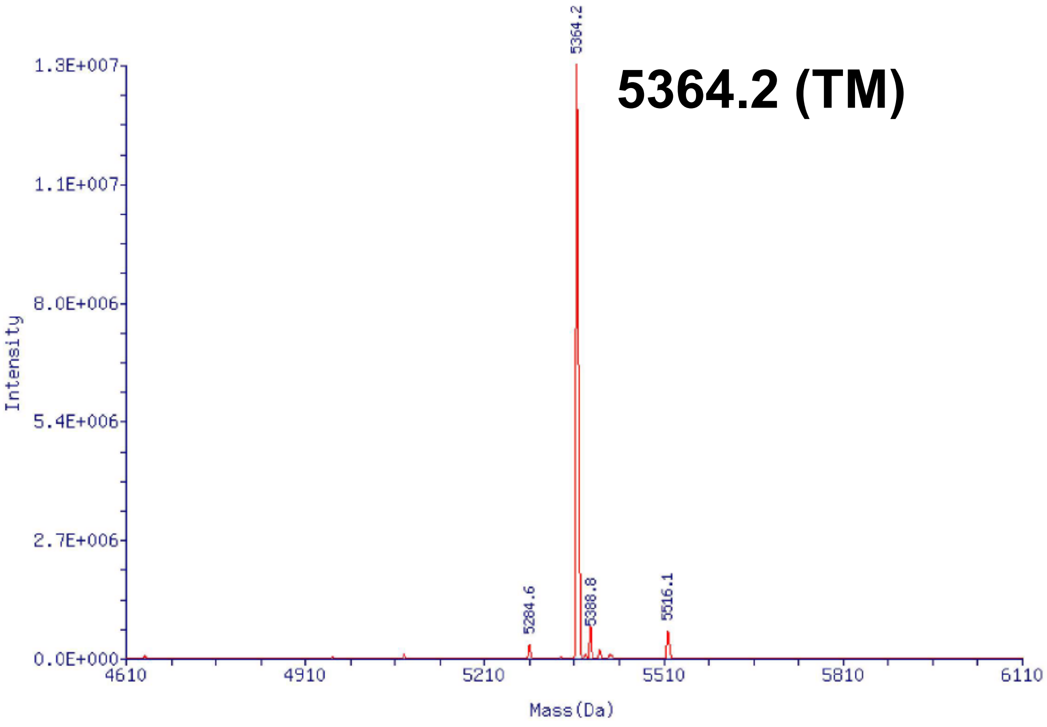

6m’

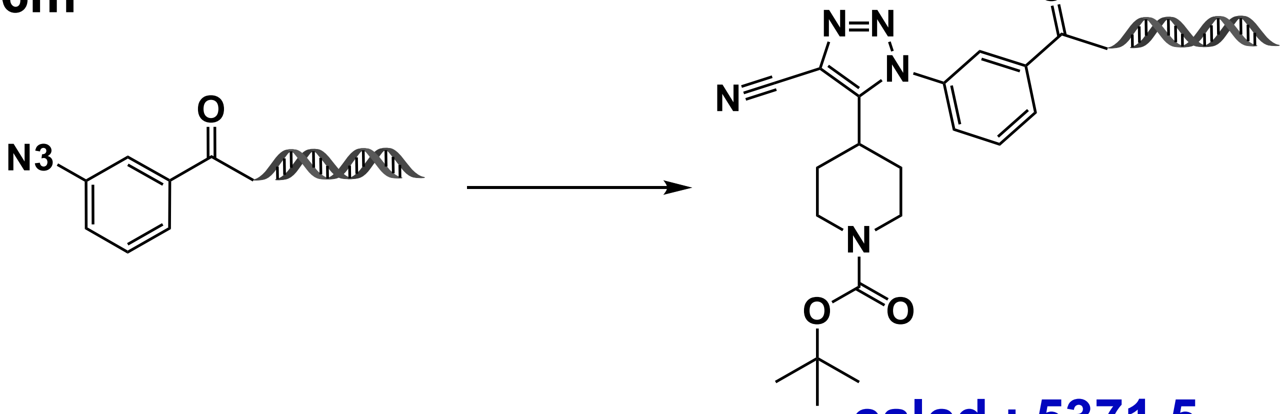

calcd.: 5371.5

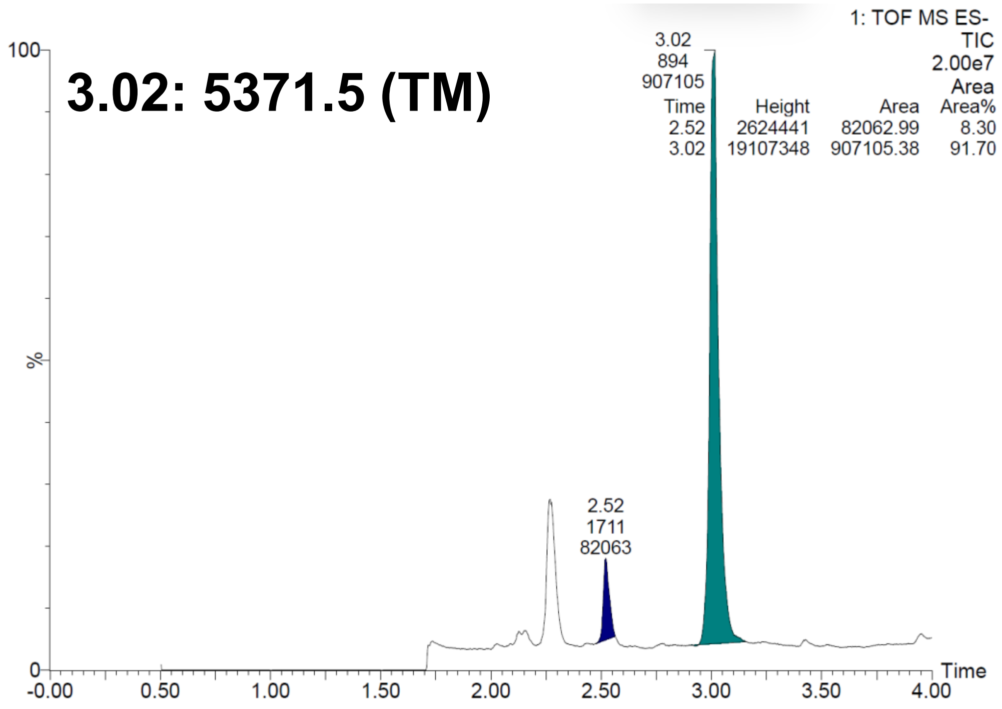

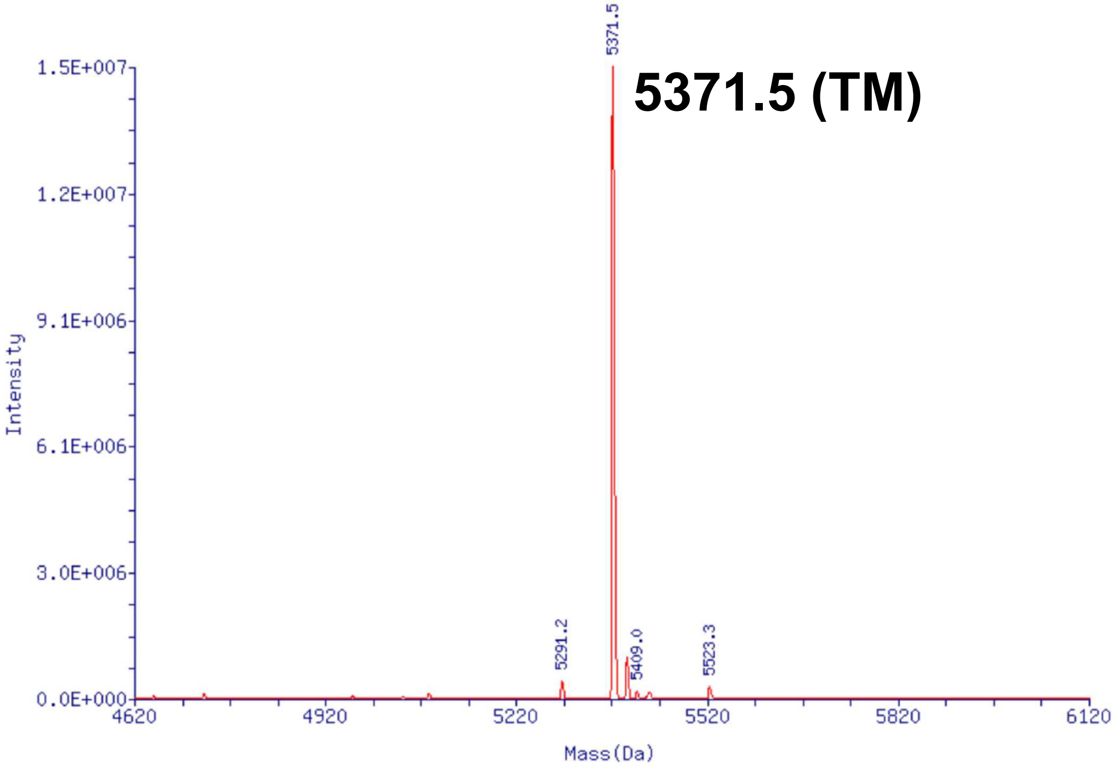

6n’

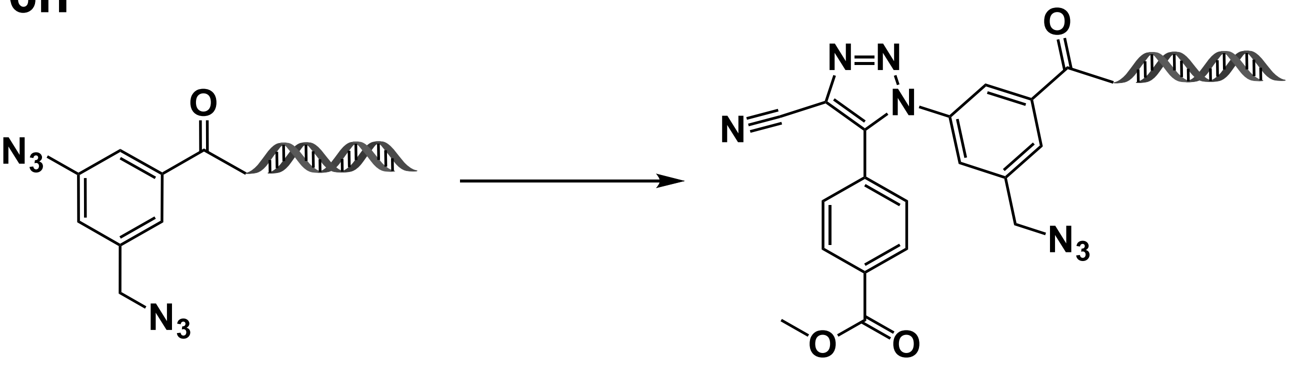

calcd.: 5322.1

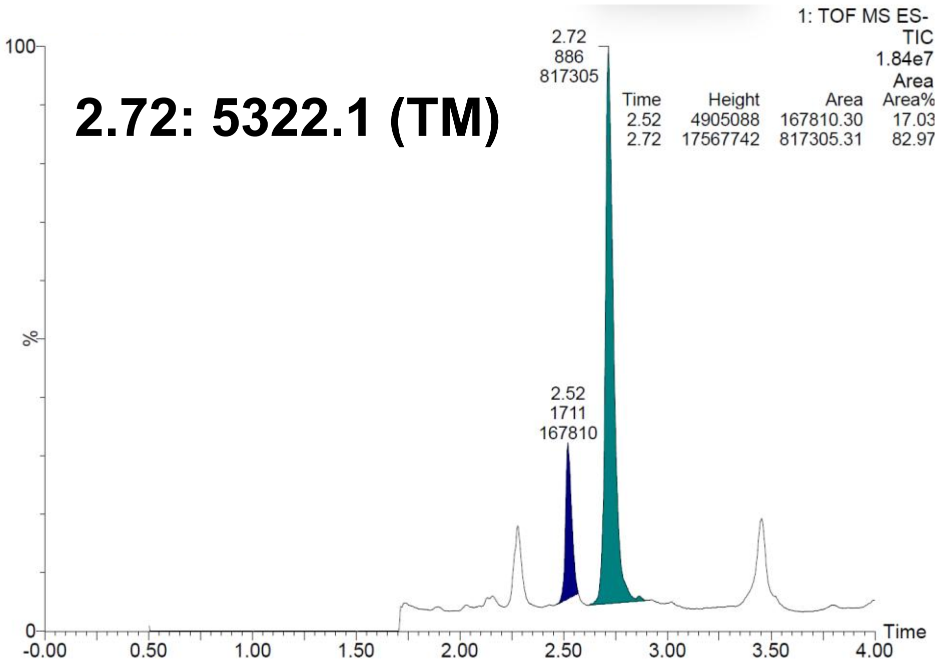

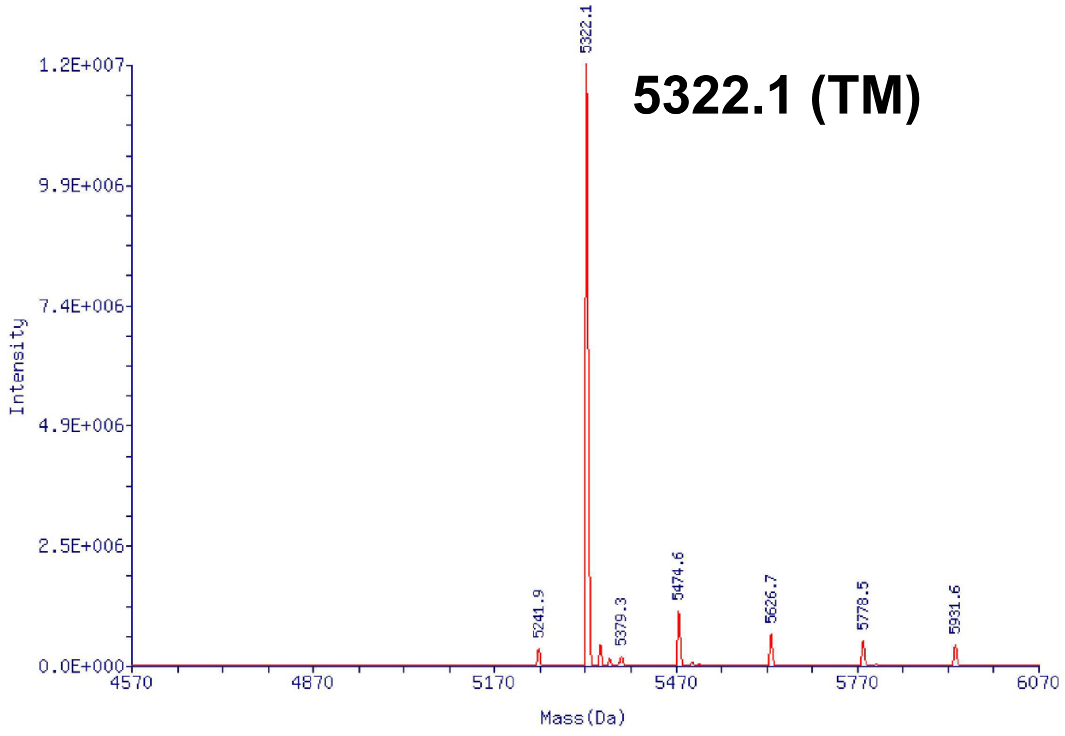

2.2 Stepwise Synthesis of DEL Warheads Using C-D-DAP (1): On-DNA Diazido Platform

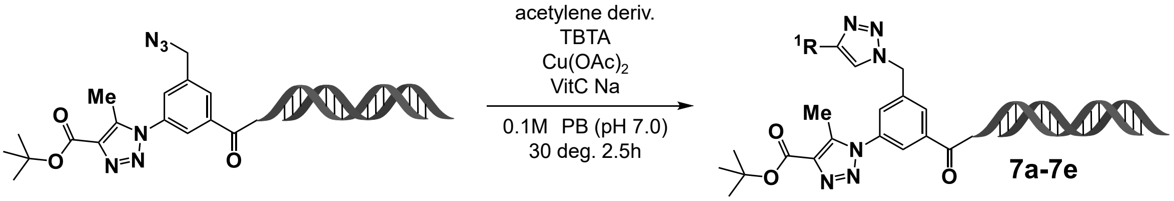

No.	acethylene derivative	R	conversion (%)	MS. (calcd/found)
7a	4-methylphenylacetylene	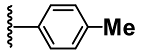	quant.	5393.0 / 5392.9
7b	4-carboxyphenylacetylene	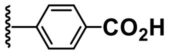	quant.	5423.0 / 5423.1
7c	2-ethynylpyridine	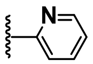	quant.	5380.0 / 5380.0
7d	4-methoxyphenylacetylene	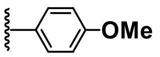	98.5	5409.0 / 5409.1
7e	cyclopropylacetylene	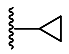	91.4	5343.0 / 5343.1

7a

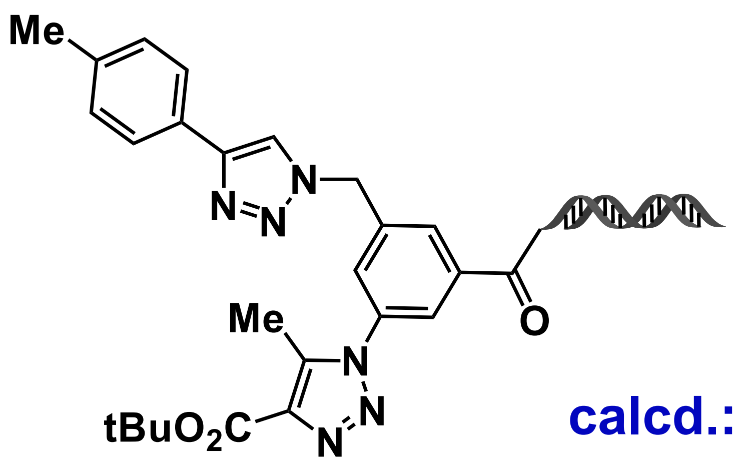

calcd.: 5393.0

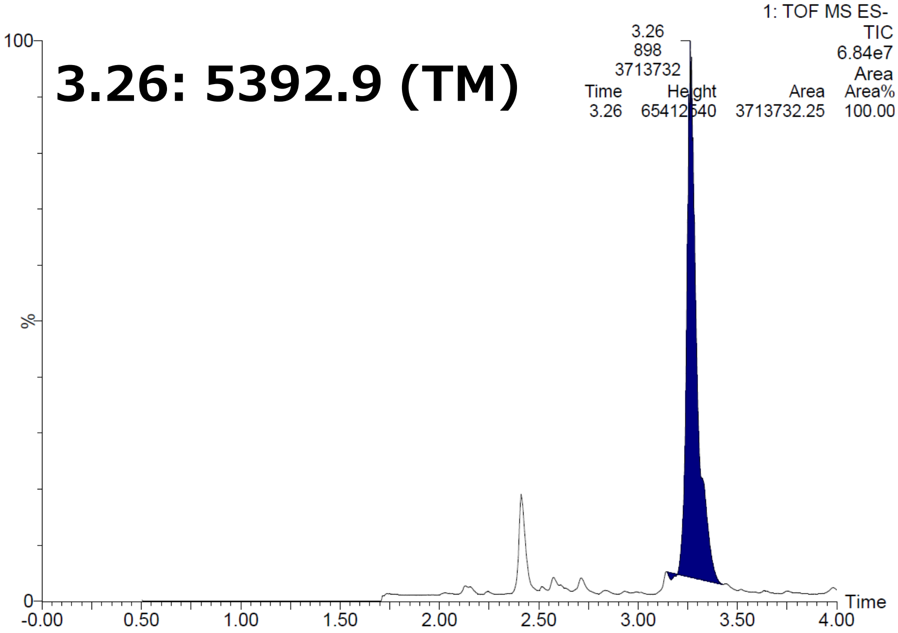

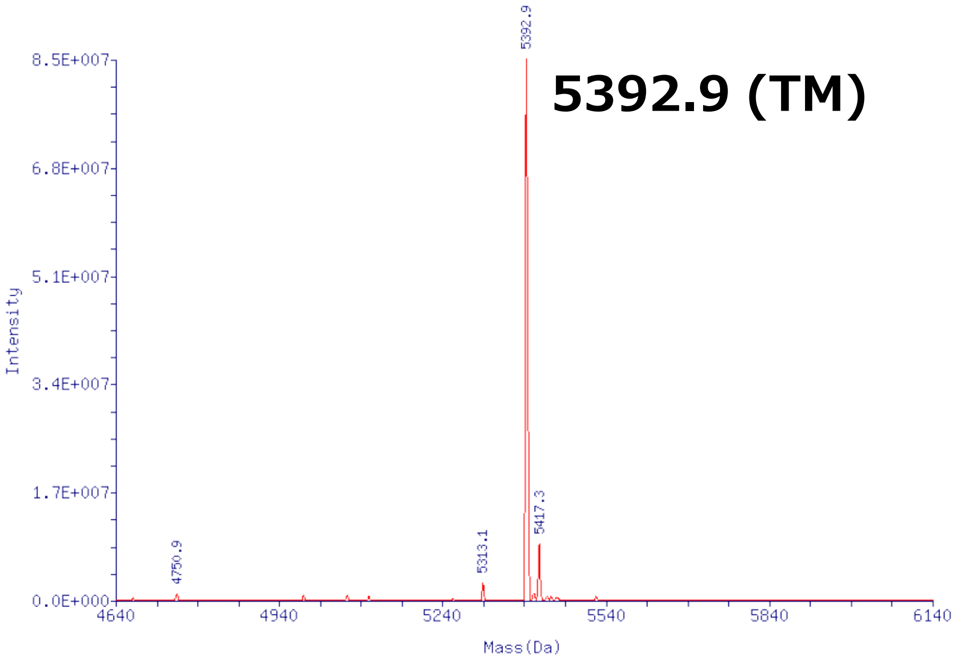

7b

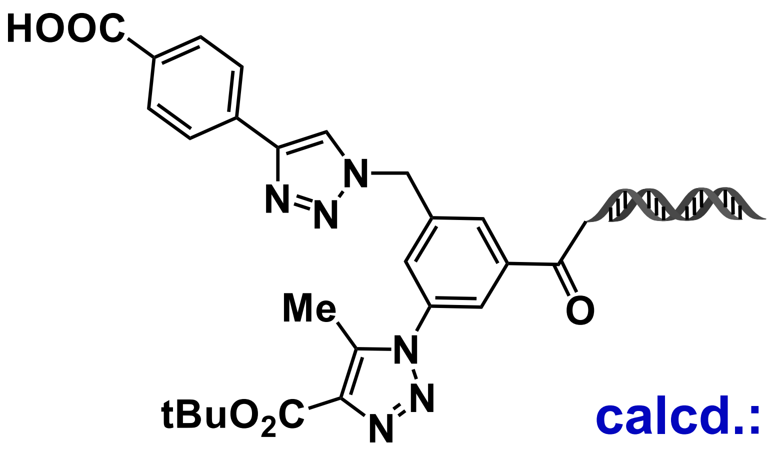

calcd.: 5423.0

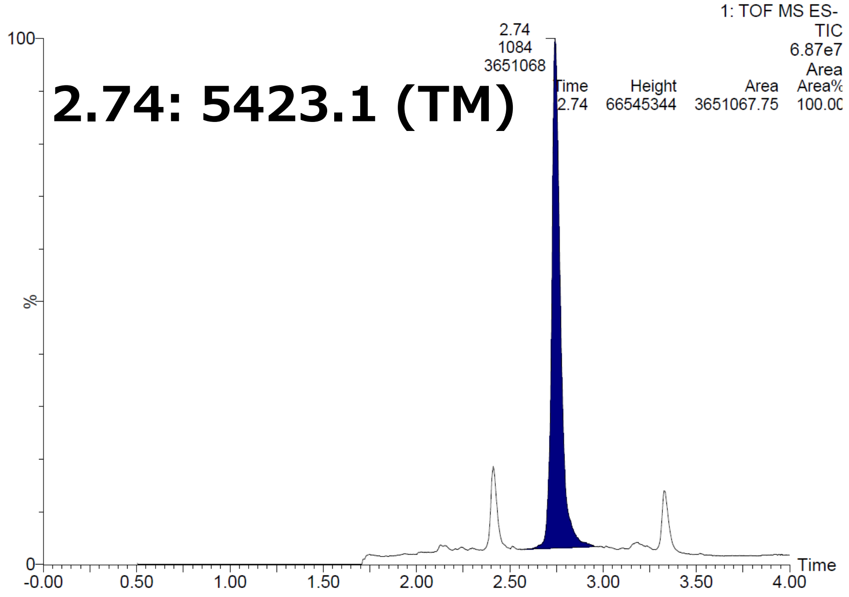

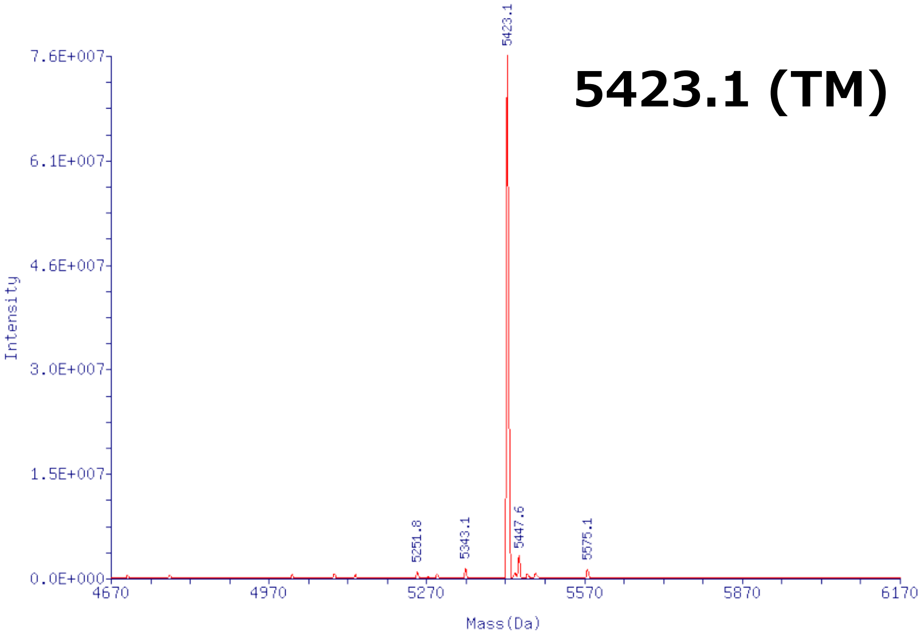

7c

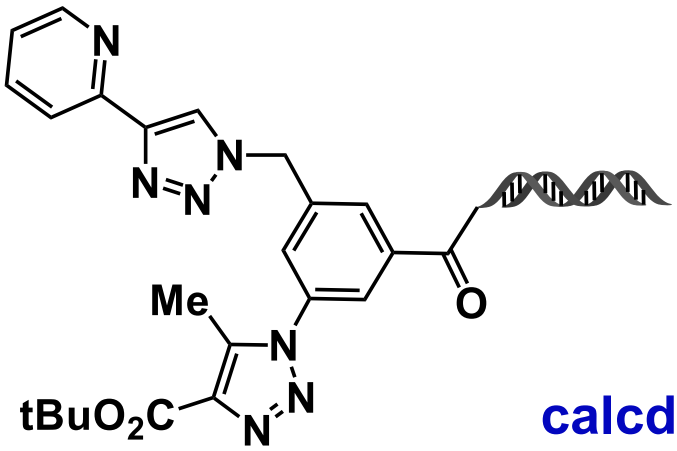

calcd.: 5380.0

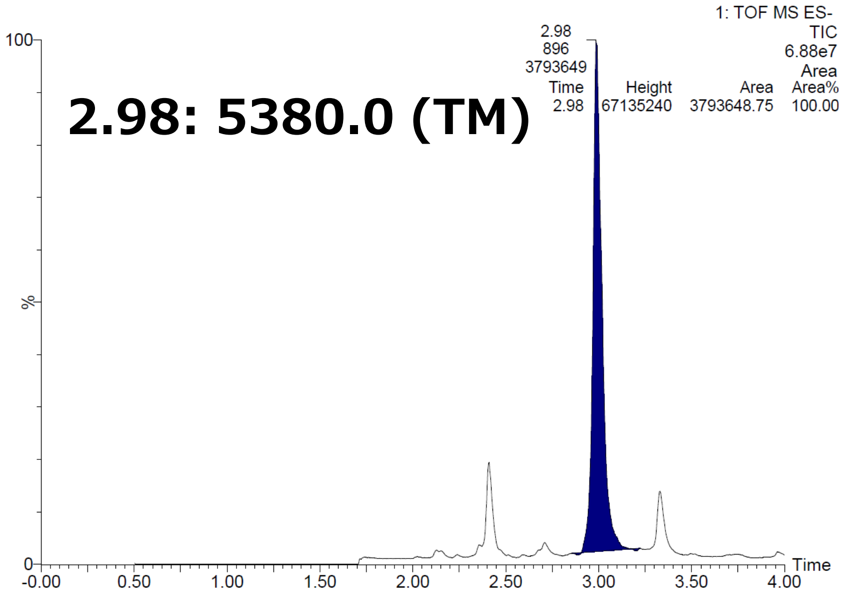

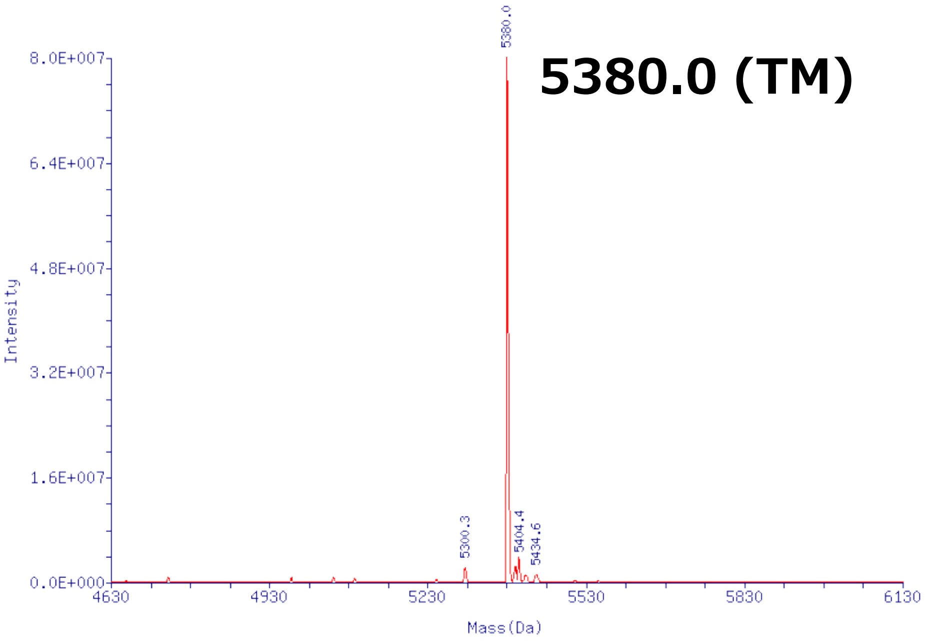

7d

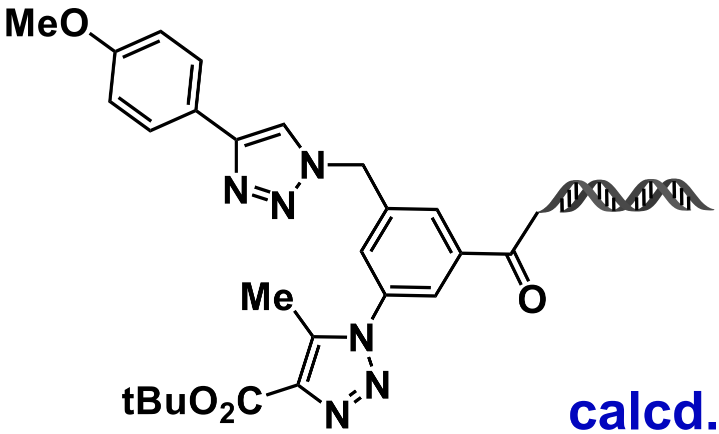

calcd.: 5409.0

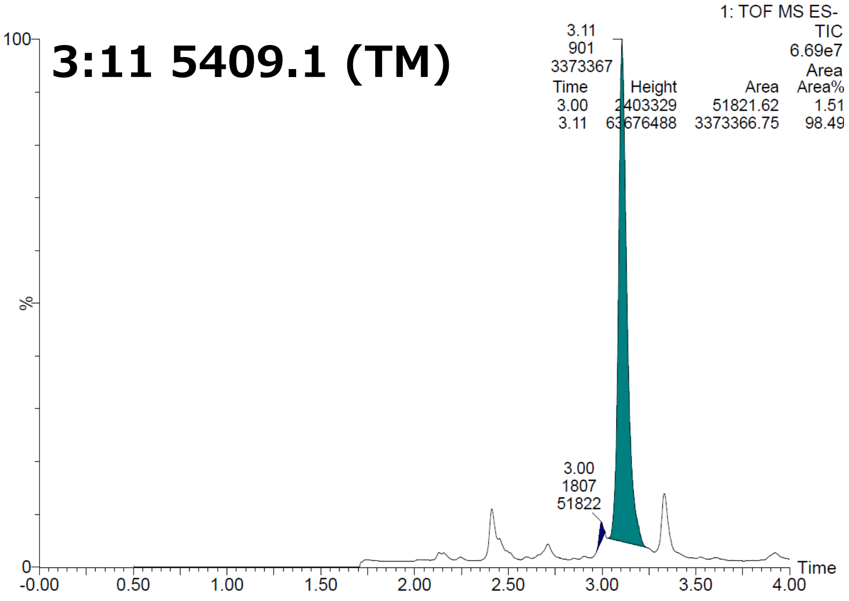

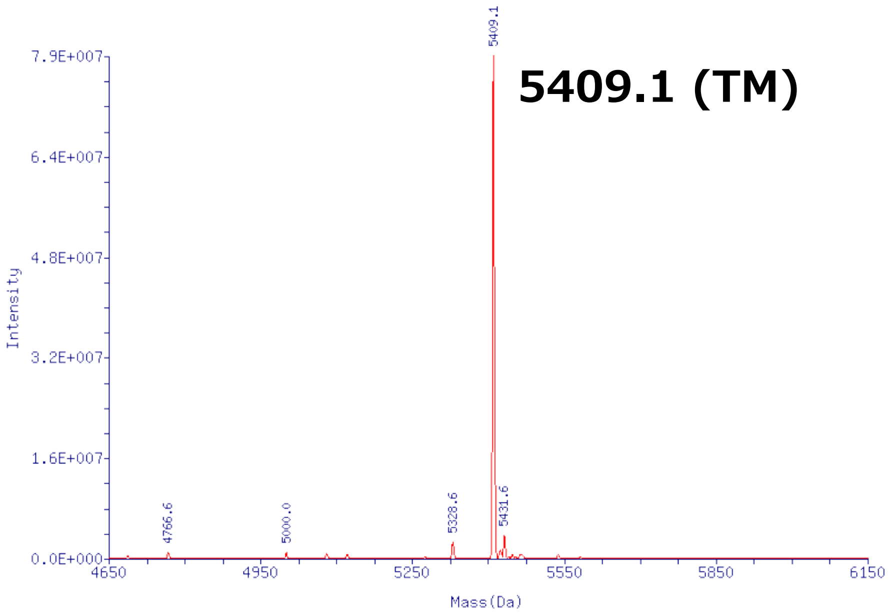

7e

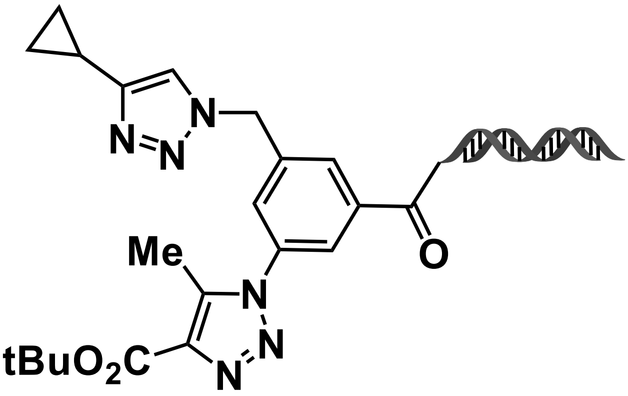

calcd.: 5343.0

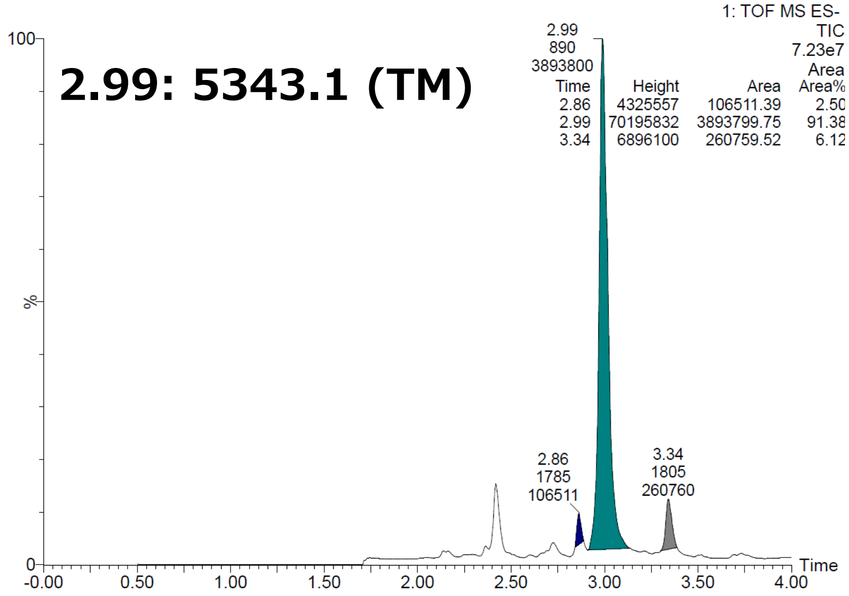

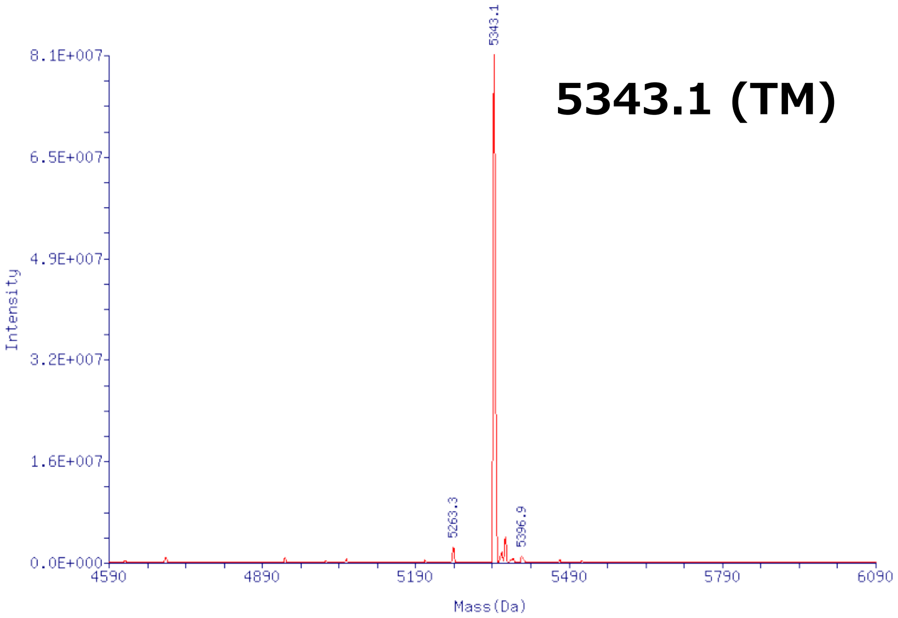

2.3. DEL Synthesis Using C-D-DAP (2): On-DNA Azido–Amino Platform

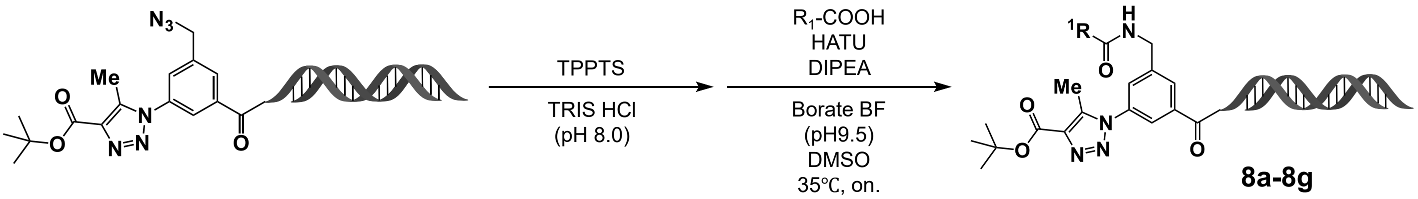

No.	capping cpd.	R¹	conversion (%)	MS. (calcd/found)
8a	3-MeO-benzoic acid	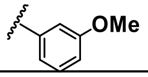	70.8	5385.0 / 5385.1
8b	4-Cl-benzoic acid	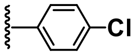	67.1	5389.0 / 5389.7
8c	cyclopropanecarboxylic acid	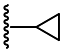	68.5	5319.0 / 5319.2
8d	cyclobutanecarboxylic acid	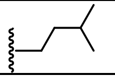	69.3	5349.1 / 5349.2
8e	4-isonicotinic acid	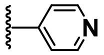	71.2	5356.0 / 5356.1
8f	crotonic acid	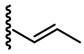	74.2	5319.0 / 5319.4
8g	phenylpropiolic acid	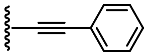	68.7	5379.0 / 5379.1

8a

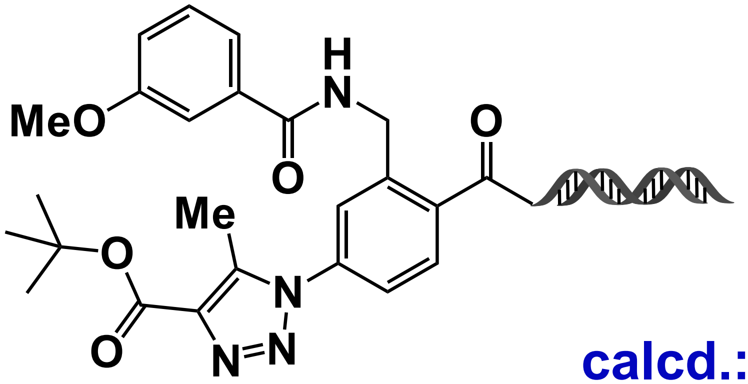

calcd.: 5385.0

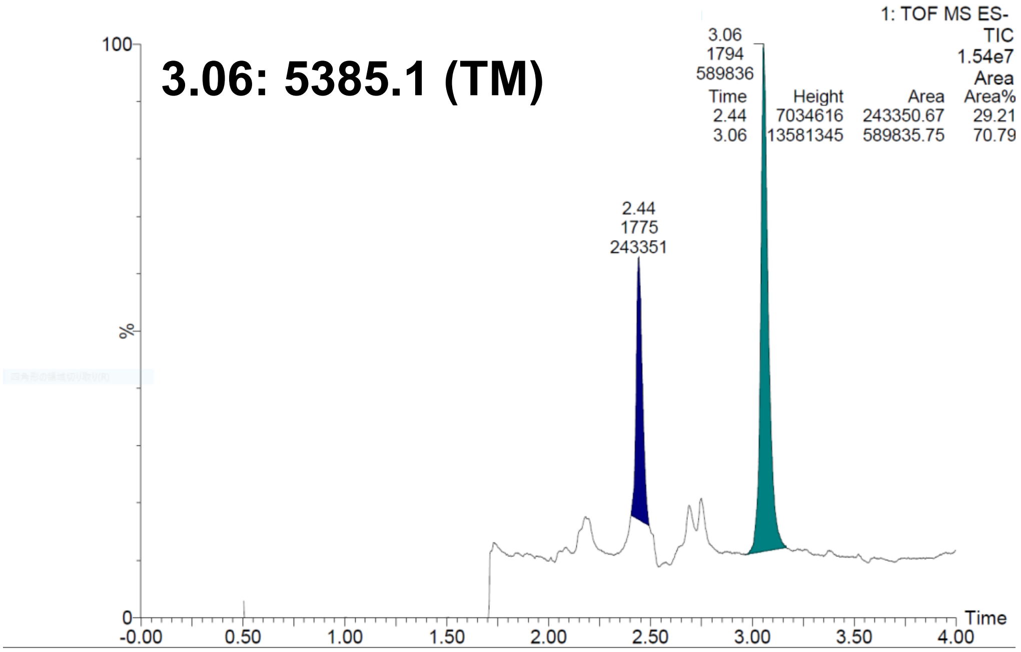

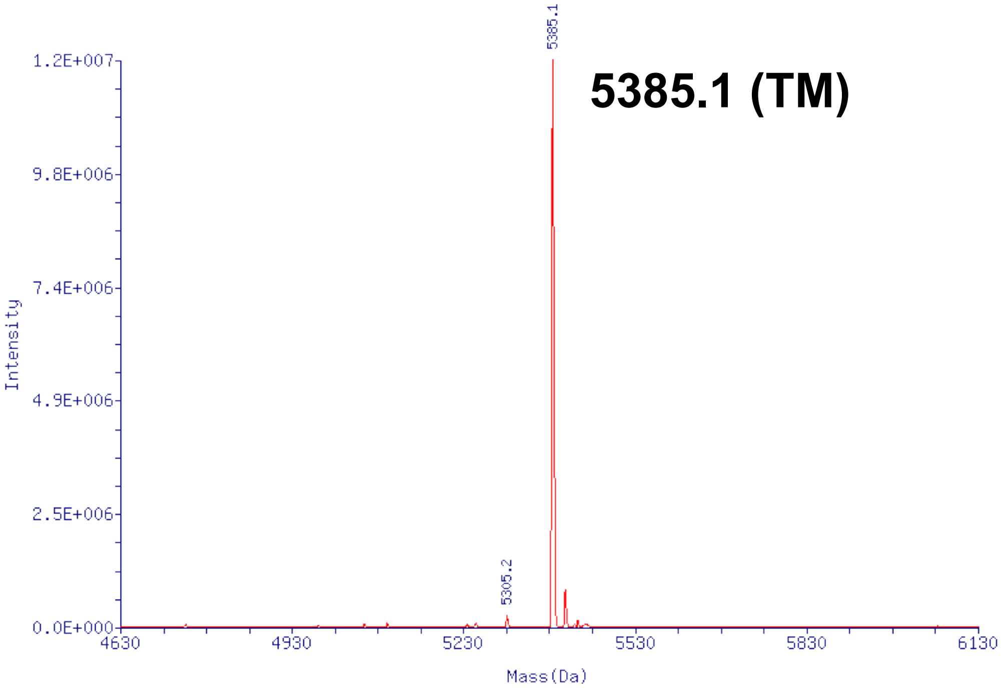

8b

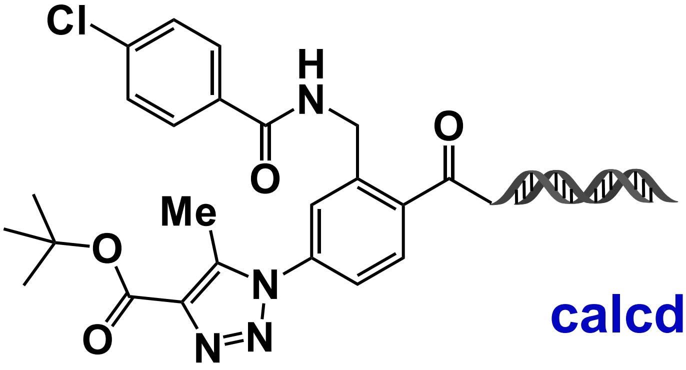

calcd.: 5389.0

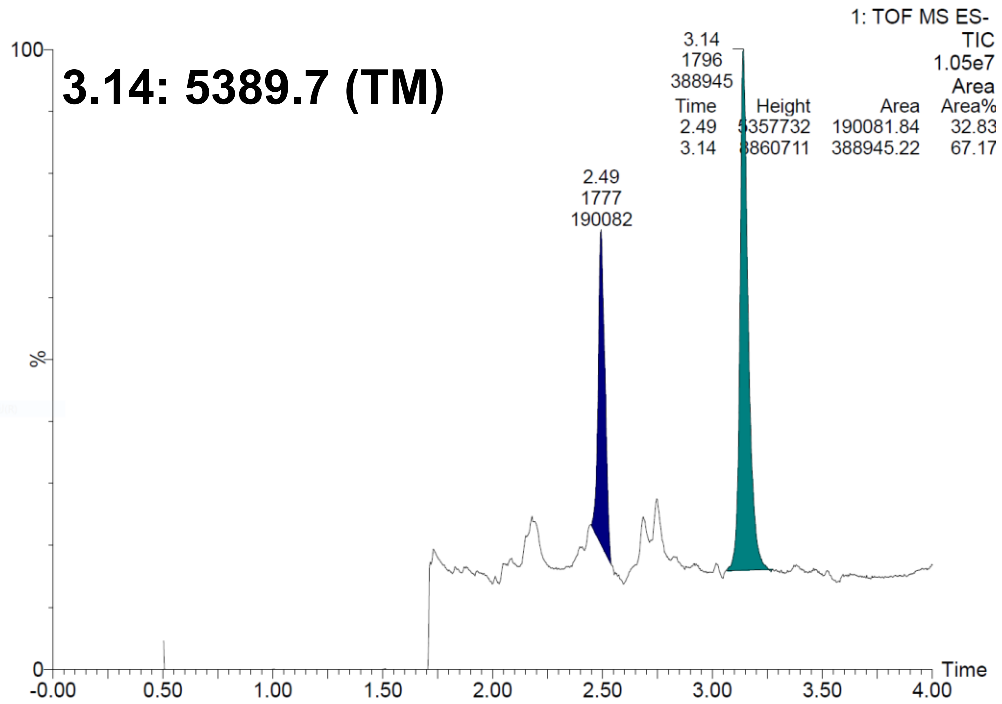

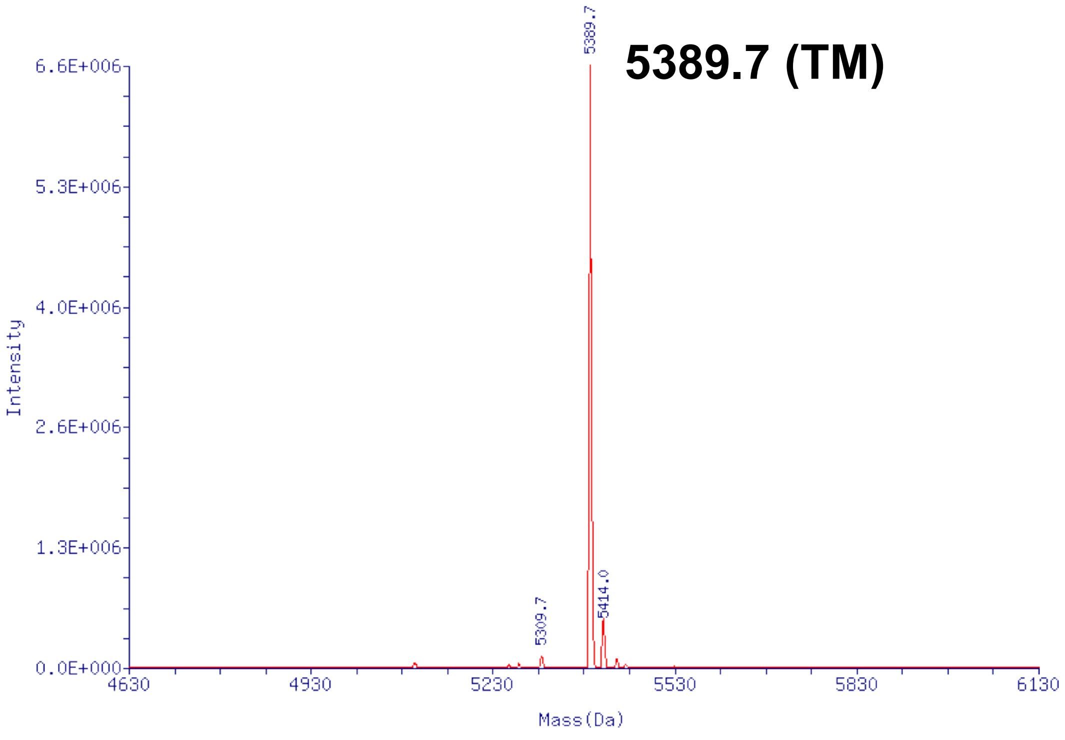

8c

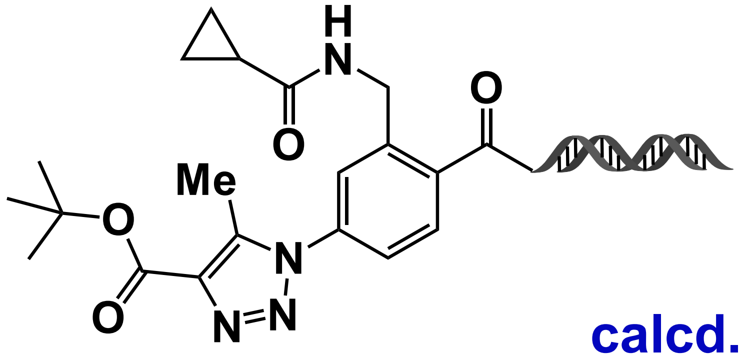

calcd.: 5319.0

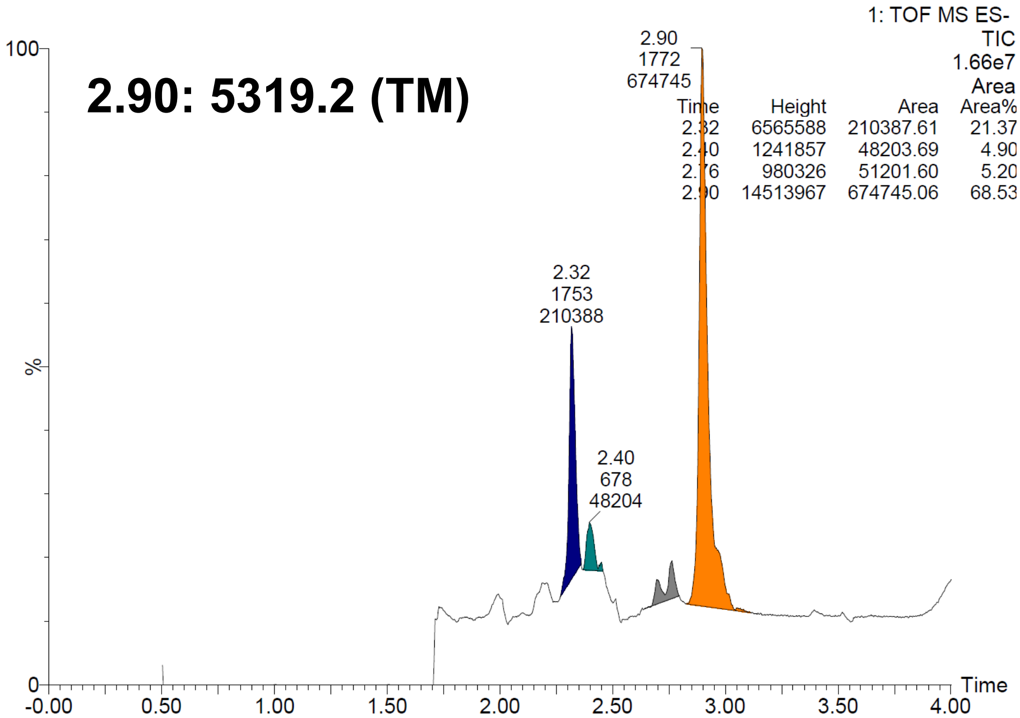

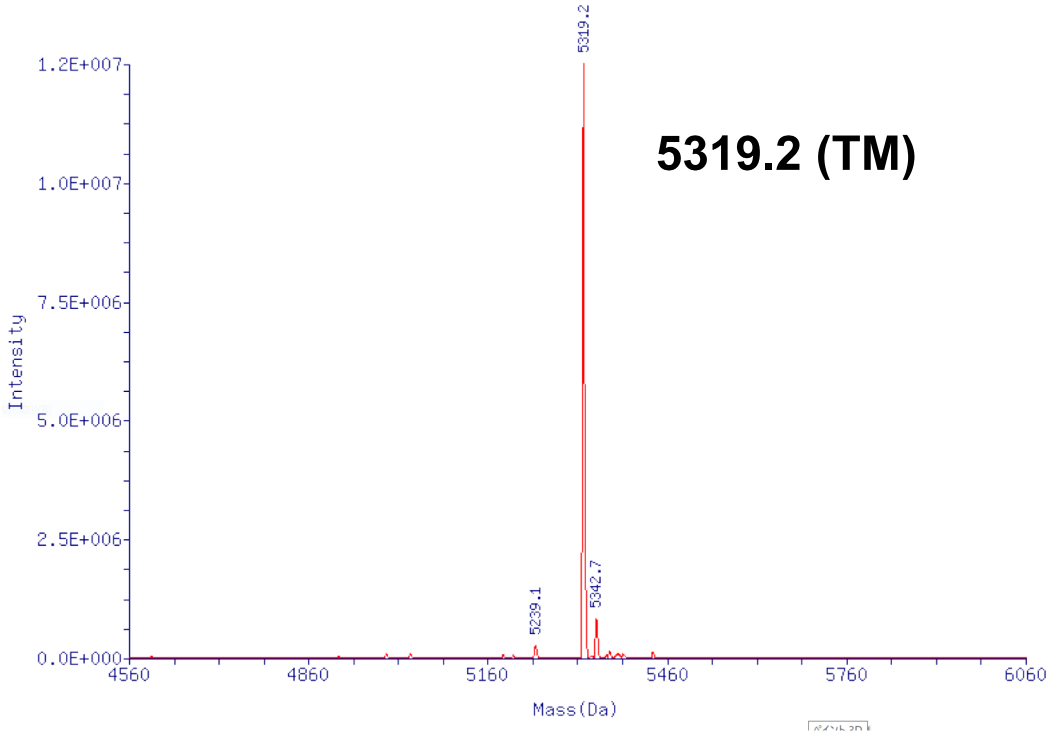

8d

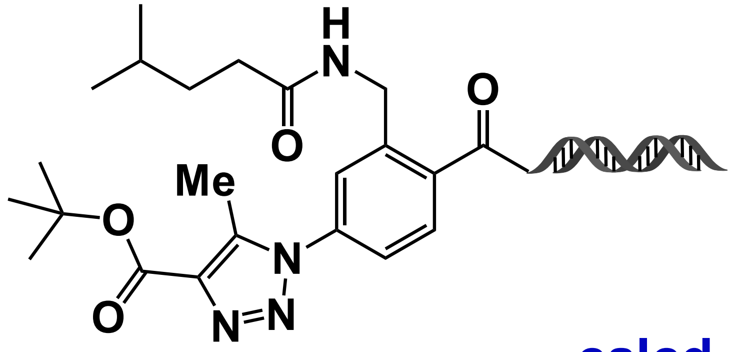

calcd.: 5349.1

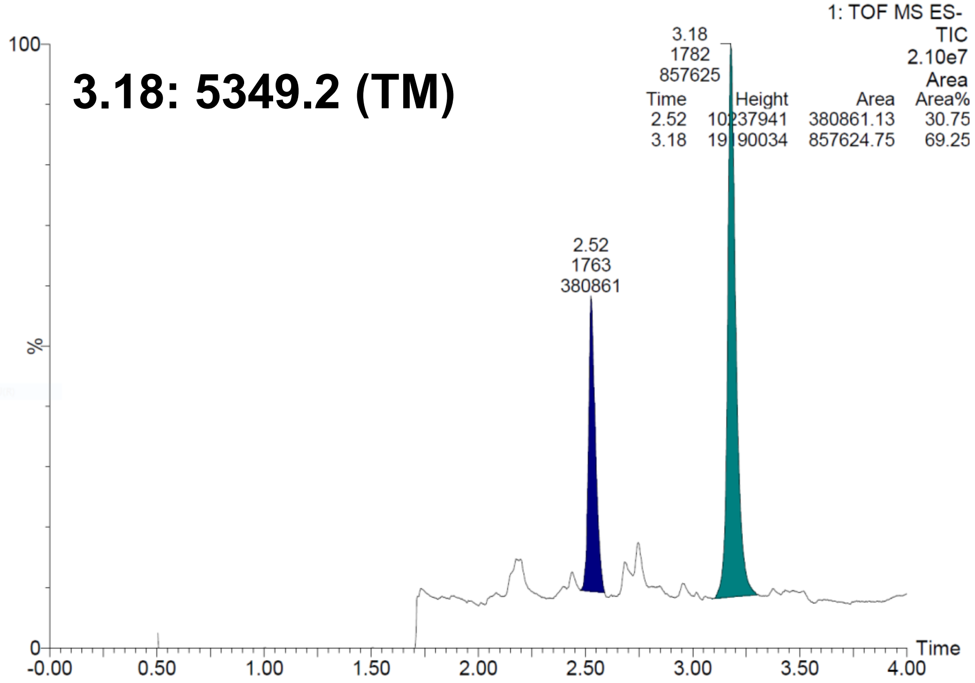

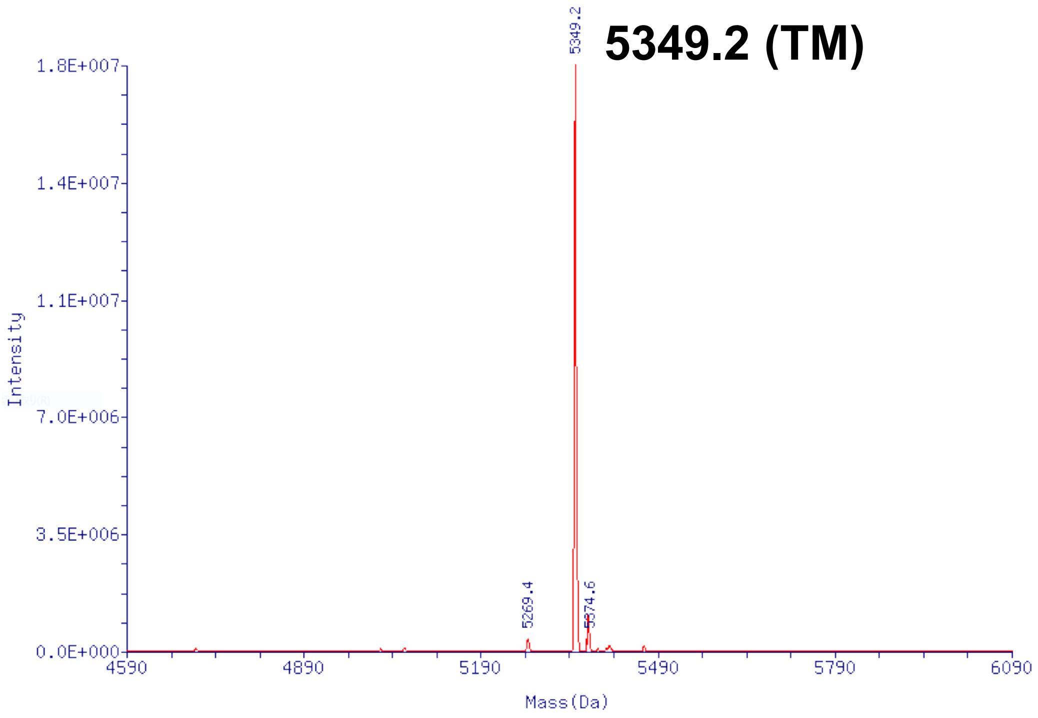

8e

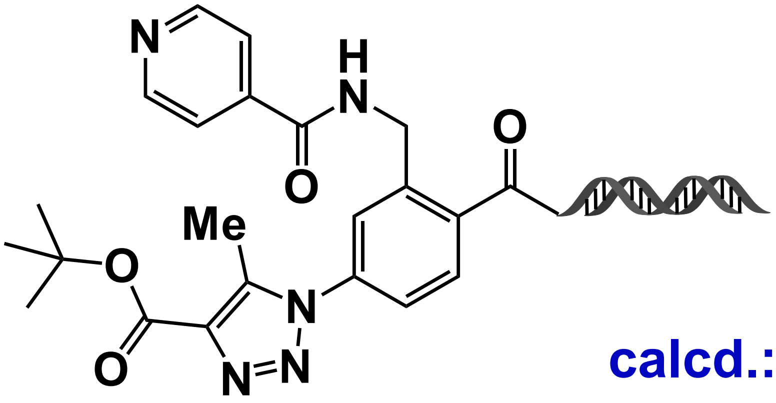

calcd.: 5356.0

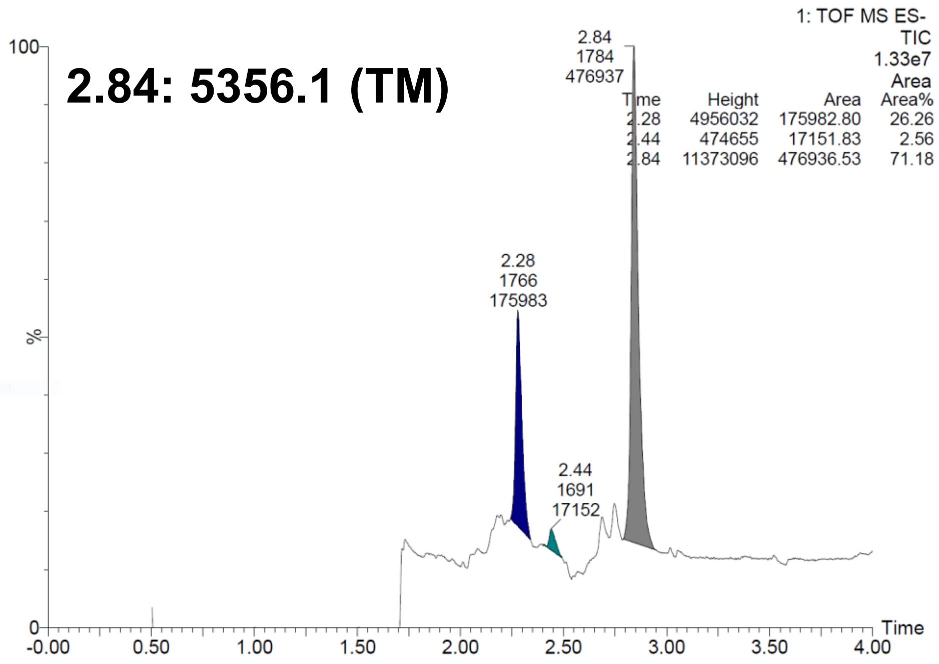

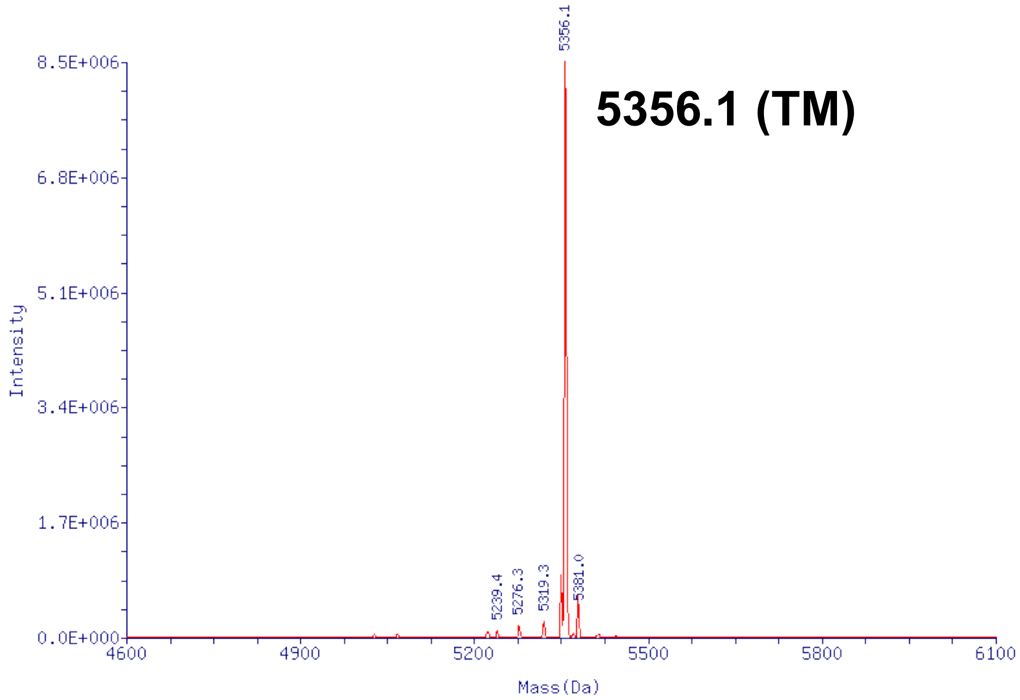

8f

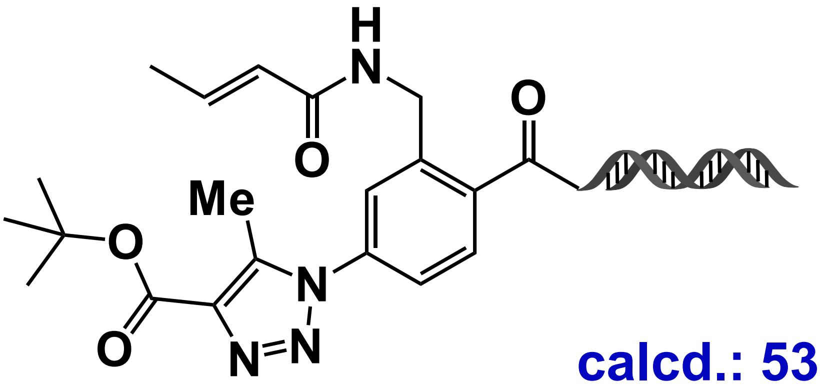

calcd.: 5319.0

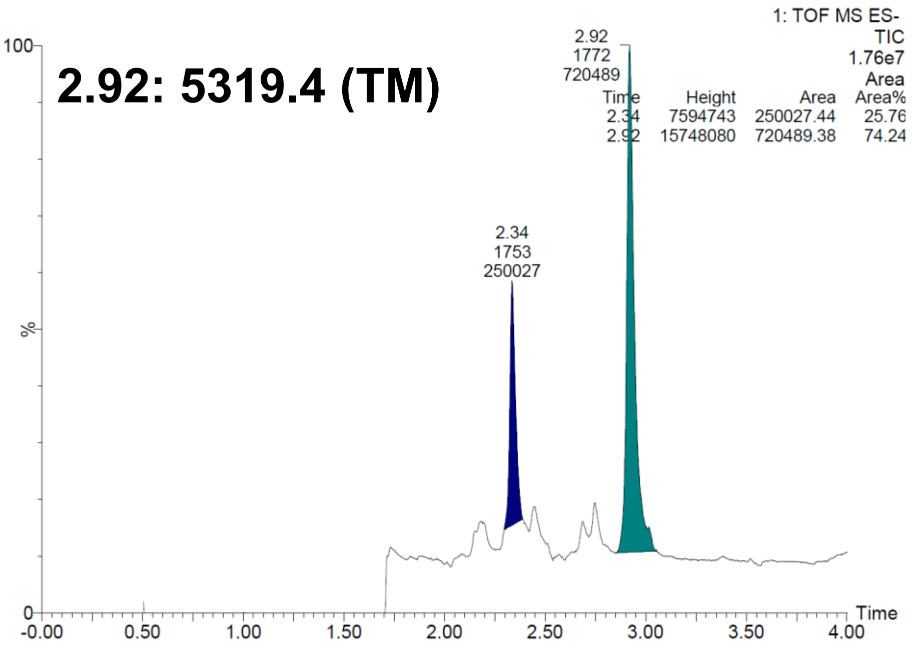

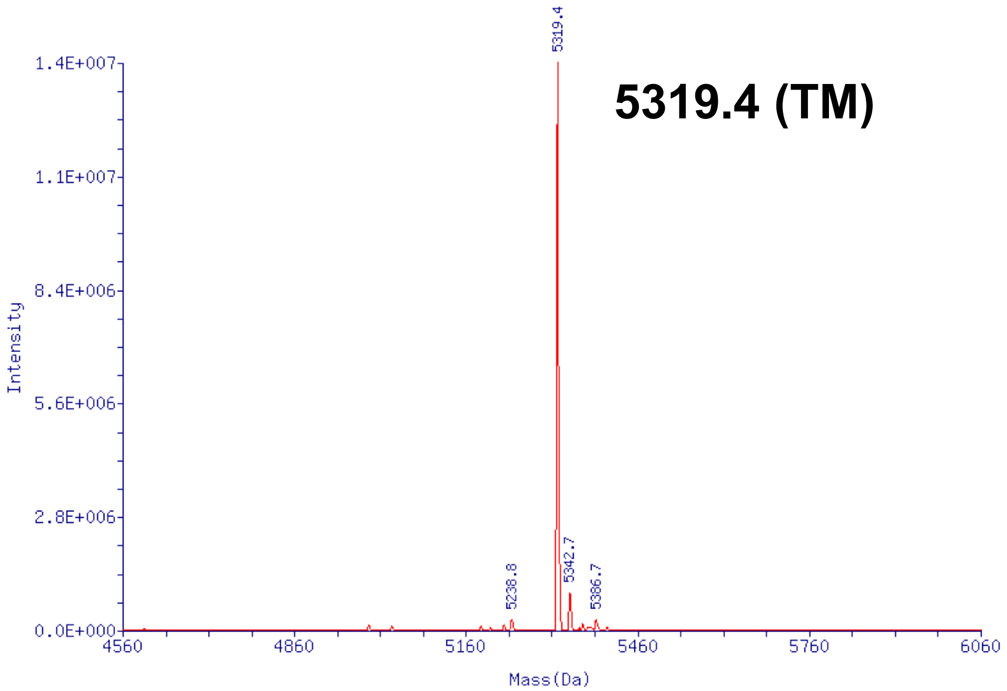

8g

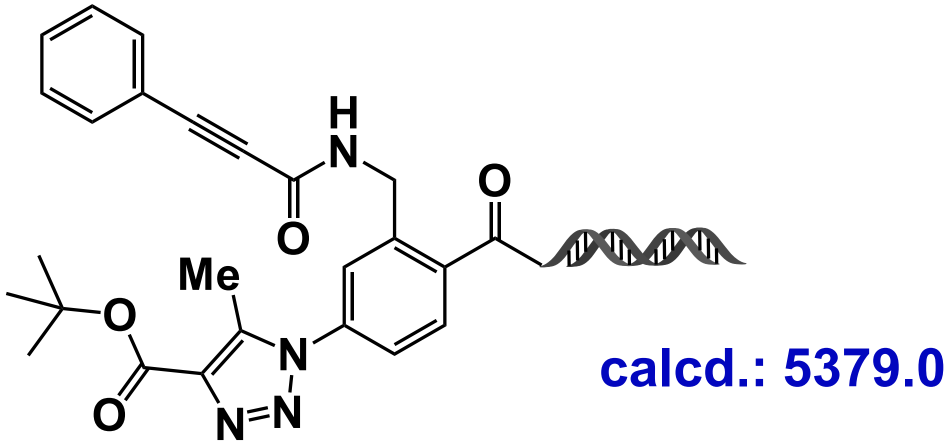
Me
O
N
H
calcd.: 5379.0

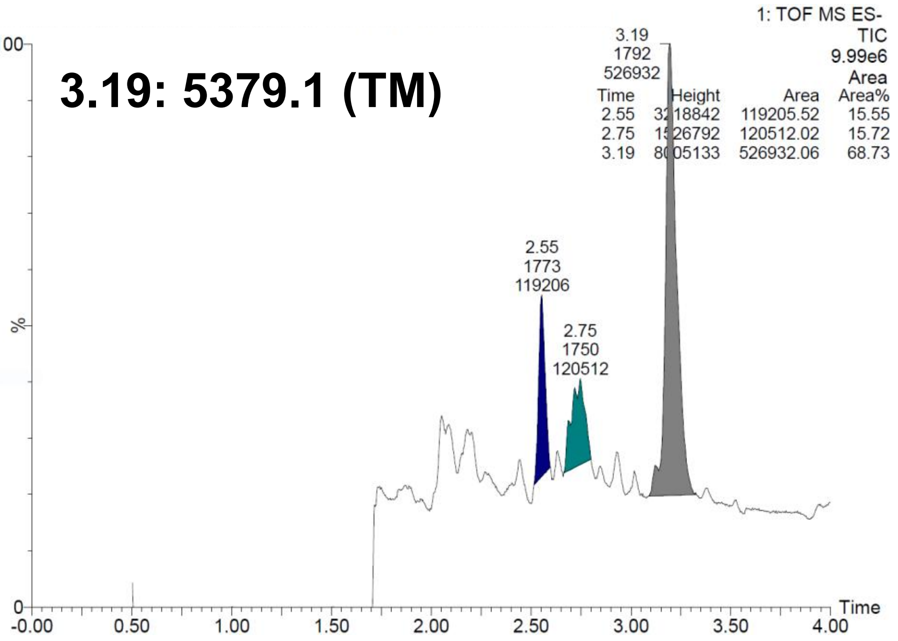
3.19: 5379.1 (TM)
1: TOF MS ES-
TIC
9.99e6
Area
3.19
1792
526932
Time Height Area Area%
2.55 3[illegible]18842 119205.52 15.55
2.75 1[illegible]26792 120512.02 15.72
3.19 8[illegible]05133 526932.06 68.73
2.55
1773
119206
2.75
1750
120512
100
%
0
-0.00
0.50
1.00
1.50
2.00
2.50
3.00
3.50
4.00
Time

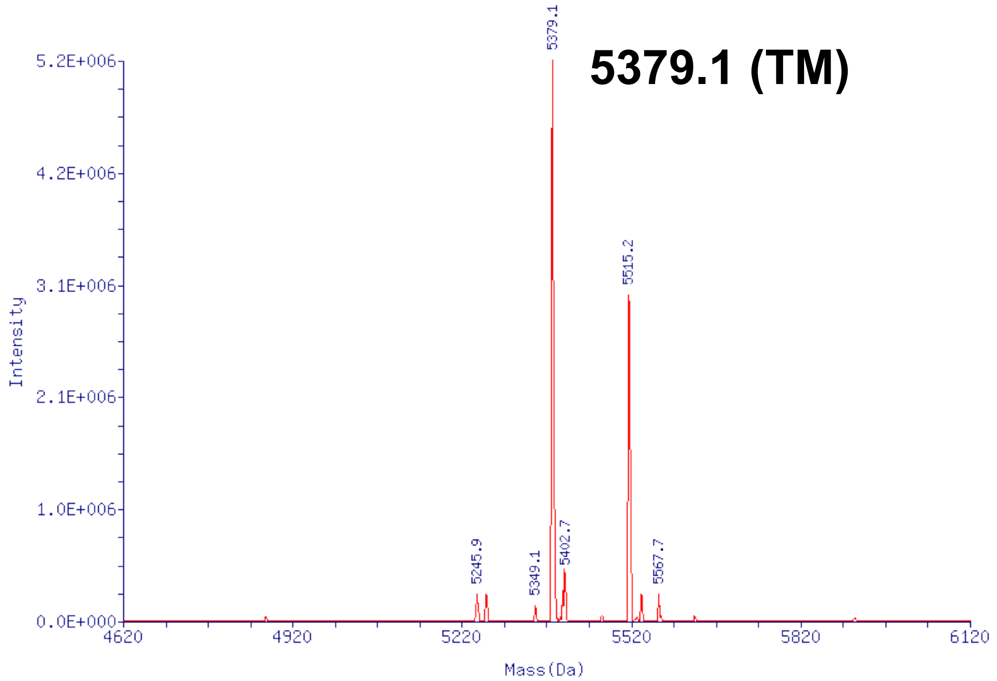
5379.1 (TM)
5379.1
5515.2
5245.9
5349.1
5402.7
5567.7
5.2E+006
4.2E+006
3.1E+006
2.1E+006
1.0E+006
0.0E+000
Intensity
4620
4920
5220
5520
5820
6120
Mass(Da)

2.4. Selective Reduction of Aromatic Azides: A New Approach for DEL Construction Initiated from C-D-DAP

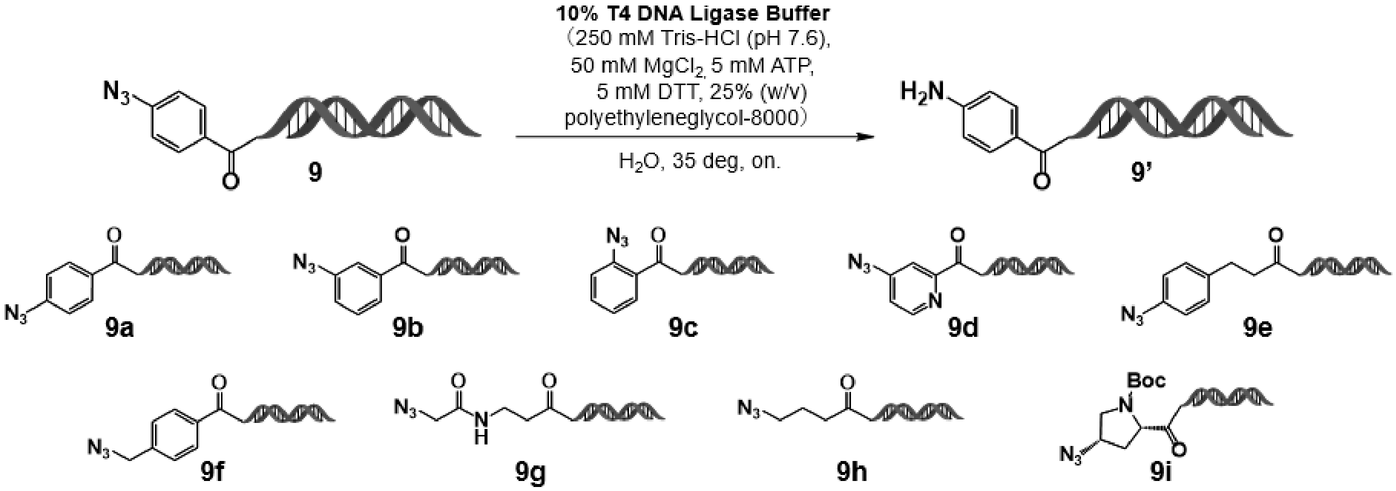

No.	azido-HP	T4 DNA ligase buffer conversion (%)	DTT conversion (%)	TPPTS conversion (%)	2-ME conversion (%)
9a	4-azidobenzoyl-HP	quant.	quant.	quant.	no reaction
9b	3-azidobenzoyl-HP	quant.	quant.	quant.	no reaction
9c	2-azidobenzoyl-HP	quant.	quant.	quant.	no reaction
9d	5-azidopicolinoyl-HP	quant.	quant.	quant.	no reaction
9e	3-(4-azidophenyl)-propanoyl-HP	55.0	60.0	quant.	no reaction
9f	4-(azidomethyl)benzoyl-HP	no reaction	no reaction	quant.	no reaction
9g	azidoacethyl-βAla-HP	no reaction	no reaction	quant.	no reaction
9h	4-azidobutanoyl-HP	no reaction	no reaction	quant.	no reaction
9i	(2*S*,4*S*)-4-azido-1-(*tert*-butoxycarbonyl)pyrrolidine-2-carbonyl-HP	no reaction	no reaction	quant.	no reaction

9a: starting material (SM)

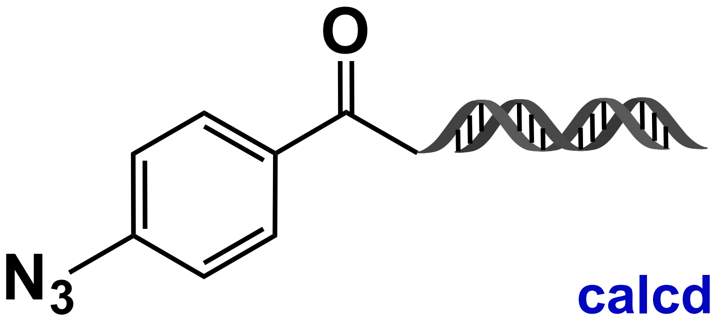

calcd.: 5082.1

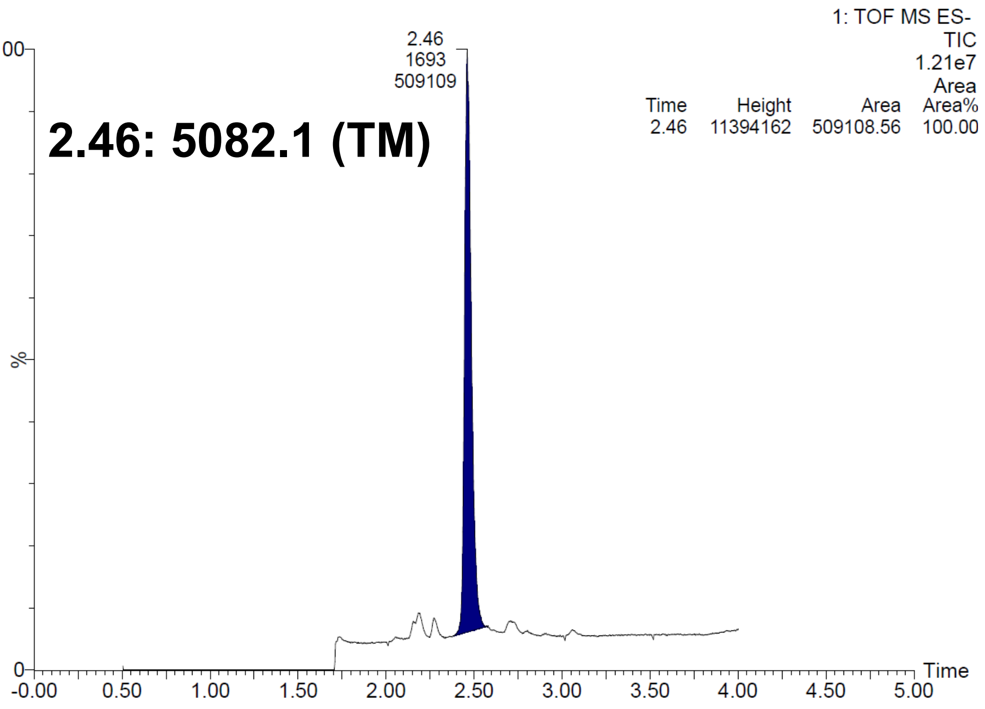

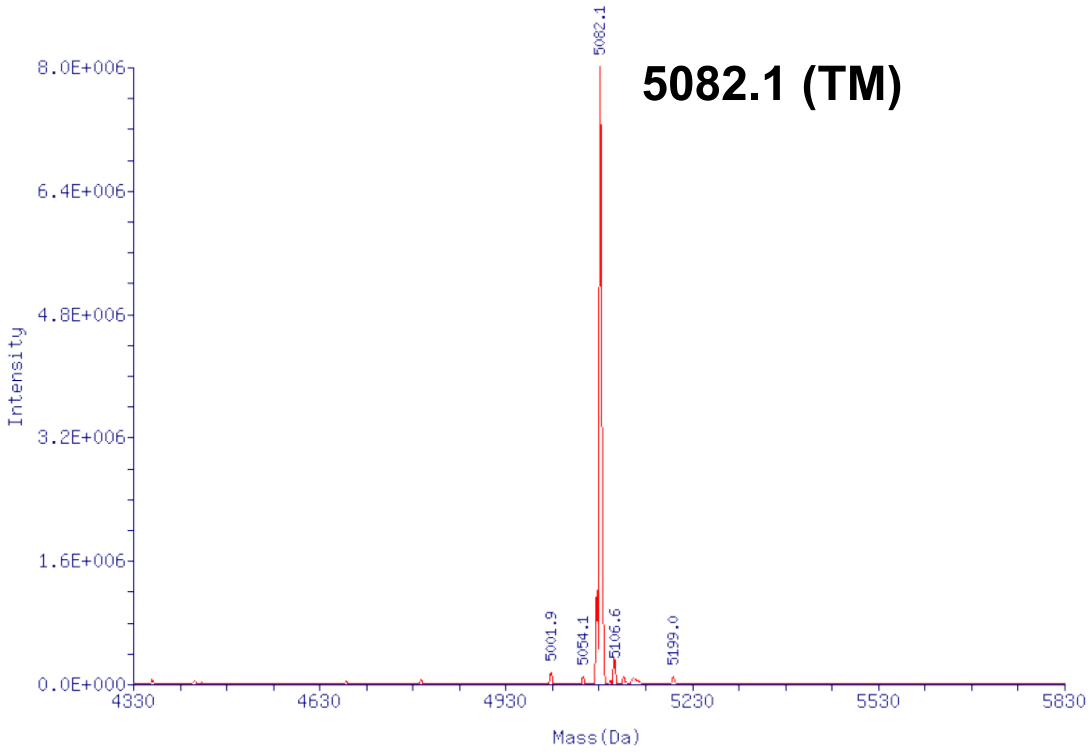

9b : starting material (SM)

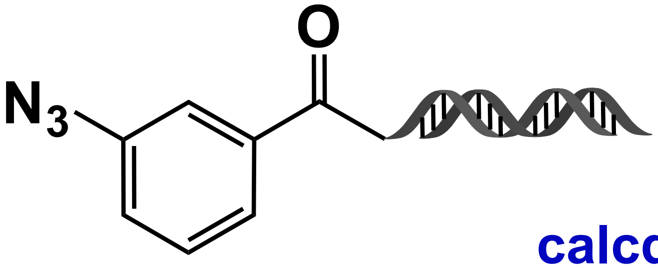

calcd.: 5082.1

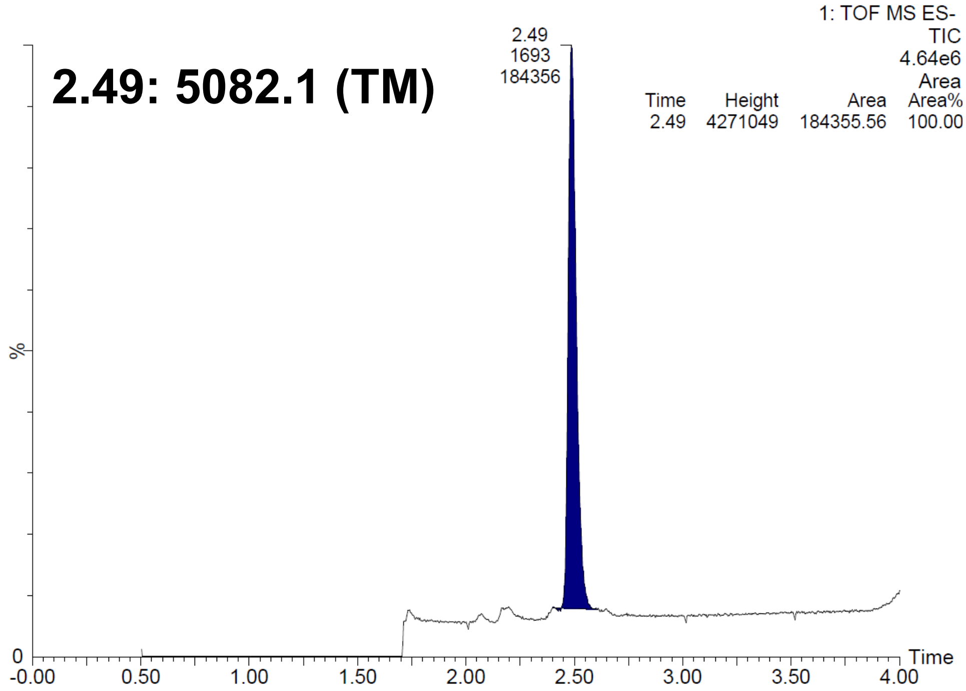

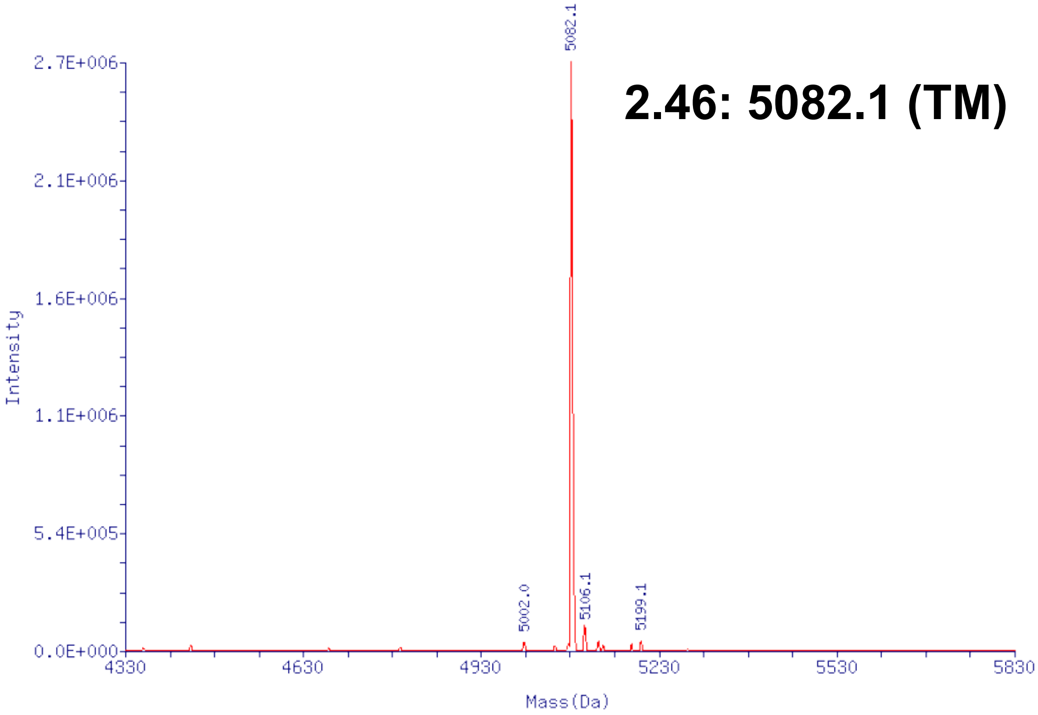

9c : starting material (SM)

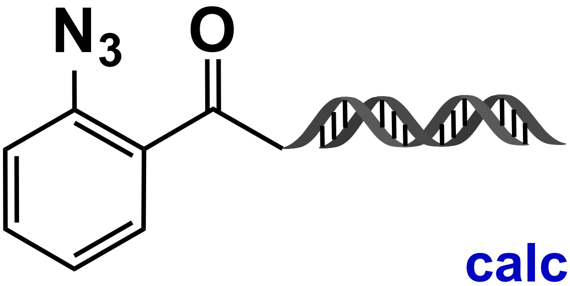

calcd.: 5082.1

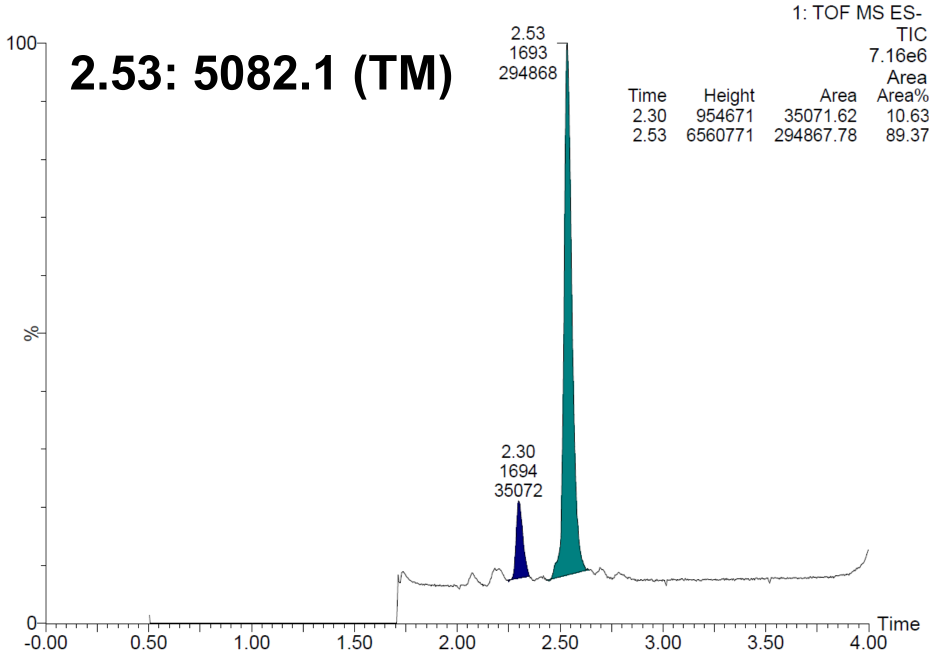

Time	Height	Area	Area Area%
2.30	954671	35071.62	10.63
2.53	6560771	294867.78	89.37

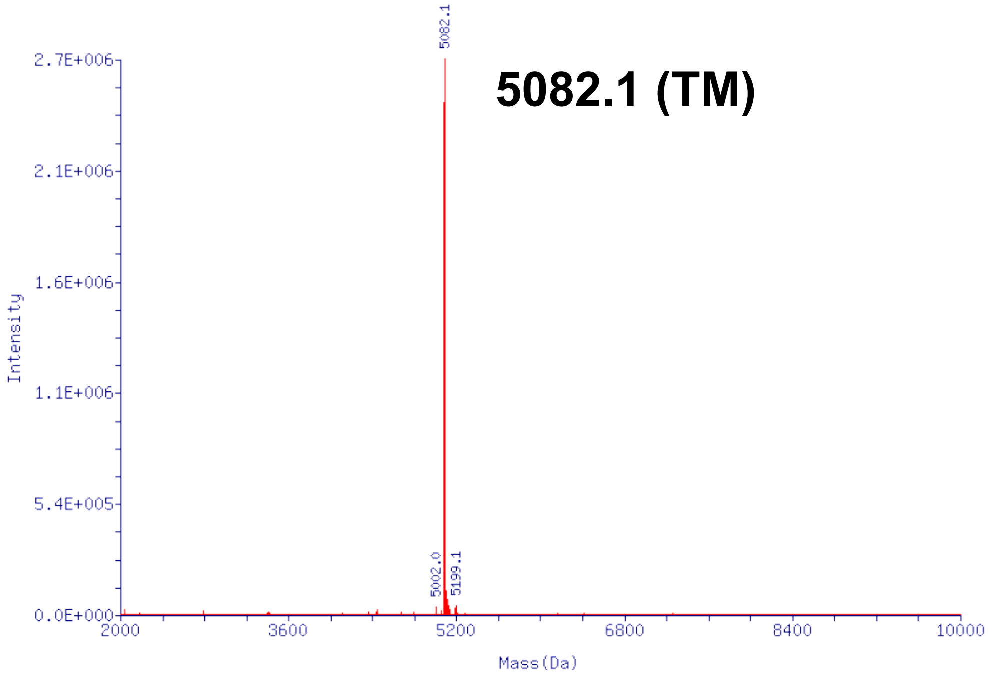

9d : starting material (SM)

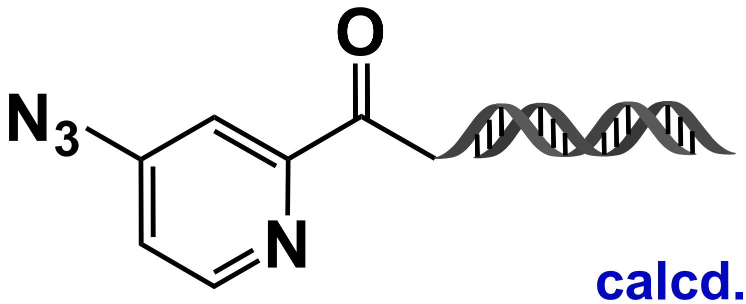

calcd.: 5082.9

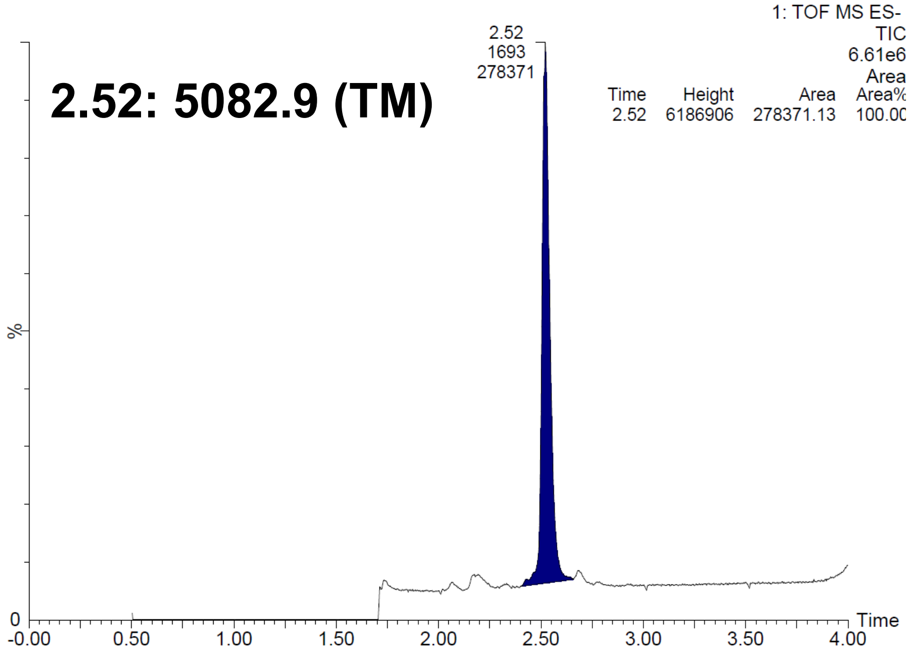

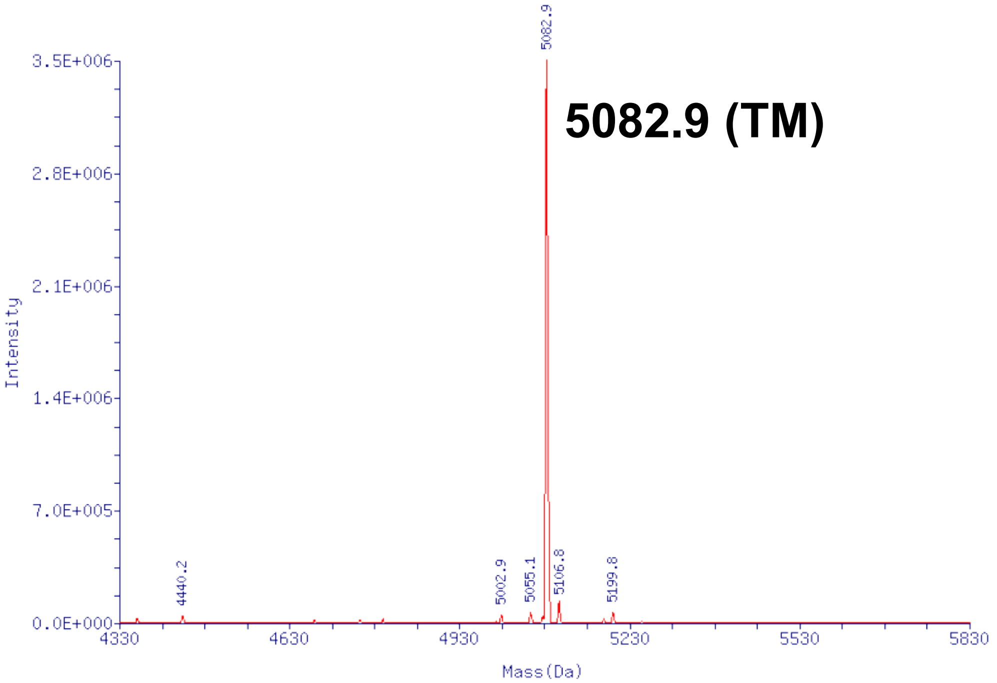

9e : starting material (SM)

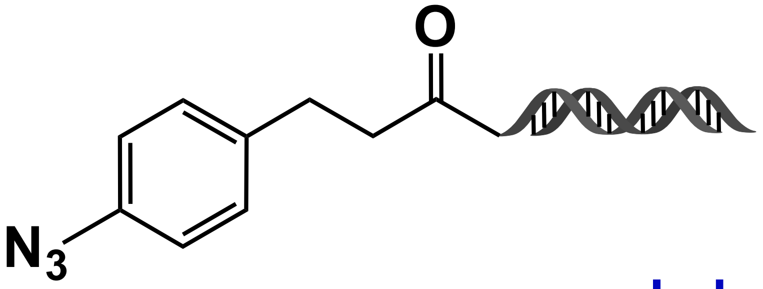

calcd.: 5109.9

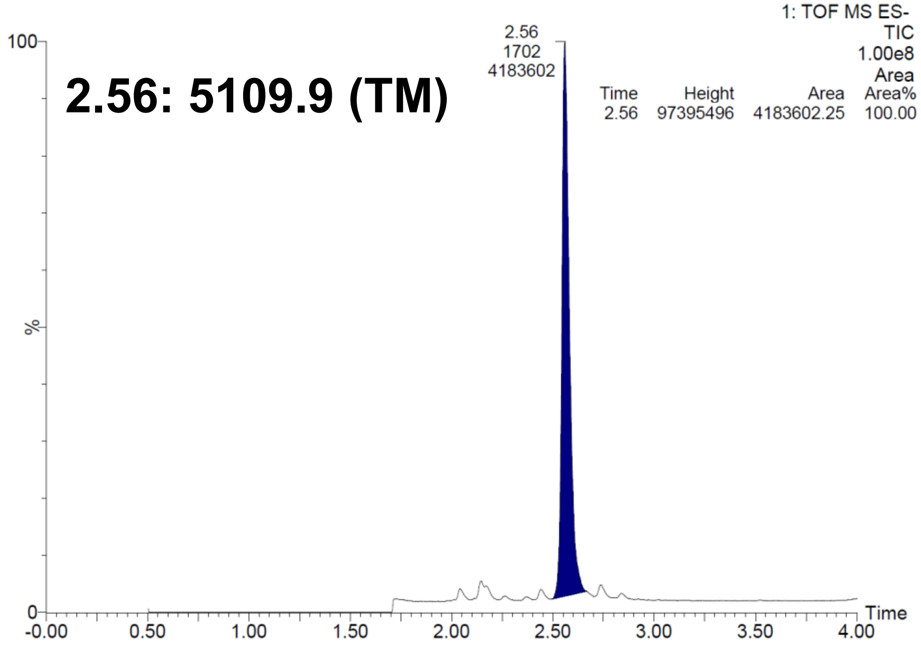

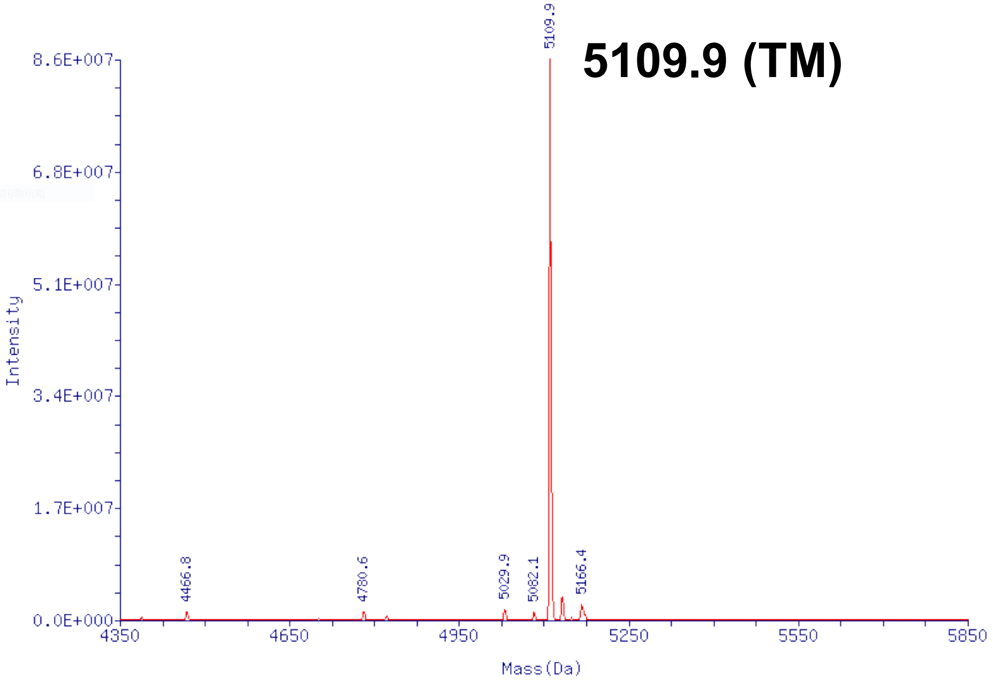

9f : starting material (SM)

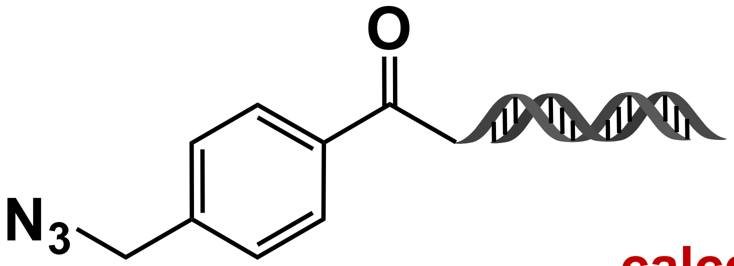

calcd.: 5095.9

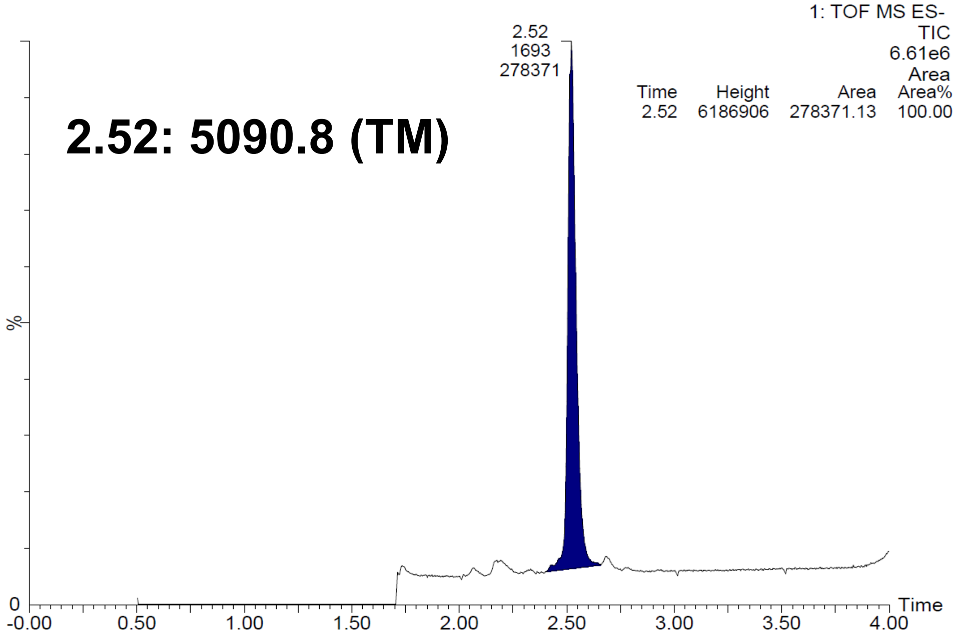

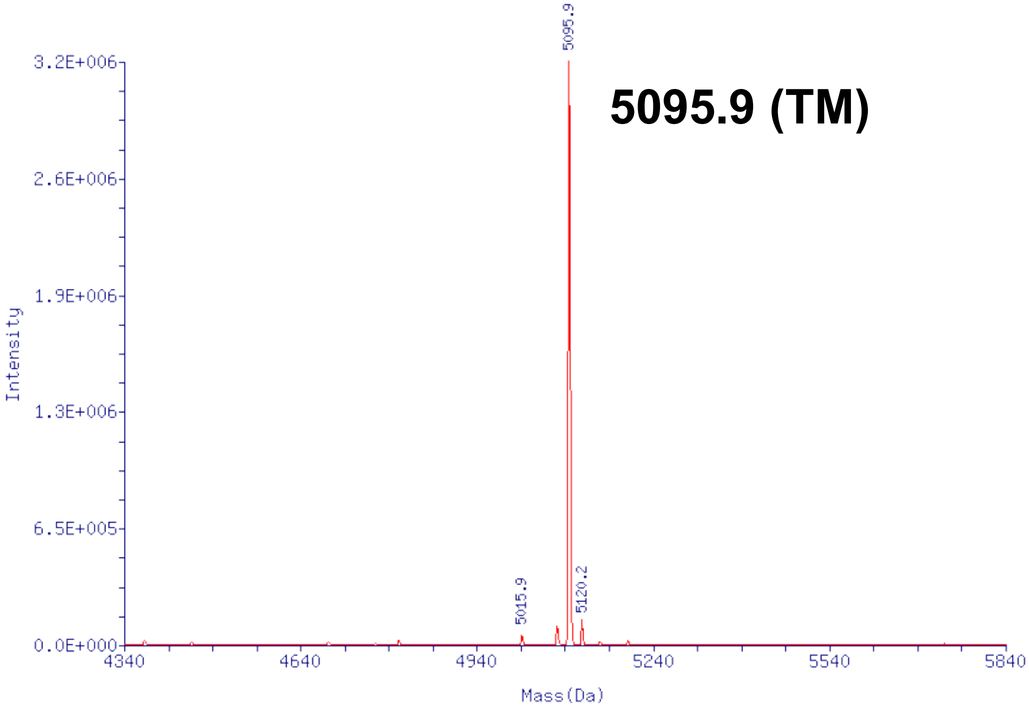

9g : starting material (SM)

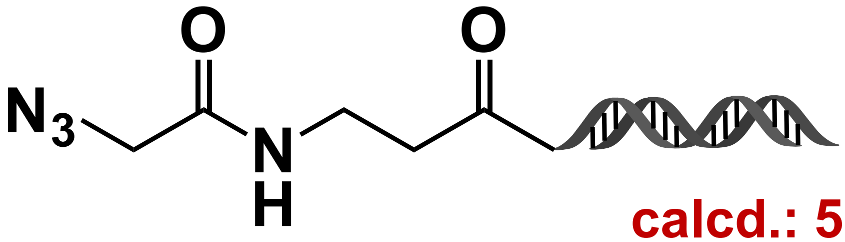

calcd.: 5090.8

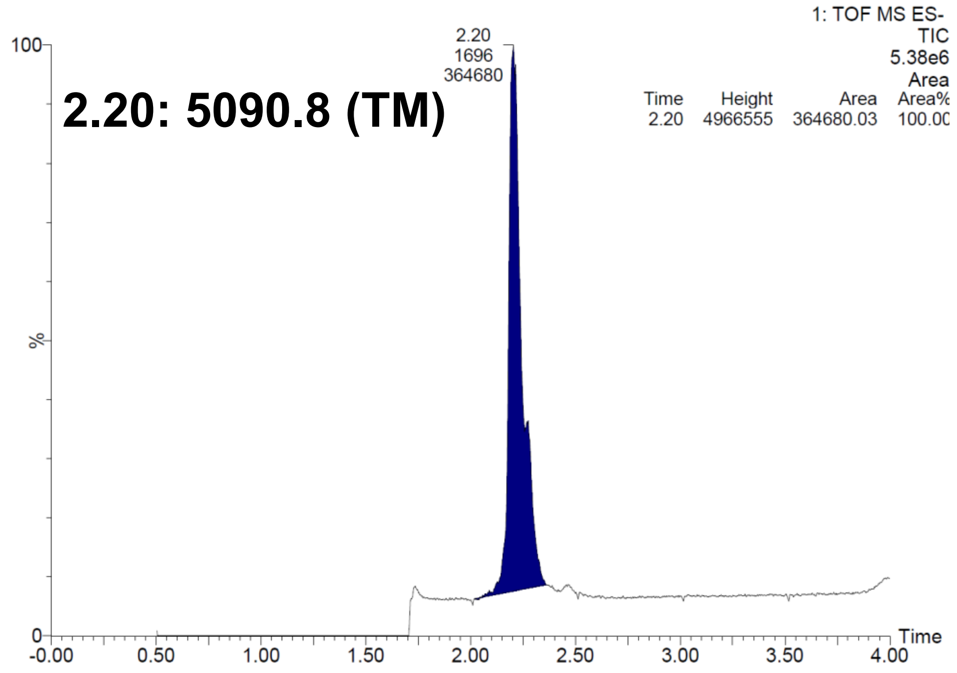

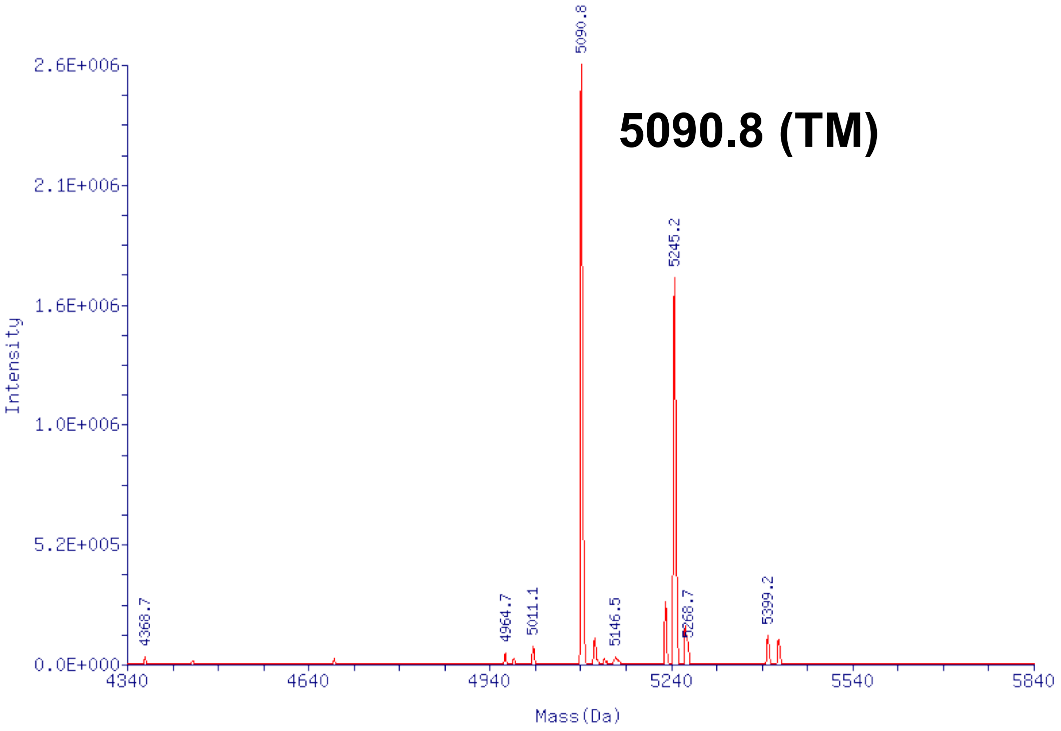

9h : starting material (SM)

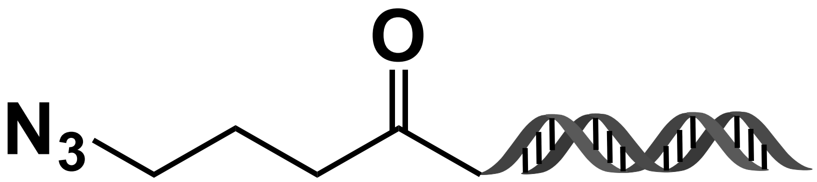

calcd.: 5048.1

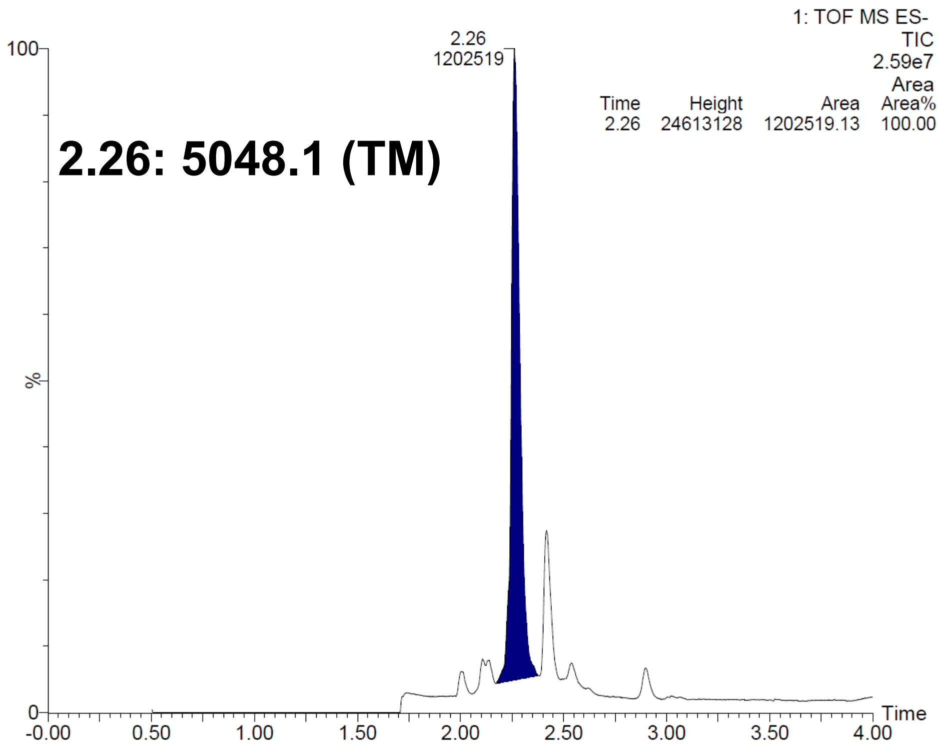

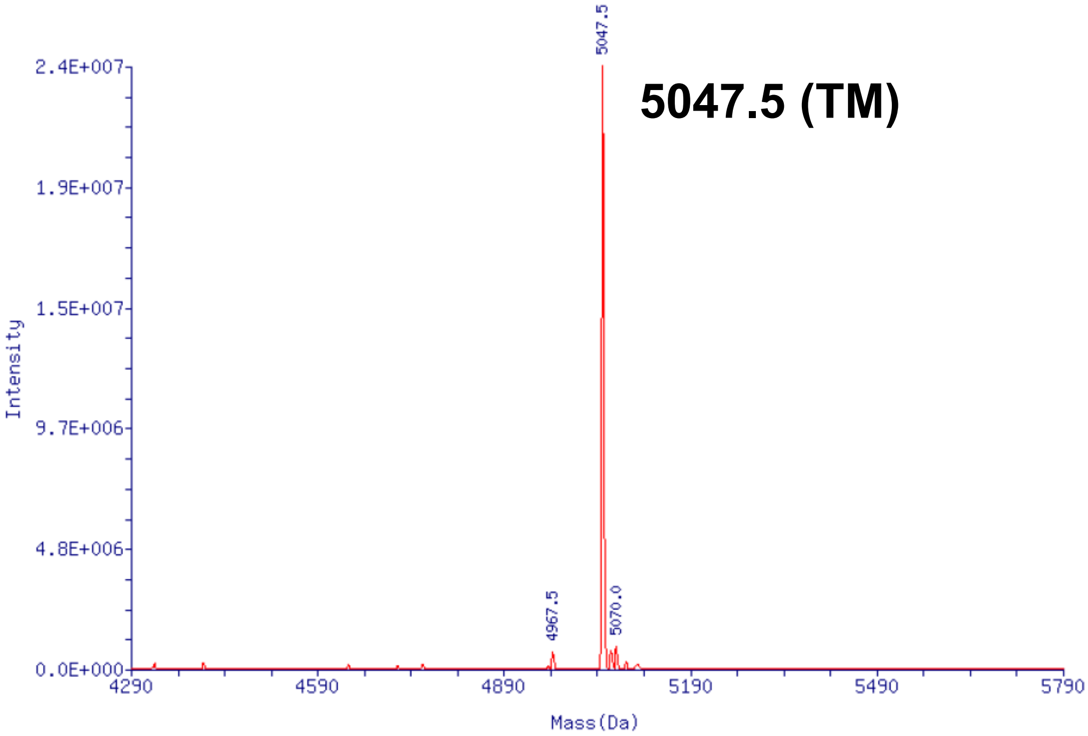

9i : starting material (SM)

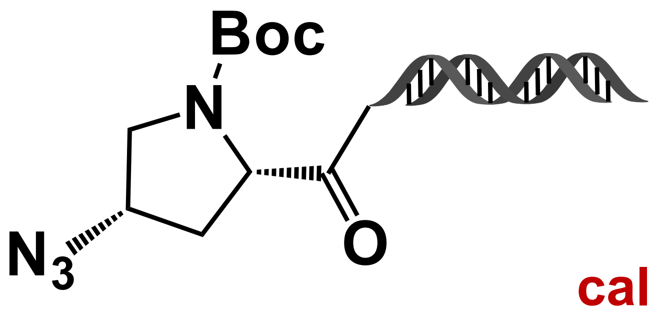

calcd.: 5175.0

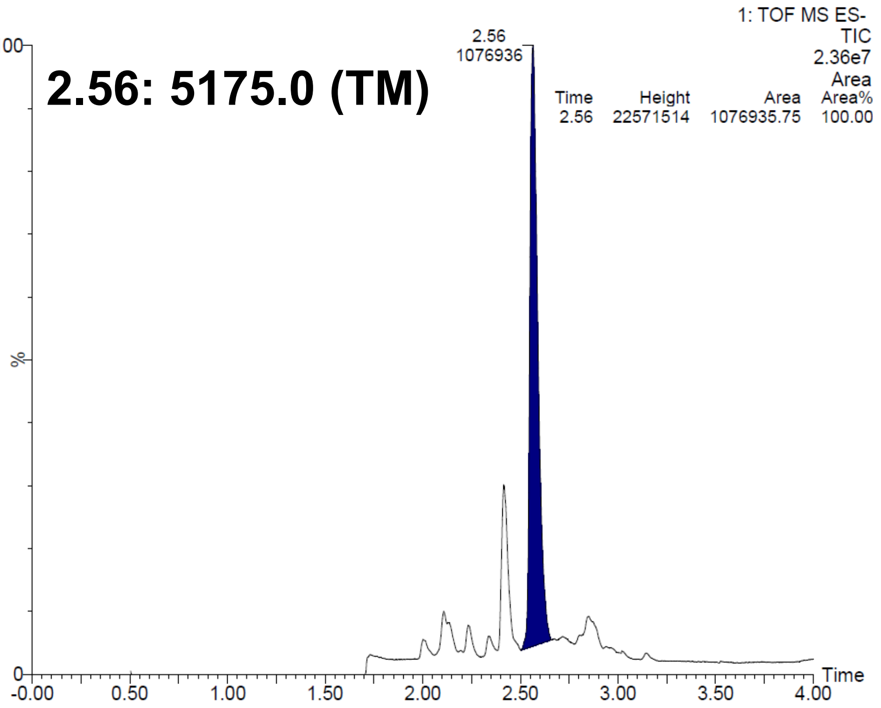

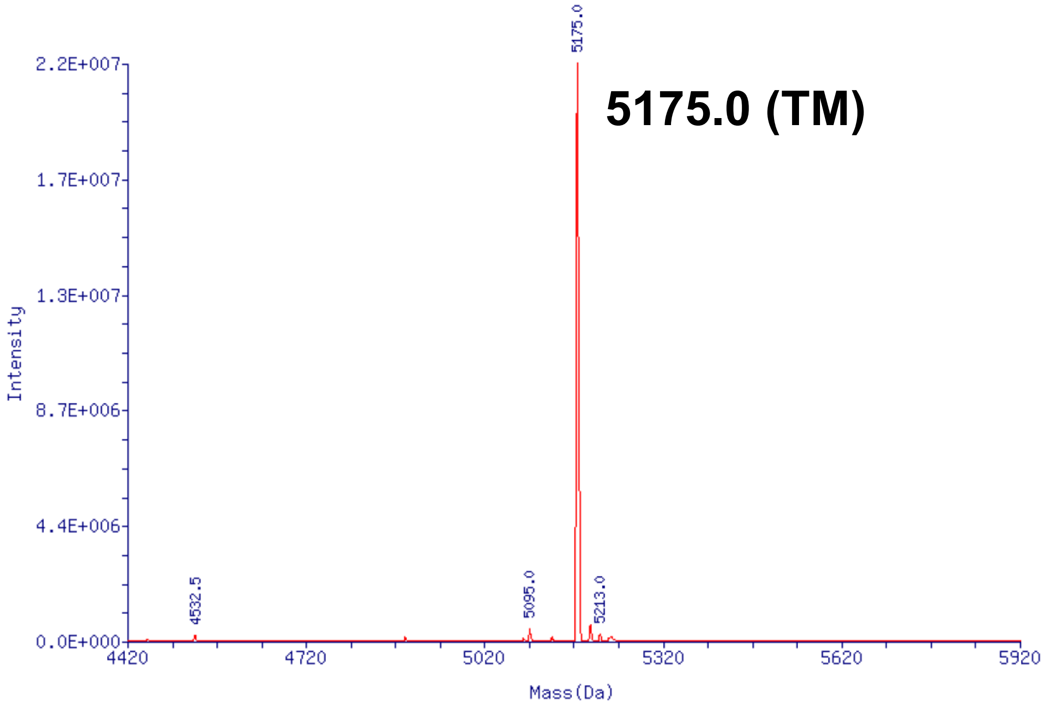

Reduction of 9a with T4 DNA ligase buffer.

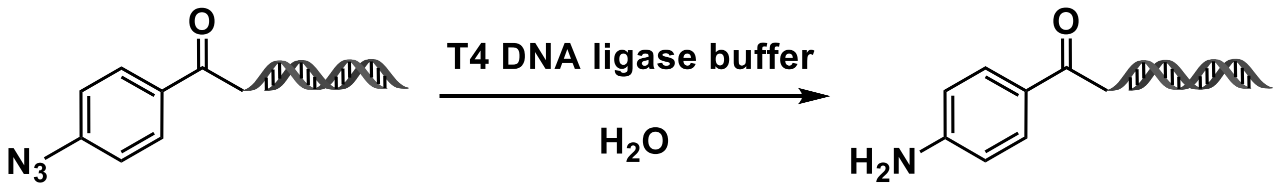

calcd.: 5055.90

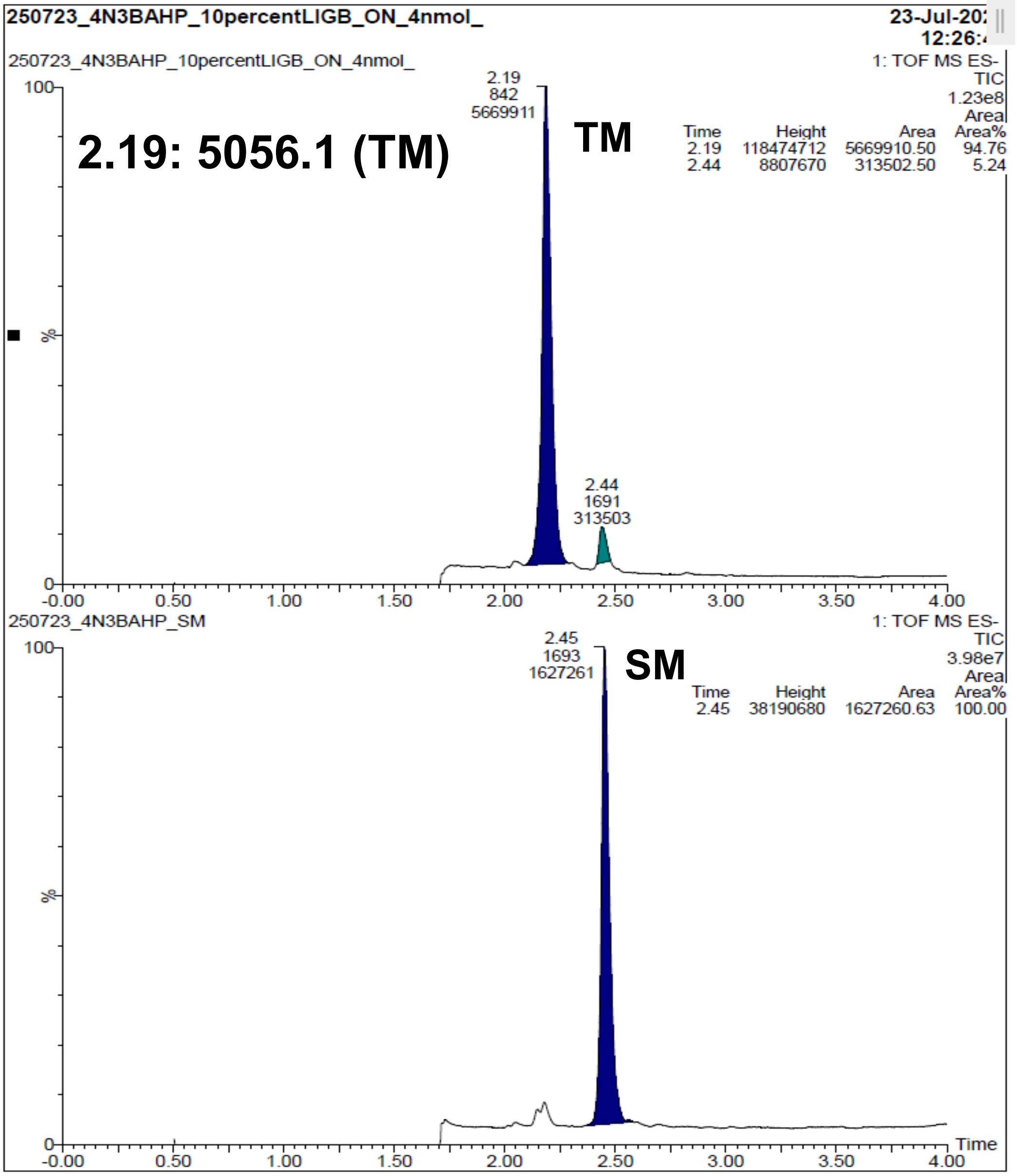

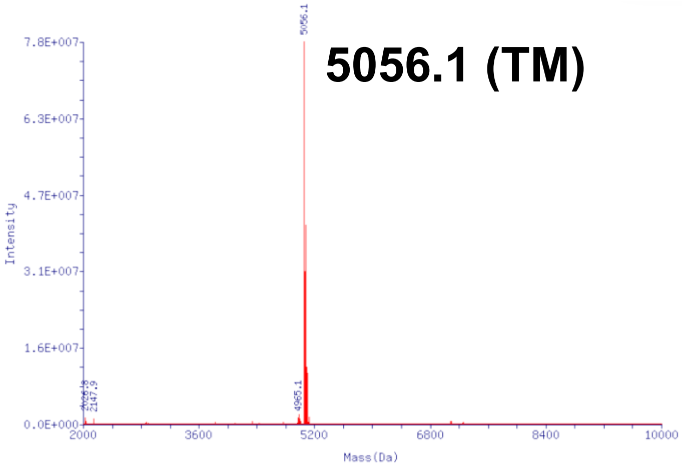

Reduction of 9b with T4 DNA ligase buffer.

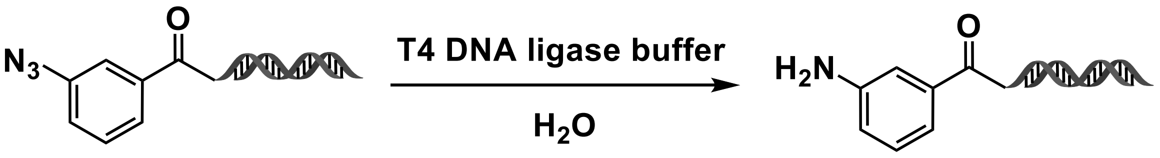

calcd.: 5055.90

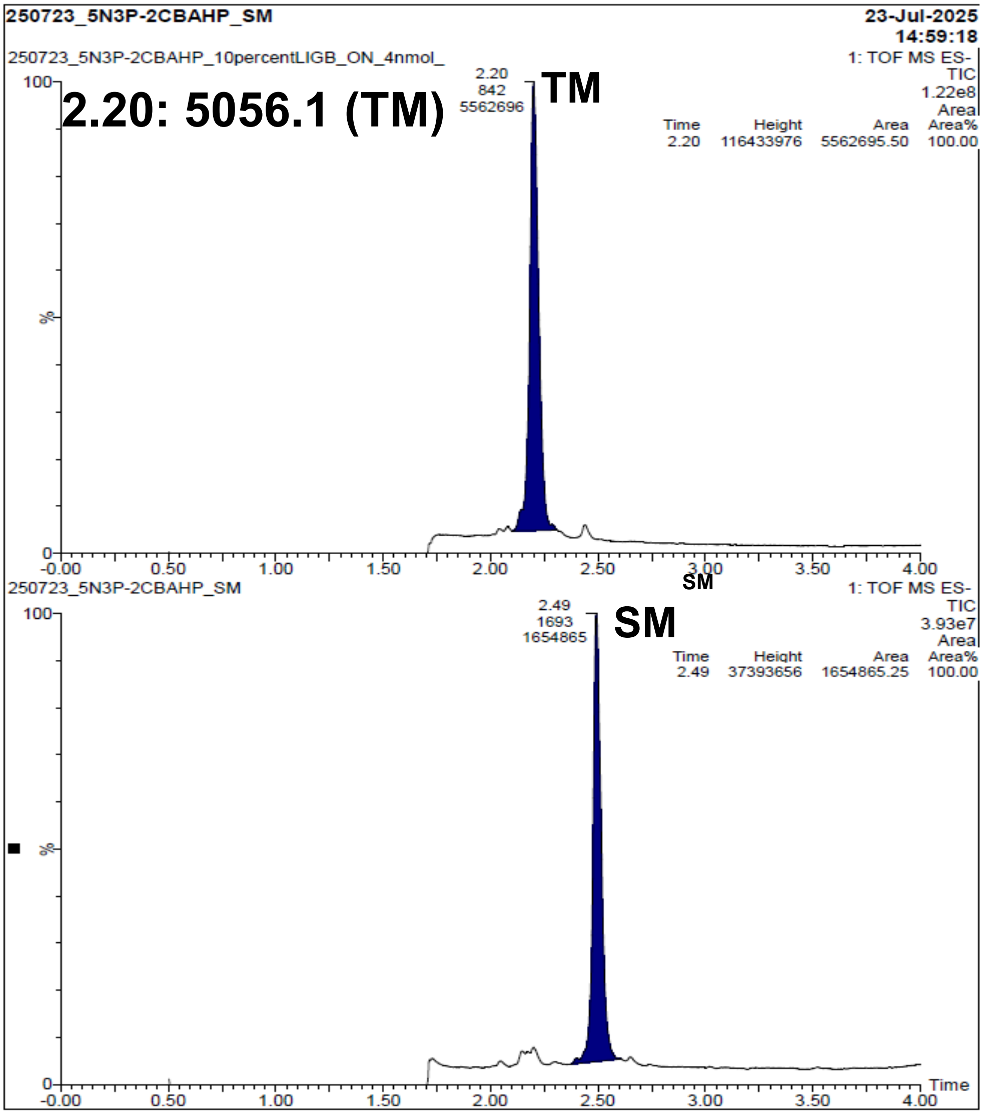

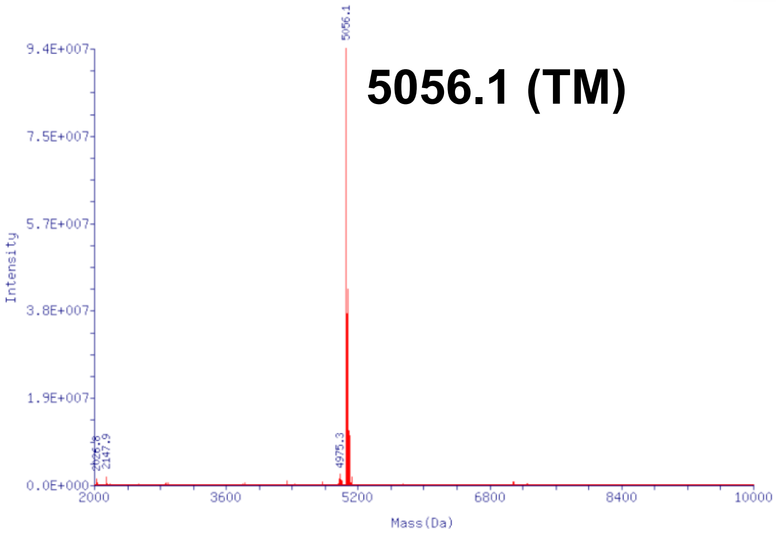

Reduction of 9c with T4 DNA ligase buffer.

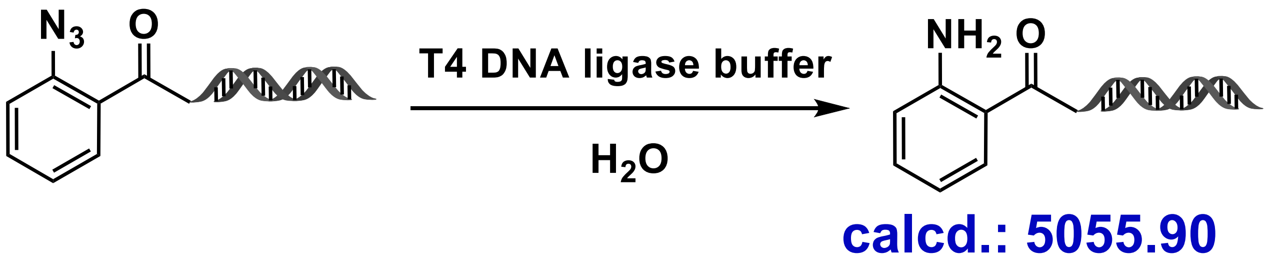

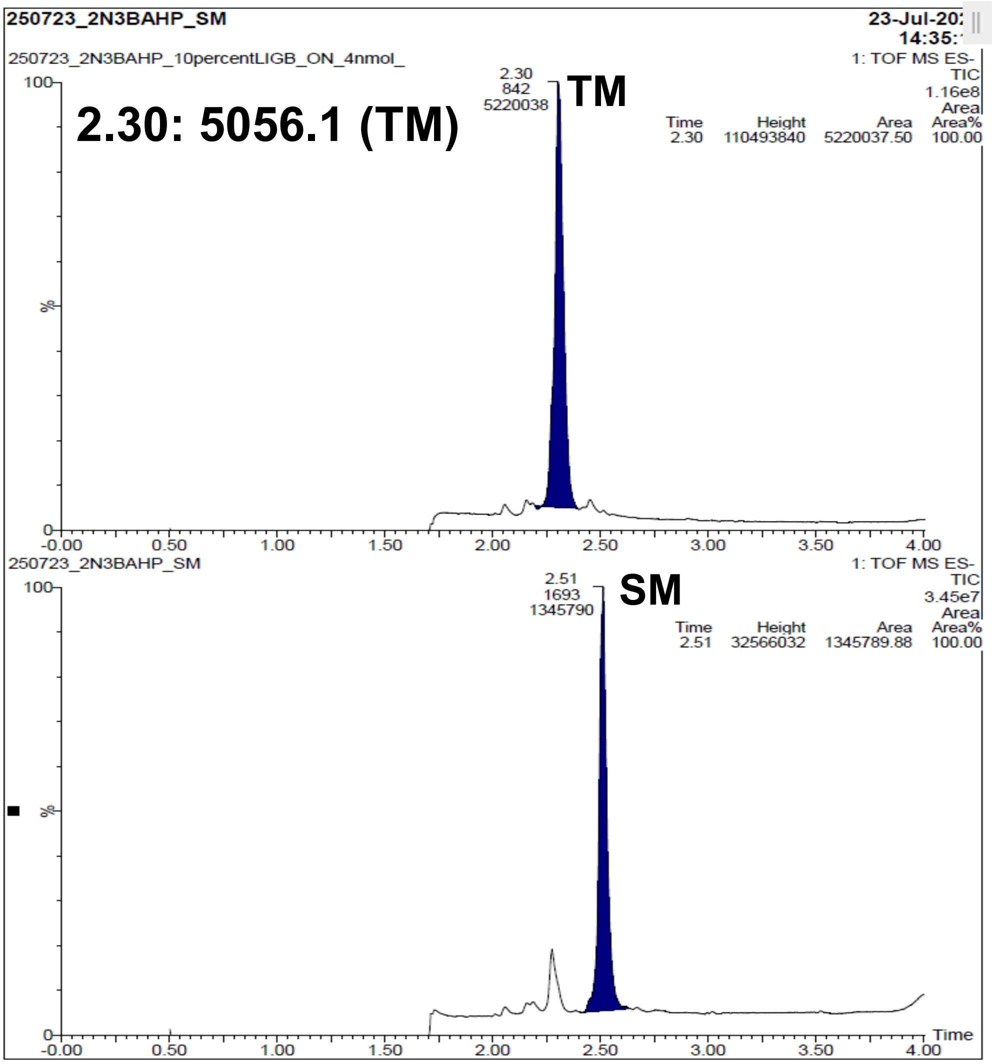

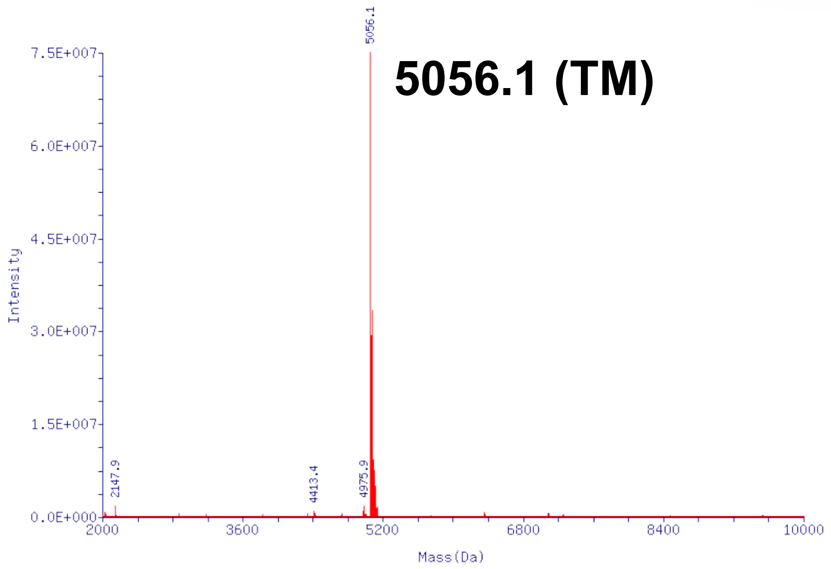

Reduction of 9d with T4 DNA ligase buffer.

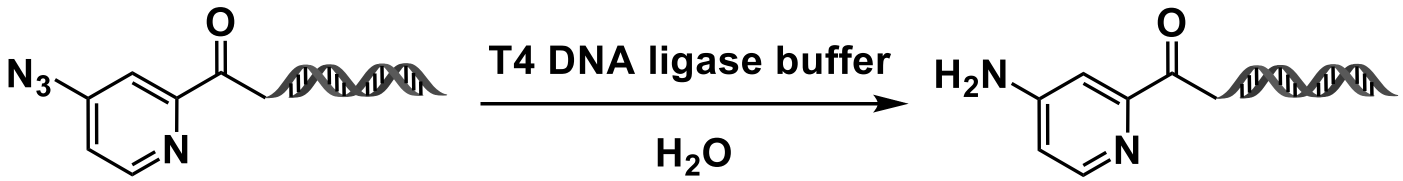

calcd.: 5056.9

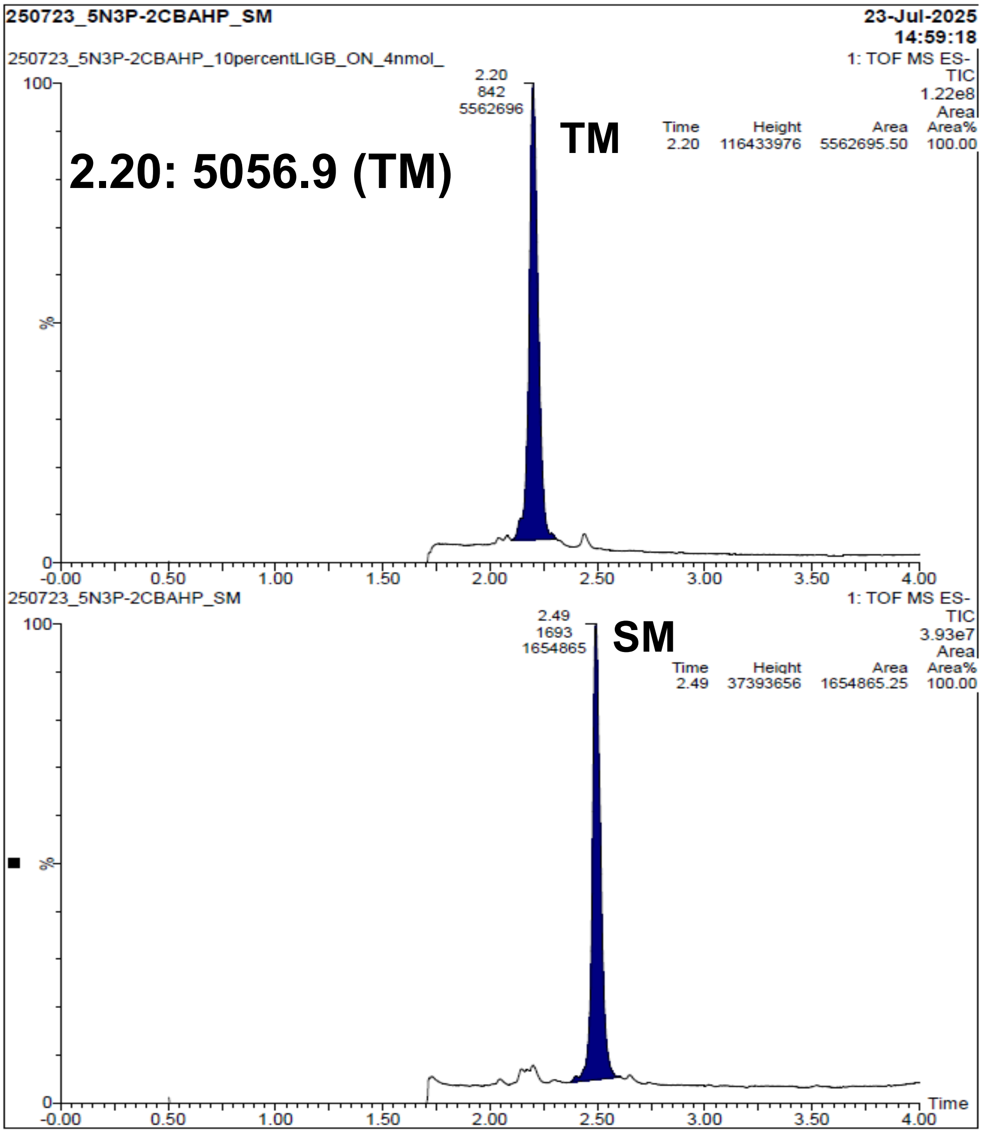

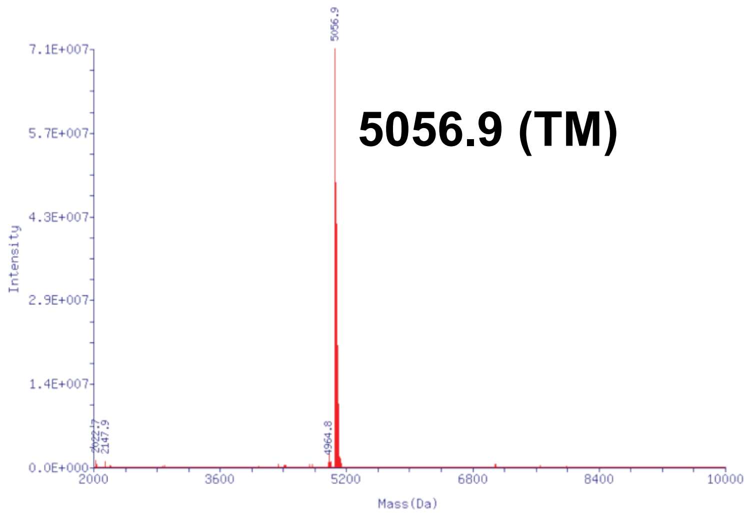

Reduction of 9e with T4 DNA ligase buffer.

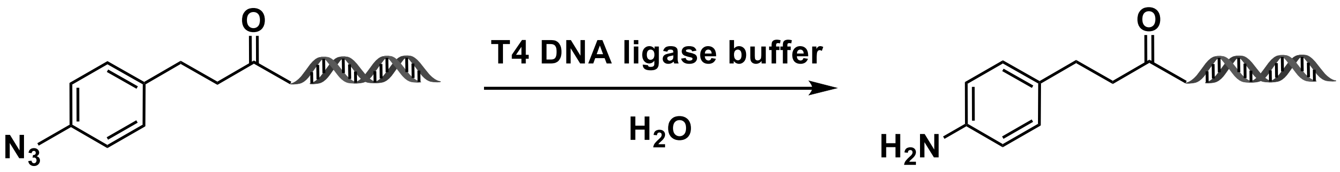

calcd.: 5083.9

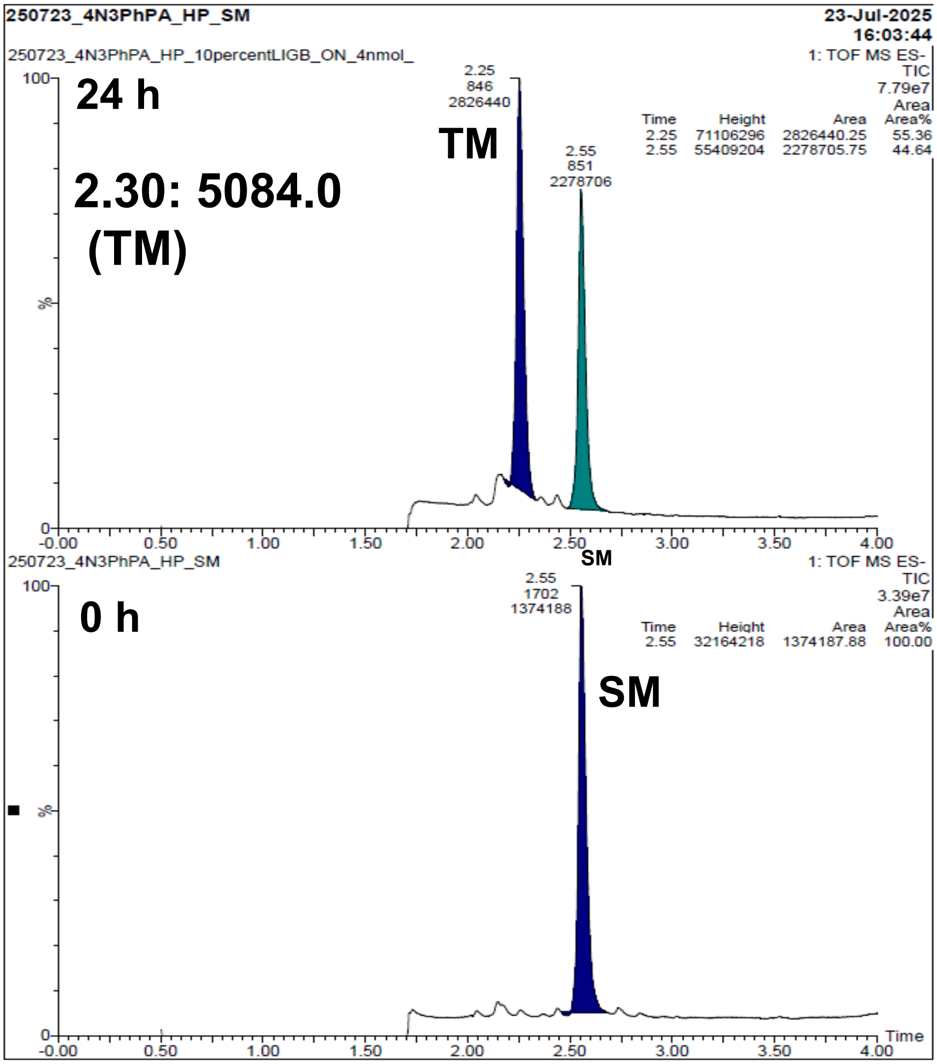

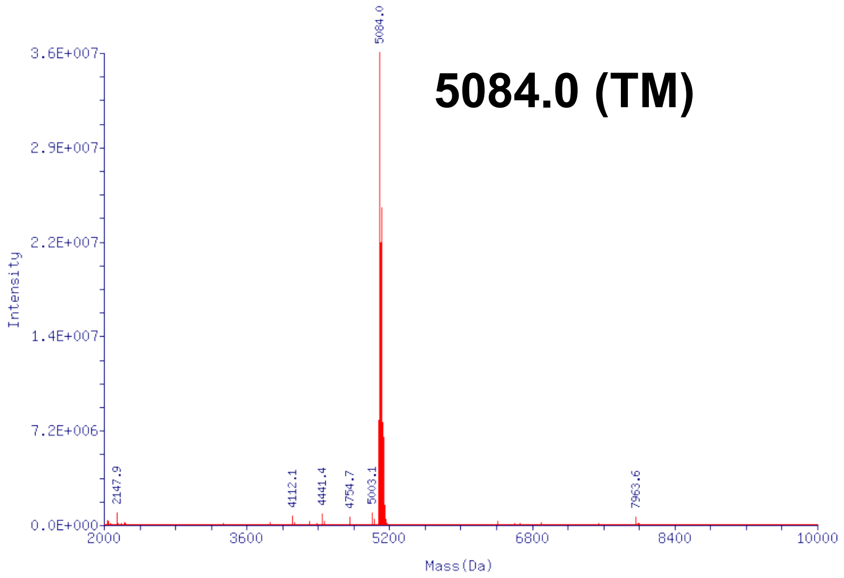

Reduction of 9f with T4 DNA ligase buffer.

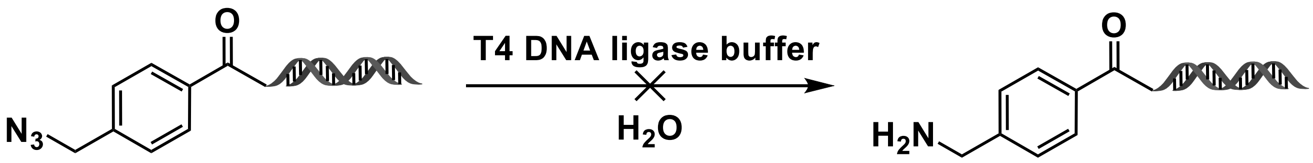

after 24 h

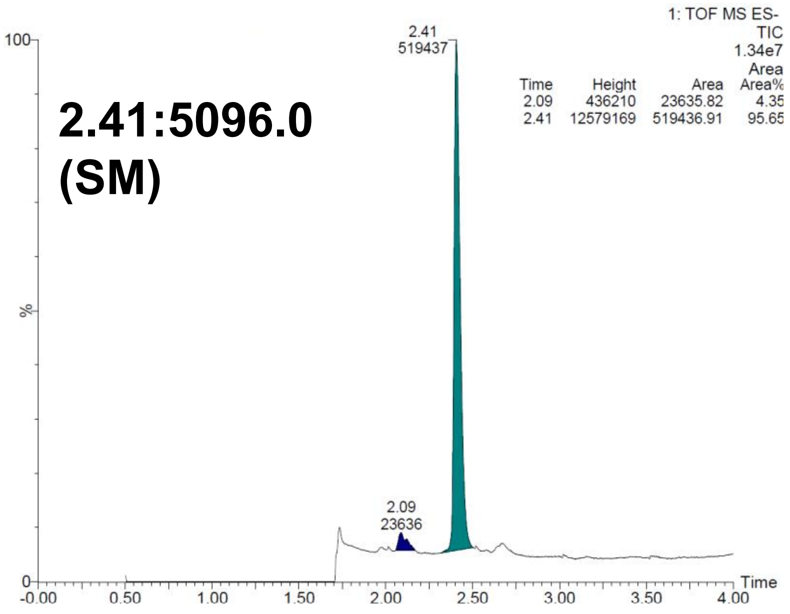

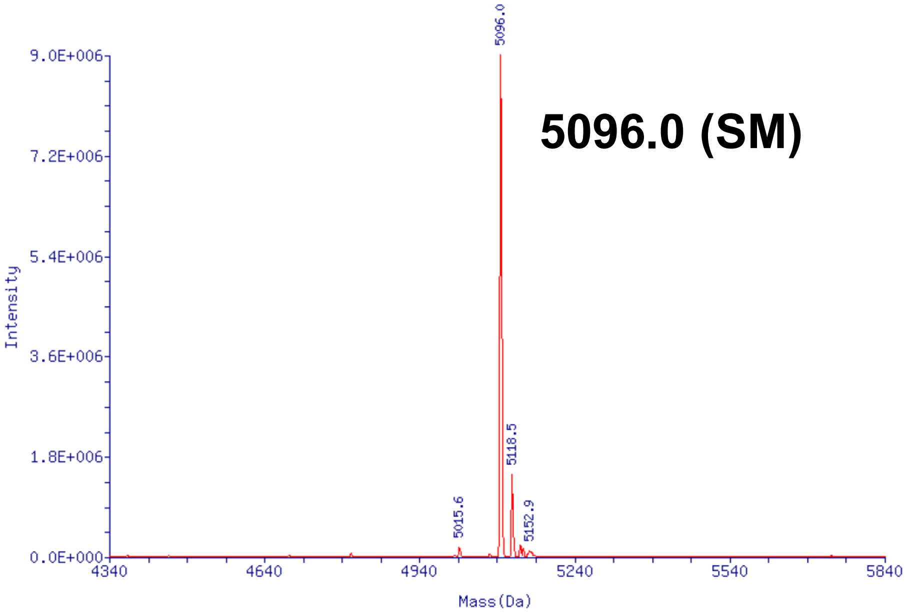

Reduction of 9g with T4 DNA ligase buffer.

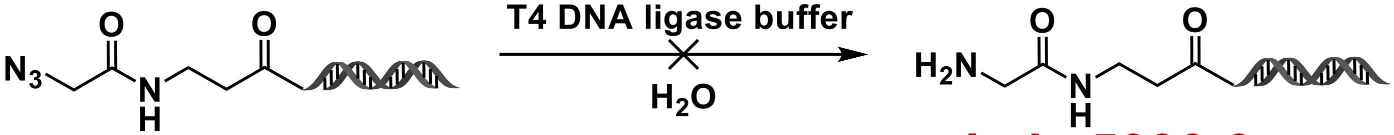

calcd.: 5090.6

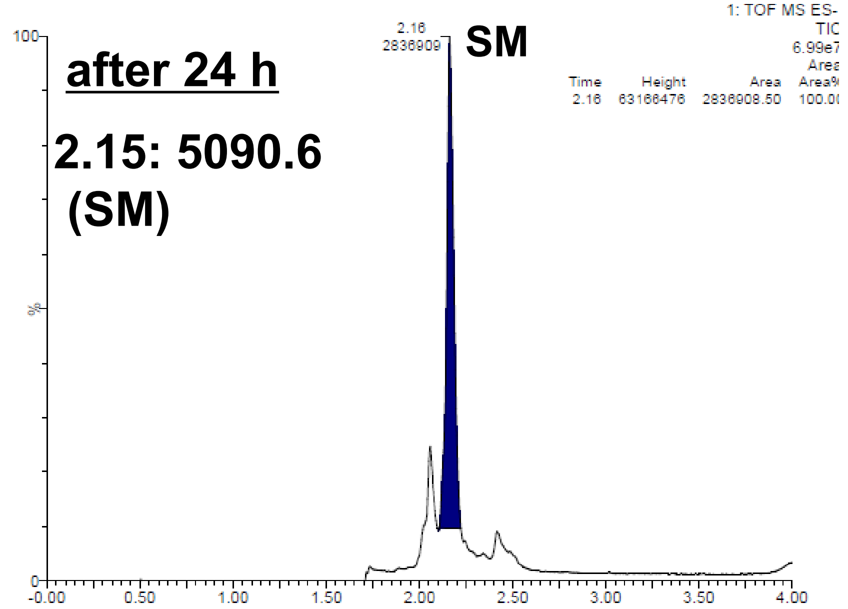

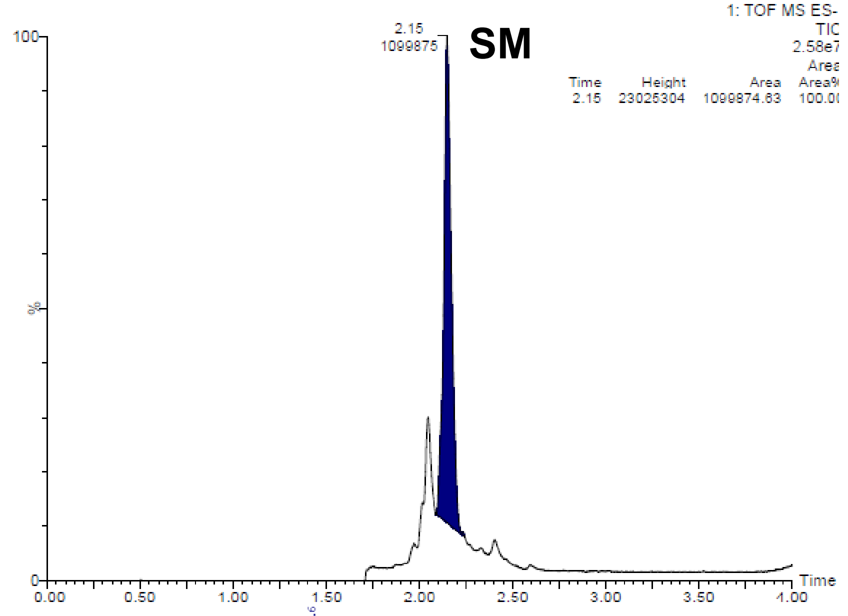

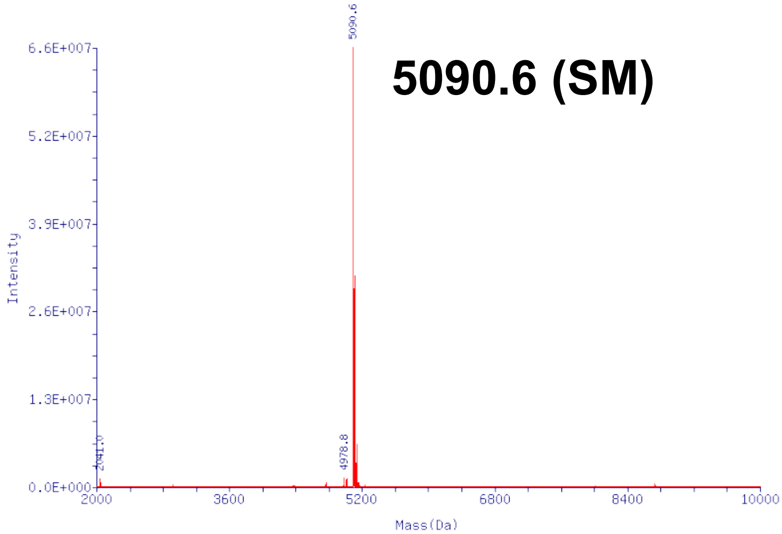

Reduction of 9h with T4 DNA ligase buffer.

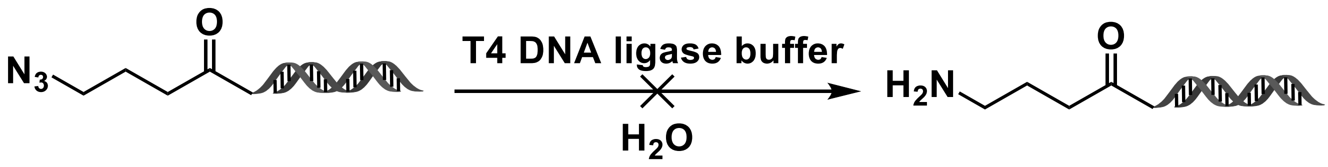

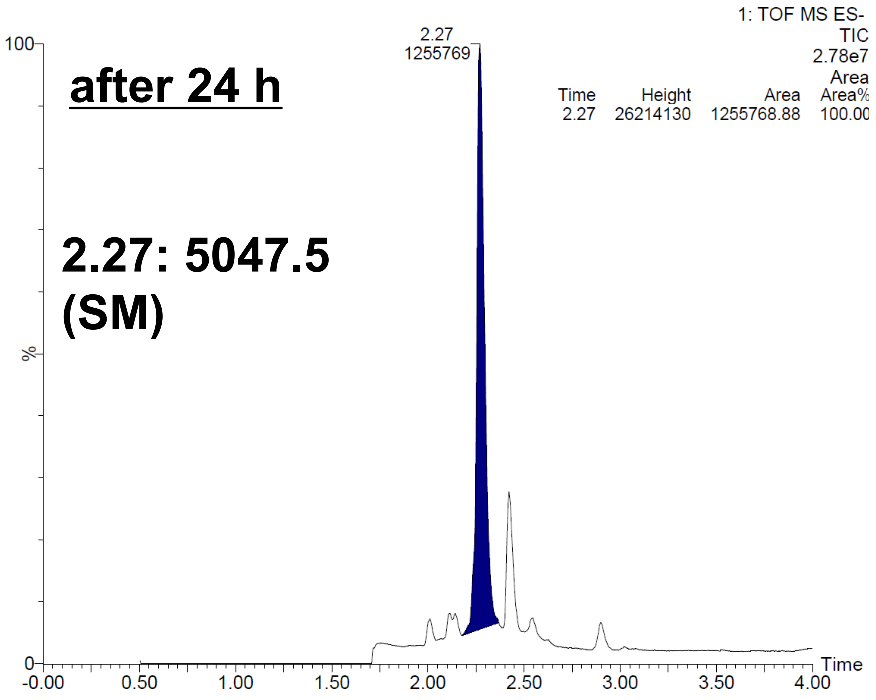

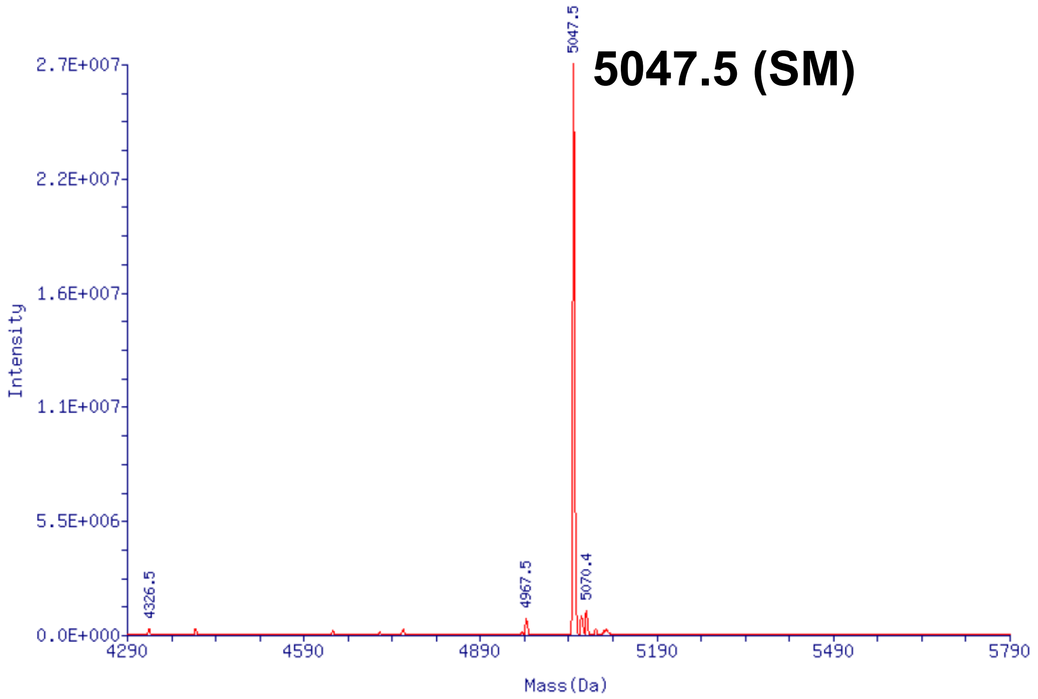

Reduction of 9i with T4 DNA ligase buffer.

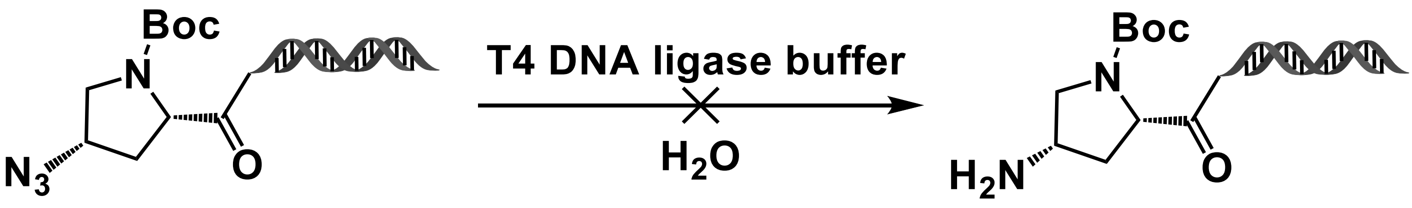

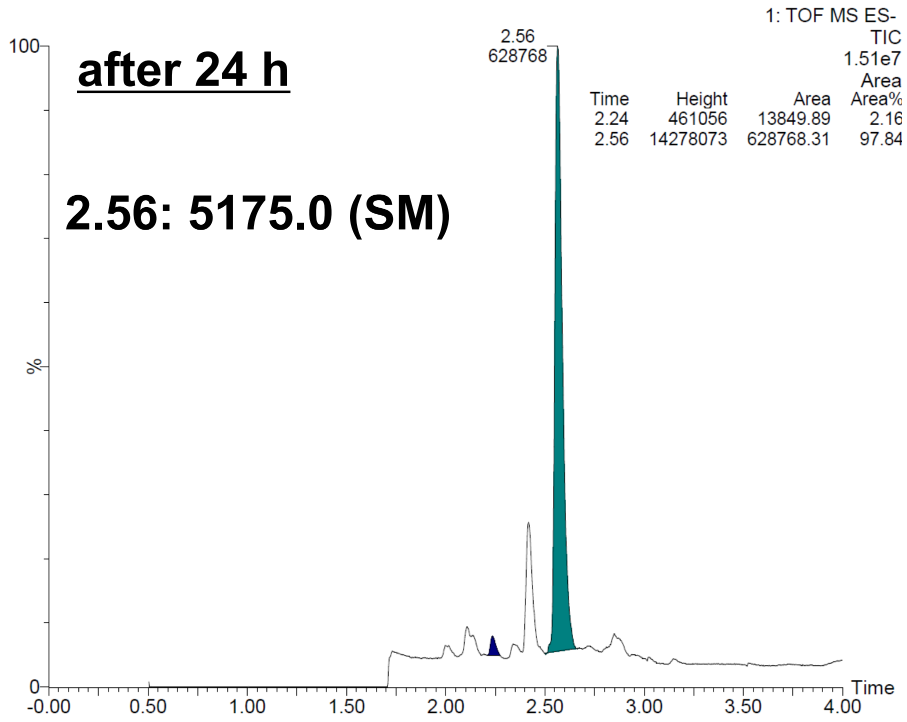

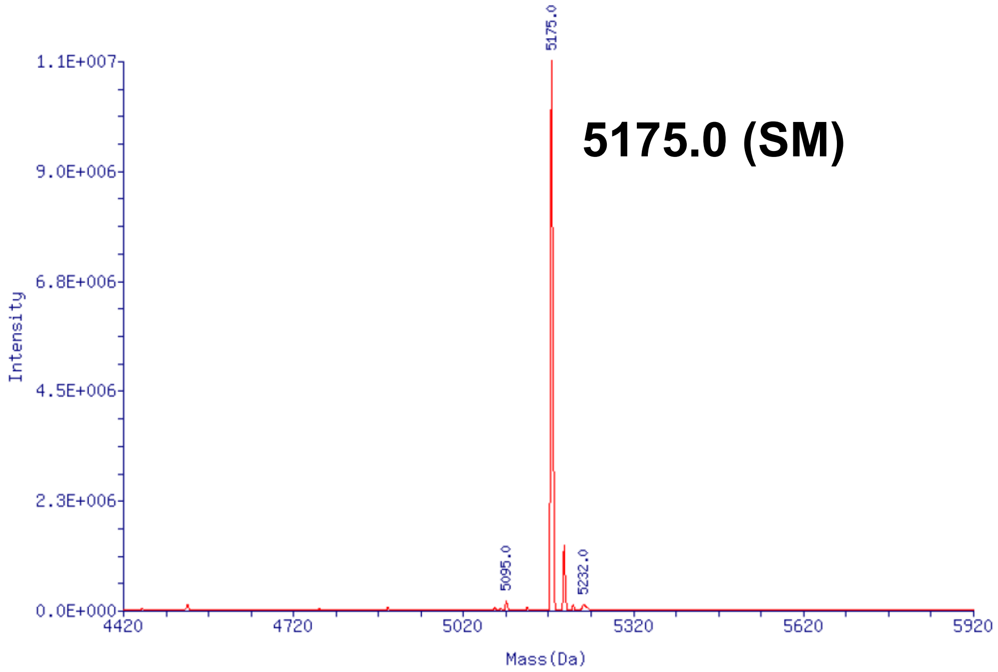

Reduction of 9a with DTT.

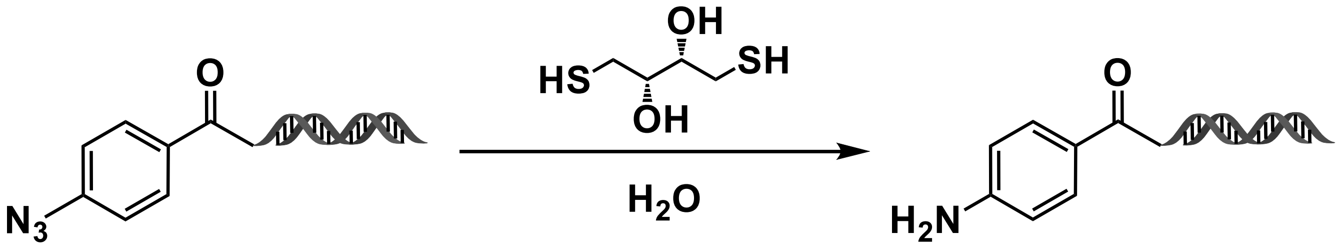

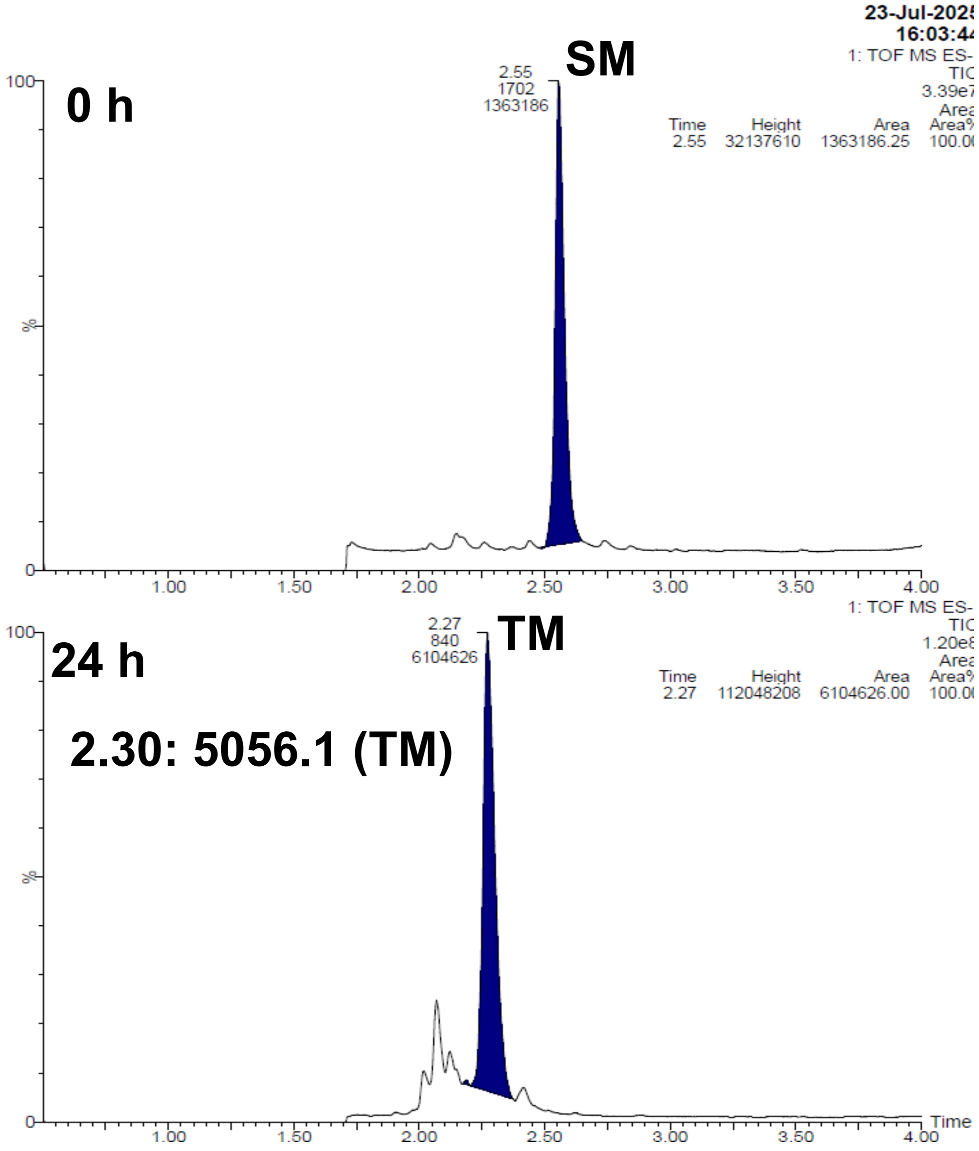

Reduction of 9b with DTT.

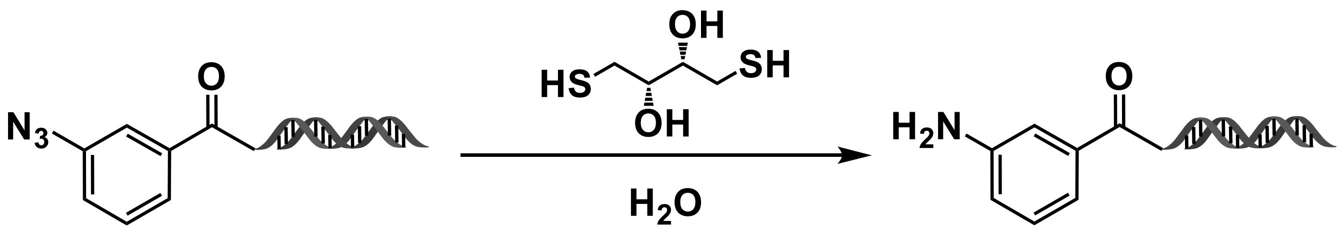

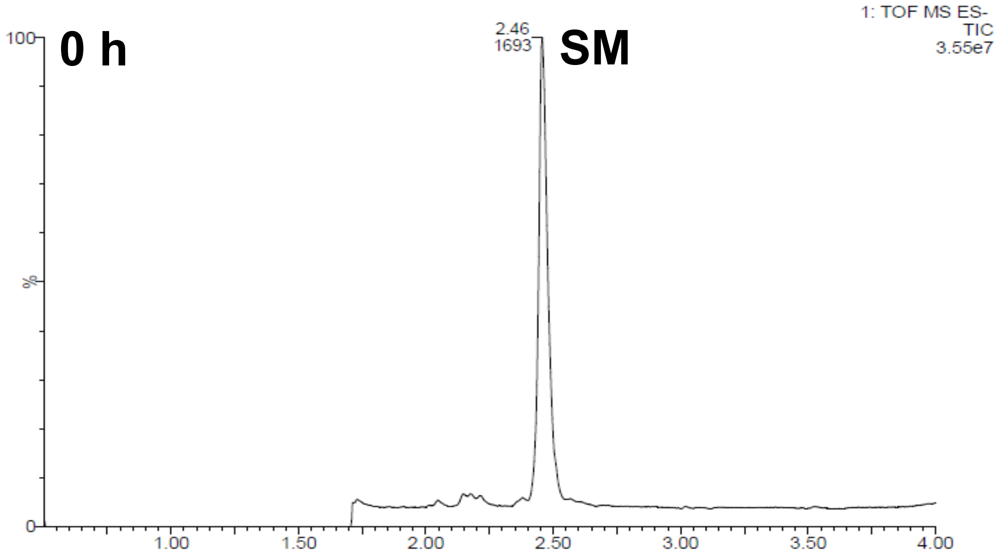

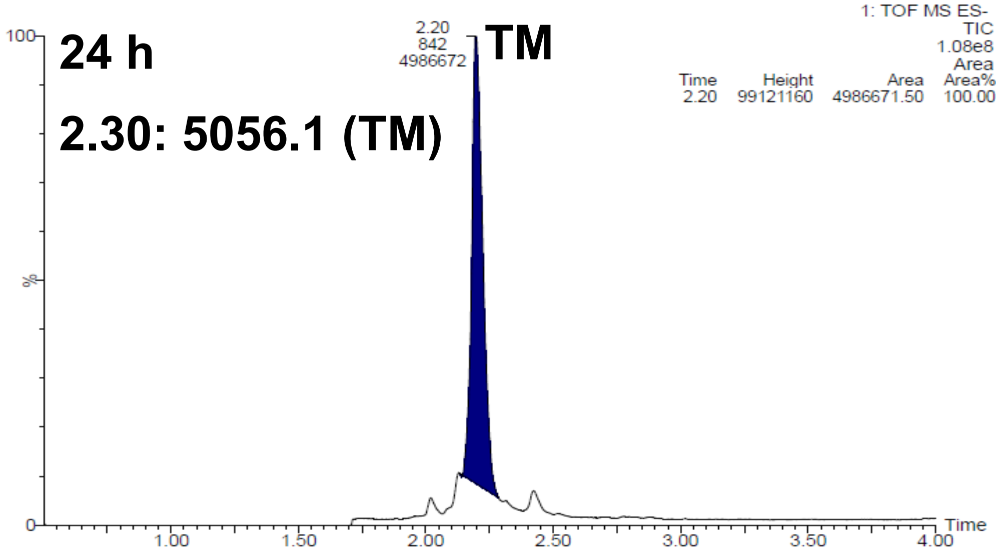

Reduction of 9c with DTT.

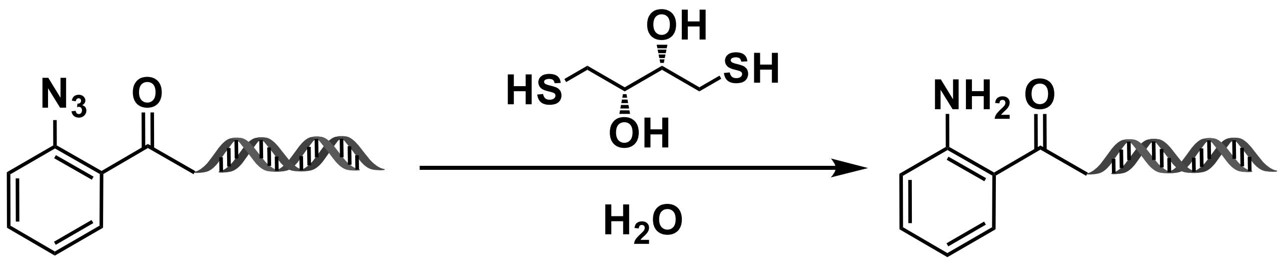

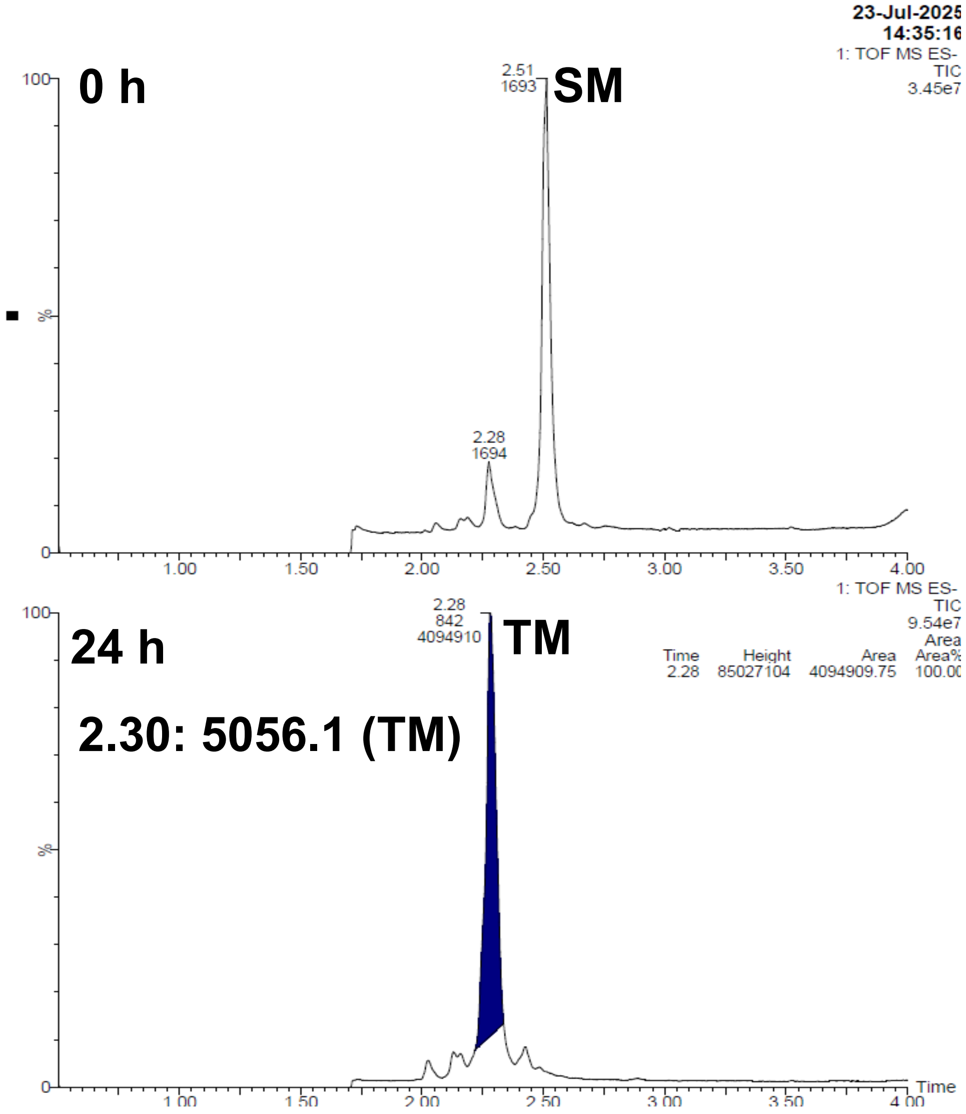

Reduction of 9d with DTT.

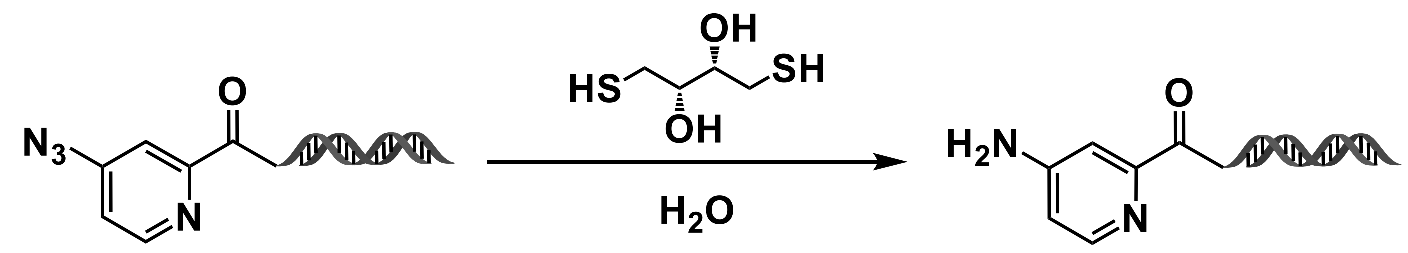

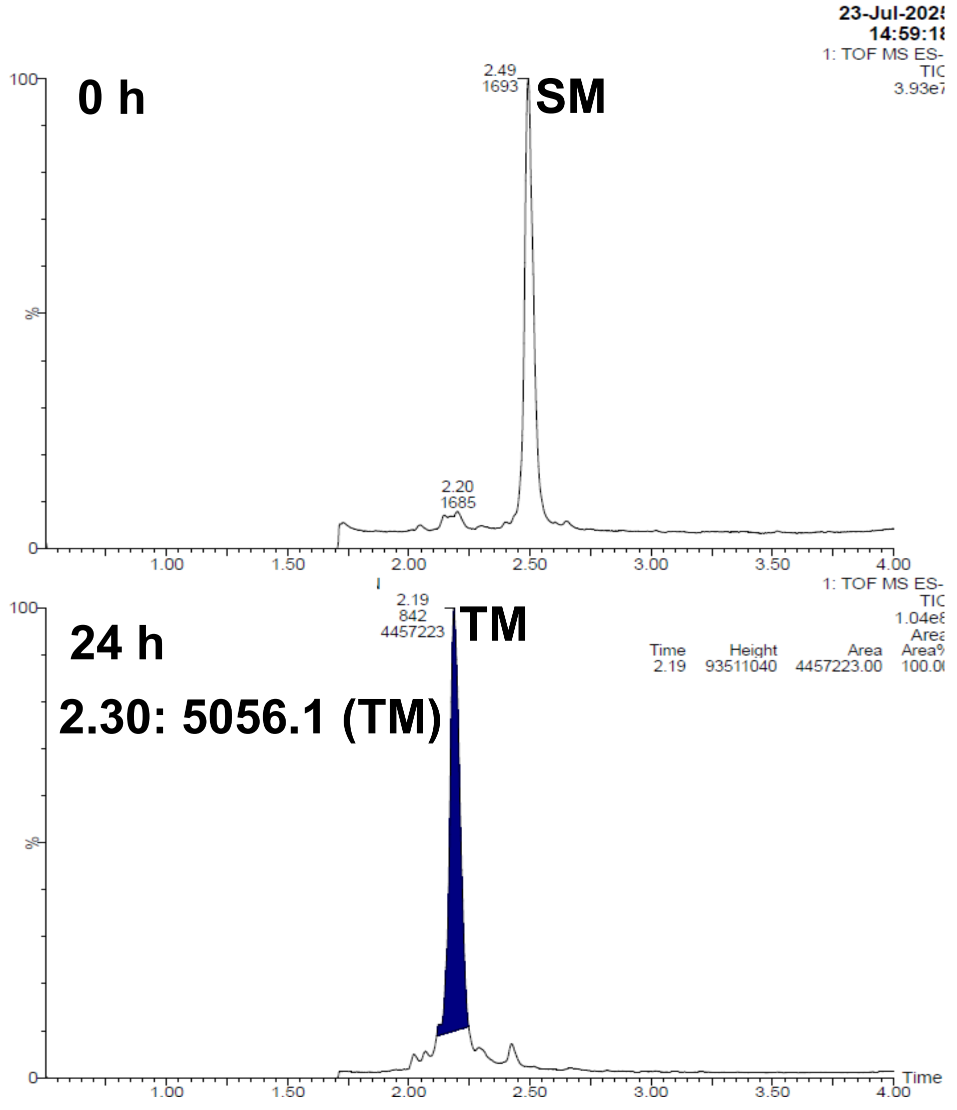

Reduction of 9e with DTT.

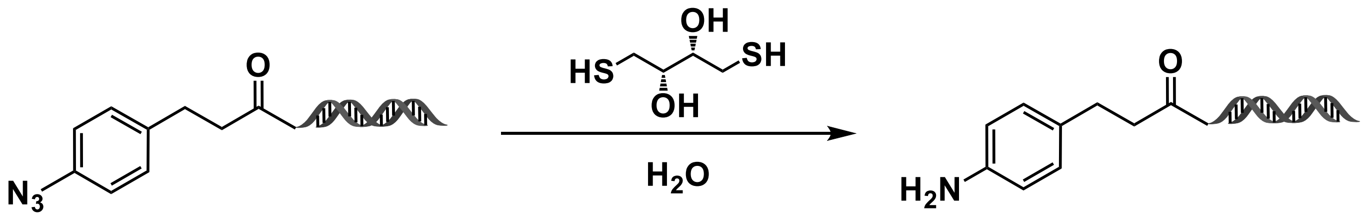

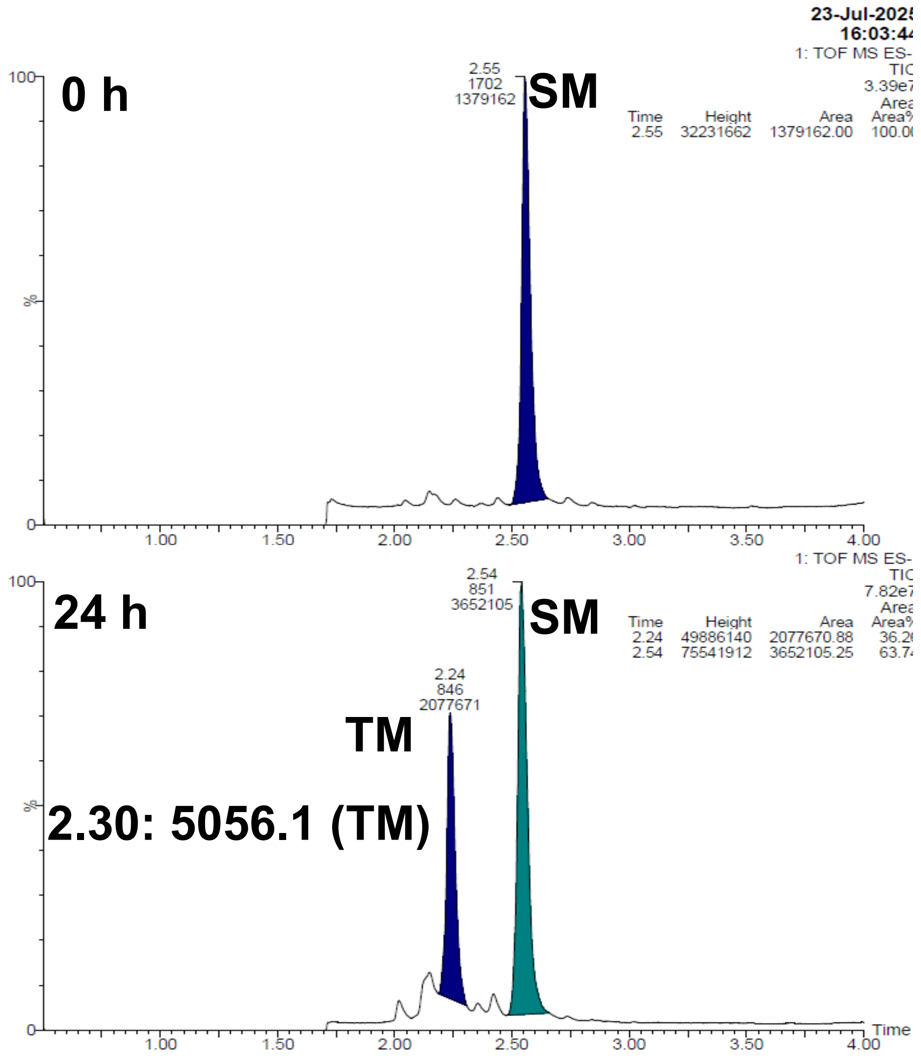

Reduction of 9f with DTT.

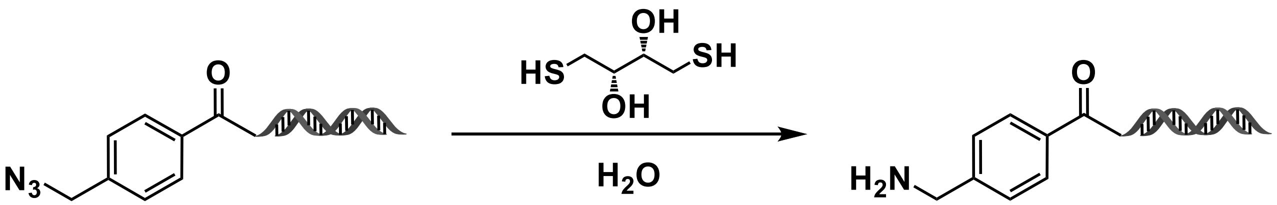

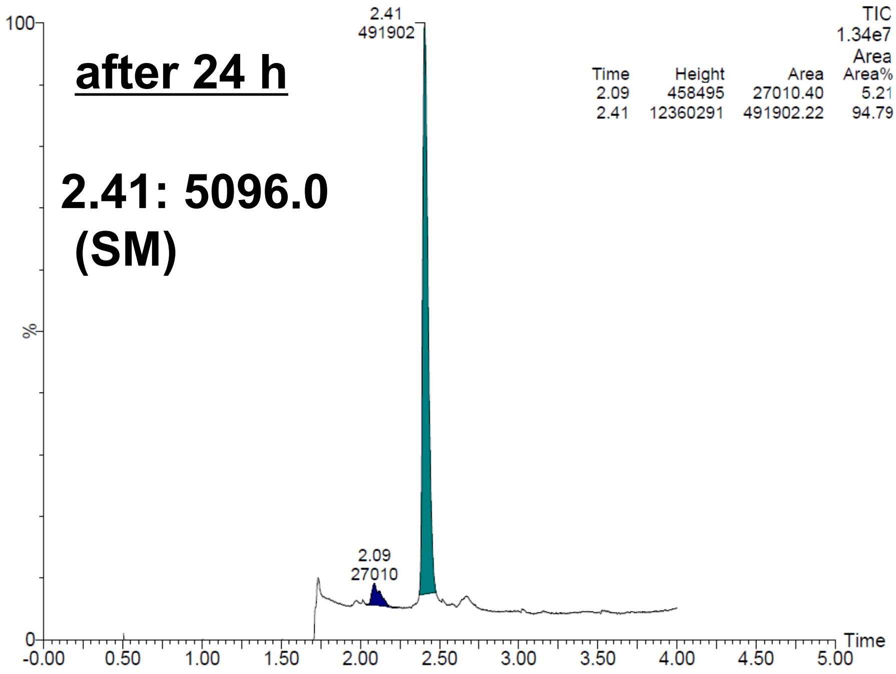

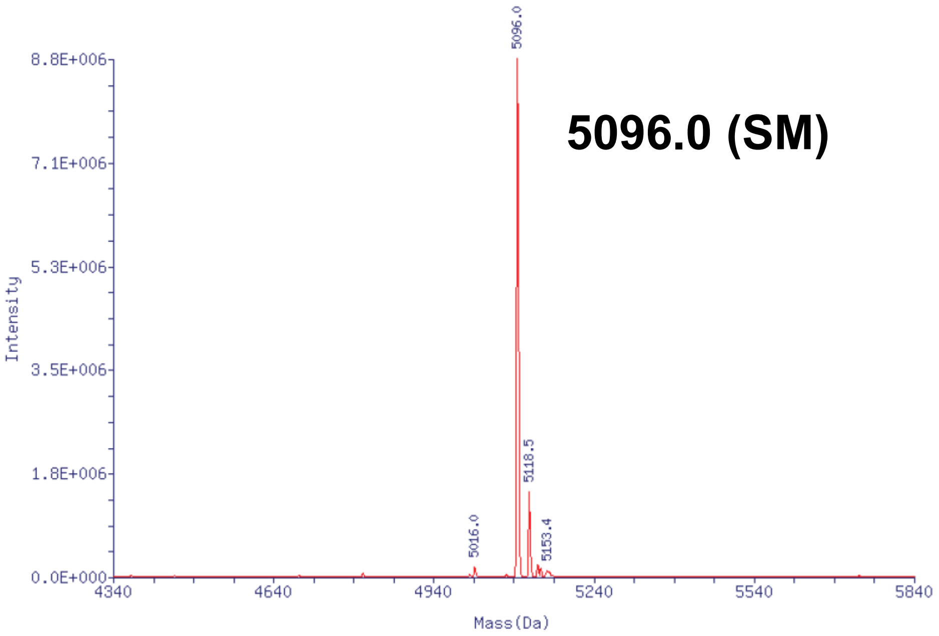

Reduction of 9g with DTT.

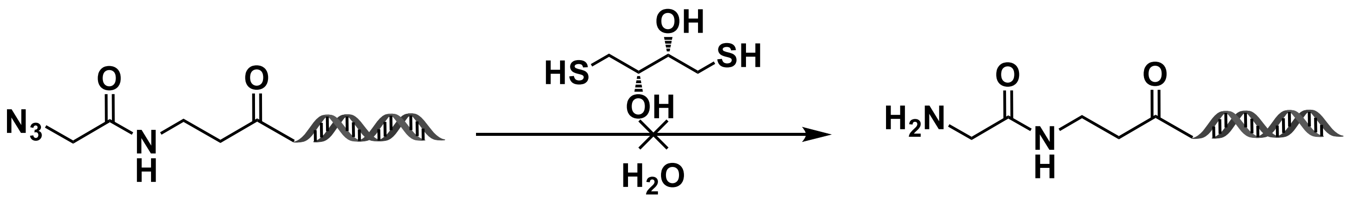

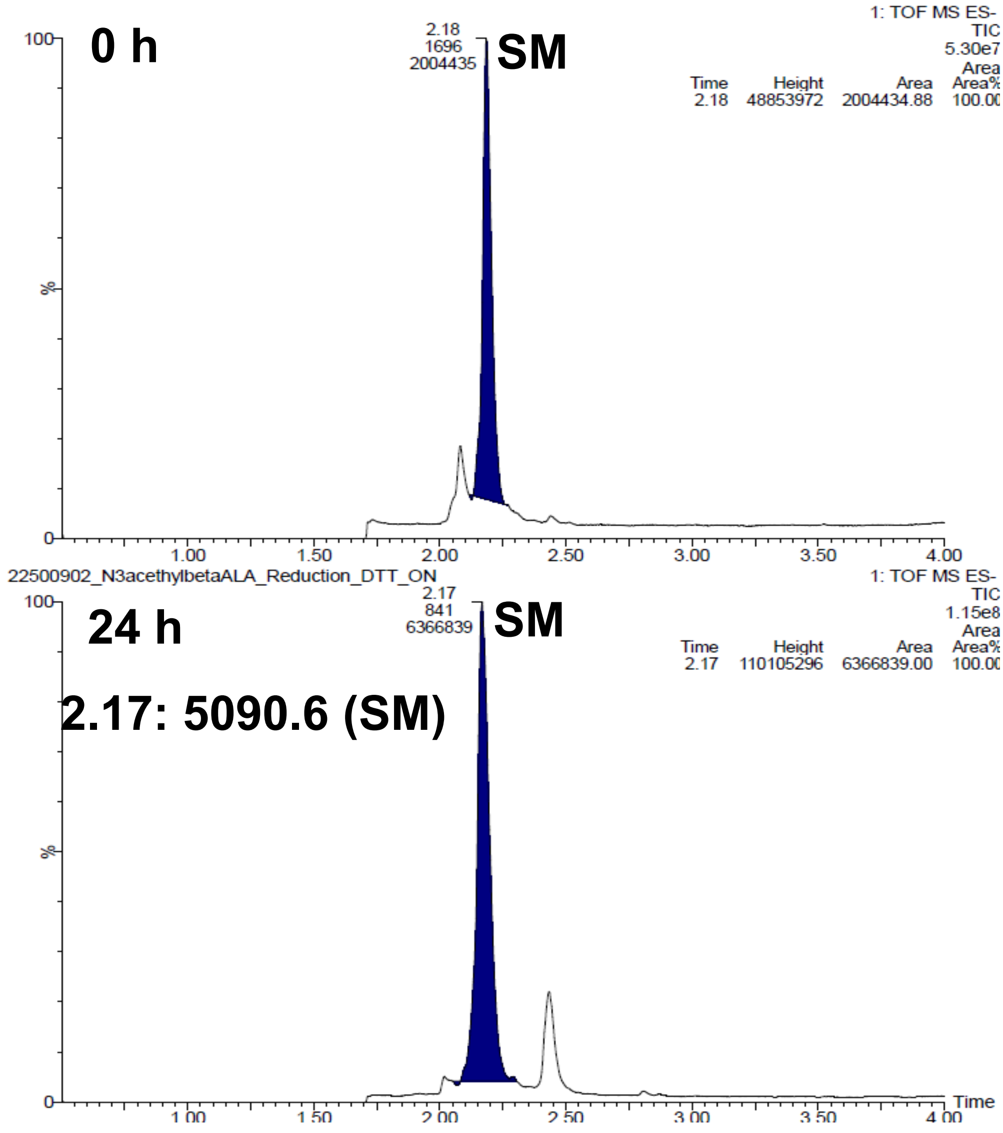

Reduction of 9h with DTT.

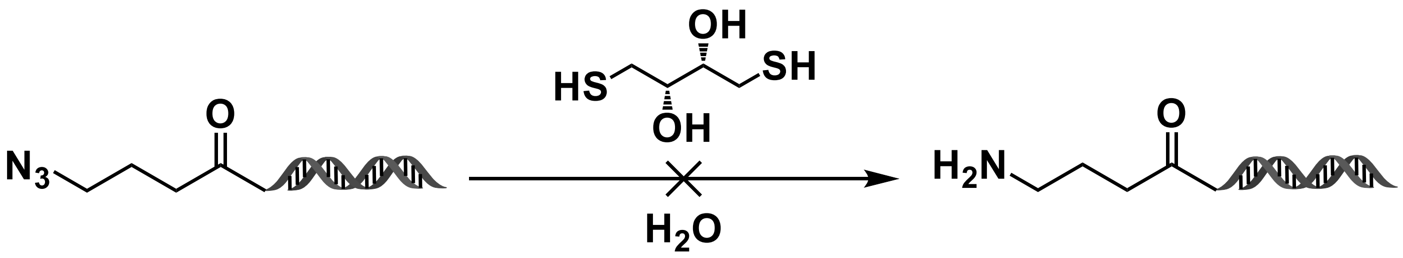

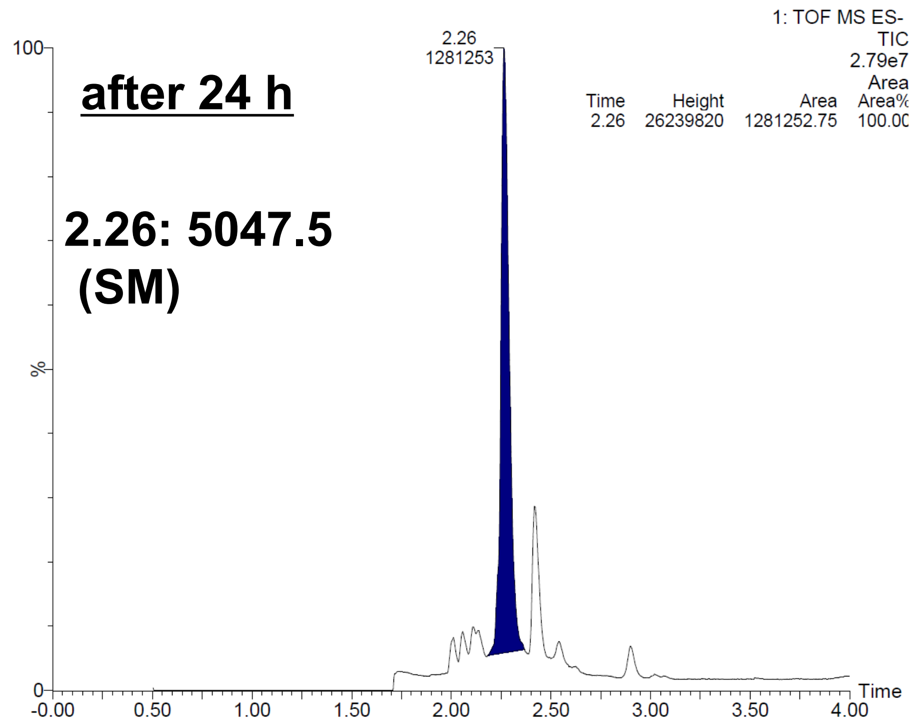

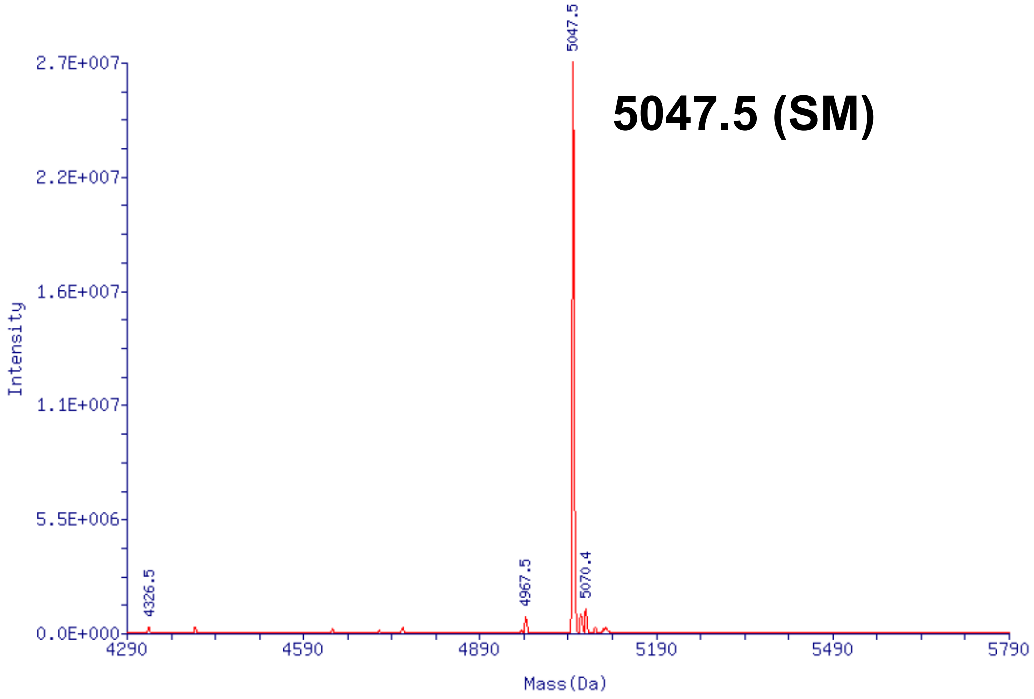

Reduction of 9i with DTT.

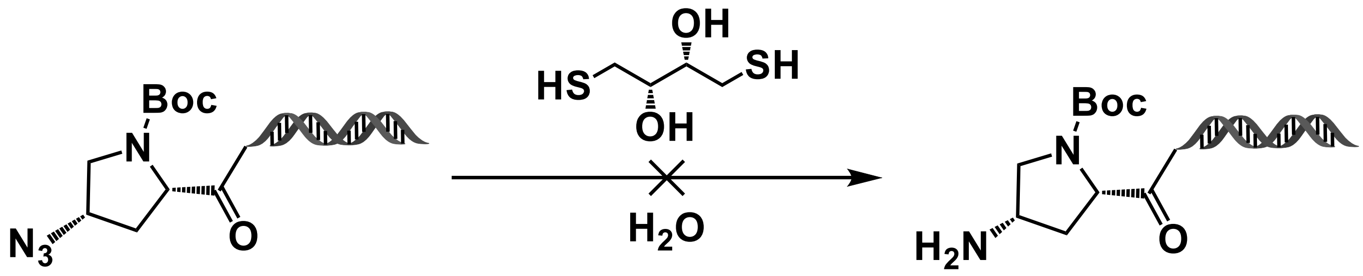

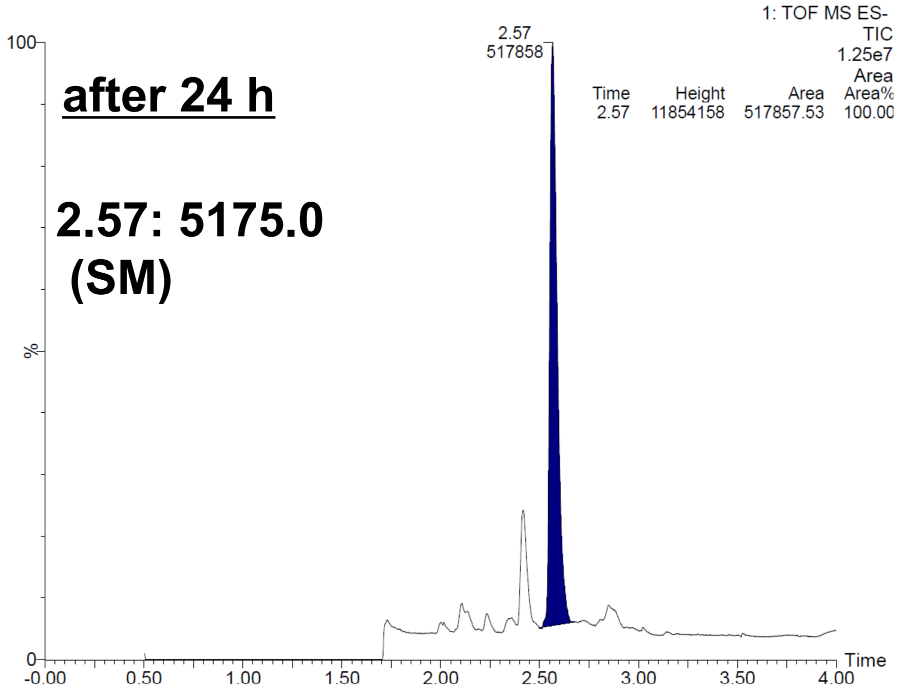

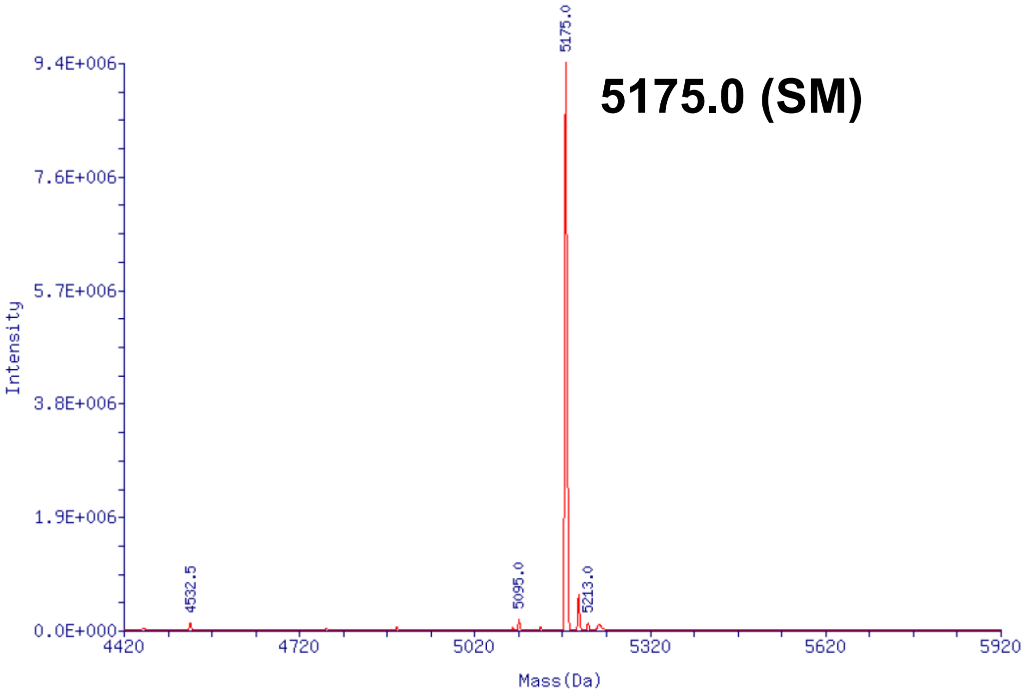

Reduction of 9a with TPPTS.

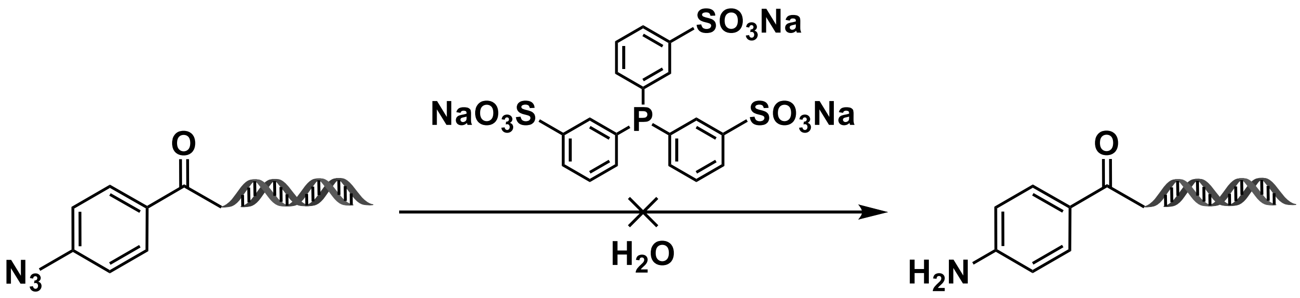

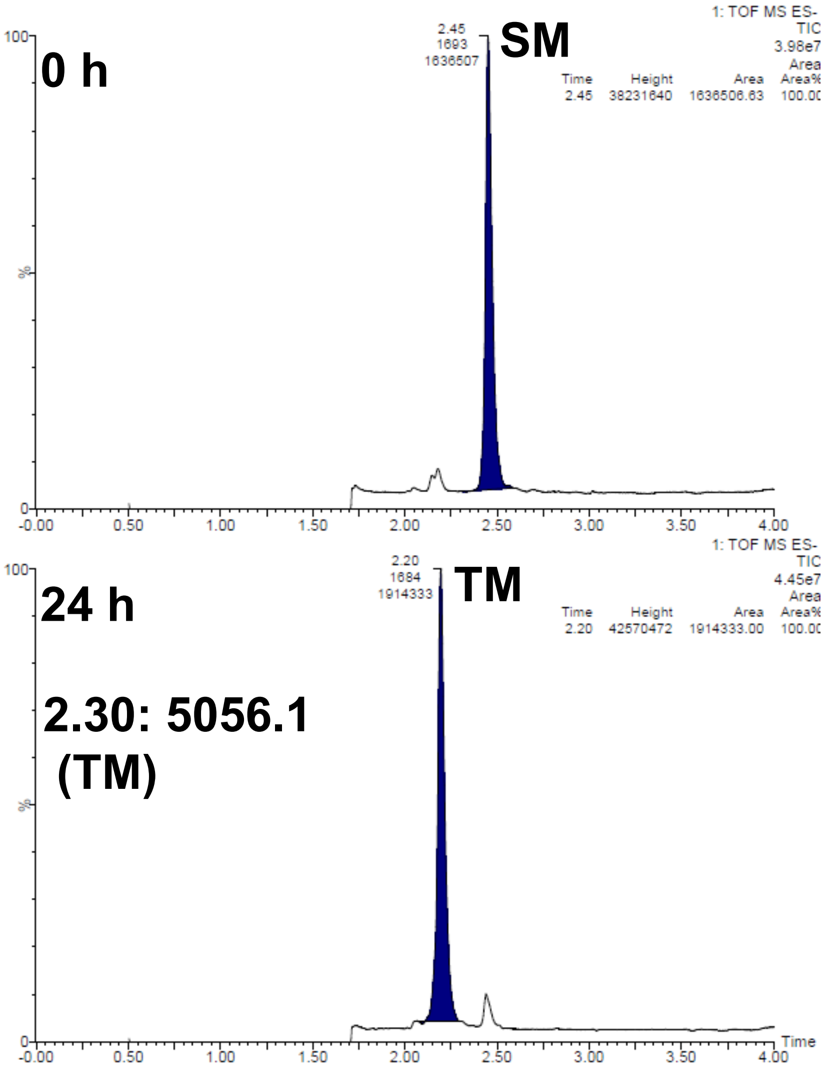

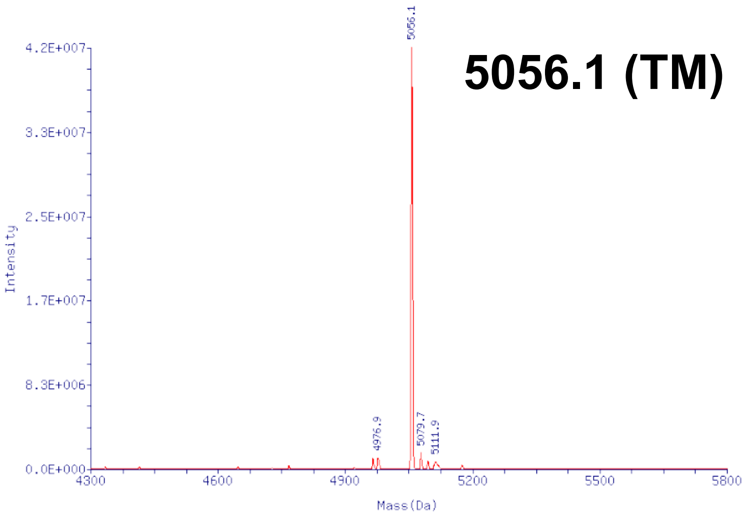

Reduction of 9b with TPPTS.

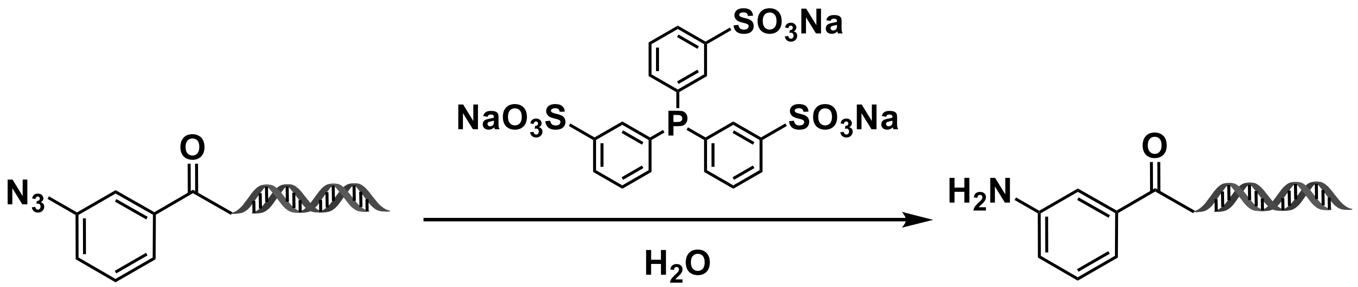

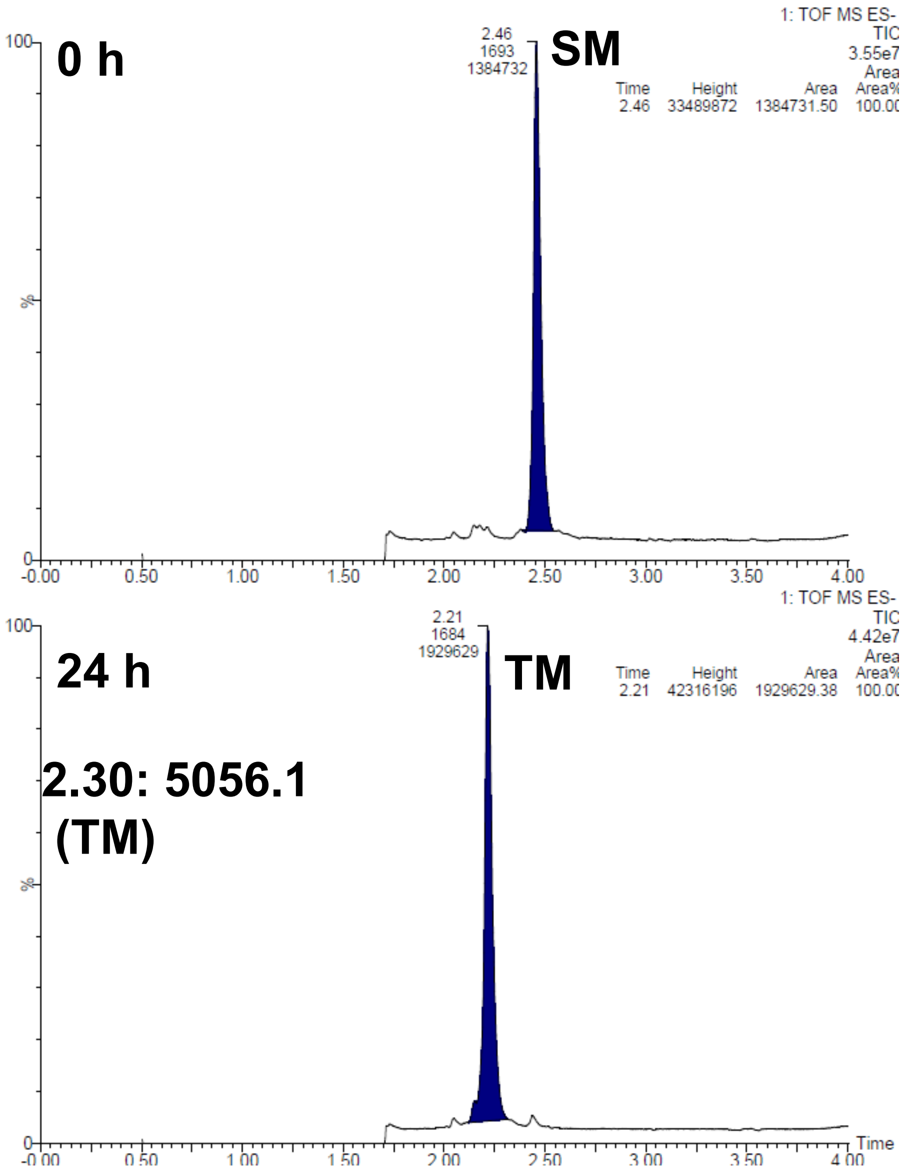

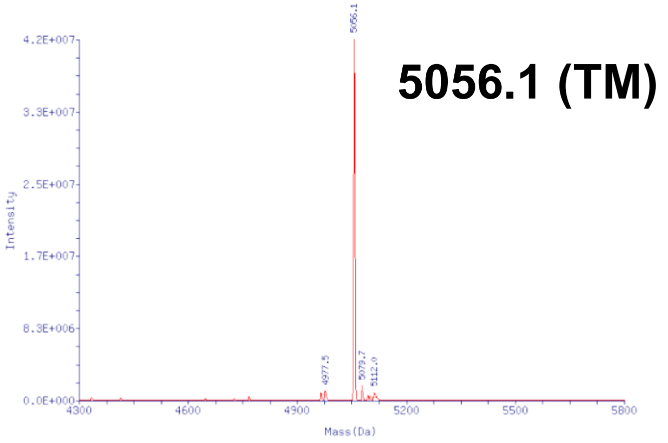

Reduction of 9c with TPPTS.

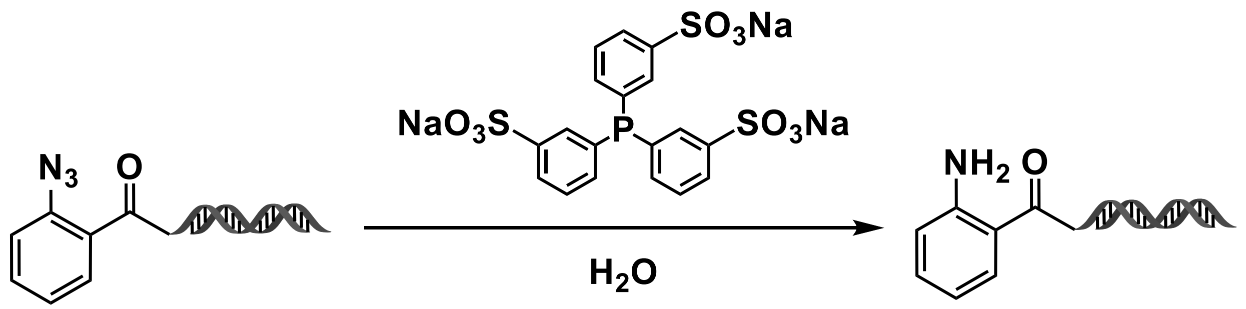

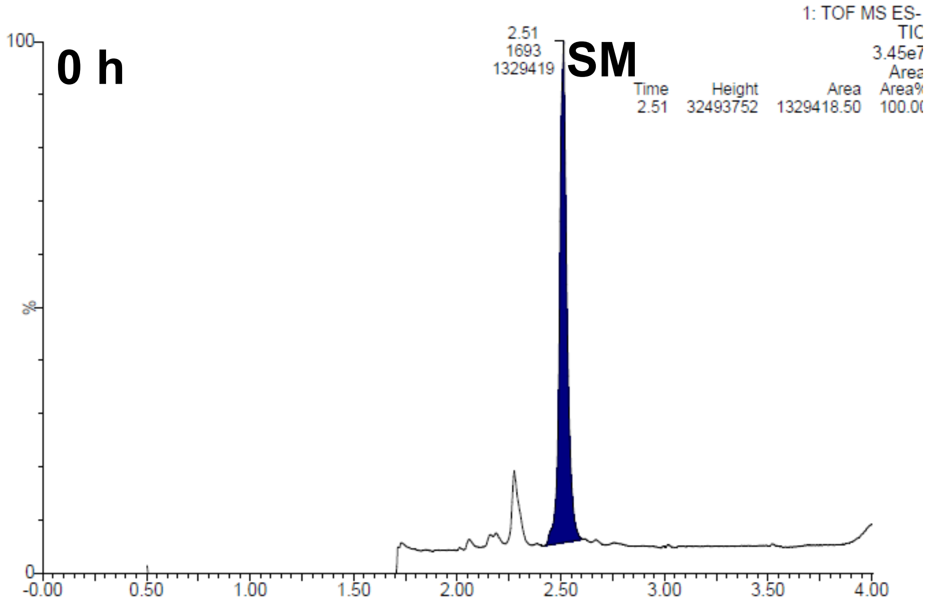

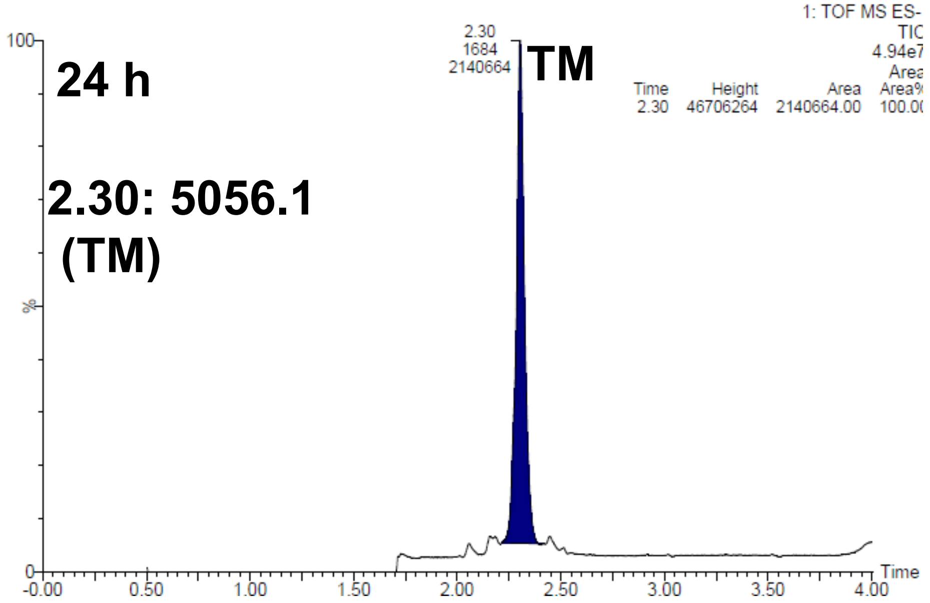

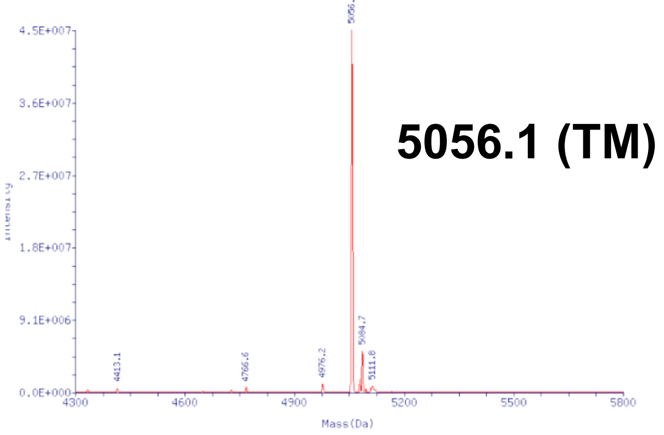

Reduction of 9d with TPPTS.

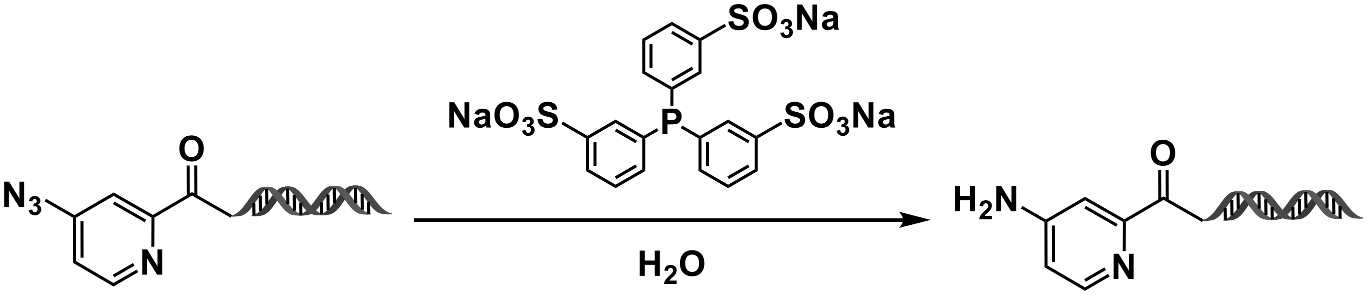

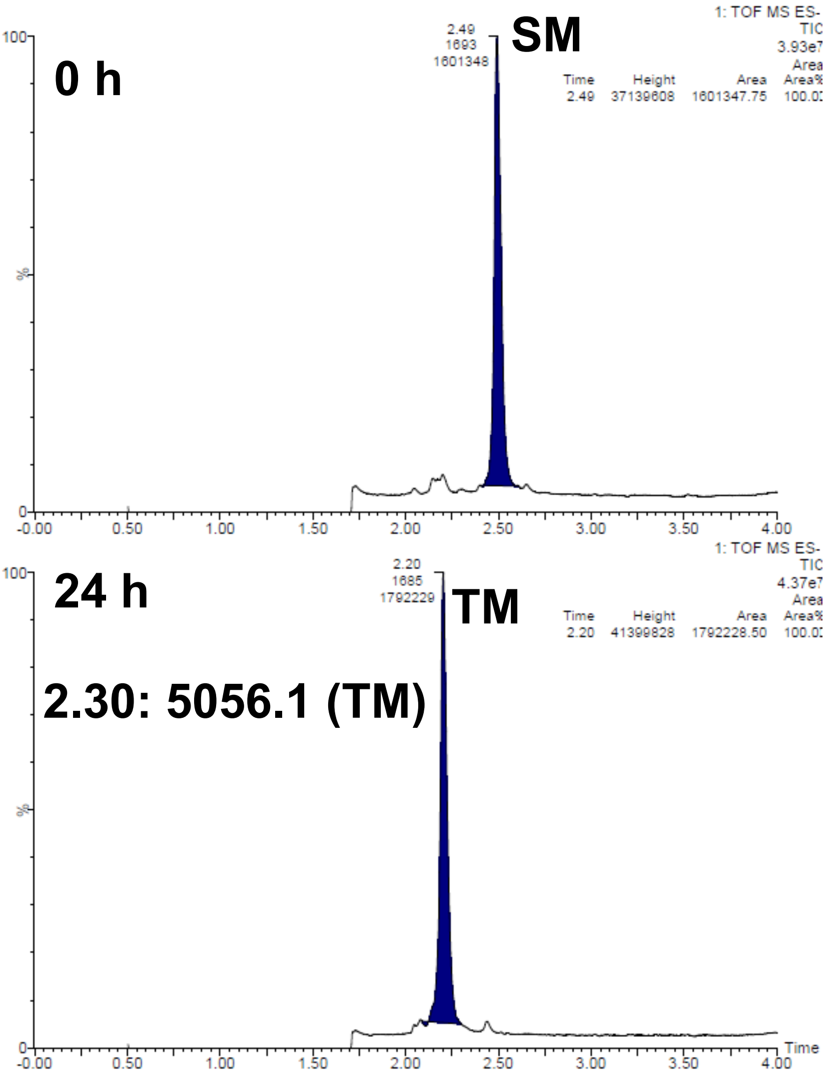

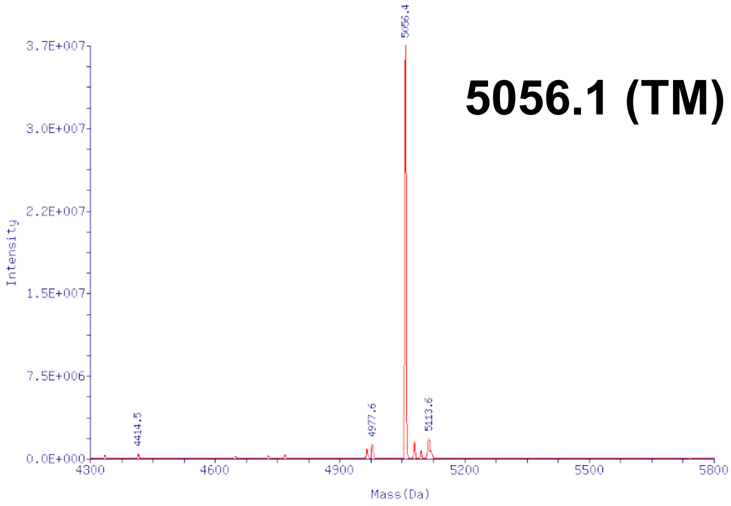

Reduction of 9e with TPPTS.

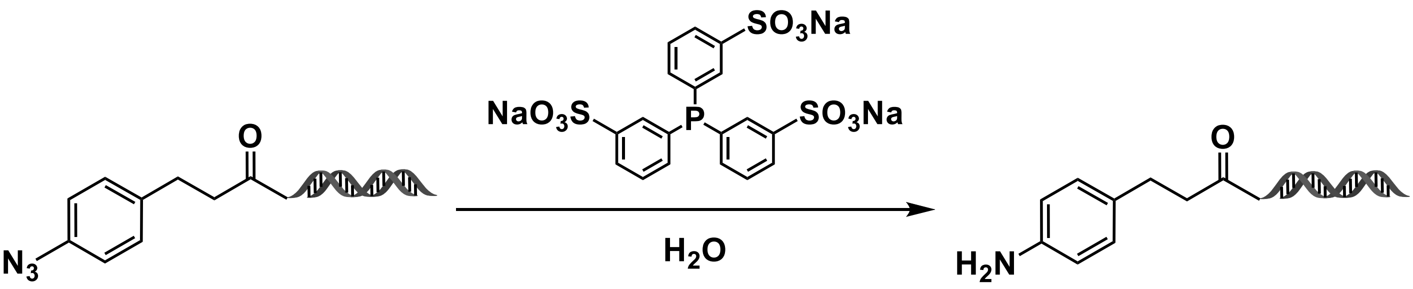

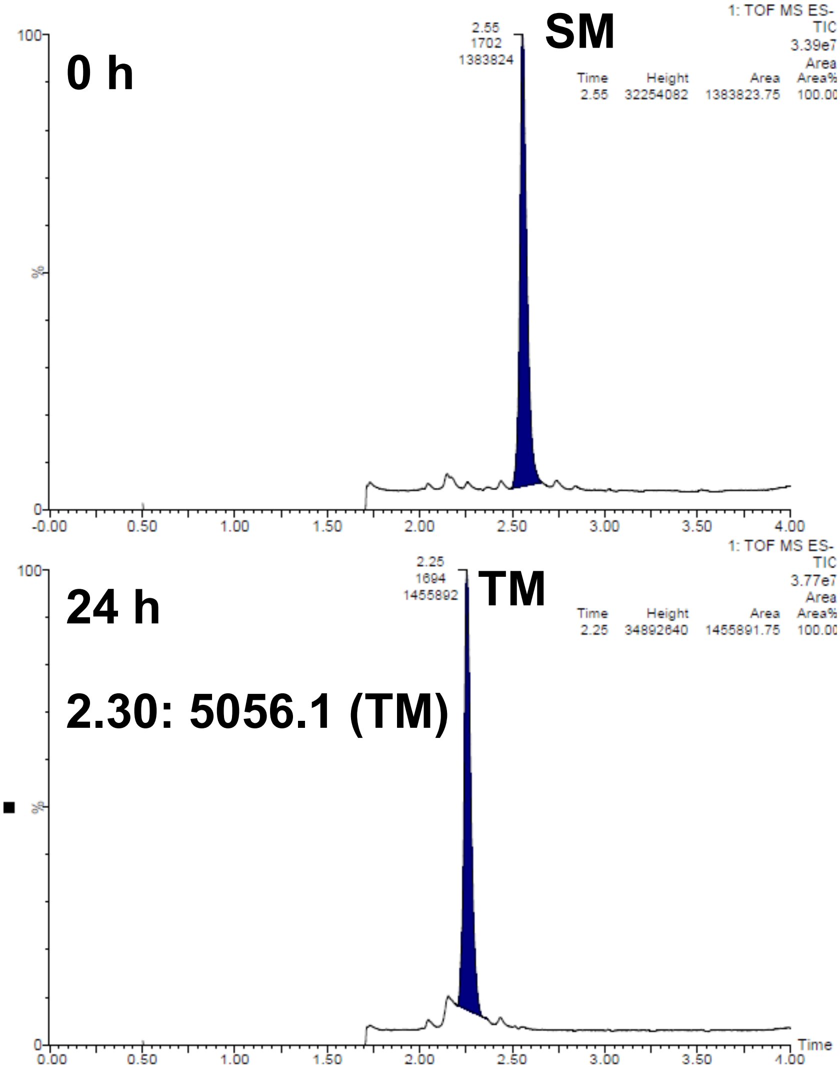

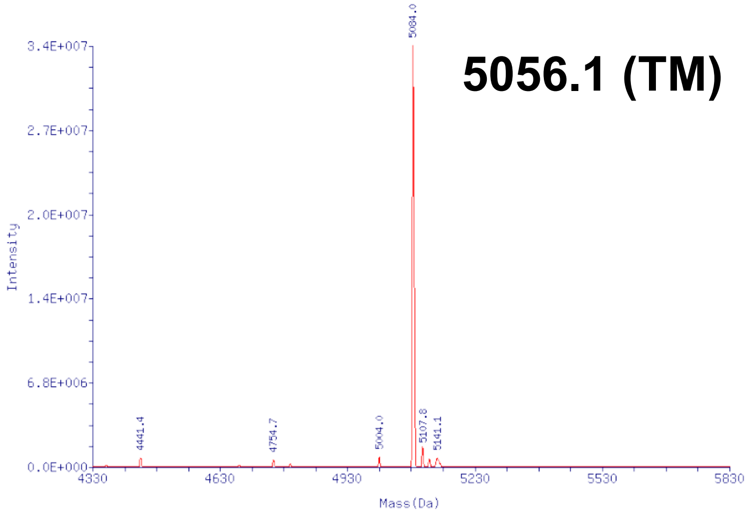

Reduction of 9f with TPPTS.

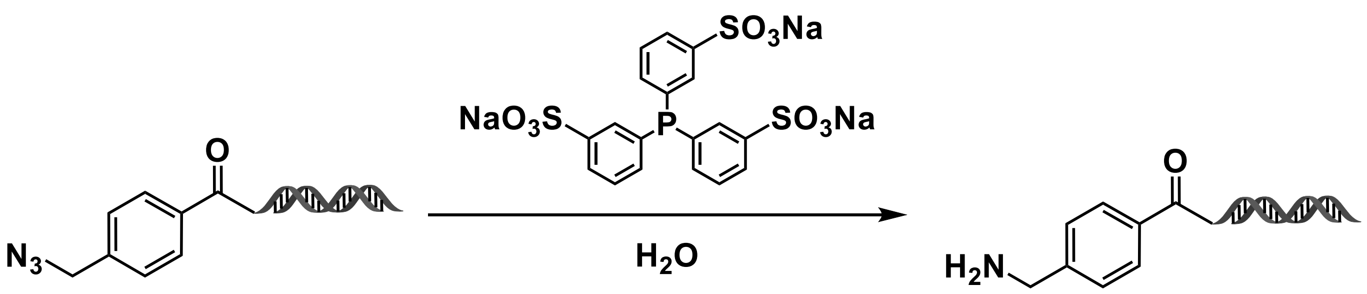

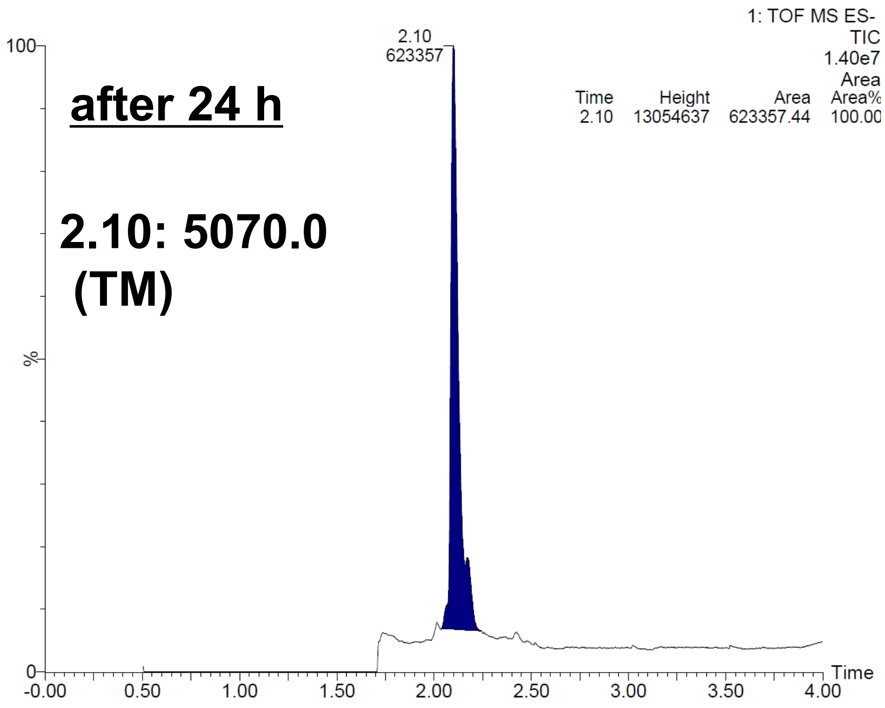

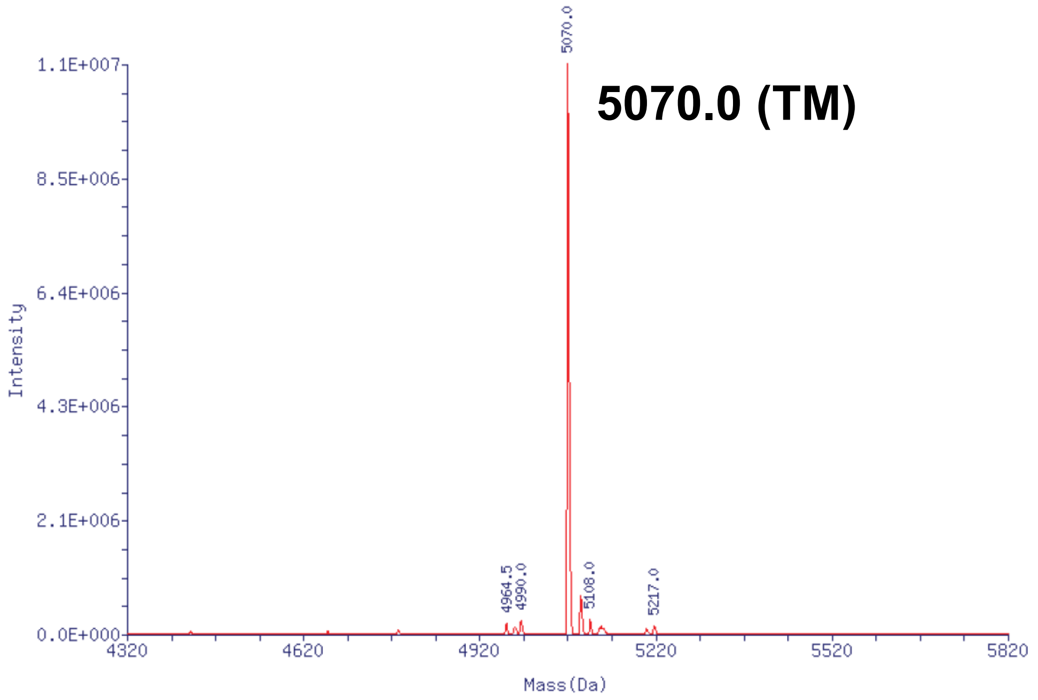

Reduction of 9g with TPPTS.

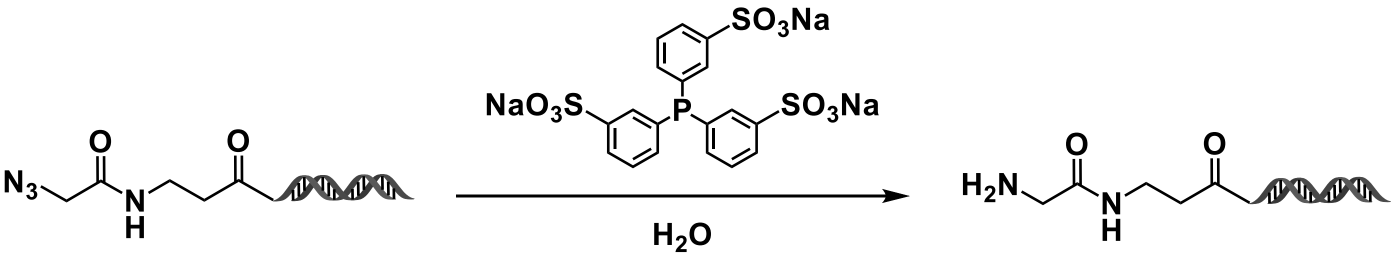

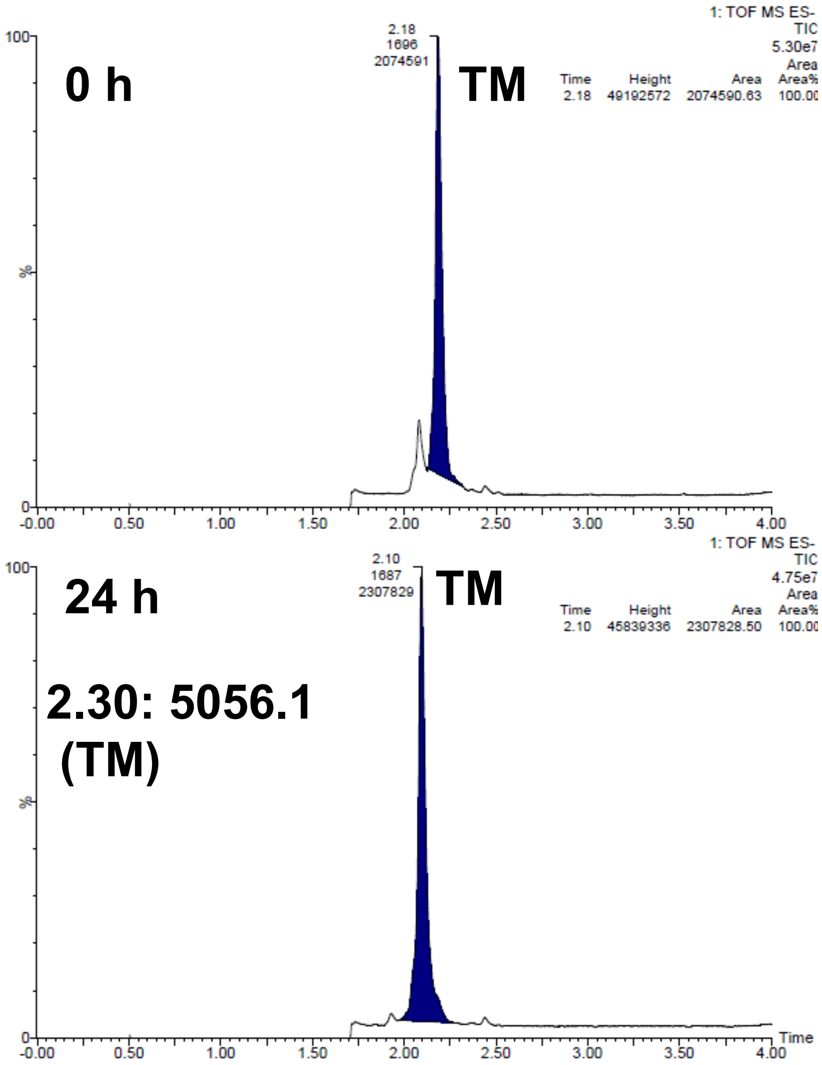

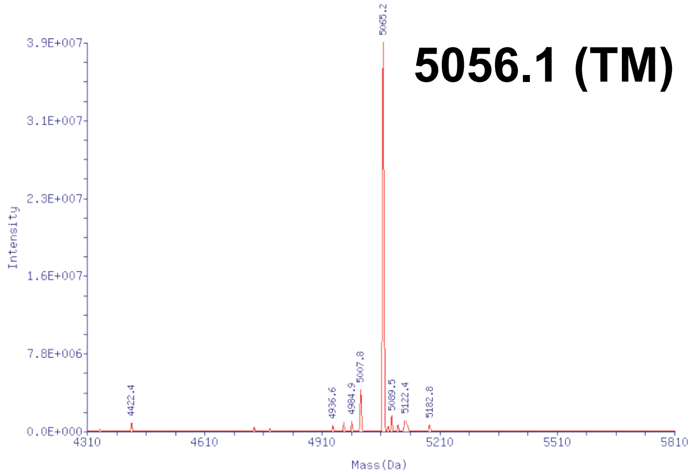

Reduction of 9h with TPPTS.

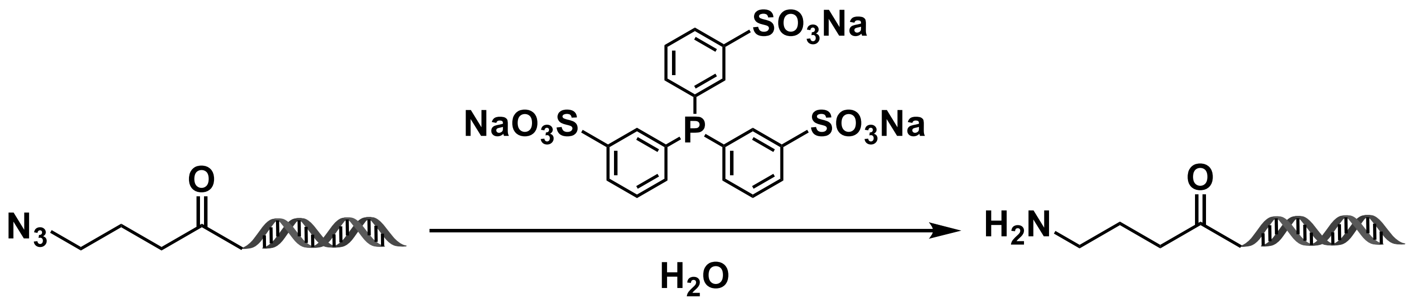

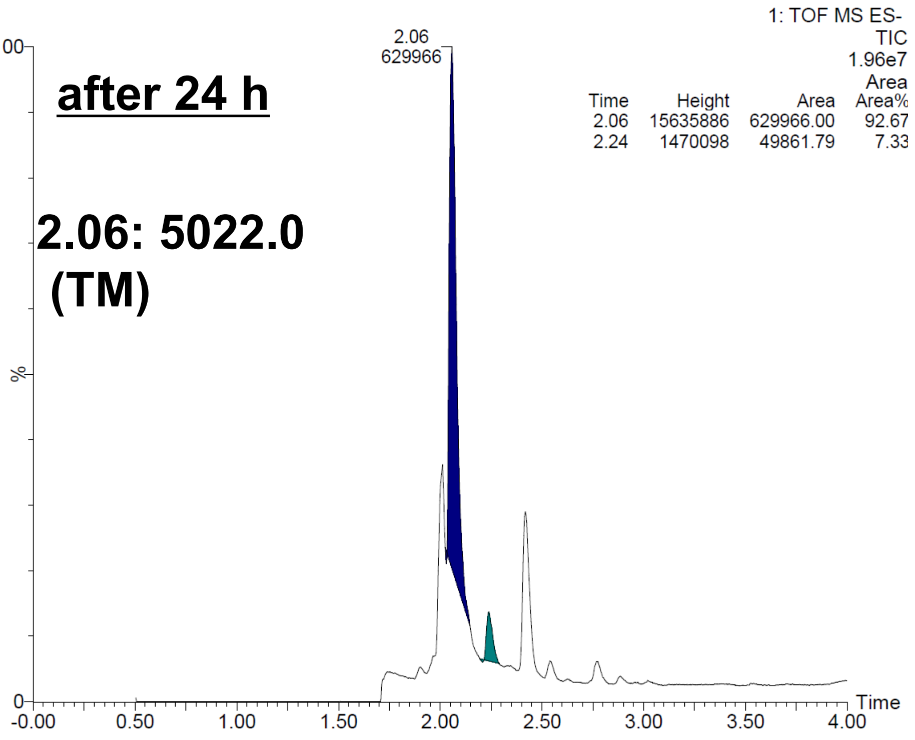

Time	Height	Area	Area%
2.06	15635886	629966.00	92.67
2.24	1470098	49861.79	7.33

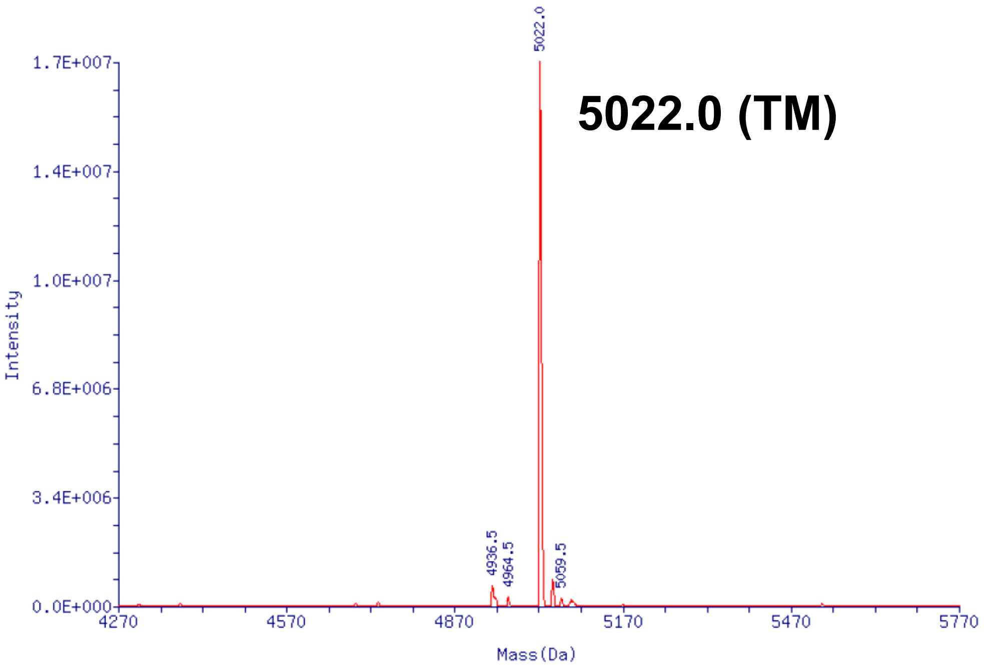

Reduction of 9i with TPPTS.

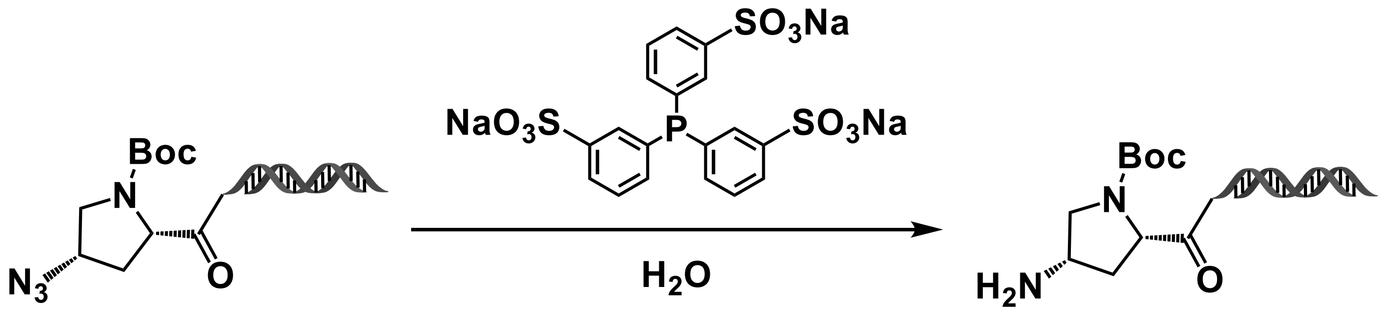

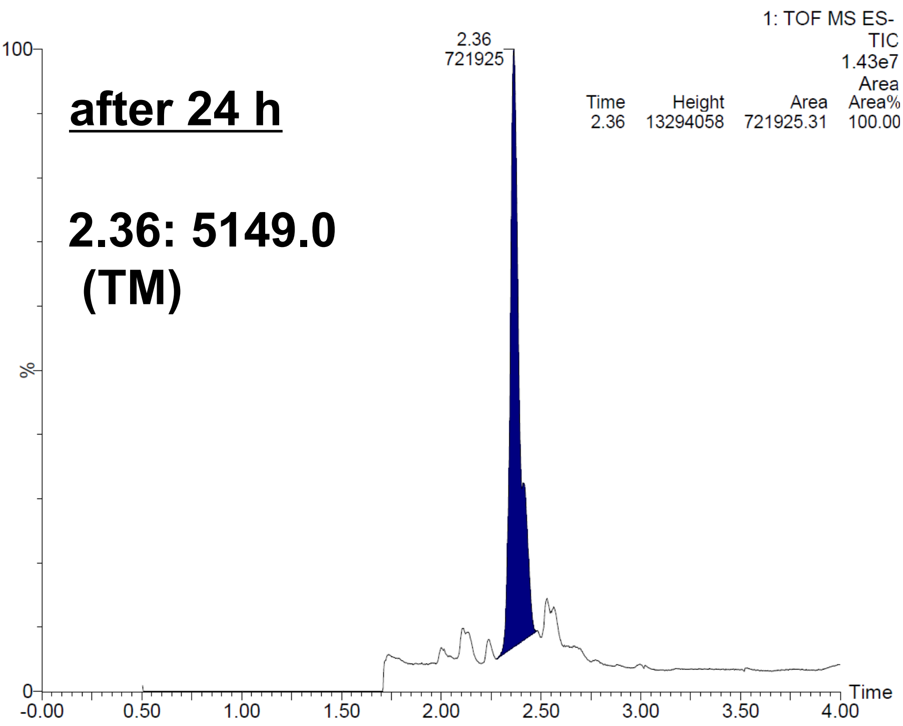

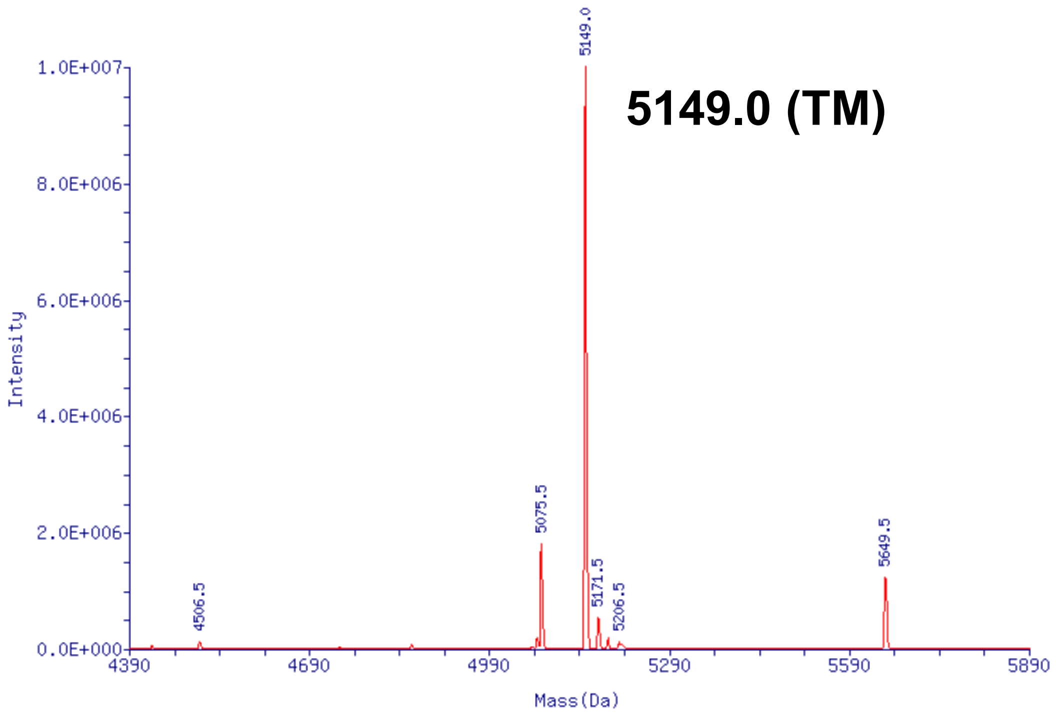

Reduction of 9a with 2-ME.

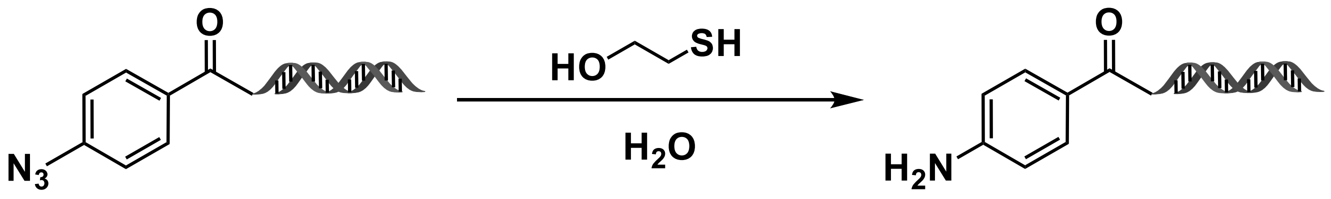

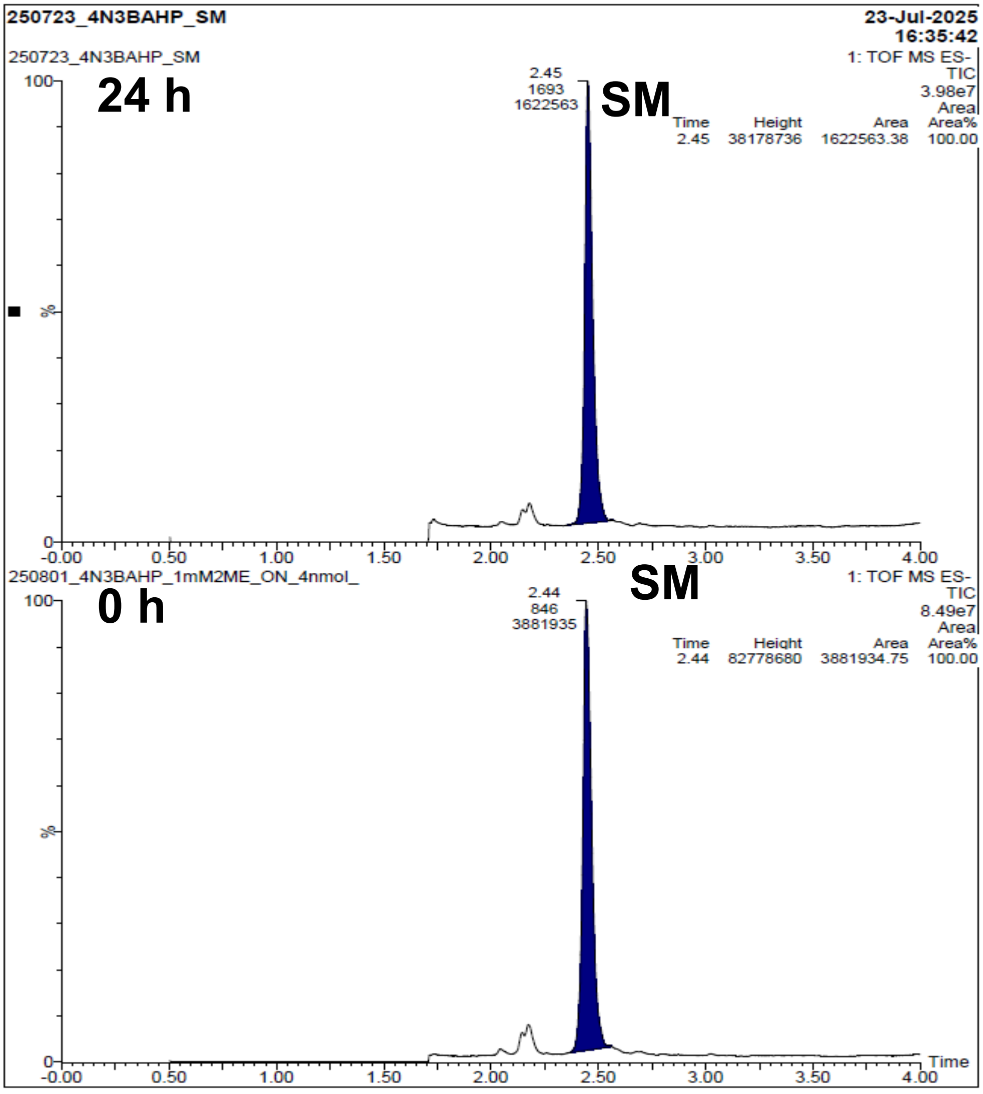

Reduction of 9b with 2-ME.

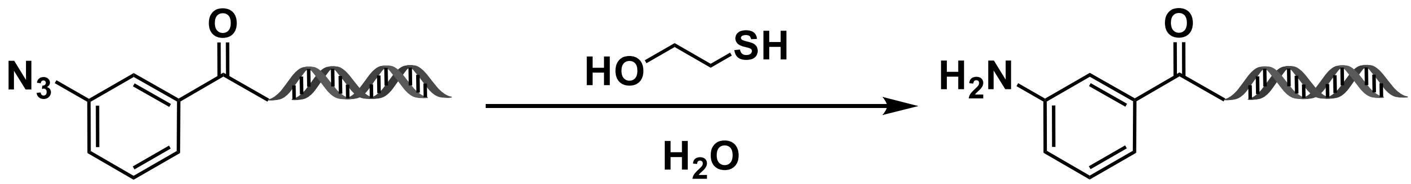

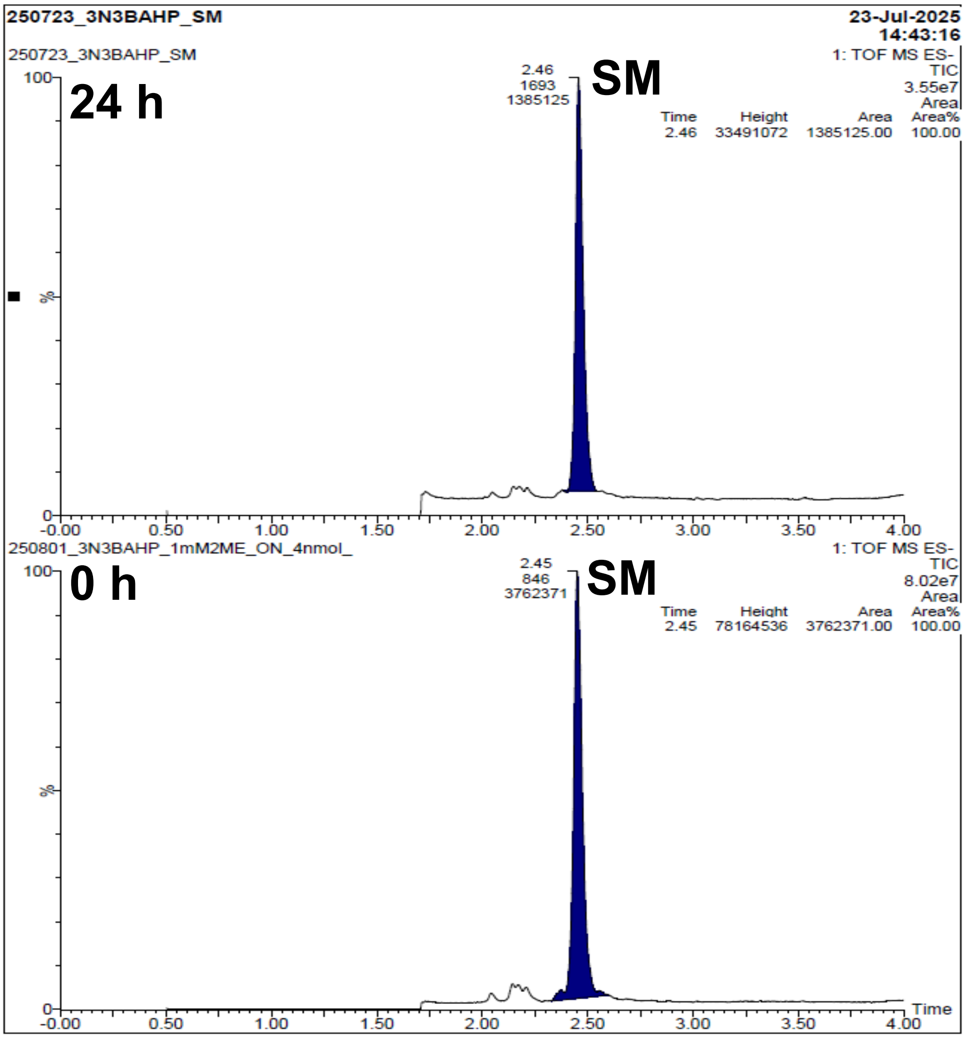

Reduction of 9c with 2-ME.

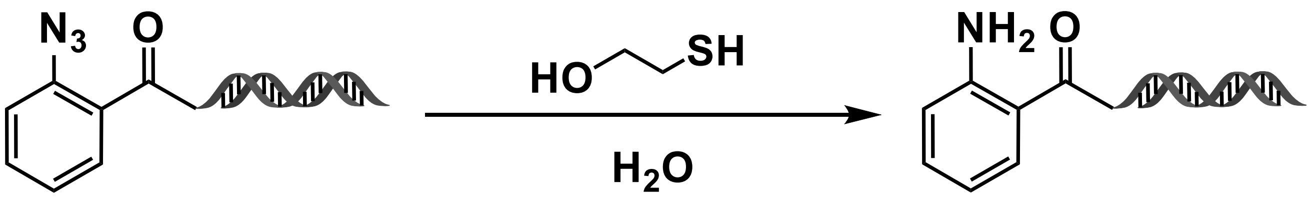

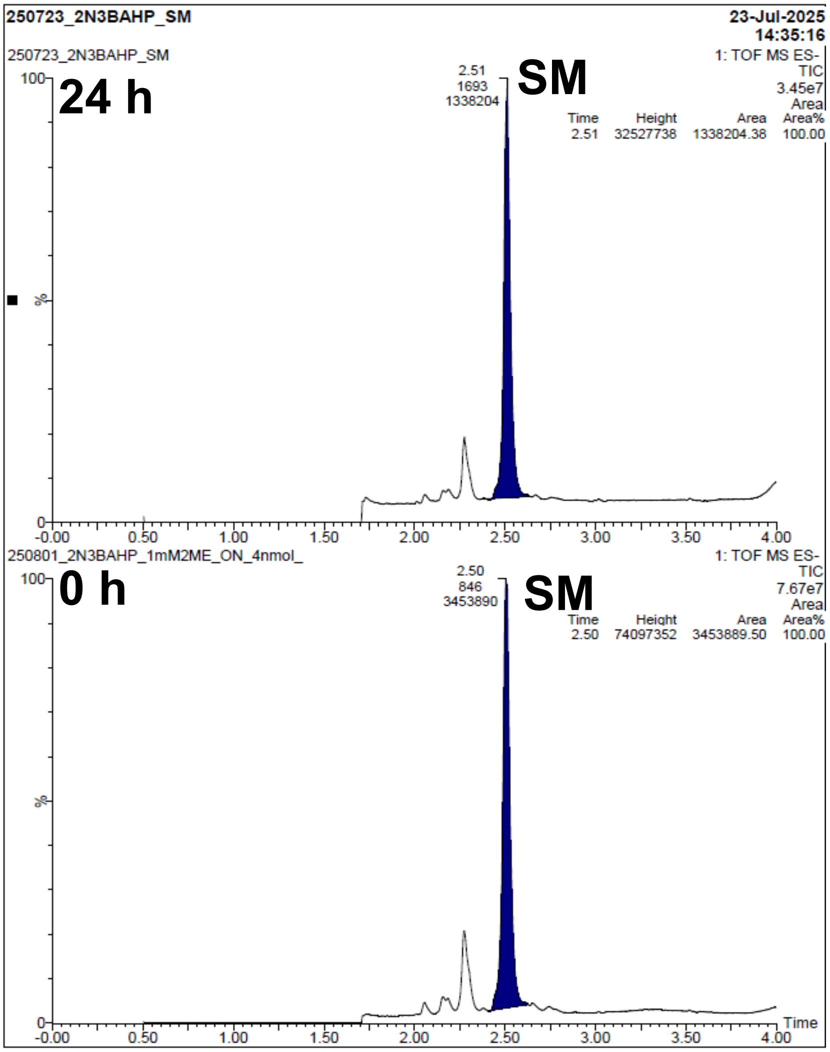

Reduction of 9d with 2-ME.

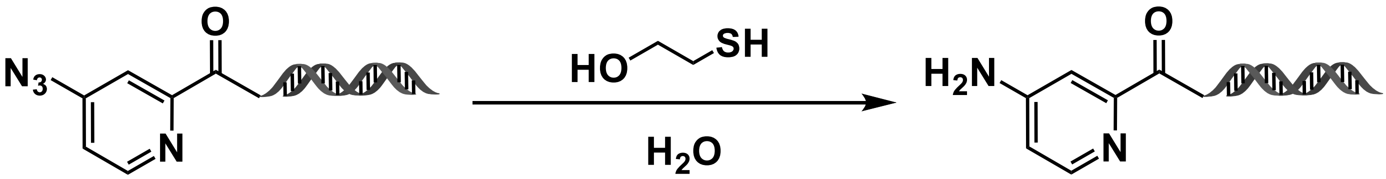

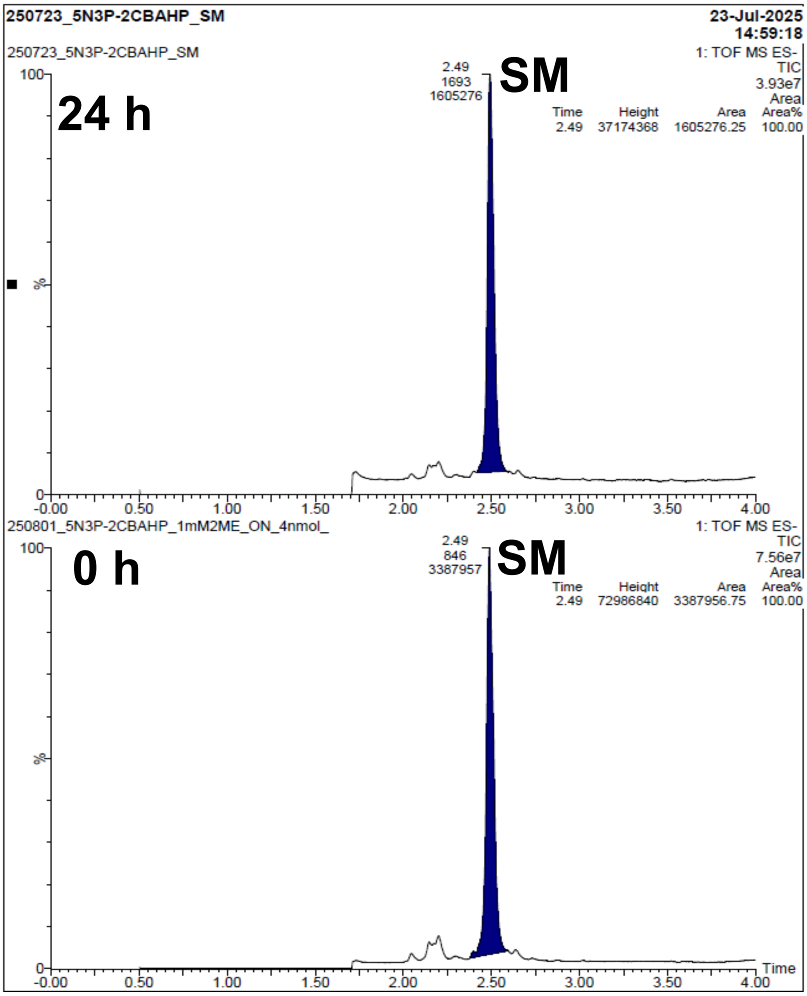

Reduction of 9e with 2-ME.

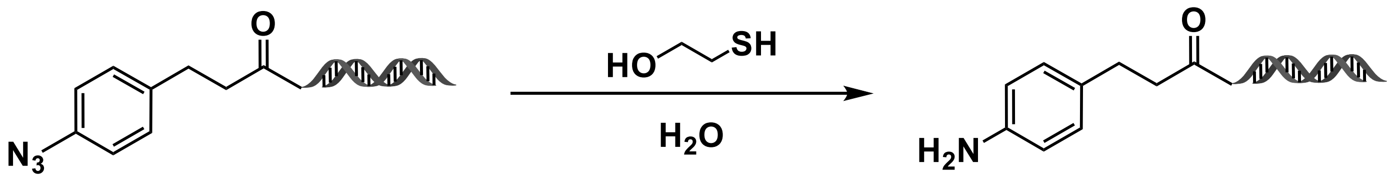

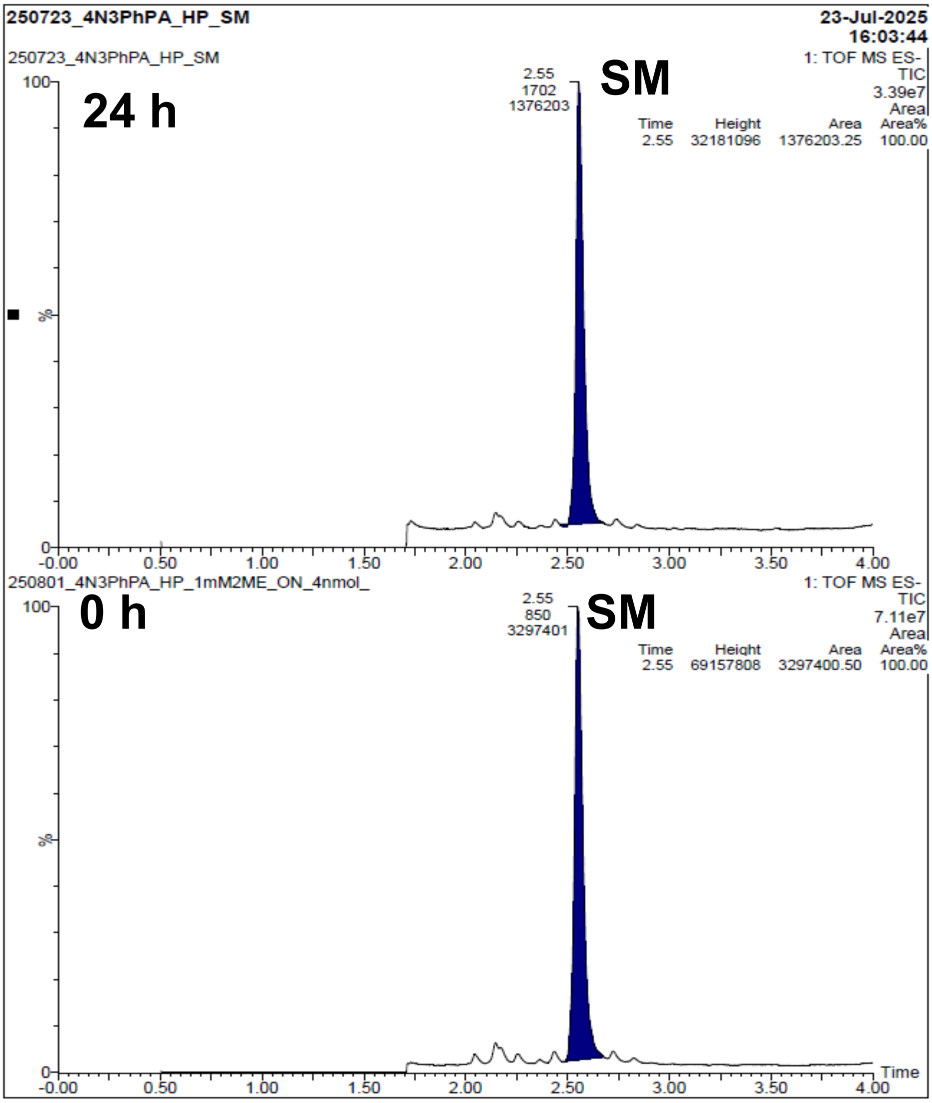

Reduction of 9f with 2-ME.

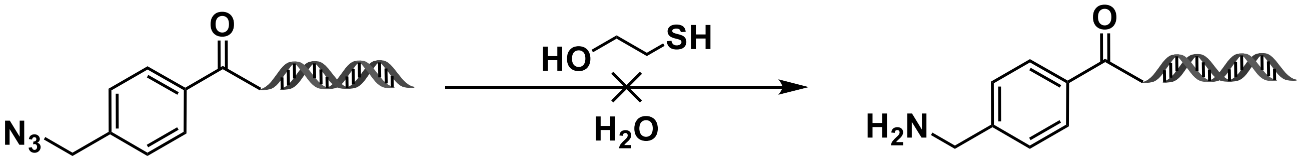

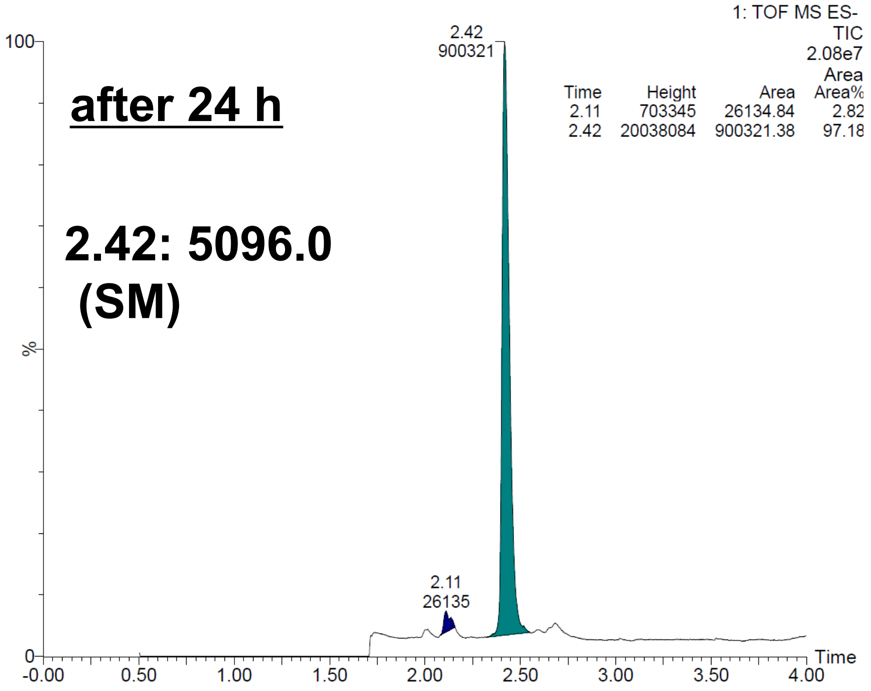

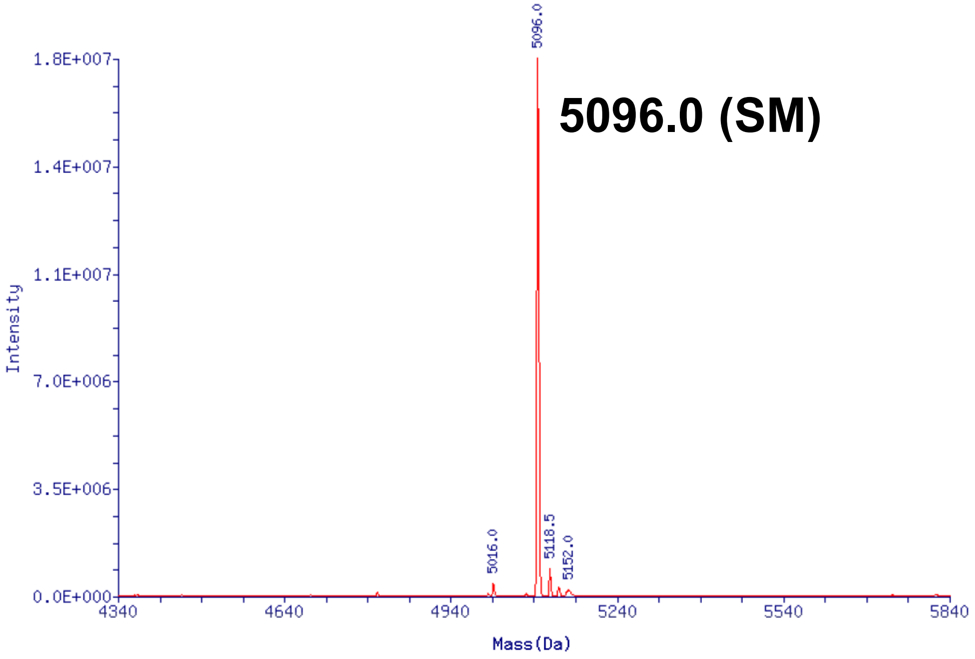

Reduction of 9g with 2-ME.

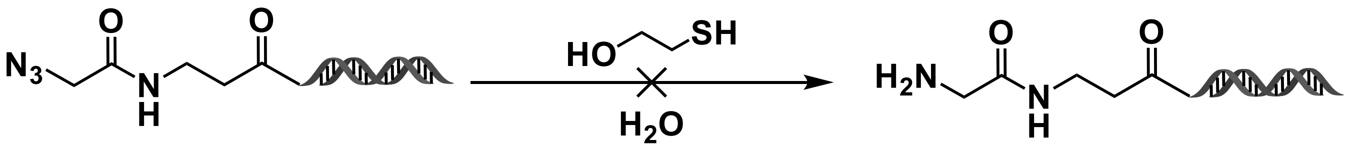

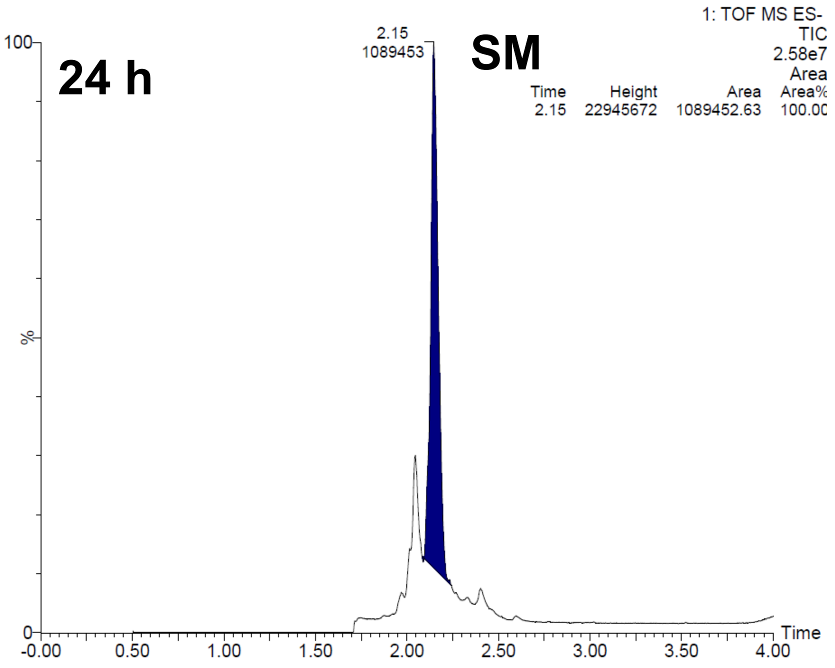

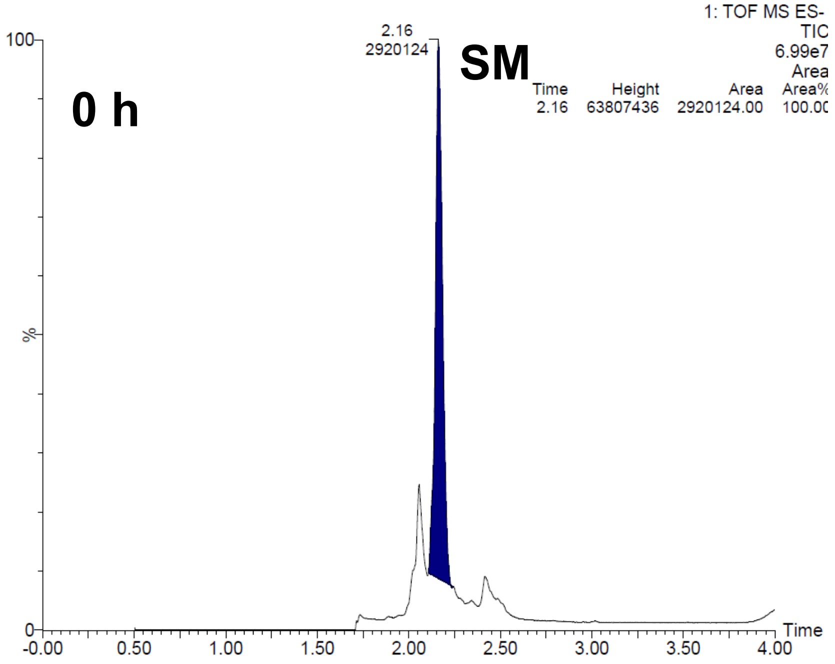

Reduction of 9h with 2-ME.

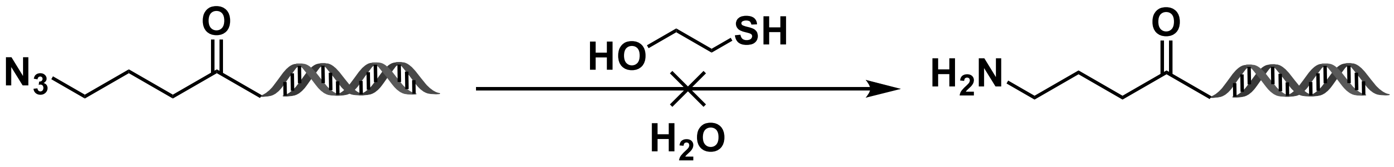

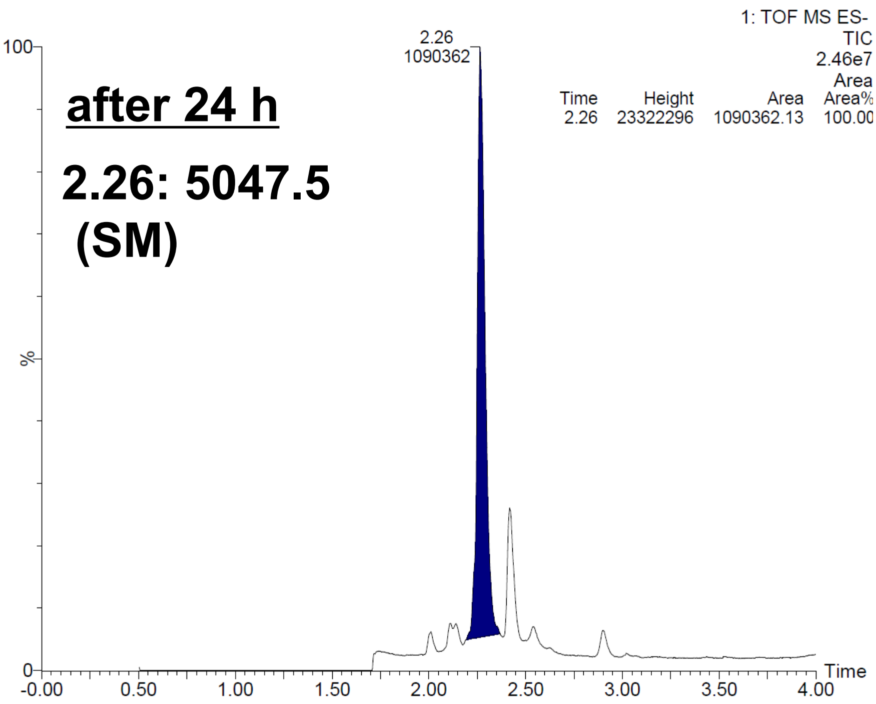

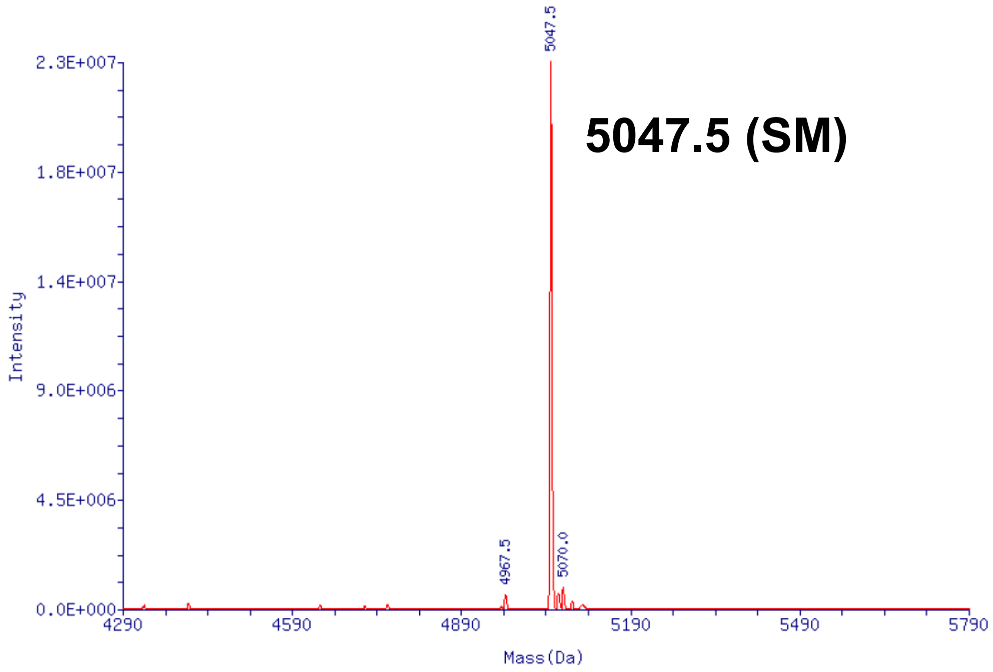

Reduction of 9i with 2-ME.

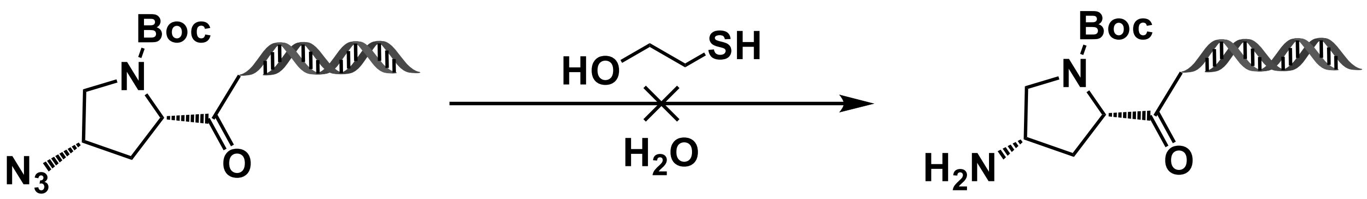

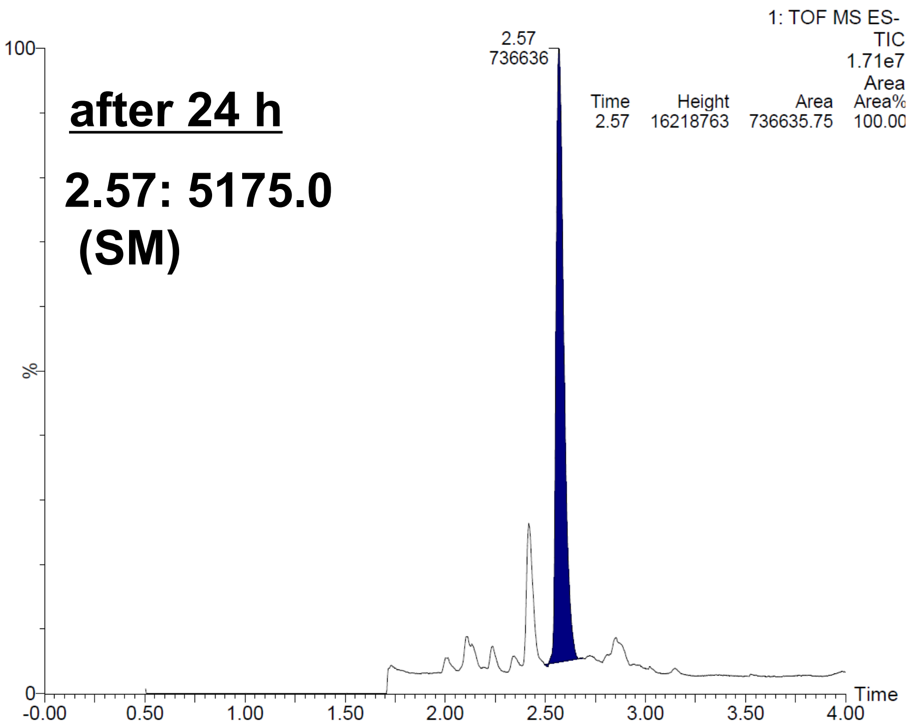

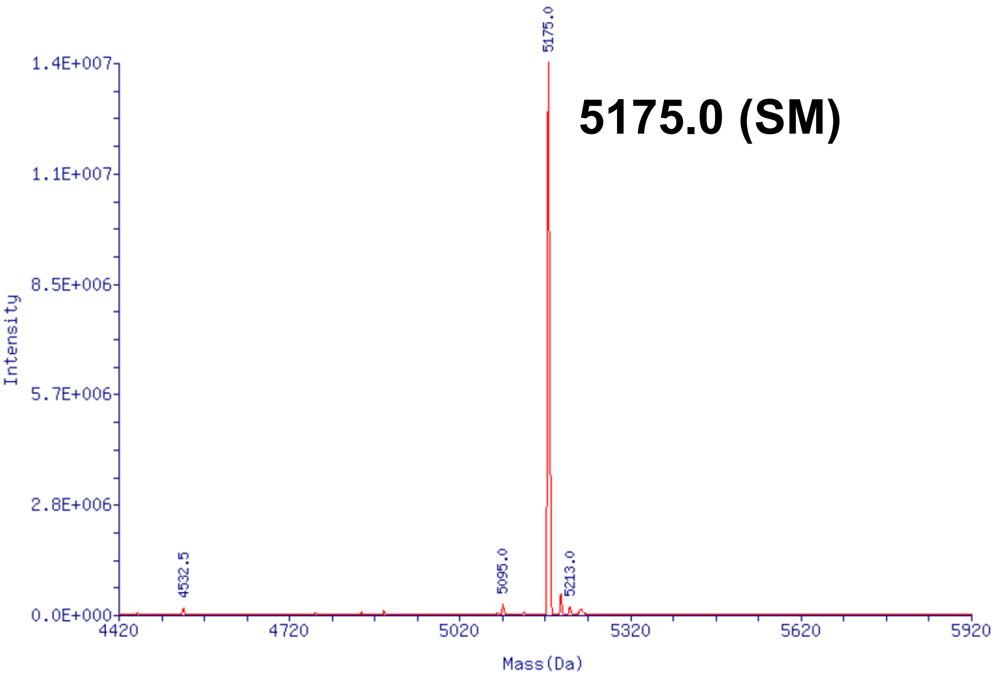

Reduction kinetics of on-DNA diazide 4 in T4 DNA ligase buffer at 35° C: formation of monoamine 10 and diamine 11.

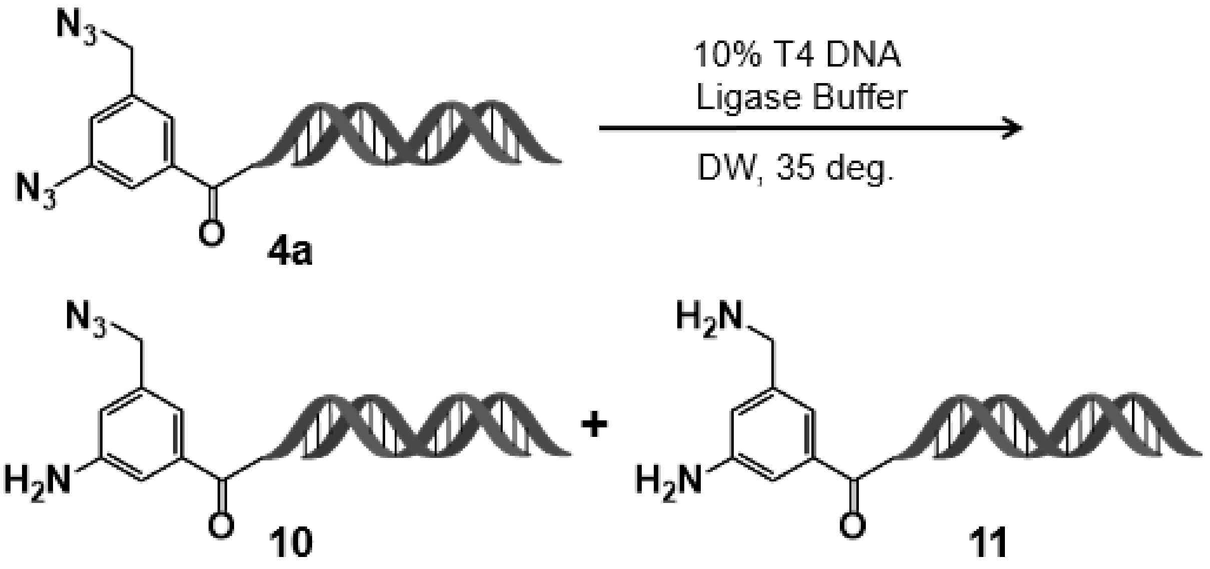

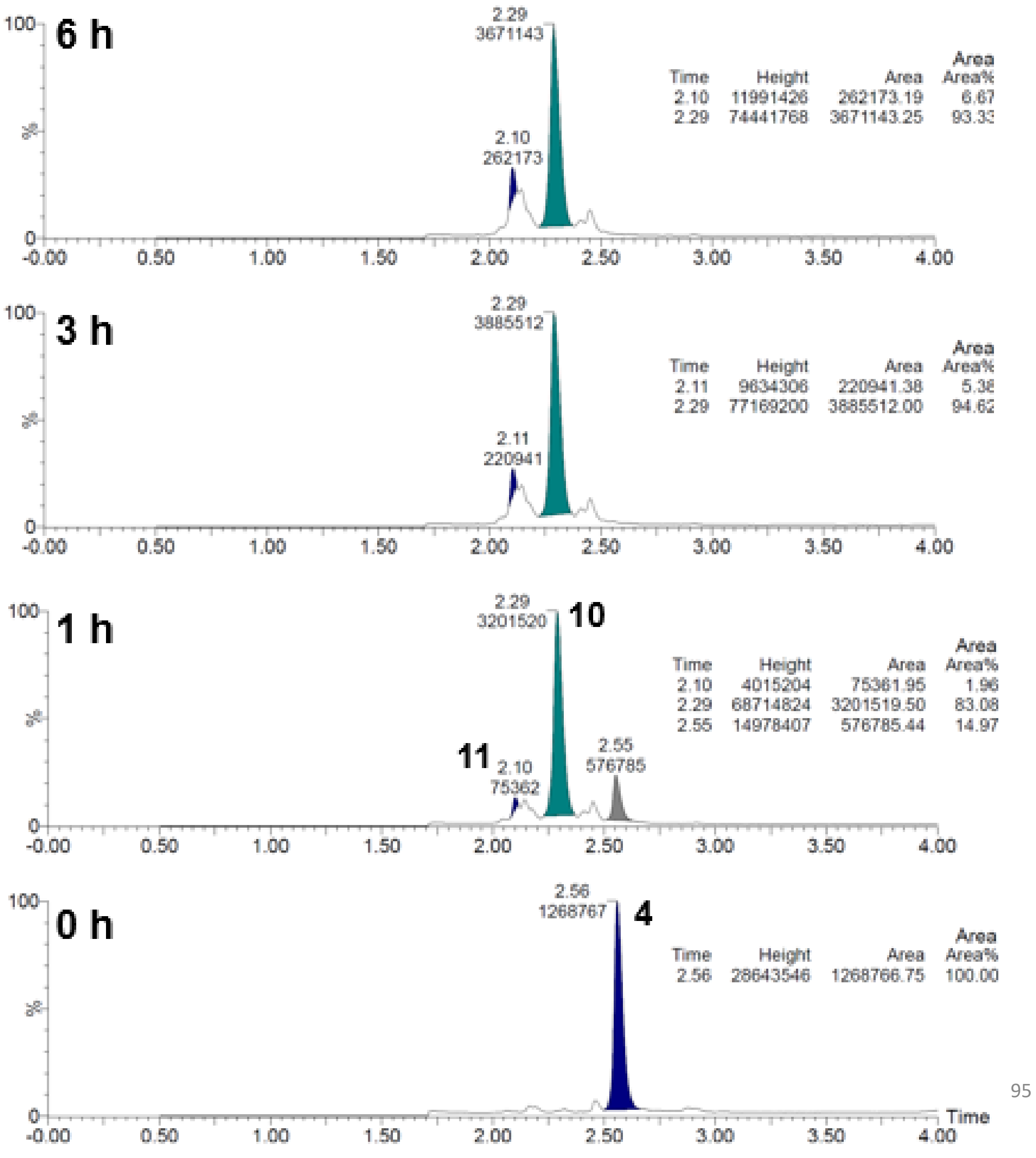

2.5. DEL Construction Using C-D-DAP (3): On-DNA Amino–Azido Platform

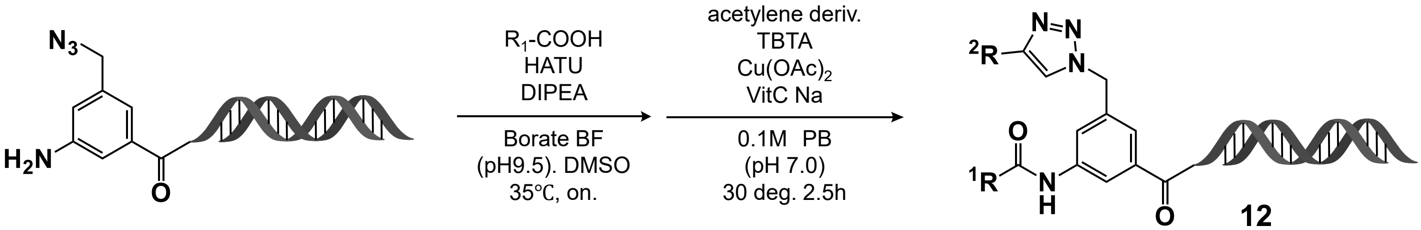

12

No.	R^1	R^2	conversion (%)	MS. (calcd/found)
12a	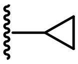	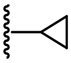	96.2	5244.90 / 5245.0
12b		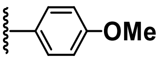	96.8	5311.0 / 5311.3
12c		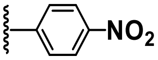	96.9	5326.0 / 5326.2
12d		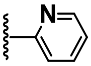	96.4	5282.0 / 5281.9
12e	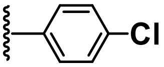	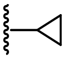	98.0	5315.0 / 5315.5
12f		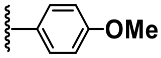	97.0	5381.0 / 5381.6
12g		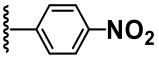	99.1	5396.0 / 5396.9

12a

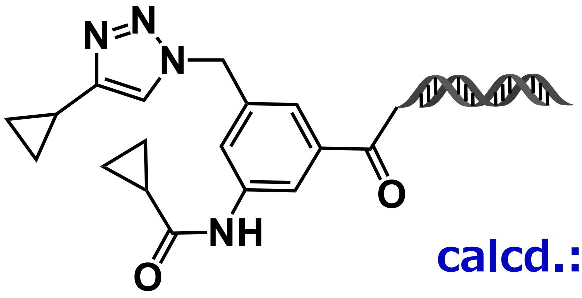

calcd.: 5244.9

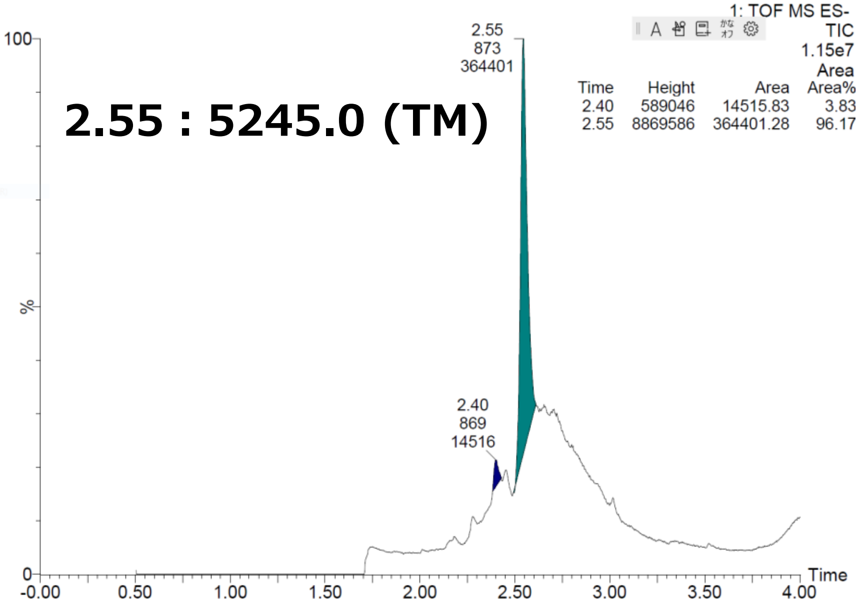

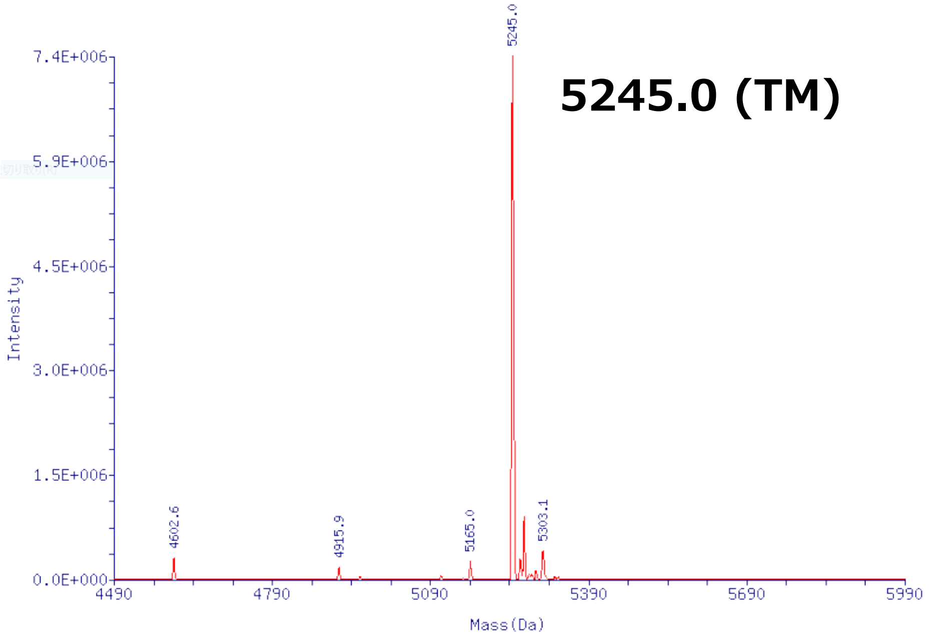

12b

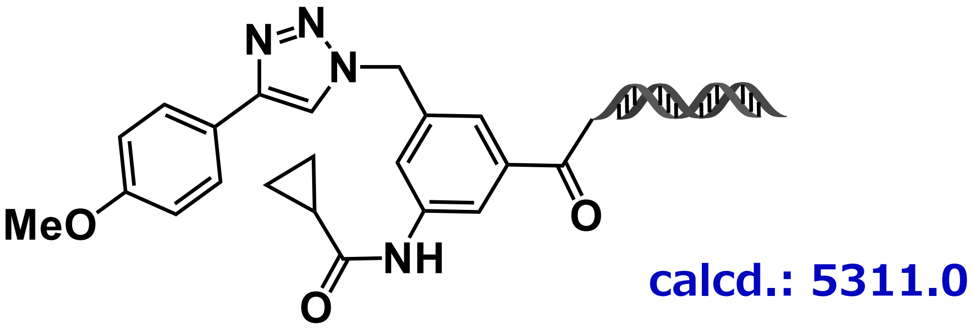
MeO
NH
calcd.: 5311.0

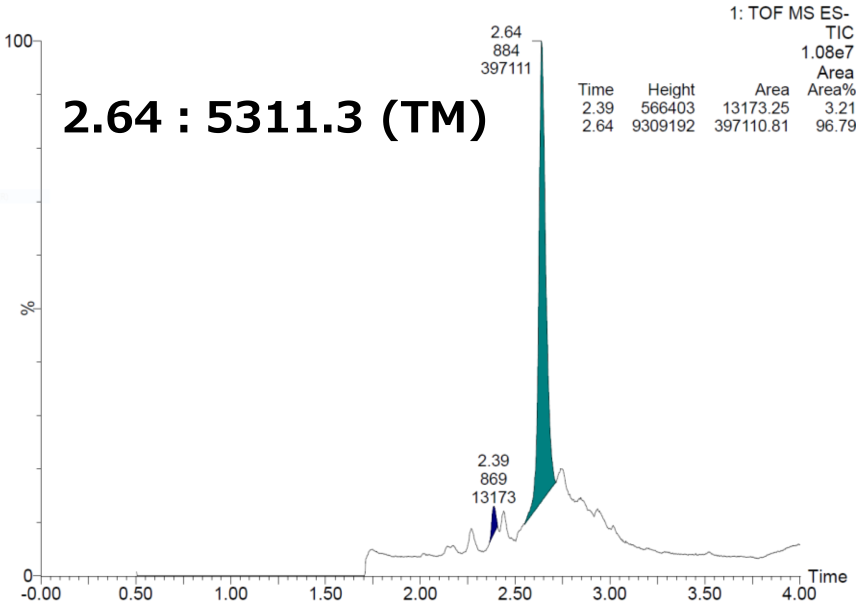
1: TOF MS ES-
TIC
1.08e7
Area
2.64 : 5311.3 (TM)
Time Height Area Area%
2.39 566403 13173.25 3.21
2.64 9309192 397110.81 96.79
2.64
884
397111
2.39
869
13173
Time
%

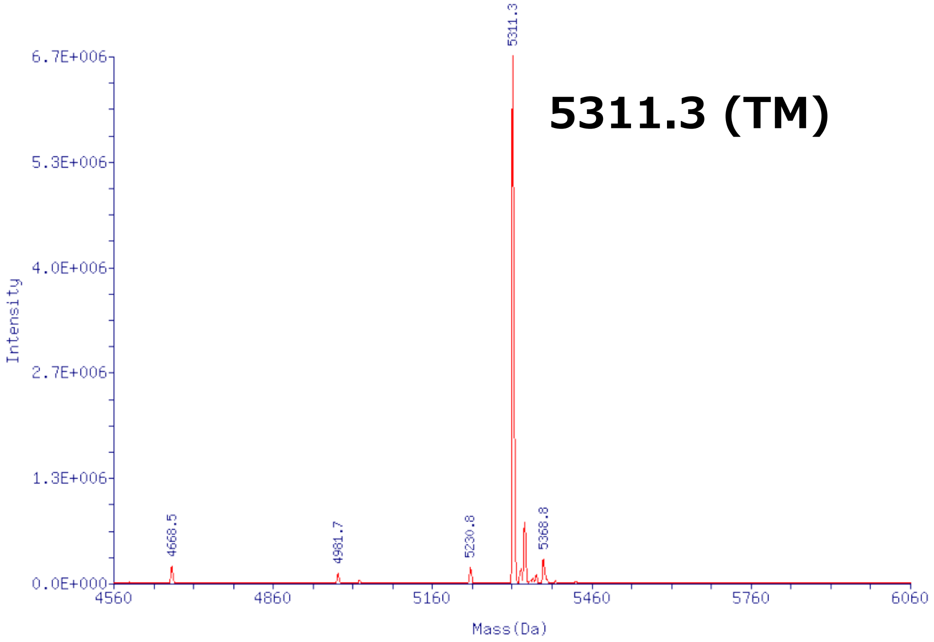
5311.3
5311.3 (TM)
4668.5
4981.7
5230.8
5368.8
Intensity
Mass(Da)

12c

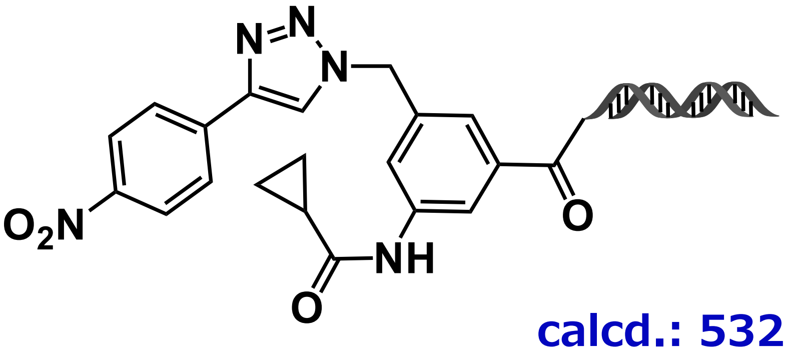

calcd.: 5326.0

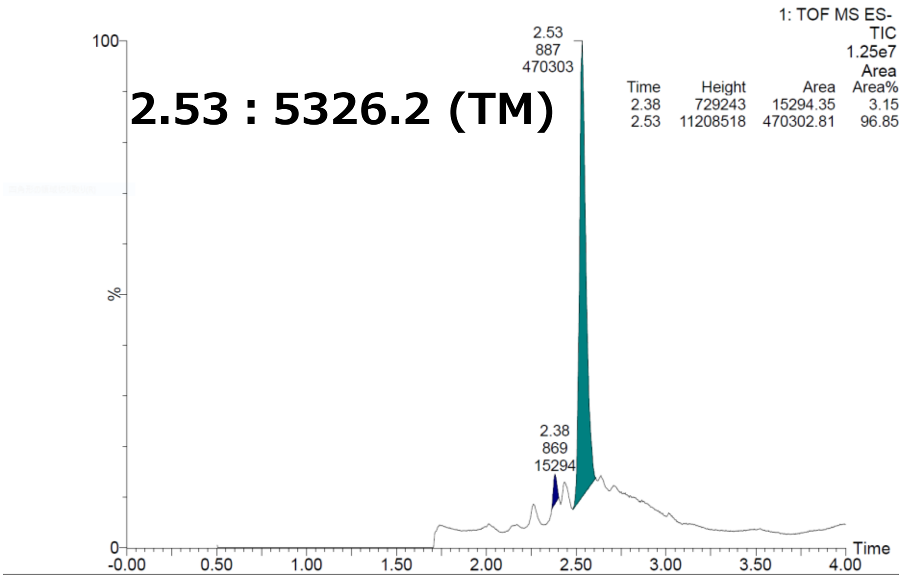

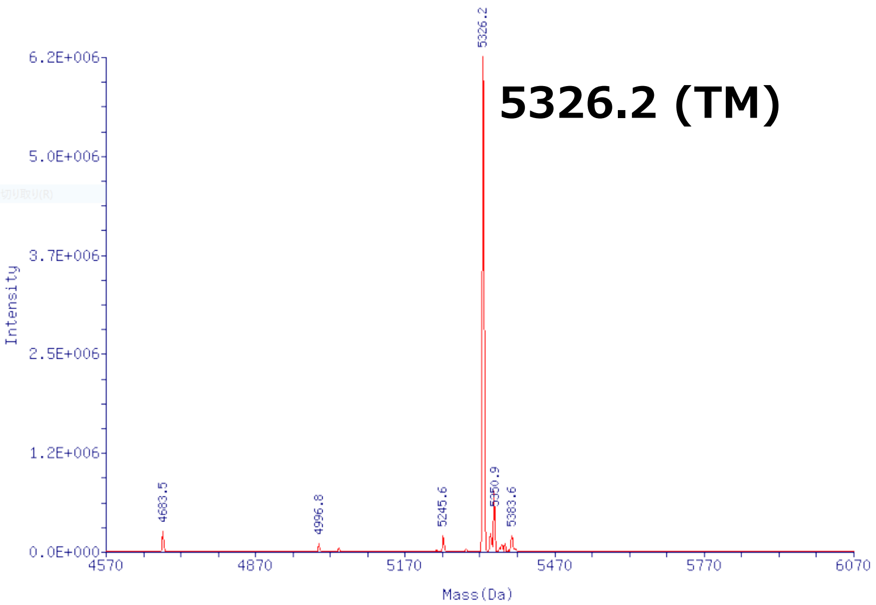

12d

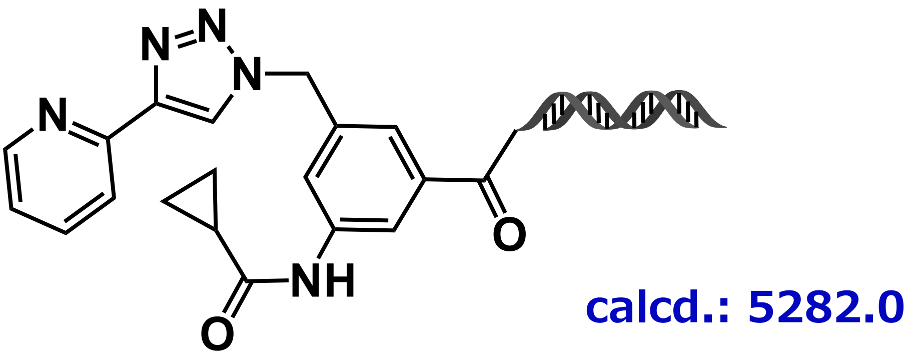

N
N
N
N
NH
O
O
calcd.: 5282.0

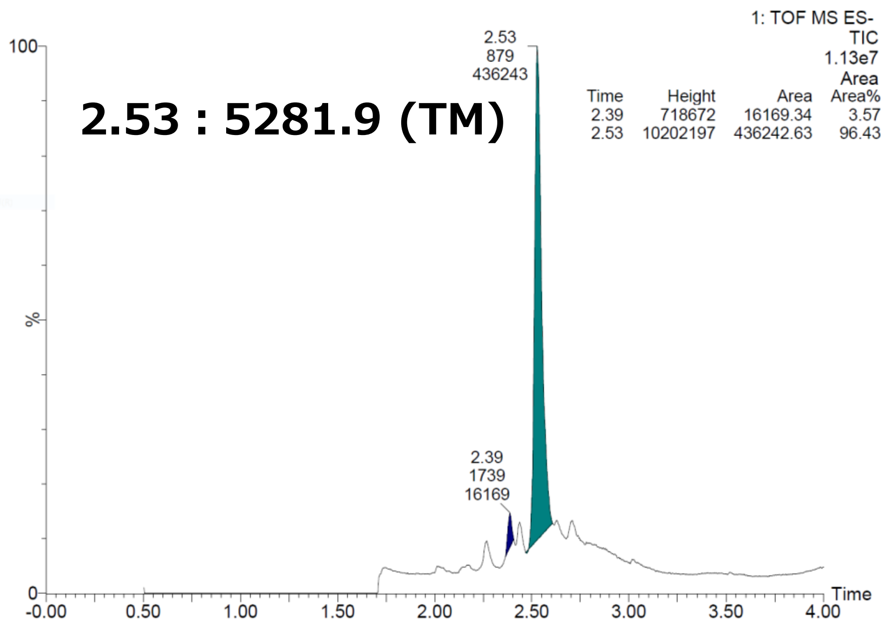

1: TOF MS ES-
TIC
1.13e7
Area
2.53 : 5281.9 (TM)
2.53
879
436243
2.39
1739
16169
Time Height Area Area%
2.39 718672 16169.34 3.57
2.53 10202197 436242.63 96.43
100
%
0
-0.00 0.50 1.00 1.50 2.00 2.50 3.00 3.50 4.00
Time

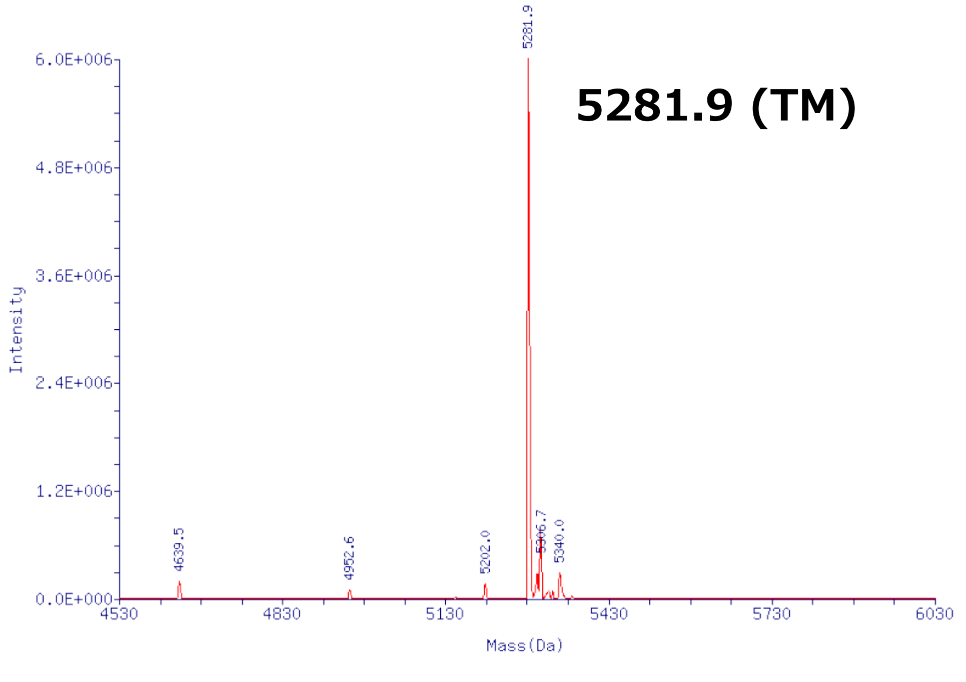

5281.9
5281.9 (TM)
6.0E+006
4.8E+006
3.6E+006
2.4E+006
1.2E+006
0.0E+000
Intensity
4639.5
4952.6
5202.0
5306.7
5340.0
4530 4830 5130 5430 5730 6030
Mass(Da)

12e

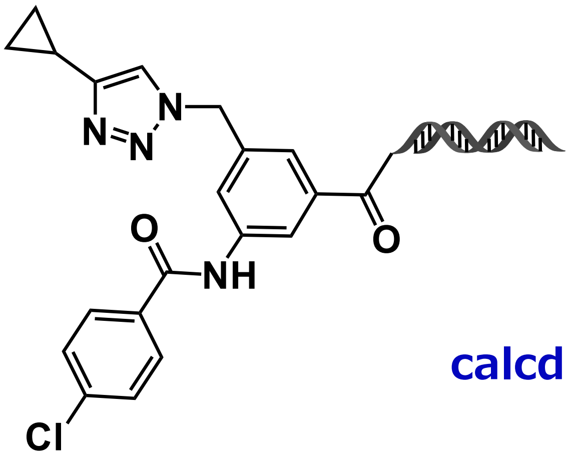

calcd.: 5315.0

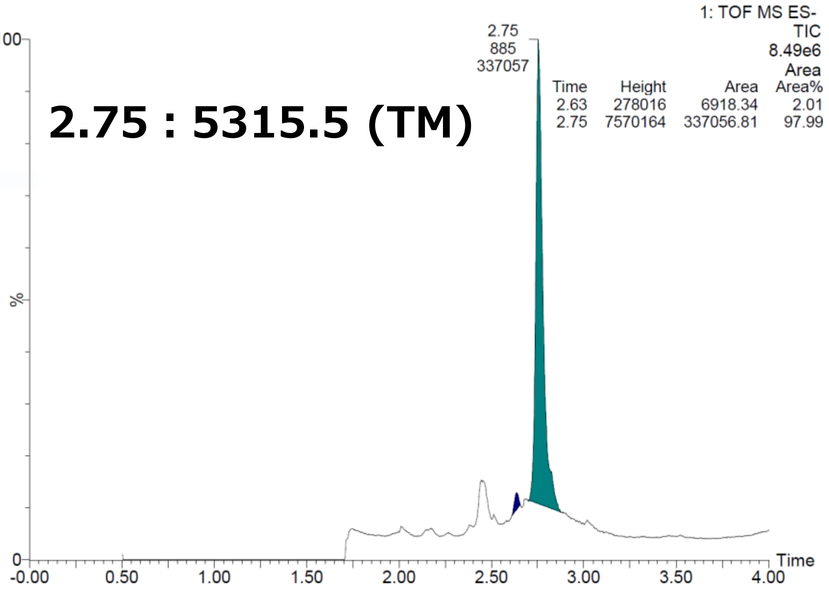

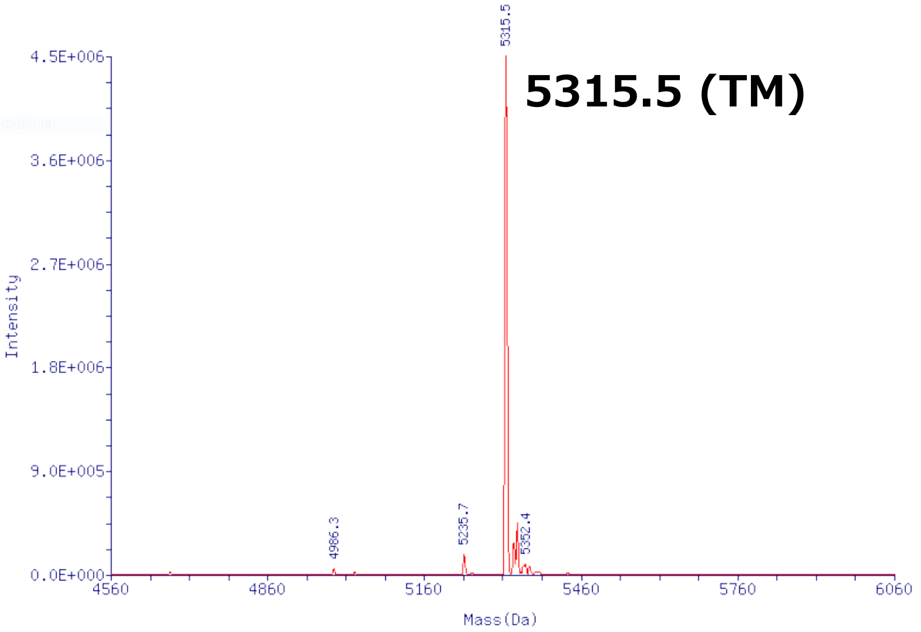

12f

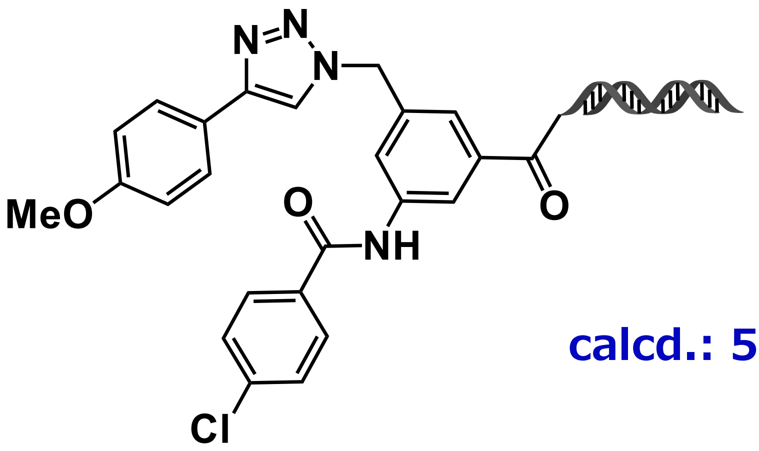

calcd.: 5381.0

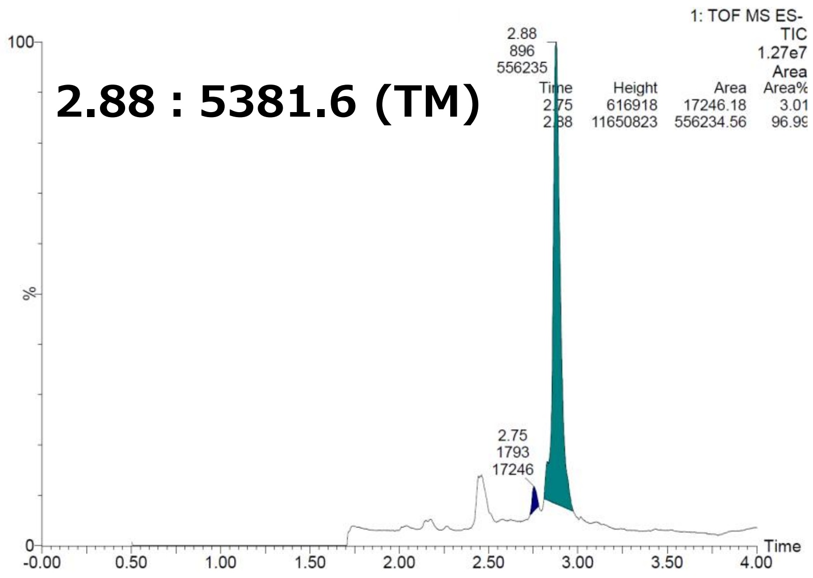

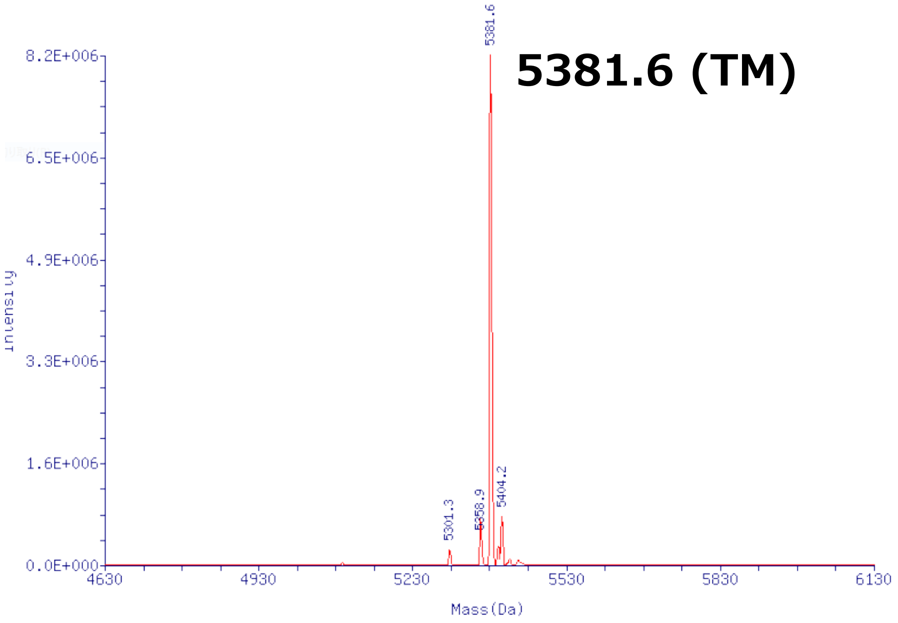

12g

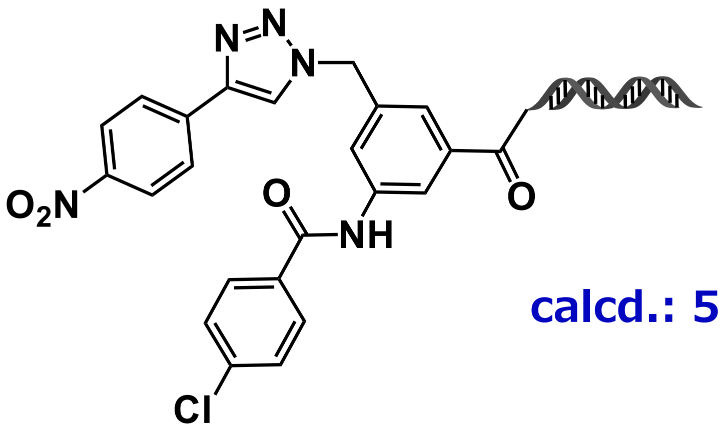

calcd.: 5396.0

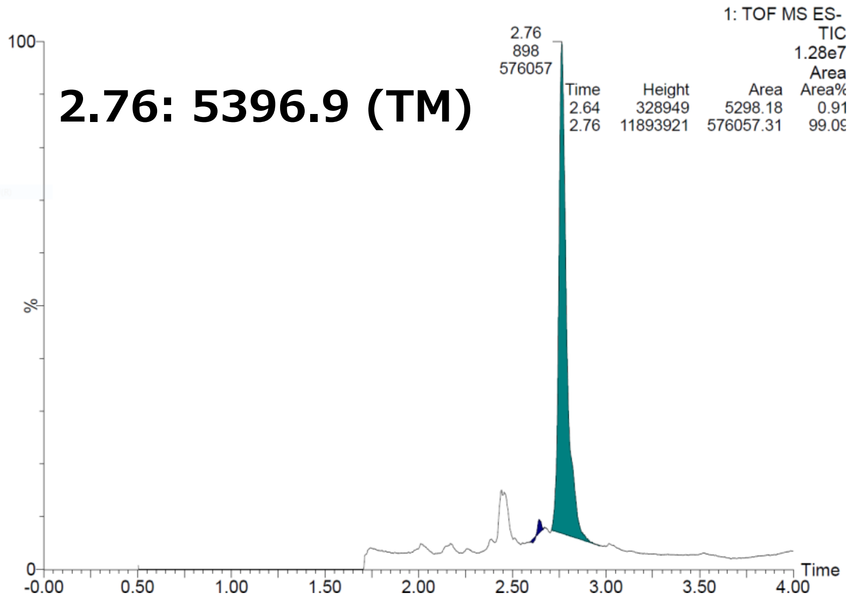

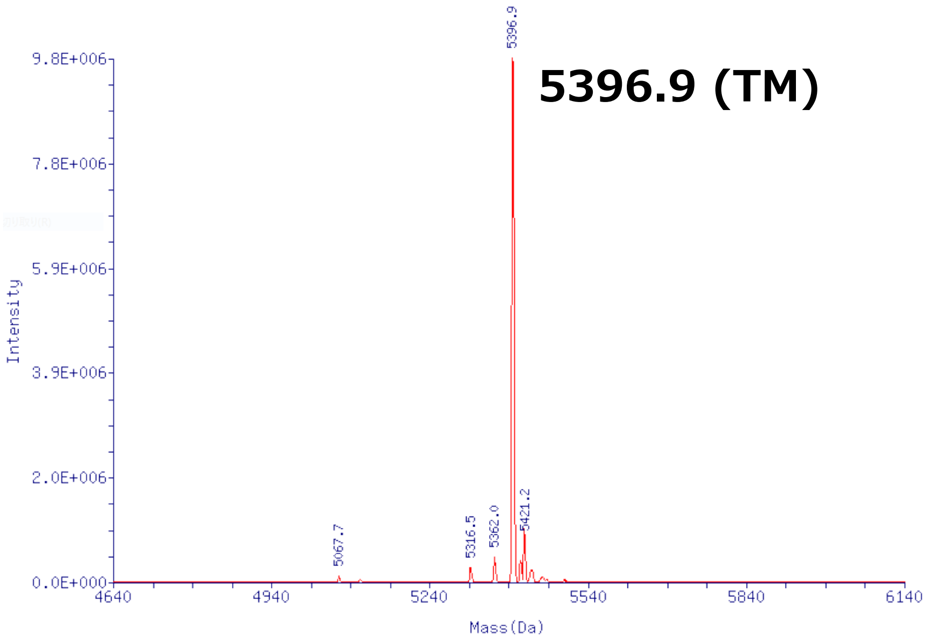

2.6. DEL Construction Using C-D-DAP (4): On-DNA Diamino Platform

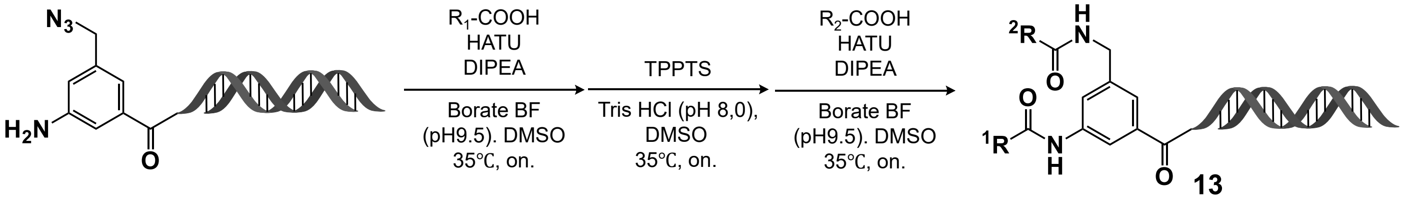

No.	R^1	R^2	conversion (%)	MS. (calcd/found)
13a	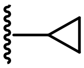	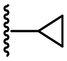	91.0	5221.0 / 5221.2
13b		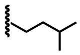	96.2	5251.0 / 5251.0
13c		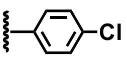	91.5	5290.9 / 5291.2
13d		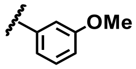	90.5	5287.0 / 5287.5
13e		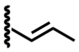	92.3	5221.0 / 5221.2
13f		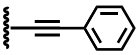	40.8	5281.0 / 5281.0

13a

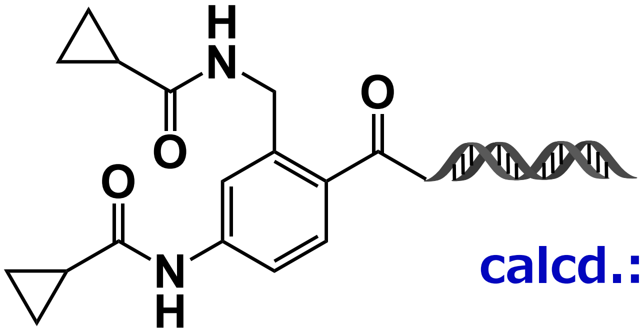

calcd.: 5221.0

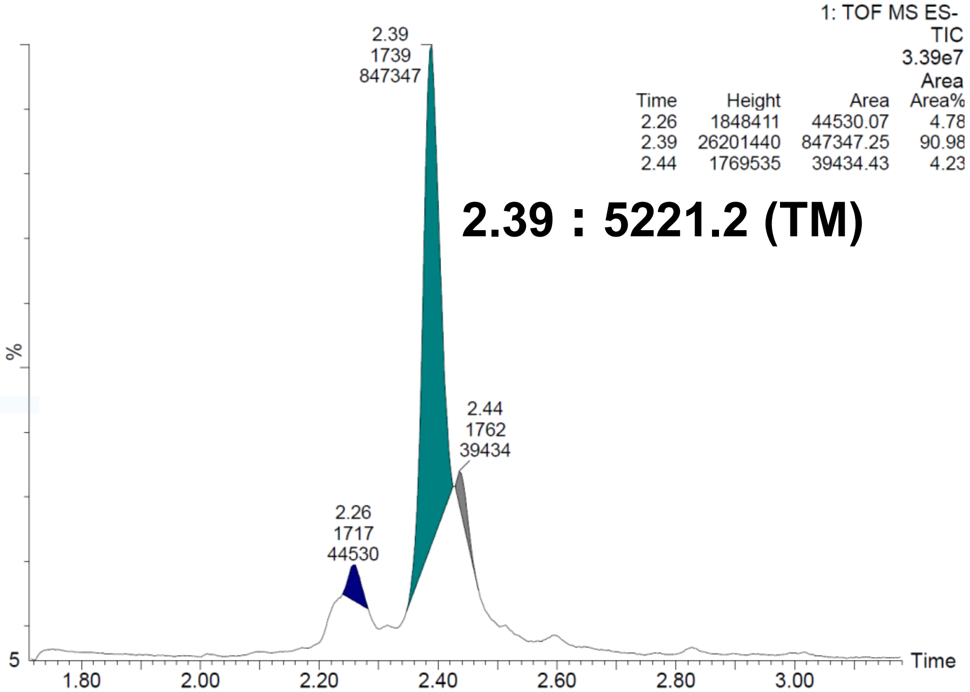

2.39 : 5221.2 (TM)

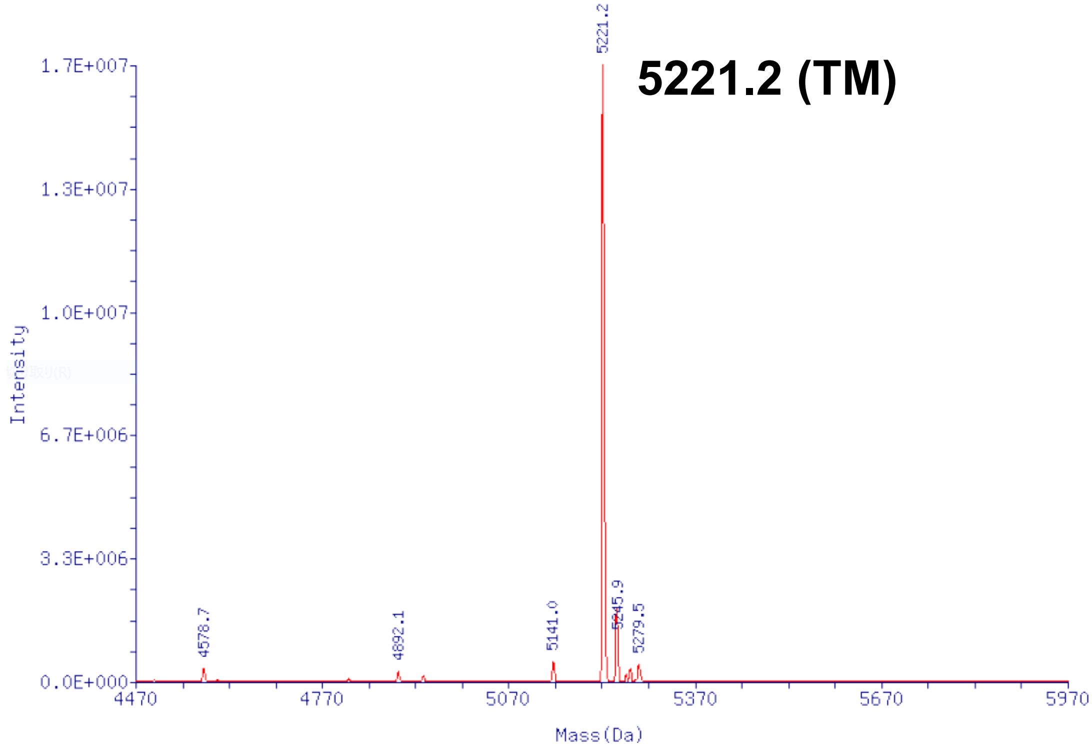

5221.2 (TM)

13b

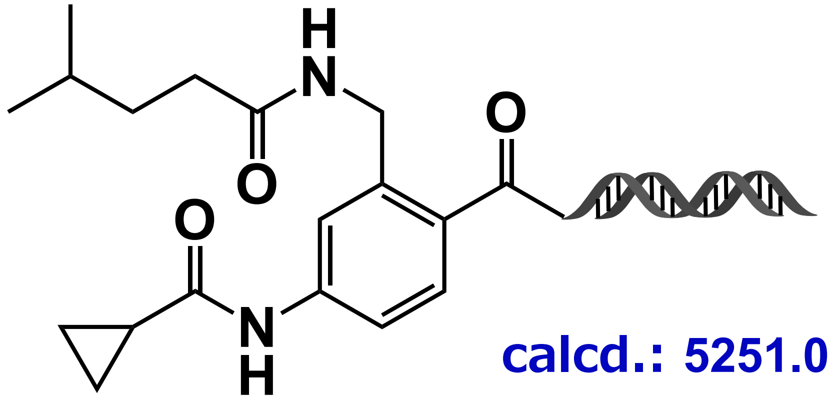
calcd.: 5251.0

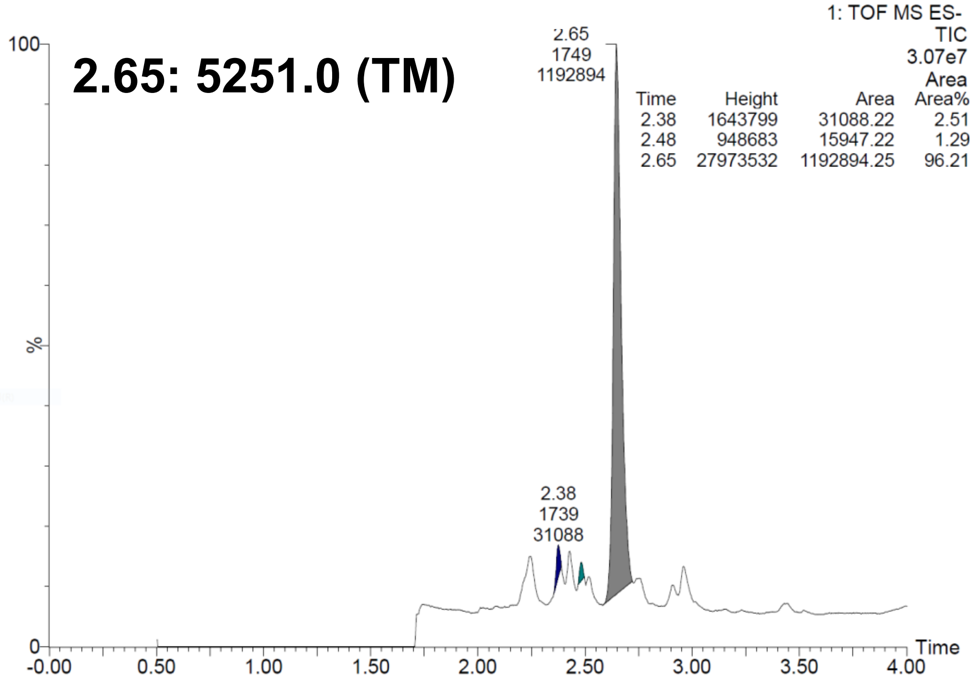
2.65: 5251.0 (TM)
1: TOF MS ES-
TIC
3.07e7
2.65
1749
1192894
Time Height Area Area%
2.38 1643799 31088.22 2.51
2.48 948683 15947.22 1.29
2.65 27973532 1192894.25 96.21
2.38
1739
31088
%
Time

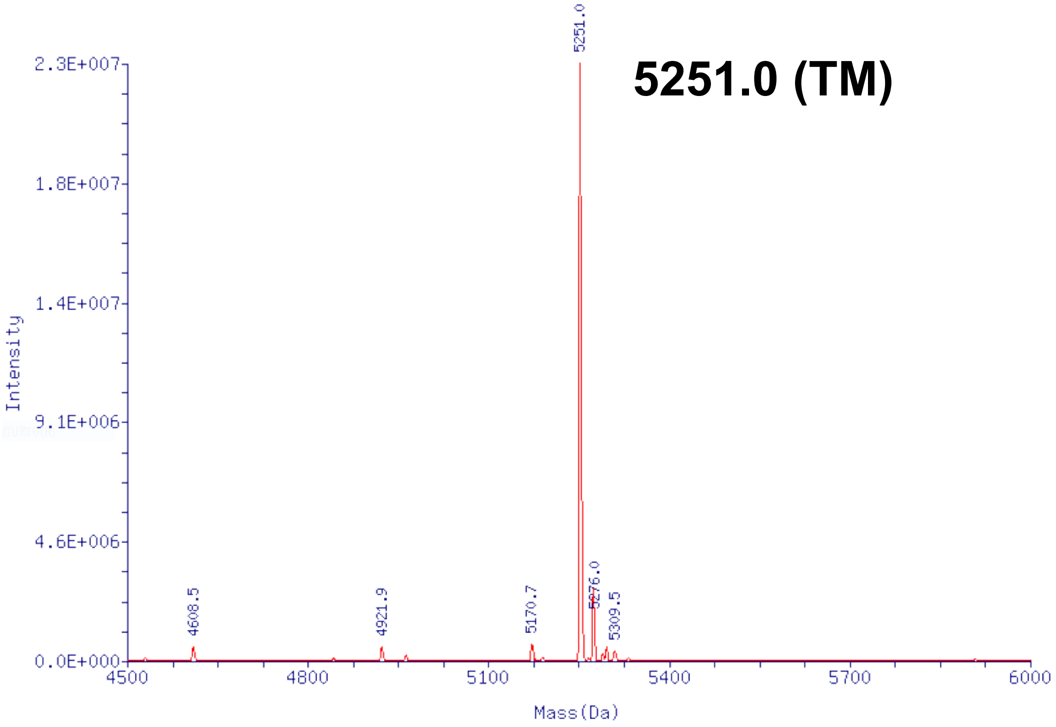
5251.0 (TM)
5251.0
4608.5
4921.9
5170.7
5276.0
5309.5
Intensity
Mass(Da)

13c

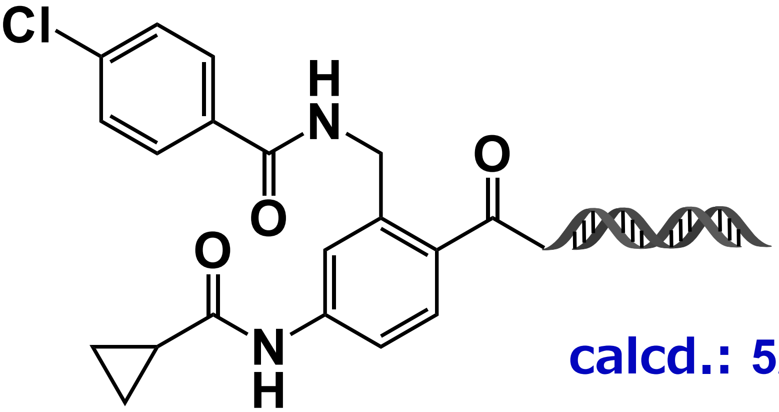

calcd.: 5290.9

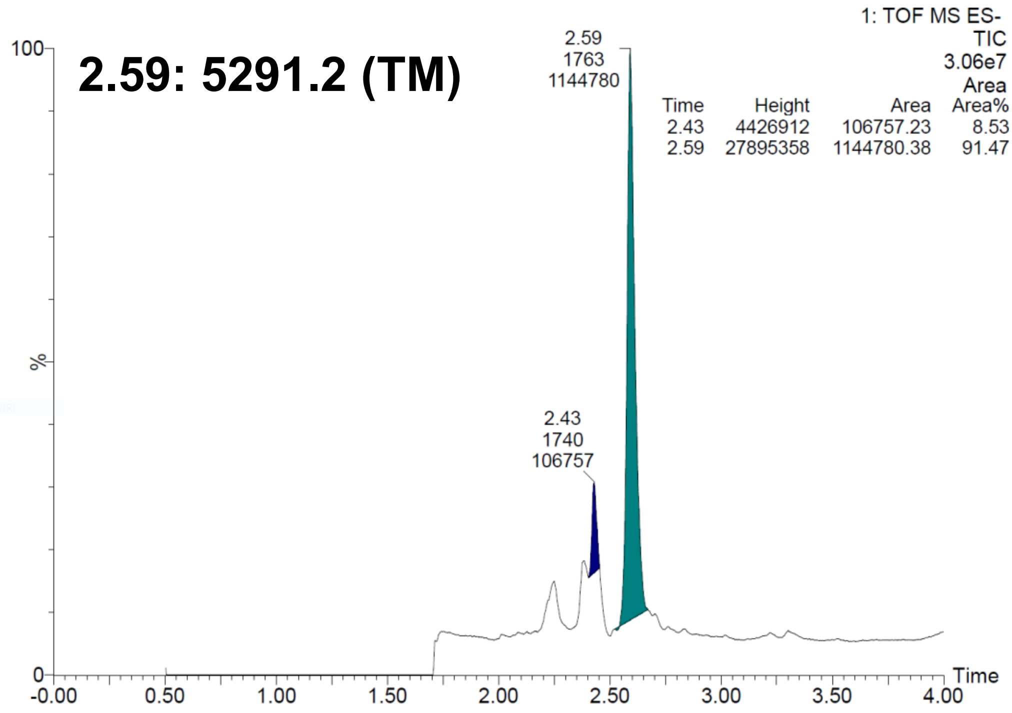

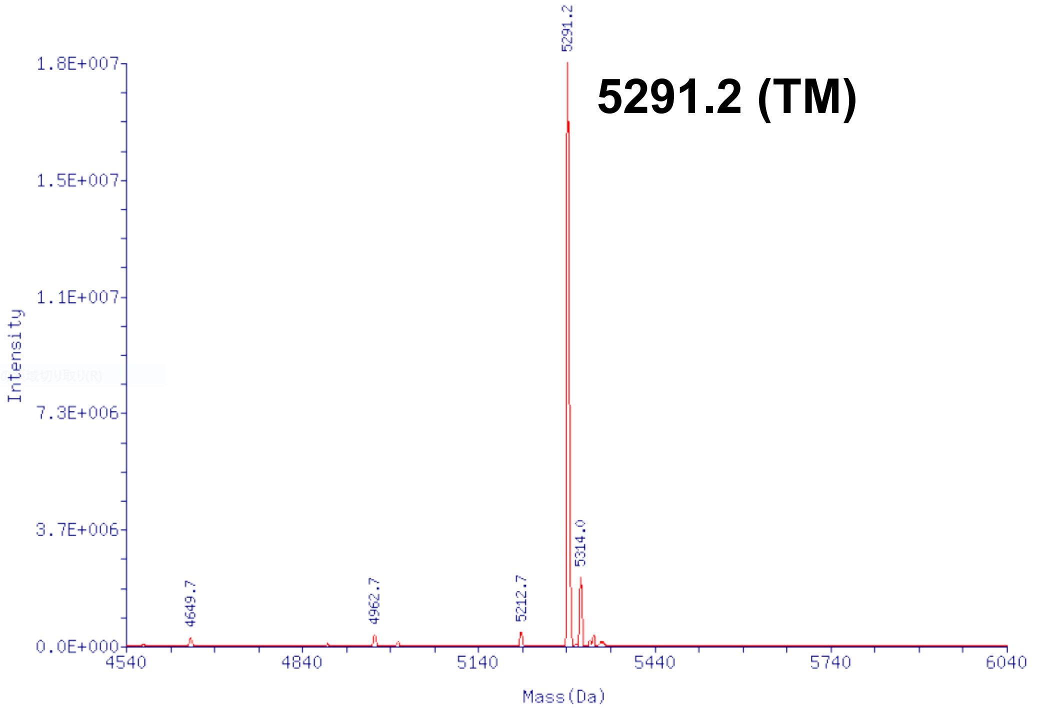

13d

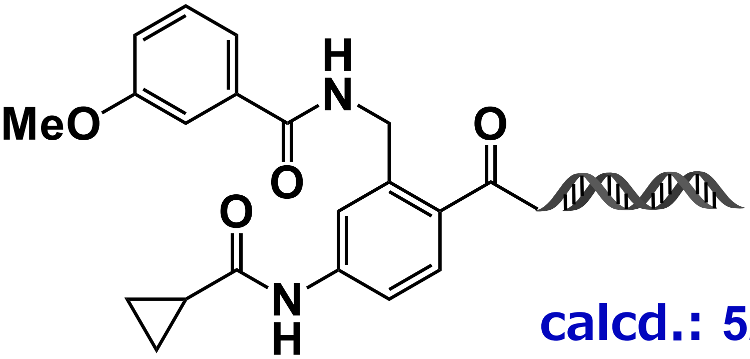

calcd.: 5287.0

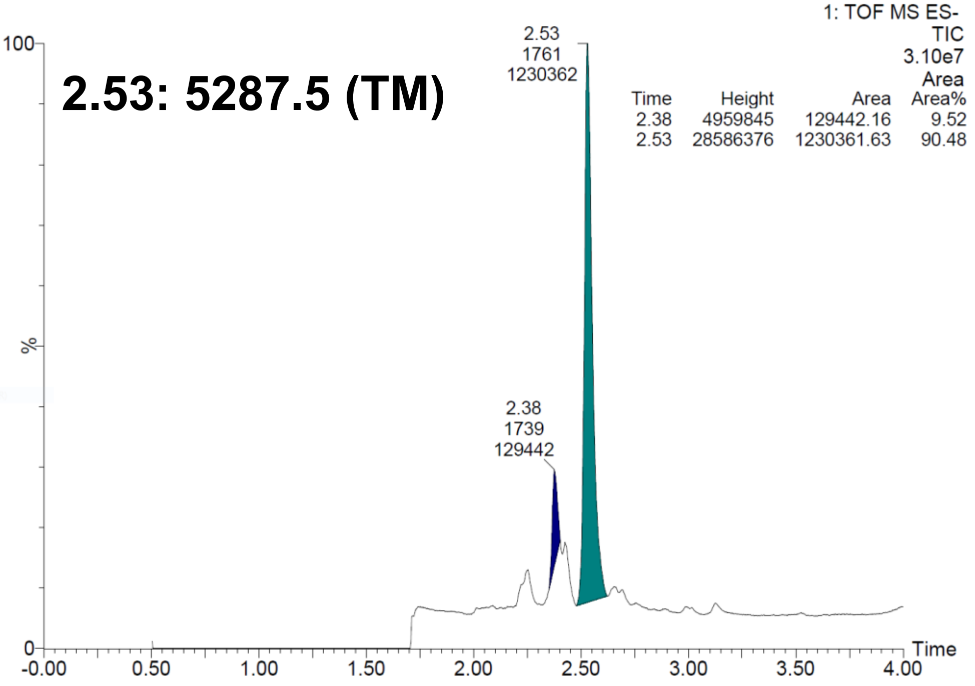

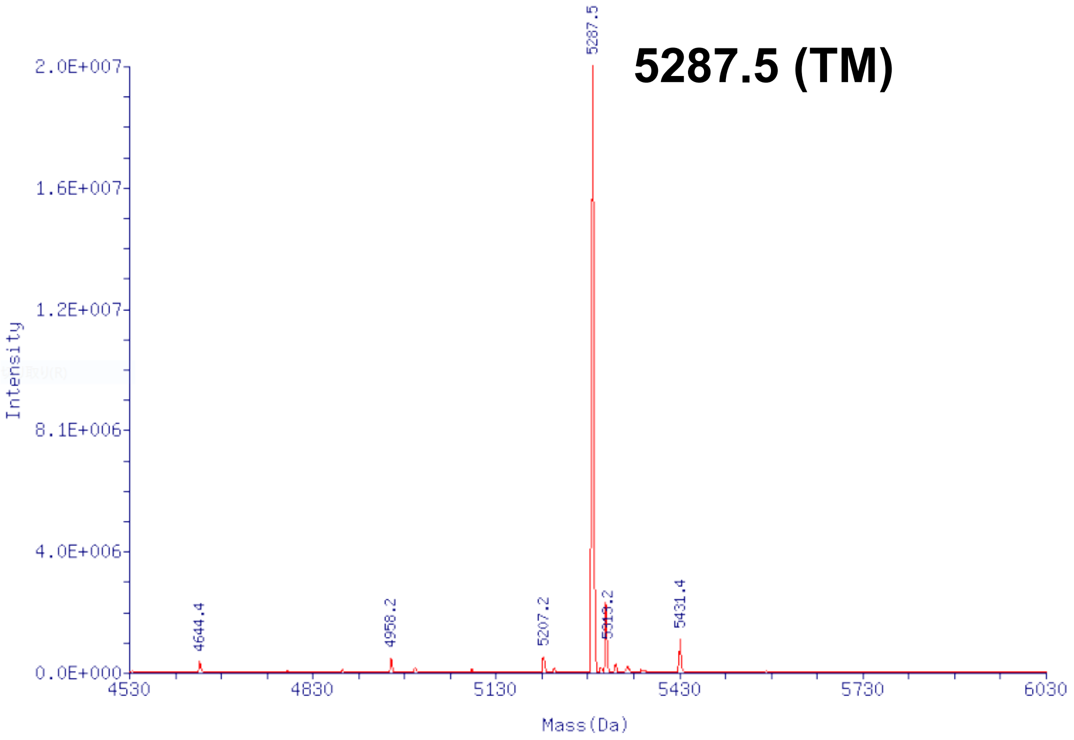

13e

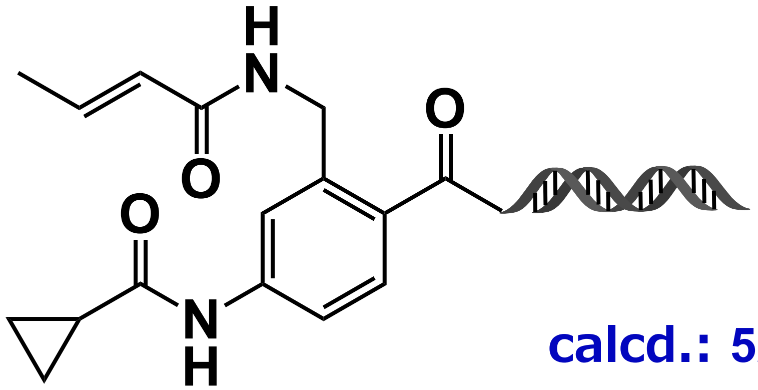

calcd.: 5221.0

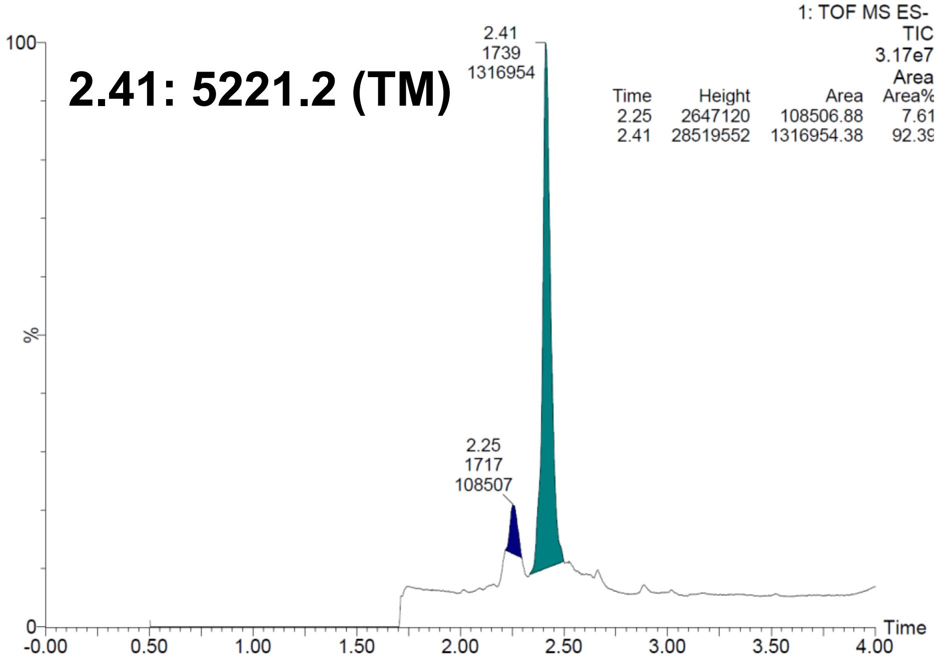

Time	Height	Area	Area%
2.25	2647120	108506.88	7.61
2.41	28519552	1316954.38	92.39

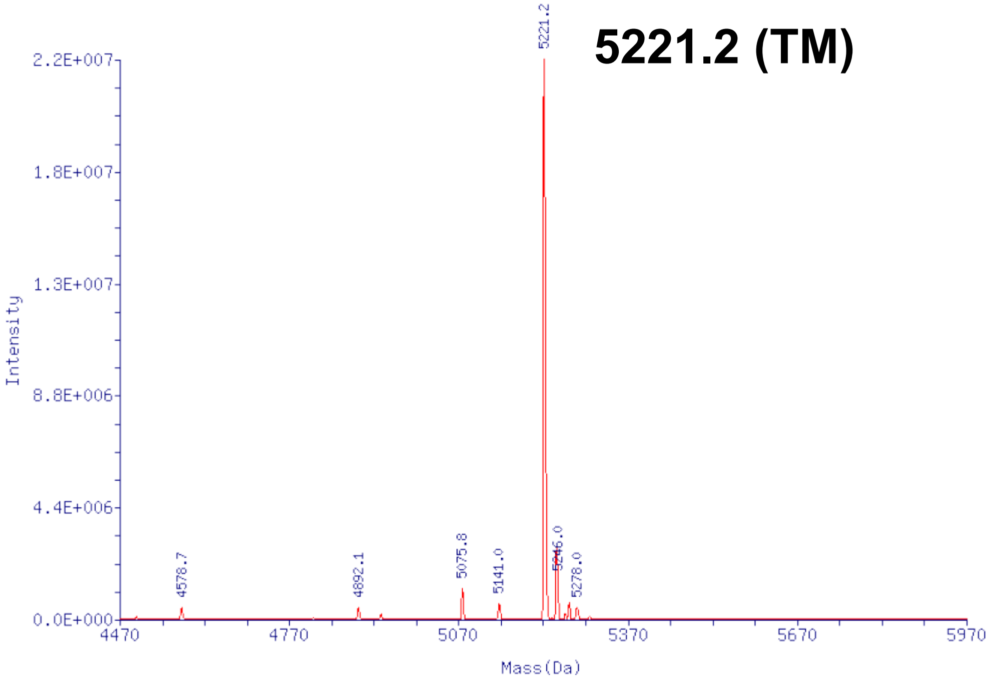

13f

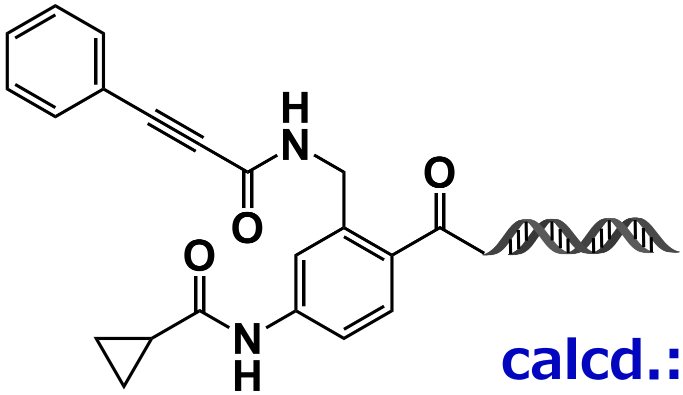

calcd.: 5281.0

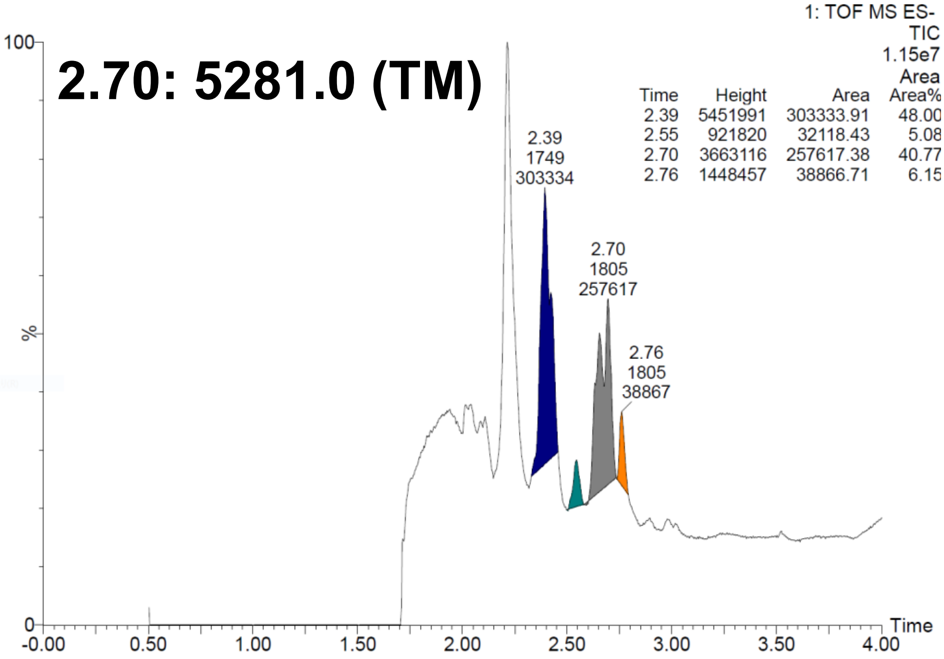

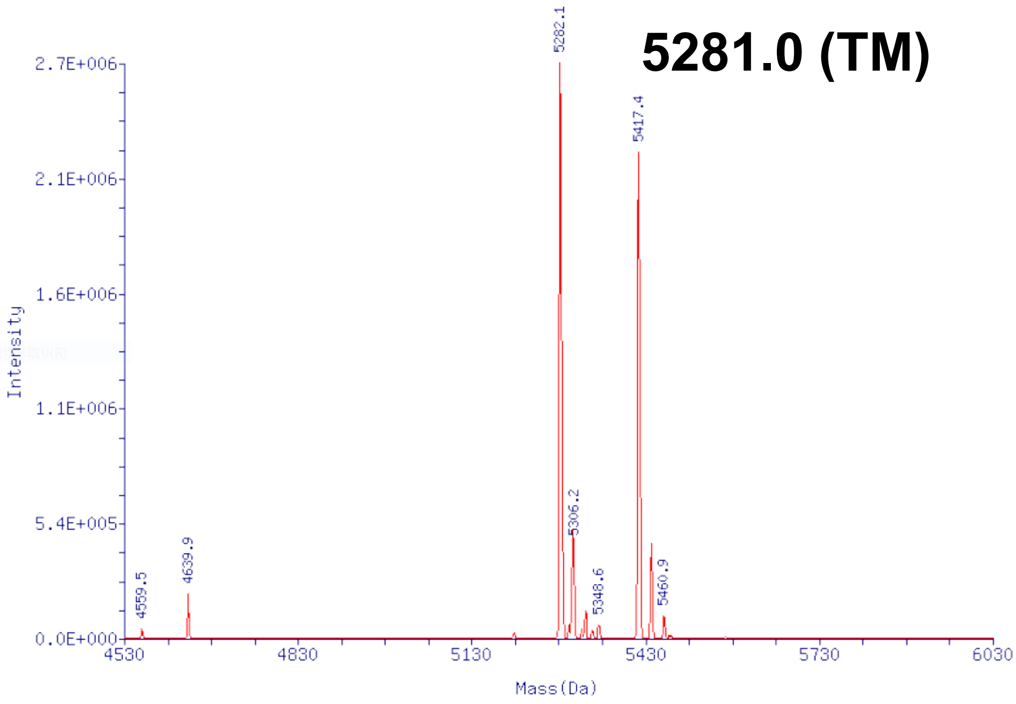

Selective reduction of the azido group at the 4-position of the benzoyl moiety of 2 under T4 DNA ligase buffer conditions.

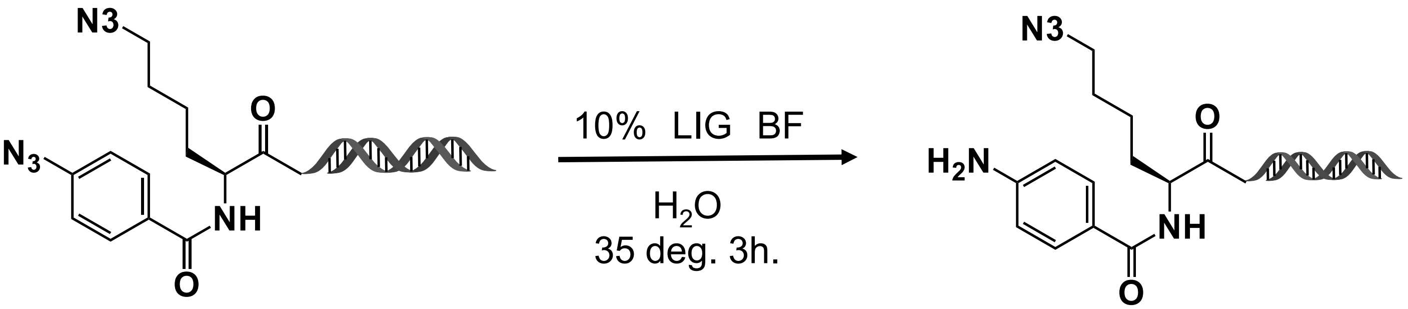

2 **14**

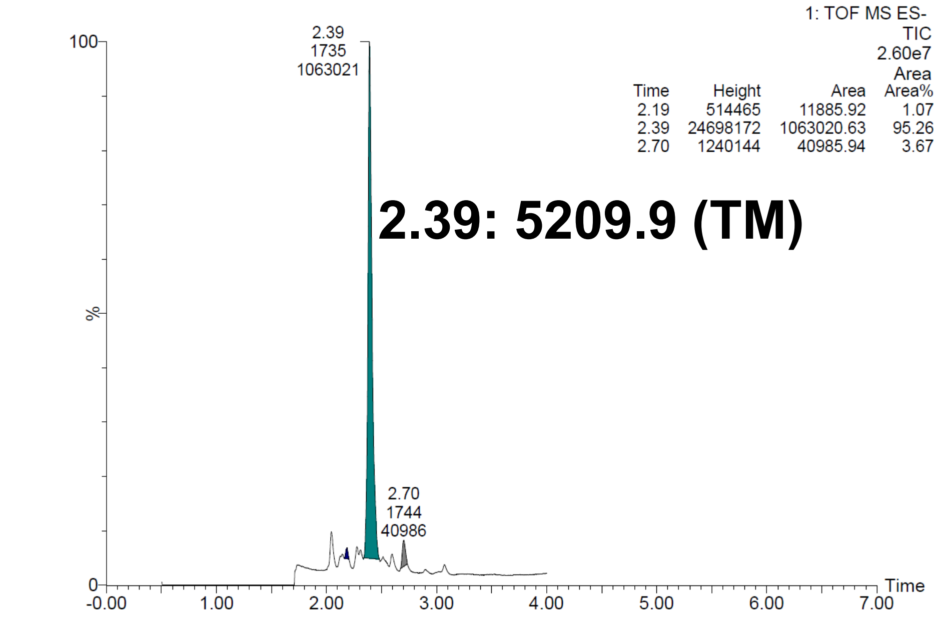

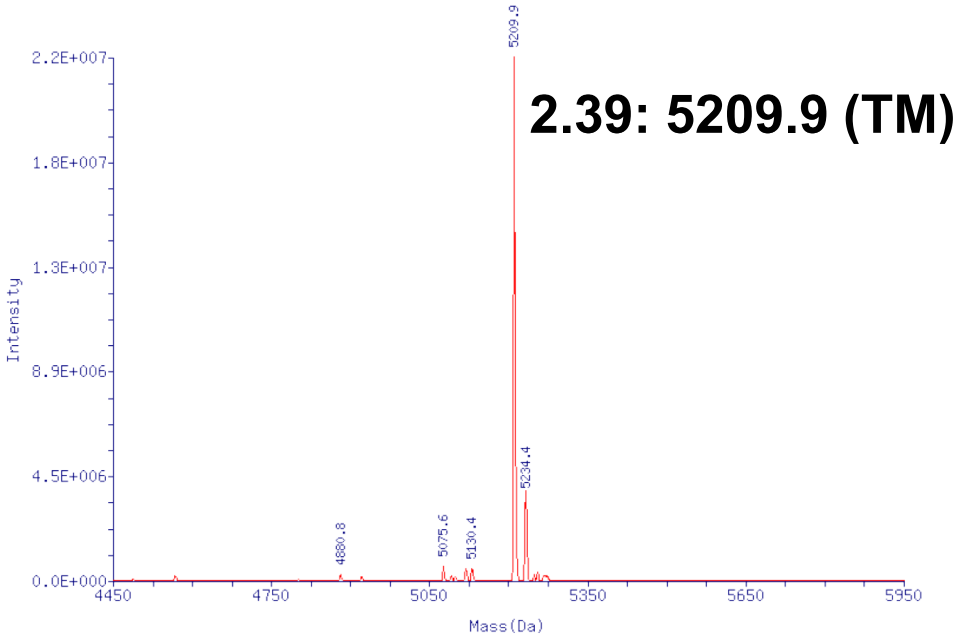

Time course of the selective reduction of the azido group at the 4-position of the benzoyl moiety of under T4 DNA ligase buffer conditions.

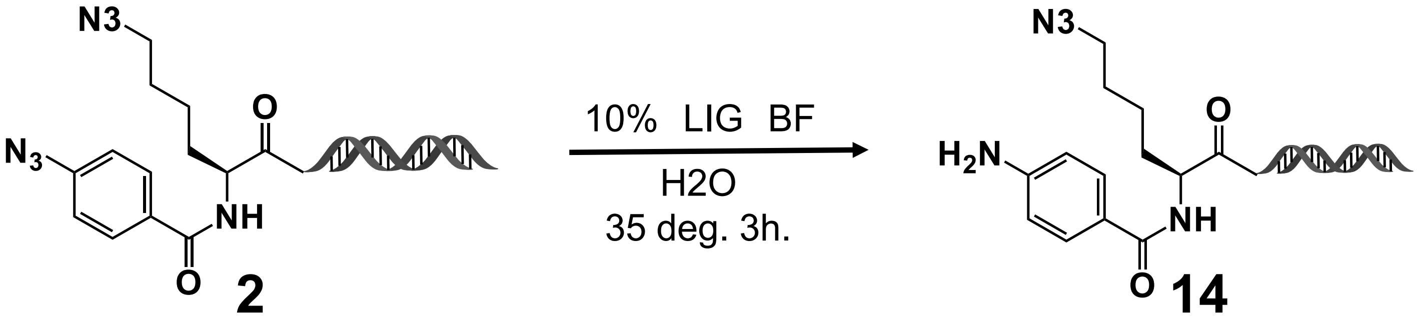

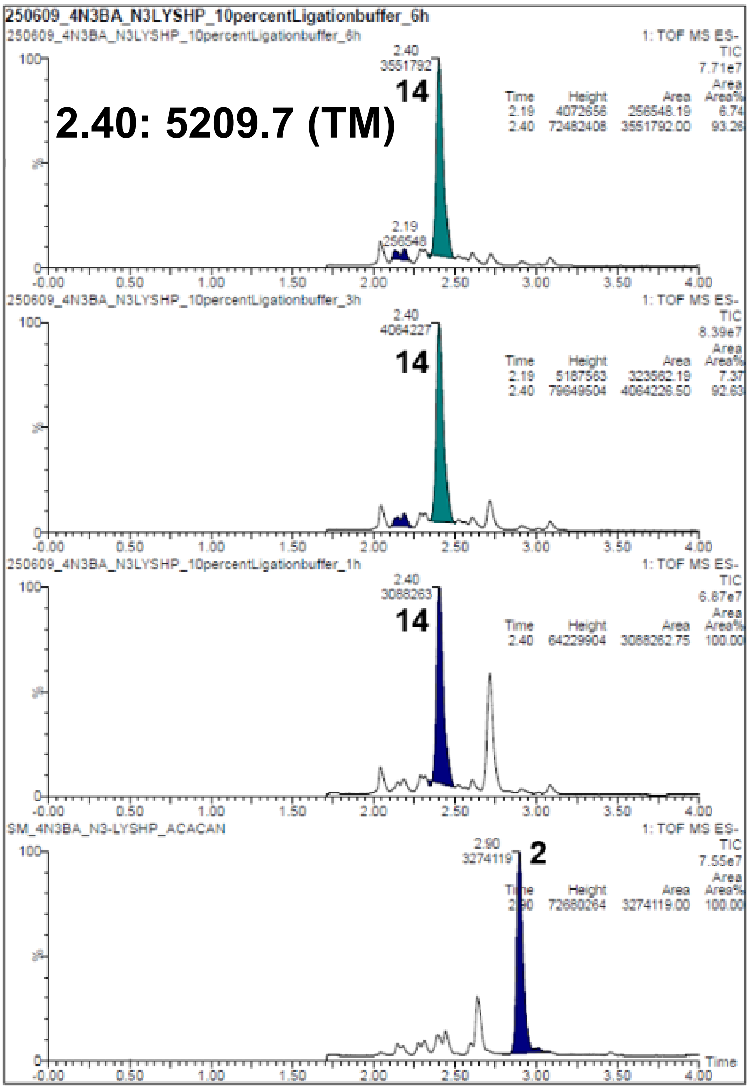

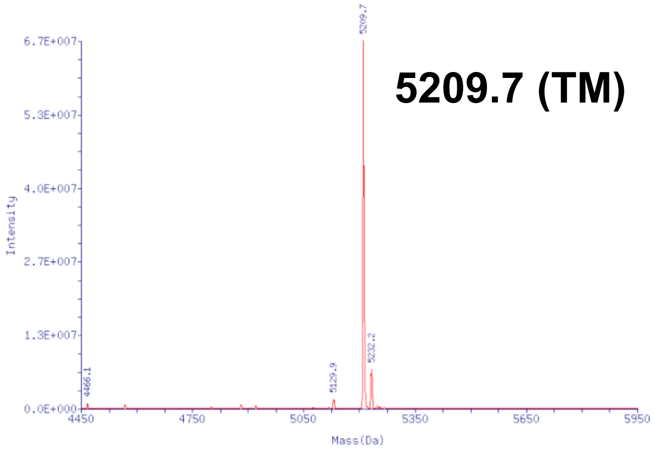

Selective reduction of the azido group at the 4-position of the benzoyl moiety of under T4 DNA ligase buffer conditions, followed by amidation.

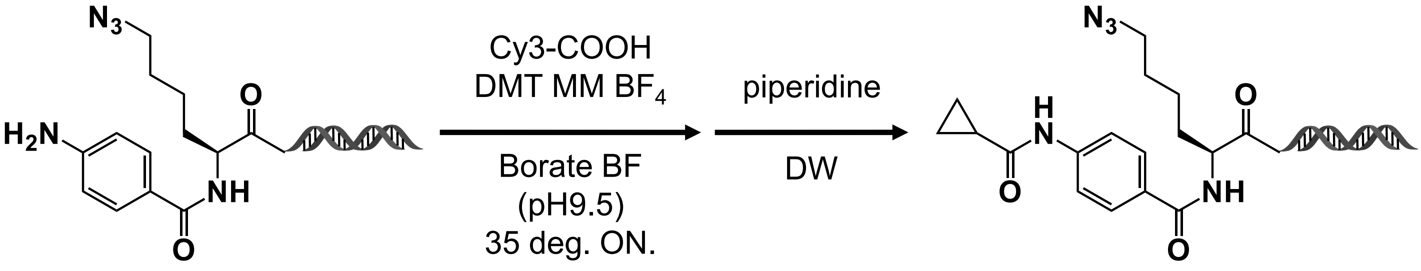

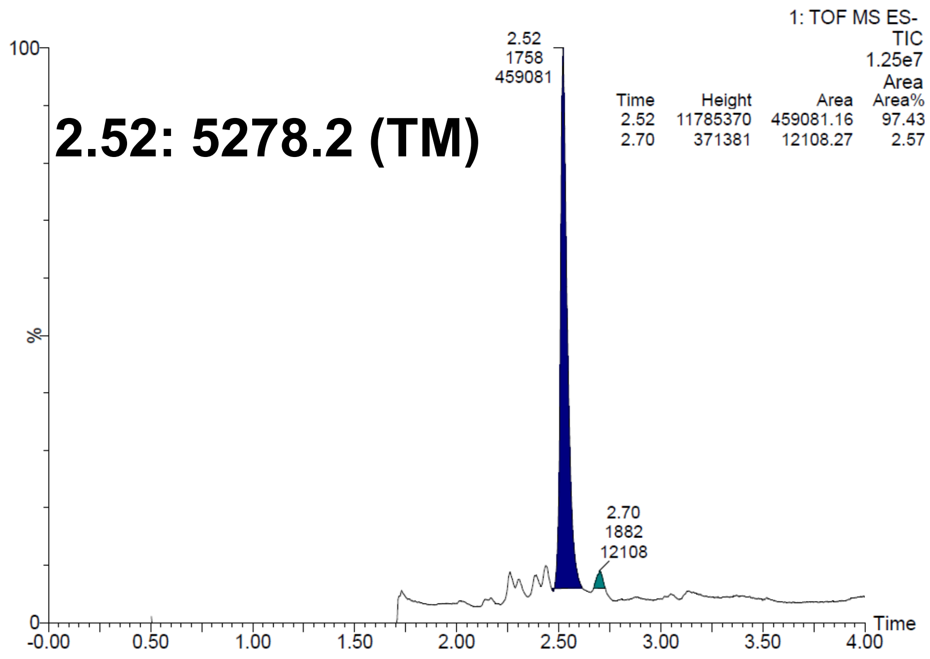

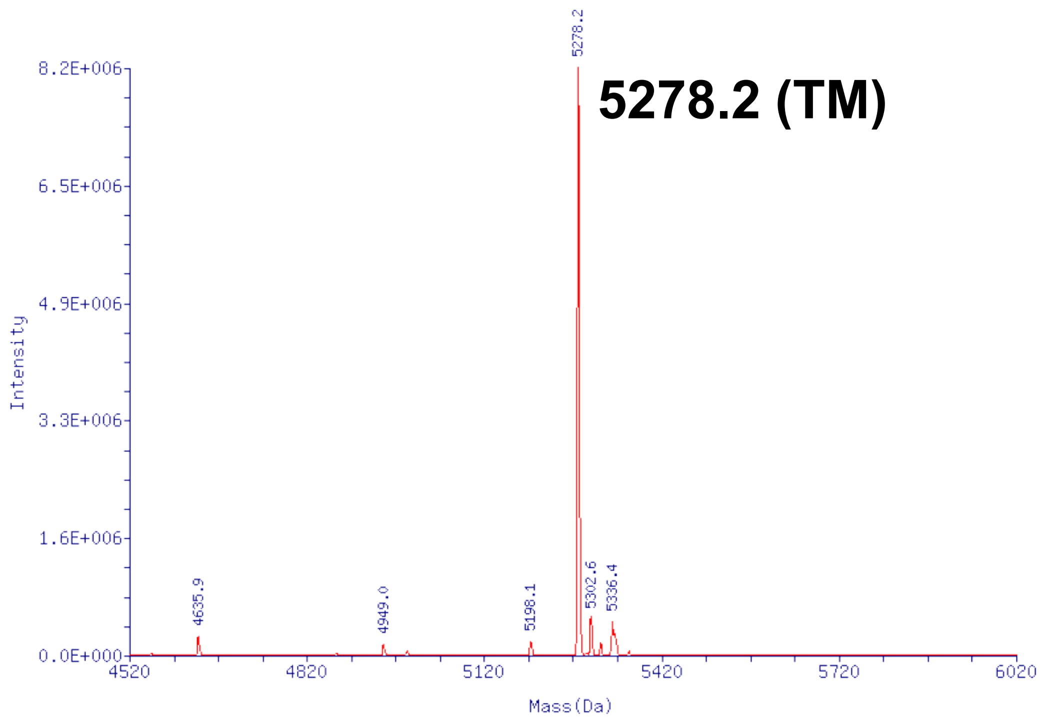

Selective reduction of the azido group at the 4-position of the benzoyl moiety of 2 under T4 DNA ligase buffer conditions, followed by amine capping and subsequent transformation of the aliphatic azide side.

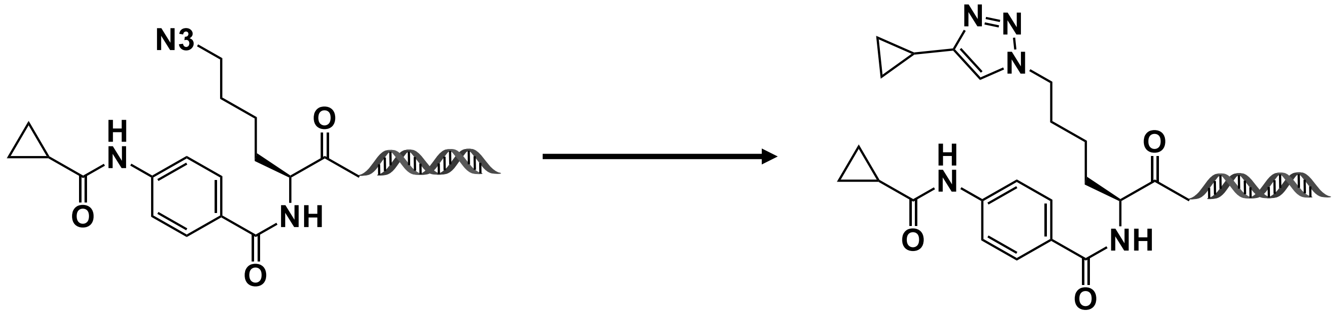

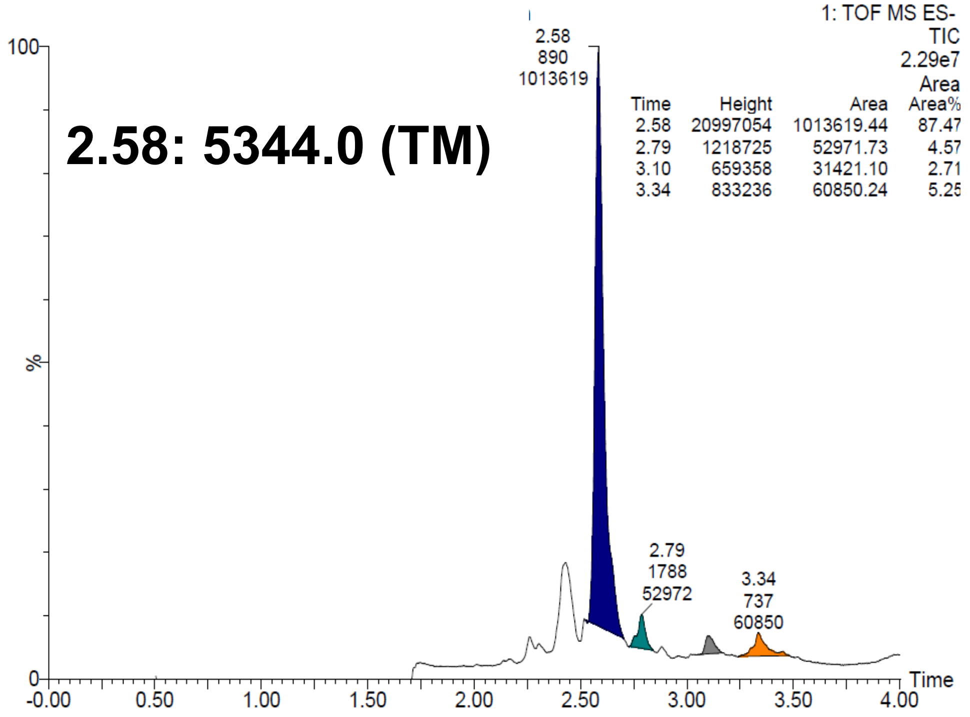

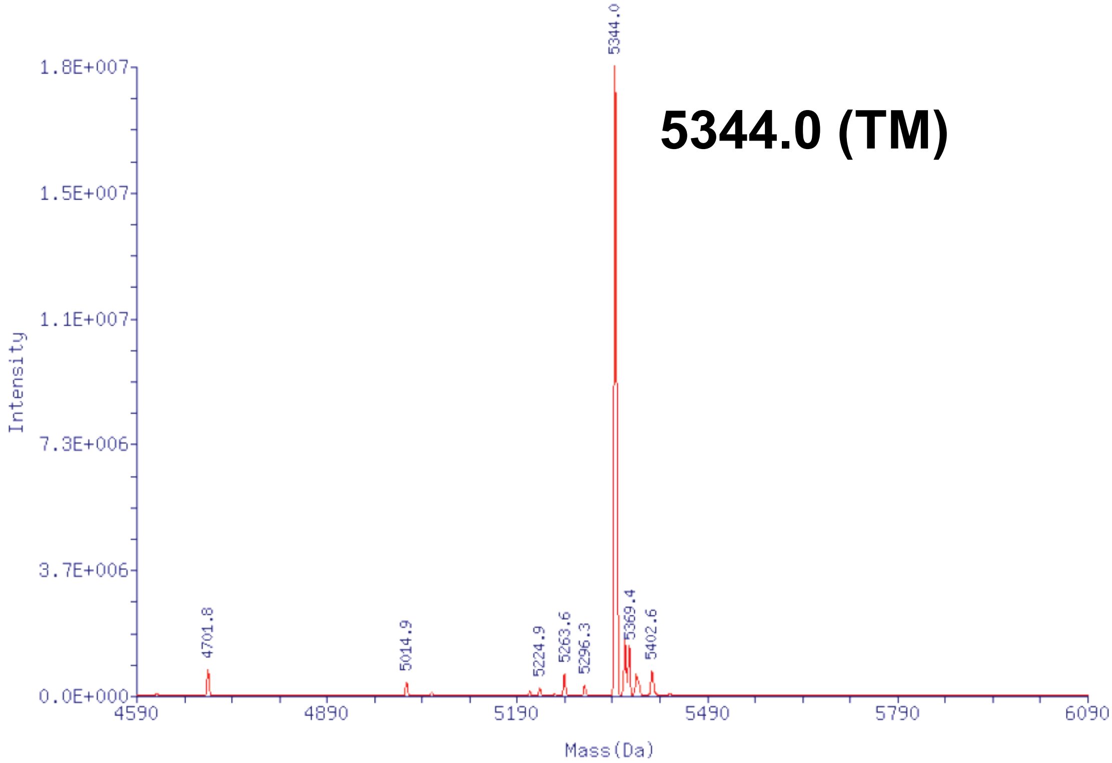

The list of the representative large peptide-based therapeutics involves in CEMBL-approved dataset with low-QED values.

ID	Scaffold	MW	clogP	QED
CHEMBL4297211	ChEMBL_approved	3061.598	5.92058	0.004008
CHEMBL2104391	ChEMBL_approved	5916.927	-13.3793	0.007283
CHEMBL2103758	ChEMBL_approved	3949.455	-23.8251	0.008446
CHEMBL3833353	ChEMBL_approved	4009.507	-23.7342	0.008446
CHEMBL502097	ChEMBL_approved	7594.906	-63.9436	0.00956
CHEMBL525610	ChEMBL_approved	4117.787	-15.2403	0.009576
CHEMBL4084119	ChEMBL_approved	3751.262	-10.1617	0.009577
CHEMBL2103784	ChEMBL_approved	2933.492	-7.96449	0.009579
CHEMBL414357	ChEMBL_approved	4186.637	-20.6043	0.009763
CHEMBL2104987	ChEMBL_approved	3752.14	-16.8852	0.009763
CHEMBL441738	ChEMBL_approved	1646.874	-4.09973	0.009996
CHEMBL4297213	ChEMBL_approved	1706.926	-4.00883	0.01002
CHEMBL1201247	ChEMBL_approved	1269.433	-2.89603	0.010404
CHEMBL1200501	ChEMBL_approved	1329.485	-2.80513	0.010469
CHEMBL4802223	ChEMBL_approved	1305.528	-1.72443	0.011185
CHEMBL387675	ChEMBL_approved	1620.693	-5.6218	0.011297
CHEMBL3085504	ChEMBL_approved	1513.655	-8.35638	0.011554
CHEMBL1201334	ChEMBL_approved	1311.473	-2.08513	0.01194
CHEMBL3545184	ChEMBL_approved	1048.273	-7.22862	0.011996
CHEMBL3545183	ChEMBL_approved	1084.734	-6.80682	0.011999
CHEMBL4594214	ChEMBL_approved	2639.187	-16.2129	0.01265
CHEMBL3989767	ChEMBL_approved	3431.909	-21.5289	0.012654

Theexamples of the representative large peptide-based therapeutics involves in CEMBL-approved dataset with low-QED values.

CHEMBL441738

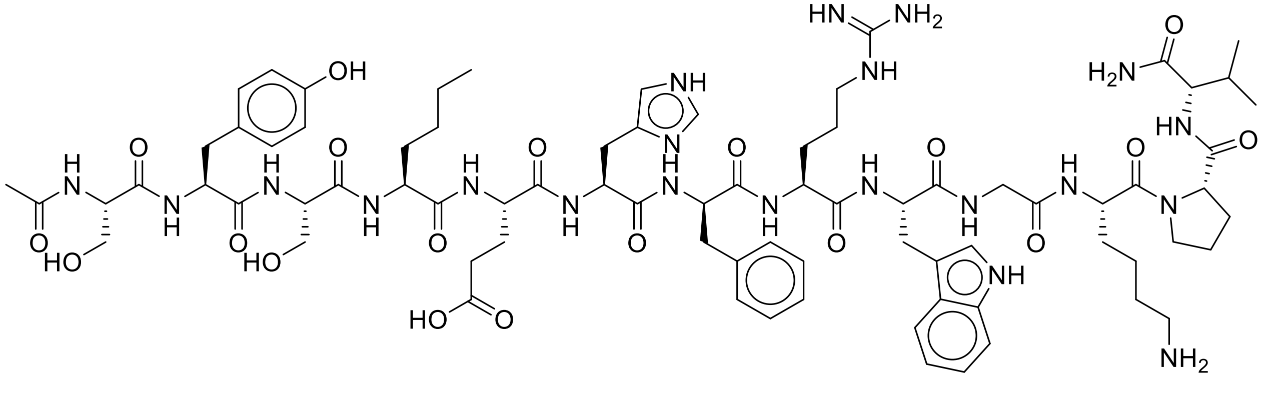

CHEMBL4297213

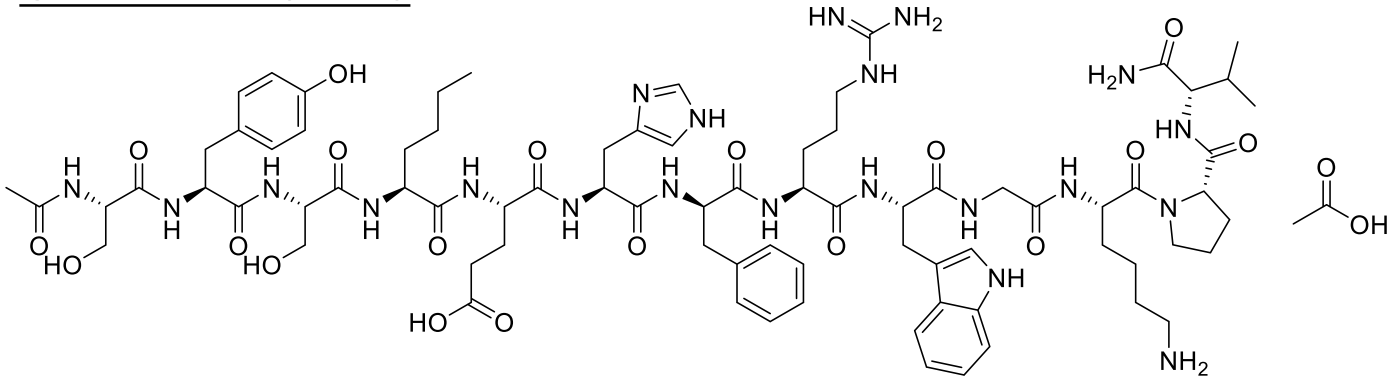

CHEMBL3545183

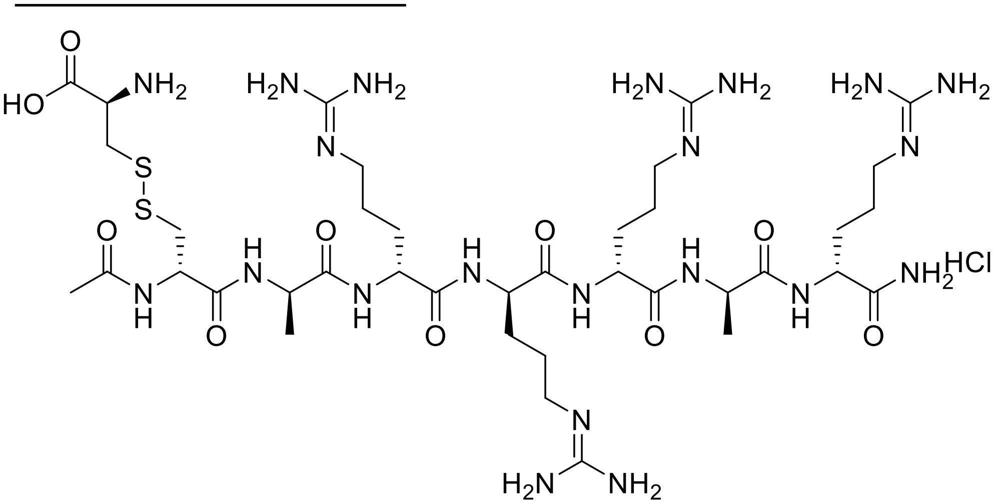

A linear correlation ($r^2 = 0.93$) between the coverage rate (%) and average QED value.

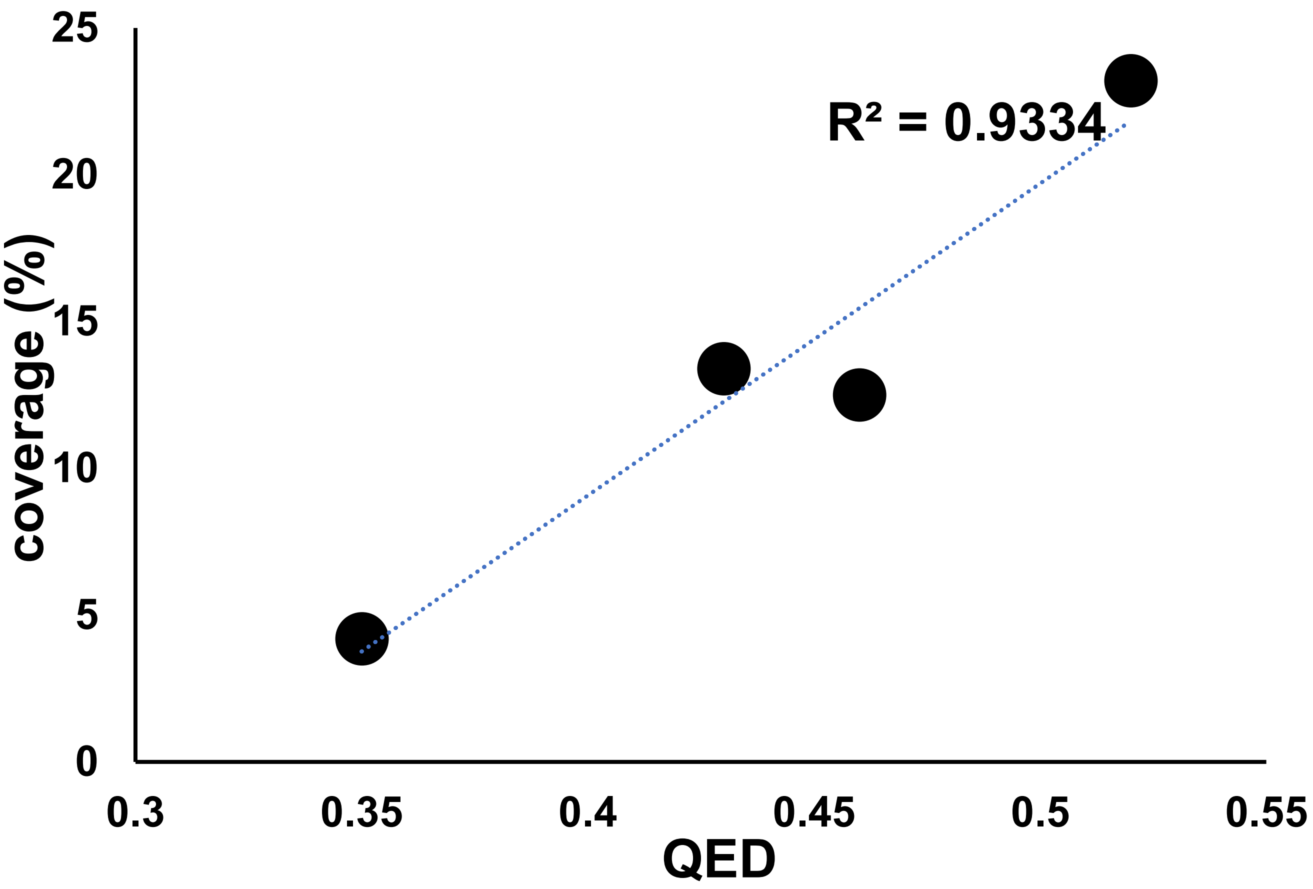

A correlation (r = 0.96) between the proportion of compounds with Ro5VC = 0 and the QED value.

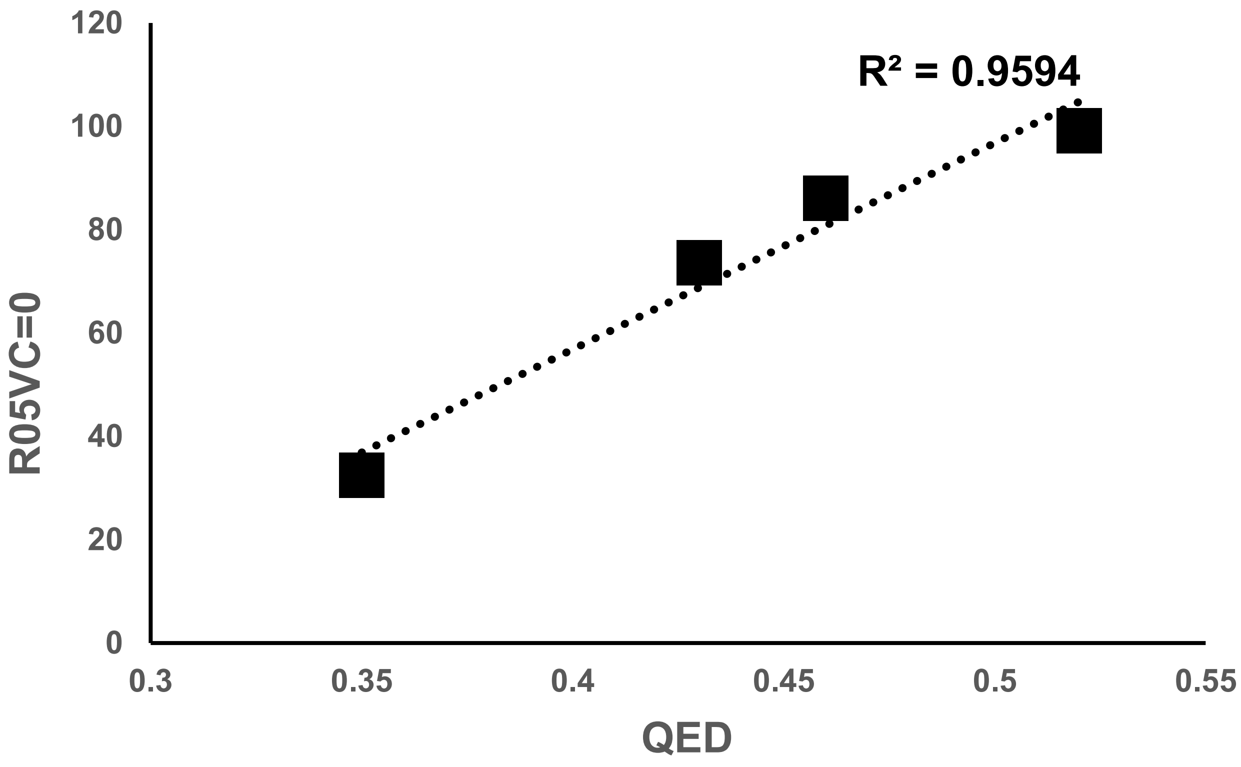

Supplement: Supplementary file 1 [file ijms-27-00828-s001.zip › ijms-4060242-supplementary.pdf]
